# IRAK4 degrader in hidradenitis suppurativa and atopic dermatitis: a phase 1 trial

**DOI:** 10.1038/s41591-023-02635-7

**Published:** 2023-11-13

**Authors:** Lindsay Ackerman, Gerard Acloque, Sandro Bacchelli, Howard Schwartz, Brian J. Feinstein, Phillip La Stella, Afsaneh Alavi, Ashwin Gollerkeri, Jeffrey Davis, Veronica Campbell, Alice McDonald, Sagar Agarwal, Rahul Karnik, Kelvin Shi, Aimee Mishkin, Jennifer Culbertson, Christine Klaus, Bradley Enerson, Virginia Massa, Eric Kuhn, Kirti Sharma, Erin Keaney, Randy Barnes, Dapeng Chen, Xiaozhang Zheng, Haojing Rong, Vijay Sabesan, Chris Ho, Nello Mainolfi, Anthony Slavin, Jared A. Gollob

**Affiliations:** 1 Medical Dermatology Specialists, Phoenix, AZ USA; 2 Encore Medical Research, LLC, Hollywood, FL USA; 3 Encore Medical Research, LLC, Weston, FL USA; 4 CenExel RCA, Hollywood, FL USA; 5 Encore Medical Research, LLC, Boynton Beach, FL USA; 6 TKL Research, Fair Lawn, NJ USA; 7 Mayo Clinic, Rochester, MN USA; 8 Kymera Therapeutics, Inc., Watertown, MA USA

**Keywords:** Translational research, Target validation

## Abstract

Toll-like receptor–driven and interleukin-1 (IL-1) receptor–driven inflammation mediated by IL-1 receptor–associated kinase 4 (IRAK4) is involved in the pathophysiology of hidradenitis suppurativa (HS) and atopic dermatitis (AD). KT-474 (SAR444656), an IRAK4 degrader, was studied in a randomized, double-blind, placebo-controlled phase 1 trial where the primary objective was safety and tolerability. Secondary objectives included pharmacokinetics, pharmacodynamics and clinical activity in patients with moderate to severe HS and in patients with moderate to severe AD. KT-474 was administered as a single dose and then daily for 14 d in 105 healthy volunteers (HVs), followed by dosing for 28 d in an open-label cohort of 21 patients. Degradation of IRAK4 was observed in HV blood, with mean reductions after a single dose of ≥93% at 600–1,600 mg and after 14 daily doses of ≥95% at 50–200 mg. In patients, similar IRAK4 degradation was achieved in blood, and IRAK4 was normalized in skin lesions where it was overexpressed relative to HVs. Reduction of disease-relevant inflammatory biomarkers was demonstrated in the blood and skin of patients with HS and patients with AD and was associated with improvement in skin lesions and symptoms. There were no drug-related infections. These results, from what, to our knowledge, is the first published clinical trial using a heterobifunctional degrader, provide initial proof of concept for KT-474 in HS and AD to be further confirmed in larger trials. ClinicalTrials.gov identifier: NCT04772885.

## Abstract (web summary)

The heterobifunctional degrader KT-474 shows target engagement and is safe to use in healthy volunteers and in patients with hidradenitis suppurativa and patients with atopic dermatitis, and it exhibits preliminary efficacy in the improvement of skin lesions and symptoms in patients.

## Main

Toll-like receptors (TLRs) and interleukin-1 receptors (IL-1Rs) mediating cellular activation by TLR agonists and IL-1 family cytokines^1,2^, respectively, have been implicated in multiple autoimmune diseases, including the skin diseases hidradenitis suppurativa (HS) and atopic dermatitis (AD)^3–9^. Most TLRs and all IL-1Rs signal through the myddosome, a complex of proteins that includes IL-1 receptor–associated kinase 4 (IRAK4), which is assembled upon activation of MYD88 (refs. ^10,11^). A master regulator of innate immunity, IRAK4 has scaffolding and kinase functions that are essential for downstream signaling through nuclear factor (NF)-κB, mitogen-activated protein (MAP) kinases and IRF5 and IRF7 (refs. ^12,13^). The scaffolding function of IRAK4 is required for proper assembly of the myddosome and activation of NF-κB and MAP kinases, leading to the induction of proinflammatory cytokines and chemokines^14,15^.

Clinical validation for targeting the TLR/IL-1R pathway in autoimmune diseases has come from the activity of antibodies targeting different IL-1 family cytokines in diseases, such as cryopyrin-associated autoinflammatory syndromes (IL-1) (ref. ^16^), rheumatoid arthritis (RA; IL-1) (ref. ^17^), generalized pustular psoriasis (IL-36) (ref. ^18^), macrophage activation syndrome (IL-18) (ref. ^19^), HS (IL-1) (ref. ^20^) and asthma (IL-33) (ref. ^21^). IRAK4 targeting would have the advantage of inhibiting signaling by all IL-1 family cytokines as well as by almost all TLRs with a single drug, and, to that end, IRAK4 kinase inhibitors have been in development for RA, HS and lupus^22–24^. Although modest activity was shown in RA in a randomized phase 2 trial with an IRAK4 kinase inhibitor, PF-06650833 (ref. ^25^), that same drug did not show activity compared to placebo in a phase 2 trial in HS^26^, suggesting that efficacy may be limited when IRAK4 targeting does not address the kinase and scaffolding functions of the protein.

Targeted protein degradation involves the use of heterobifunctional small molecules to bring an E3 ligase together with a protein of interest to mediate its ubiquitination and degradation by the proteasome^27^. This co-opting of the ubiquitin–proteasome system can be applied to targets that are undruggable by small molecules or to targets such as IRAK4 that have both kinase and scaffolding functions that require removal of the protein. KT-474 (SAR444656) is a selective small-molecule degrader of IRAK4 in development for the treatment of TLR/IL-1R–driven autoimmune diseases. Here we report the results of the first-in-human phase 1 trial of KT-474, showing on-target proof of mechanism and functional pathway inhibition in addition to initial clinical proof of concept in patients with HS and patients with AD.

## Results

### KT-474 is a potent and selective degrader of IRAK4

**Extended Data Fig. 1:**
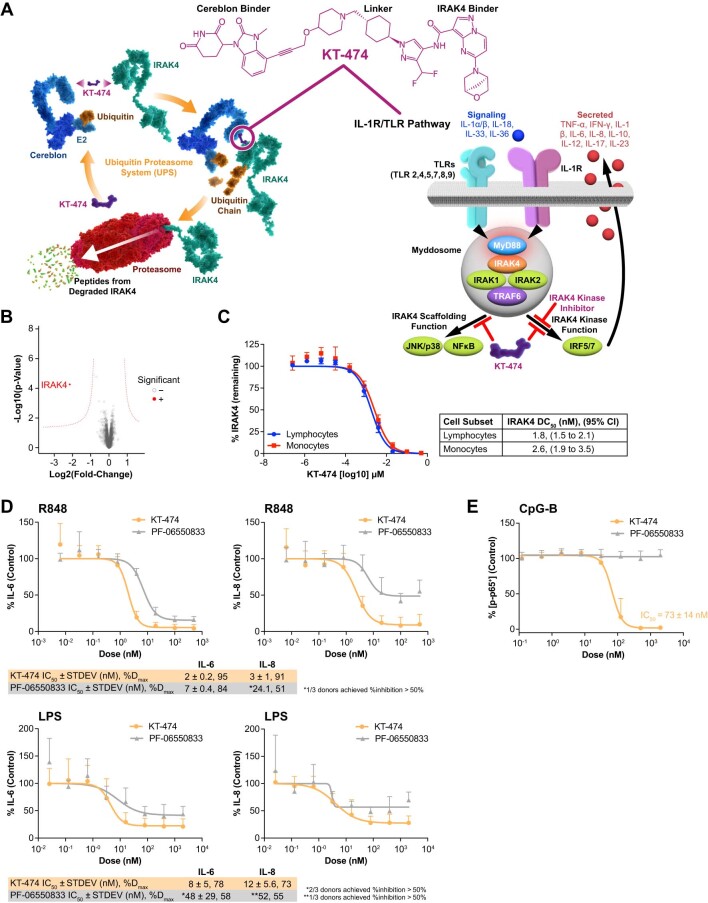
Identification of KT-474 (SAR444656) as a potent and selective degrader of IRAK4. **(a)** KT-474 chemical structure, mechanism of action and potential advantage over IRAK4 kinase inhibitors on inhibition of TLR–/IL-1R–driven myddosome signaling through its effects on scaffolding and kinase functions of IRAK4. **© 2023 Kymera Therapeutics, Inc. (b)** Highly selective degradation of IRAK4 by KT-474 in human PBMCs determined by discovery proteomics. A paired statistical analysis was performed using linear models and significant degradation was determined by application of a weighted cutoff incorporating both significance and fold-change. **(c)** Potent degradation of IRAK4 by KT-474 in lymphocyte and monocyte subsets of PBMCs using N = 3 biologically independent donors over 1 experiment. **(d**) Superior inhibition of IL-6 and IL-8 production in R848- or LPS-stimulated PBMCs by KT-474 compared to the IRAK4 kinase inhibitor PF-06550833 using N = 3 biologically independent donors and 2 technical replicates over 1 experiment. **(e)** Inhibition of NF-kB activation (phospho-p65) in CpG-B stimulated B cells by KT-474 but not PF-06550833 using N = 5 biologically independent donors over 2 experiments. All data in (c) through (e) are graphed as mean values +/- STDEV. CI = confidence interval; IC50 = concentration of a drug needed to inhibit a biological process or response by 50%; IL = interleukin; LPS = lipopolysaccharide; PBMCs = peripheral blood mononuclear cells; R = receptor; STDEV = standard deviation; TLR = toll-like receptor. Source data

KT-474 is a heterobifunctional small-molecule degrader of IRAK4 composed of ligands to the E3 ligase cereblon (CRBN) and IRAK4 joined by a chemical linker (Extended Data Fig. 1a). KT-474 forms a ternary complex with CRBN and IRAK4, leading to the ubiquitination and proteasomal degradation of IRAK4. The elimination of IRAK4 from the myddosome using a degrader has the potential to block all downstream signaling, thereby inhibiting TLR-mediated and IL-1R-mediated cellular activation and cytokine induction. Proteomics in human peripheral blood mononuclear cells (PBMCs) showed that KT-474 is selective for IRAK4 (Extended Data Fig. 1b) and that CRBN engagement by KT-474 does not lead to degradation of any of the known immune-mediated inflammatory disease substrates, such as Ikaros, Aiolos or SALL4. KT-474 potently degraded IRAK4 in lymphocytes and monocytes with the half-maximal degradation concentration in the 1–2 nM range and a maximum degradation of 100% (Extended Data Fig. 1c). KT-474 was compared to the IRAK4 kinase inhibitor PF-06550833 in functional assays examining the response to TLR agonists. KT-474 exhibited more potent and stronger downregulation of both R848 (TLR7/8)-induced and lipopolysaccharide (LPS: TLR4)-induced IL-6 and IL-8 production by PBMCs (Extended Data Fig. 1d). KT-474 also blocked TLR9-mediated NF-κB activation by CpG-B in B cells, whereas PF-06550833 had no effect (Extended Data Fig. 1e).

### Baseline characteristics of study participants

**Fig. 1:**
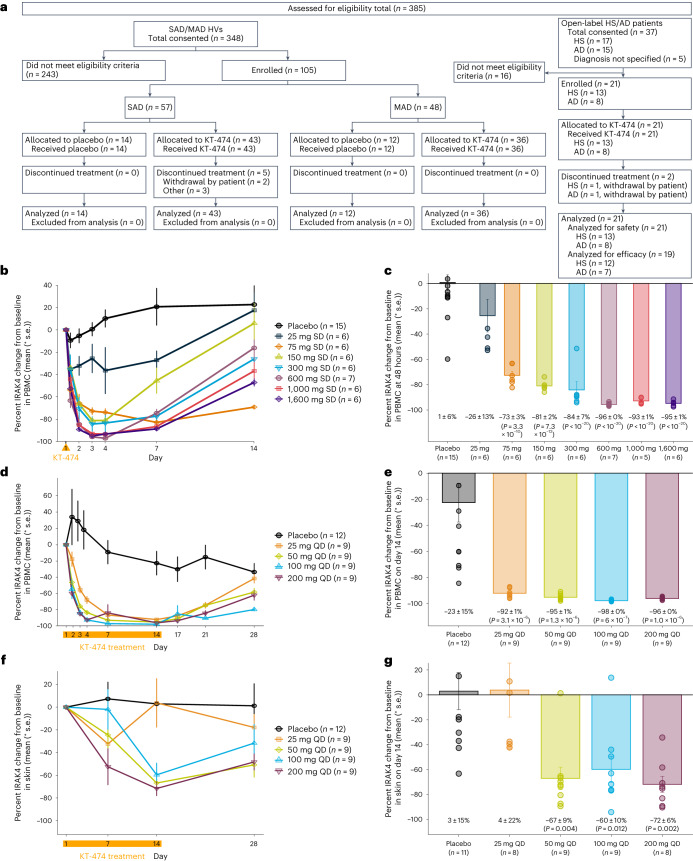
CONSORT diagram and IRAK4 degradation in blood and skin of HVs treated with KT-474 measured by targeted mass spectrometry. **a**, CONSORT diagram for phase 1 trial of KT-474. **b**, Mean (±s.e.) IRAK4 degradation over time in PBMCs after single dose of KT-474 on day 1 (dosing indicated by orange arrow, *n* = 6 per dose group except 600 mg SD (*n* = 7) and placebo (*n* = 15)). **c**, Mean (±s.e.) IRAK4 degradation in PBMCs at 48 h after single dose (*n* = 6 per dose group except 600 mg SD (*n* = 7) and placebo (*n* = 15)). **d**, Mean (±s.e.) IRAK4 degradation over time in PBMCs over 14 d of dosing with KT-474 (dosing period indicated by orange bar, *n* = 9 per dose group except placebo (*n* = 12)). **e**, Mean (±s.e.) IRAK4 degradation in PBMCs at steady-state nadir on day 14 (*n* = 9 per dose group except placebo (*n* = 12)). **f**, Mean (±s.e.) IRAK4 degradation over time in skin biopsies over 14 d of dosing with placebo (*n* = 14), 25 mg QD (*n* = 8), 50 mg QD (*n* = 9), 100 mg QD (*n* = 9) and 200 mg QD (*n* = 8). **g**, Mean (±s.e.) IRAK4 degradation in skin on day 14 (*n* = 11 for placebo, *n* = 8 for 25 mg QD and 200 mg QD, *n* = 9 for 50 mg QD and 100 mg QD). Treatment groups were compared to placebo (number of patients per group mentioned above for each subpanel) using one-way ANOVA models with Tukey correction for multiple comparisons. QD, once daily; SD, single dose. Source data

Between 4 February 2021 and 7 September 2022, 105 healthy volunteers (HVs) were enrolled into the placebo-controlled single and multiple ascending dose escalation cohorts (SAD and MAD), and 21 HS and AD patients were enrolled into the open-label patient cohort (Fig. 1a). The last patient visit was 20 October 2022 for trial completion. Extended Data Table 1 and Table 1 show the baseline characteristics for HVs and for patients, respectively. Most patients with HS and patients with AD had moderate disease severity and were not previously treated with systemic biologic therapies. Among patients, there were two early withdrawals for reasons unrelated to the study after 4–5 doses (one HS and one AD), leaving 12 patients with HS and seven patients with AD who completed treatment and follow-up through end of study.

**Table 1:** Baseline characteristics of patients with HS or AD

	HS (*n* = 13)	AD (*n* = 8)
Sex, *n* (%)		
Female	10 (76.9)	3 (37.5)
Male	3 (23.1)	5 (62.5)
Age (years)		
Median (range)	40.0 (21 – 53)	31.0 (23 – 55)
Disease severity at baseline, *n* (%)	HS-PGA	vIGA-AD
Mild	0	1 (12.5)
Moderate	10 (76.9)	5 (62.5)
Severe	1 (7.7)	2 (25.0)
Very severe	2 (15.4)	0
Extent of disease at baseline mean (min, max)		
AN count	8.4 (5, 18)	-
Fistula count	3.8 (0, 15)	-
Pain NRS (worst over past 24 h)	5.5 (0, 10)	-
Pain NRS (worst over past week)	6.8 (3, 10)	-
Pruritus NRS (worst over 24 h)	3.3 (0, 9)	7.1 (2, 10)
Pruritus NRS (worst over past week)	5.2 (0, 10)	7.6 (4, 10)
EASI score	-	17.6 (4.4, 52.3)
Patients with any prior therapy, *n* (%)		
Antibiotics/antibacterials	5 (38.5)^a^	1 (12.5)
Corticosteroids	0	7 (87.5)
Adalimumab	3 (23.1)	0
Other biologics	1 (7.7)	0

^a^ Includes two topical medications that are not coded as antibiotics (clindamycin and chlorhexidine).

vIGA-AD, validated Investigator Global Assessment for atopic dermatitis.

### Safety and pharmacokinetics

**Extended Data Fig. 2:**
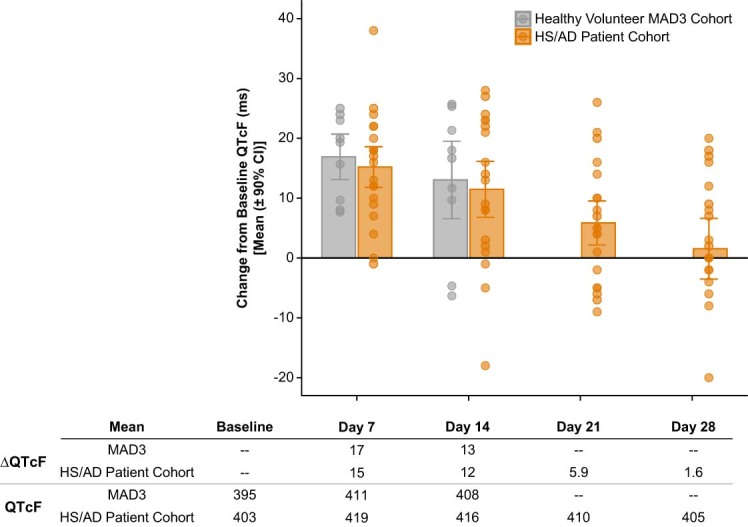
QTcF prolongation in healthy volunteer MAD cohort 3 (MAD3; 100 mg) and in open-label HS/AD patient cohort. Mean change from baseline (ΔQTcF) and absolute QTcF values are shown at the indicated time points for MAD3 (100 mg daily fasted x 14 days, n = 9) and open-label patient cohort (75 mg daily fed x 28 days, n = 20). AD = atopic dermatitis; CI = confidence interval; HS = hidradenitis suppurativa; MAD = multiple ascending doses; QTcF = corrected QT interval by Fredericia. Source data

Single doses of KT-474 up to 1,600 mg and 14 daily doses up to 200 mg administered in the fasted state were well tolerated in HVs, with no dose-limiting toxicities and no serious adverse events (SAEs). The most common adverse event (AE) observed with KT-474 in SAD and MAD was mild to moderate headache (Extended Data Tables 2 and 3). Less frequent mild to moderate AEs seen in more than one patient included nausea, vomiting and diarrhea and palpitations. The palpitations, reported in 4.6% (2/43) of SAD and 8.3% (3/36) of MAD patients, were predominantly single episodes that were not associated with arrhythmias and did not lead to interruption of dosing. There were no drug-related infections. Across all four MAD cohorts, delayed corrected QT interval by Fredericia (QTcF) prolongation was observed at day 7, with a mean increase from baseline of 19.7 ms in MAD cohort 3 (Extended Data Fig. 2). This QTcF prolongation was asymptomatic and non-adverse (ΔQTcF < 60 ms, absolute QTcF ≤ 450 ms). It decreased modestly with continued dosing between days 7 and 14 and then returned to baseline after the drug was stopped.

The safety profile in patients with HS and patients with AD who were treated with 75 mg of KT-474 daily for 28 d administered in the fed state was similar to that in HVs, with no drug-related infections (Table 2). As in HVs, non-adverse QTcF prolongation was seen in patients on days 7–14 but then resolved back to baseline with continued dosing by day 28 (Extended Data Fig. 2).

**Table 2:** TEAEs by preferred term in patients with HS or AD and total

	HS (*n* = 13)	AD (*n* = 8)	Total (*n* = 21)	HS (*n* = 13)	AD (*n* = 8)	Total (*n* = 21)
Any TEAE, *n* (%)	10 (76.9)	6 (75.0)	16 (76.2)	–	–	–
Preferred Term	TEAE any cause, *n*			Related TEAE, *n*		
Headache	7	3	10	4	2	6
Diarrhea	5	0	5	2	0	2
Fatigue	4	0	4	4	0	4
Dizziness	2	0	2	0	0	0
Myalgia	1	1	2	0	0	0
Nausea	1	1	2	0	1	1
Palpitations	2	0	2	1	0	1
Blood creatine phosphokinase increased	1	0	1	0	0	0
Costochondritis	1	0	1	0	0	0
Decreased appetite	1	0	1	0	0	0
Eye pruritus	1	0	1	1	0	1
Hidradenitis	1	0	1	0	0	0
Hot flush	0	1	1	0	0	0
Hypersomnia	0	1	1	0	0	0
Migraine	1	0	1	1	0	1
Muscular weakness	1	0	1	1	0	1
Nasopharyngitis	1	0	1	0	0	0
Procedural pain	1	0	1	0	0	0
Swelling of eyelid	1	0	1	1	0	1
Tic	1	0	1	0	0	0
Urinary tract infection	1	0	1	0	0	0

TEAE, treatment-emergent adverse event.

**Extended Data Fig. 3:**
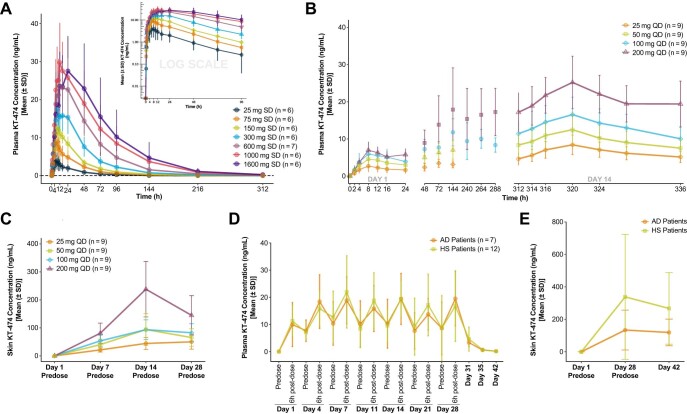
Pharmacokinetics of KT-474 in plasma and skin in healthy volunteers (HVs) and patients with atopic dermatitis (AD) and hidradenitis suppurativa (HS). Mean ( ± standard deviation [SD]) plasma concentration-time profiles for KT-474 following ascending single doses **(a)** and ascending multiple once daily doses x 14 days **(b)** of KT-474 in HVs. (**c**) Mean concentration-time profiles for KT-474 in skin following ascending multiple once daily doses x 14 days of KT-474 in HVs. Mean concentration-time profiles for KT-474 in plasma **(d)** and skin lesions **(e)** following once daily doses of KT-474 75 mg x 28 days in patients with AD (n = 7) and HS (n = 12). QD = once daily. Source data

Plasma pharmacokinetics (PK) in HVs after a single dose of KT-474 showed dose-dependent exposure increases, plateauing after the 1,000-mg dose with a maximum concentration (C_max_) of 7–24 h, a half-life of 25–40 h and low to moderate inter-patient variability (Extended Data Fig. 3a). Plasma PK with multi-dosing in HVs also showed dose-dependent exposure increases, with steady state reached by day 7 and a 3–4-fold increase in exposure on day 14 compared to day 1 (Extended Data Fig. 3b). Skin PK with multi-dosing in HVs showed dose-dependent exposure increases that did not appear to plateau by day 14 (Extended Data Fig. 3c), with trough concentration (C_trough_) levels in skin that were approximately 10–14-fold higher than plasma. KT-474 appeared to clear slowly from the skin 2 weeks after completion of dosing. There was negligible renal elimination of KT-474 with ≤1% of the administered dose excreted as unchanged drug in urine.

In patients, steady-state plasma C_max_ and C_trough_ at 75 mg dosed in the fed state were similar to what was observed in HVs dosed with 100 mg in the fasted state (Extended Data Fig. 3d). C_trough_ levels in biopsies of skin lesions at day 28 were also substantially higher than levels in plasma (Extended Data Fig. 3e).

### Pharmacodynamic and biomarker analyses

A single dose of KT-474 resulted in rapid, dose-dependent degradation of IRAK4 in PBMCs of HVs in SAD, starting with the lowest dose level of 25 mg and plateauing after 600 mg (Fig. 1b). IRAK4 reduction was seen as early as 4–8 h after dose, reached its nadir by 48 h and showed early signs of recovery 4 d later. Recovery continued through the end of follow-up on day 14. At the nadir, mean IRAK4 reduction was 93–96% at doses ≥600 mg with minimal inter-patient variability (*P* < 0.001; Fig. 1c). In MAD, lower doses administered daily for 14 d resulted in robust IRAK4 degradation in PBMCs, even at the lowest dose of 25 mg, with steady-state reduction observed by day 7 (Fig. 1d). At the nadir, mean IRAK4 reduction was 92% at 25 mg and 95–98% at 50–200 mg (*P* < 0.001; Fig. 1e). In skin, dose-dependent IRAK4 degradation was seen on day 7 with further reduction on day 14 (Fig. 1f). Mean reduction at 50–200 mg on day 14 was 60–72% (*P* < 0.05; Fig. 1g).

**Extended Data Fig. 4:**
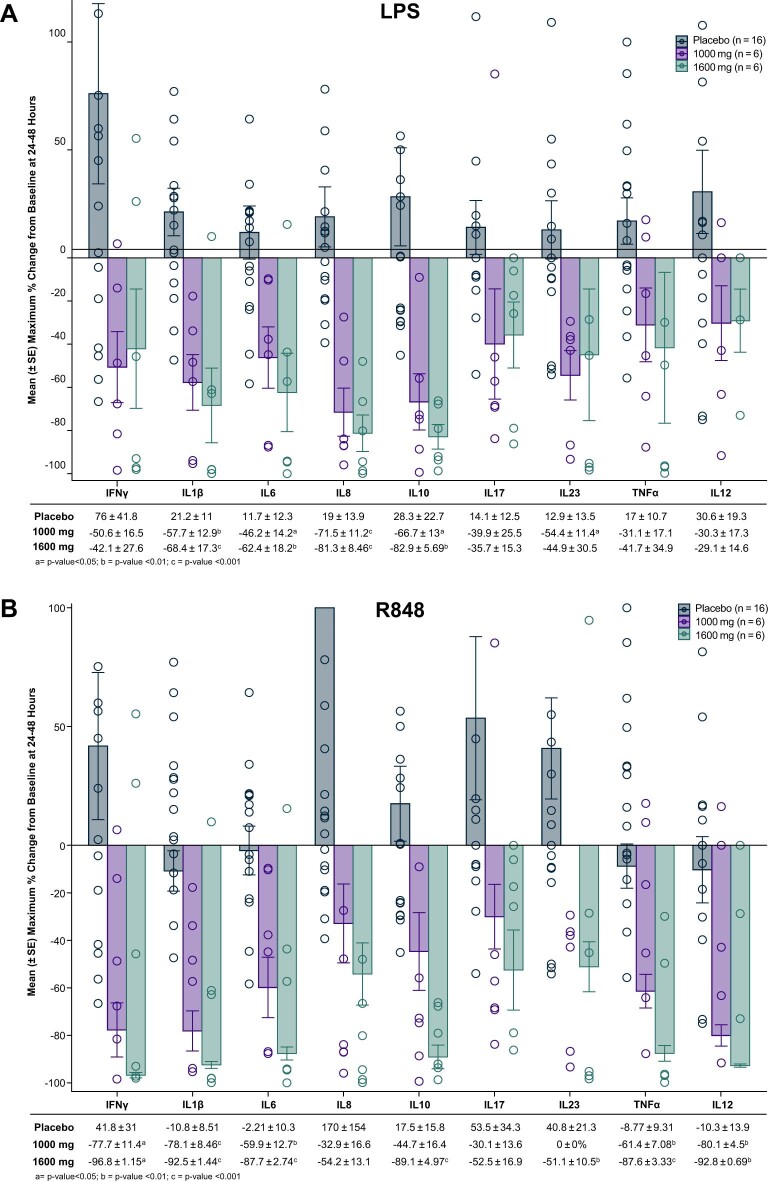
Inhibition of TLR-stimulated ex vivo cytokine production in blood of healthy volunteers treated with a single dose of KT-474. Maximum change from baseline at 24–48 hours post-dose in cytokine induction by TLR7/8 agonist R848 (**a**) or TLR4 agonist LPS (**b**) in healthy volunteers treated with placebo (n = 16), 1000 mg SD (n = 6) and 1600 mg SD (n = 6). Treatment groups were compared to placebo using one-way analysis of variance (ANOVA) models with Tukey correction for multiple comparisons. LPS = lipopolysaccharide; SAD = single ascending dose; SD = single dose; SE = standard error. Source data

The functional impact of more than 90% IRAK4 degradation in PBMCs was evaluated using an ex vivo TLR stimulation assay on whole blood. At the 1,000-mg and 1,600-mg SAD dose levels, the induction by LPS and R848 of a broad array of cytokines involved in innate immunity, as well as Th1, Th17 and Th2 responses, was inhibited 24–48 h after dose (Extended Data Fig. 4a,b). Inhibition by more than 80% at the 1,600-mg SAD dose level was seen for LPS induction of IL-8 and IL-10 and for R848 induction of IFN-γ, IL-1β, IL-6, IL-10, tumor necrosis factor-alpha (TNF-α) and IL-12.

**Fig. 2:**
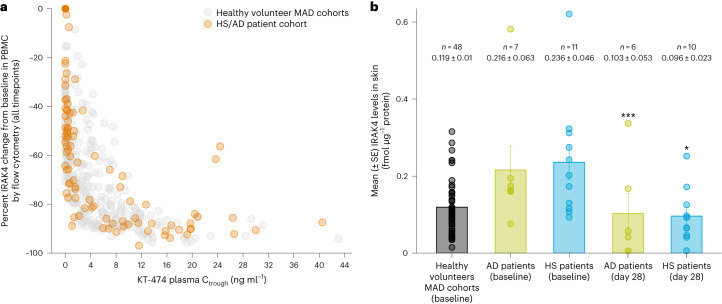
IRAK4 reduction by KT-474 in blood and skin lesions of patients with HS or AD. **a**, Relationship between IRAK4 knockdown in PBMCs and plasma KT-474 trough levels in HV MAD cohorts and the HS/AD patient cohort. **b**, Mean (±s.e.) IRAK4 levels in skin of patients with AD (*n* = 7) and patients with HS (*n* = 11) at baseline compared to HVs (MAD1–4 *n* = 48) and after 28 d (*n* = 6 for AD and *n* = 10 for HS) of KT-474 dosing. IRAK4 levels on day 28 were compared to baseline using one-way ANOVA models. *P = 0.012 and ***P = 0.0009. Source data

IRAK4 degradation in PBMCs of patients with HS and patients with AD treated with KT-474 for 28 d was similar to what was achieved in the HV MAD cohorts treated for 14 d, with an IRAK4 nadir of more than 90% and a similar relationship of plasma KT-474 C_trough_ to IRAK4 knockdown (Fig. 2a). In both HVs and patients, plasma C_trough_ > 3 ng ml^−1^ was associated with more than 80% IRAK4 degradation. The levels of IRAK4 in skin lesions of patients with HS and patients with AD was approximately twofold higher compared to the level in the skin of HVs. After 28 d of KT-474, IRAK4 was reduced by more than 50%, with normalization of IRAK4 expression to the level seen in HVs (*P* < 0.05; Fig. 2b).

Both patients with HS and patients with AD showed systemic signs of inflammation with elevation of plasma biomarkers of inflammation, including the proinflammatory cytokines IL-6 and IL-1β and the acute phase reactant C-reactive protein (CRP). In patients with HS, KT-474 treatment resulted in 48–63% reductions across the three analytes (Extended Data Table 4). In patients with AD, CRP was not elevated at baseline, but, after KT-474 treatment, IL-1β and IL-6 showed 36% and 56% reductions, respectively.

**Fig. 3:**
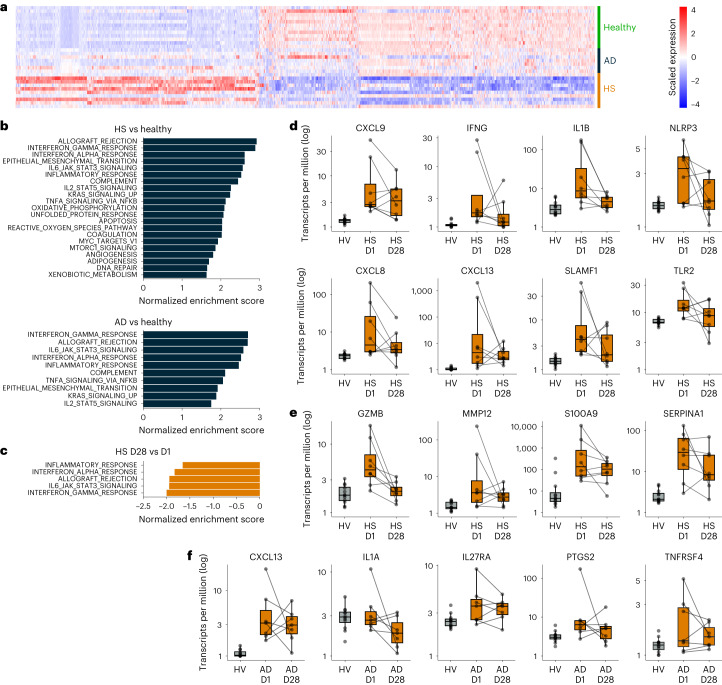
RNA-seq analysis of skin lesion biopsies in patients with HS (*n* = 8) or AD (*n* = 7) taken before and after KT-474 treatment compared to HV skin (*n* = 12). **a**, Heat map showing differentially expressed genes (FDR *q* < 0.05, absolute fold change ≥ 2) between HVs from MAD3 and patients with HS and patients with AD pre-treatment. **b**, Normalized enrichment scores for pathways that were significantly upregulated (FDR *q* < 0.01) pre-treatment in patients with HS and patients with AD compared to HVs. **c**, Normalized enrichment scores for pathways that were significantly downregulated (FDR *q* < 0.01) at day 28 versus day 1 pre-dose in patients with HS. **d**, Proinflammatory markers at day 1 pre-dose (HS D1) and day 28 (HS D28) in patients with HS with comparison to pre-treatment expression in HVs. **e**, Complement pathway markers at day 1 pre-dose (HS D1) and day 28 (HS D28) in patients with HS with comparison to pre-treatment expression in HVs. **f**, Proinflammatory markers at day 1 pre-dose (AD D1) and day 28 (AD D28) in patients with AD with comparison to pre-treatment expression in HVs. In **d**–**f**, lines connect measurements from the same patient at day 1 pre-dose and day 28. Boxes show median and interquartile range. The upper whisker extends to the largest value no further than 1.5× interquartile range. The lower whisker extends to the smallest value at most 1.5× interquartile range. MAD3, MAD cohort 3. Source data

Serial biopsies of skin lesions performed at day 1 pre-dose and day 28 were evaluable for RNA sequencing (RNA-seq) analysis in eight of 12 patients with HS and in all seven patients with AD who completed sample collections in the study. Pre-dose transcriptomes from the HS and AD patient biopsies were compared to pre-dose skin biopsies from HVs in MAD cohort 3 (*n* = 12) to identify differentially expressed genes in each disease context (Fig. 3a). A total of 955 genes were significantly upregulated and 1,309 genes were significantly downregulated in HS biopsies compared to HVs. Fewer genes were significantly changed in AD (51 upregulated and 22 downregulated), but, as seen in the heat map, most genes that were significantly changed in patients with HS showed similar trends in patients with AD. Gene set enrichment analysis (GSEA) was applied to this transcriptomic dataset to identify pathways that were significantly upregulated in patients with HS and patients with AD relative to HVs (Fig. 3b). Because GSEA does not apply a statistical cutoff but looks at transcriptome-wide trends in a set of genes, it was possible to identify significantly upregulated pathways in both sets of patients, and these pathways showed a high degree of overlap. In particular, pathways upregulated in both diseases included inflammatory response, interferon gamma response, interferon alpha response, complement, allograft rejection and interleukin-6 (IL-6)/Janus kinase (JAK)/signal transducer and activator of transcription 3 (STAT3) signaling.

Although standard differential gene expression analysis did not identify any statistically changed genes between day 1 pre-dose and day 28, GSEA was also applied to identify significantly downregulated pathways. Inflammatory response, interferon gamma response, interferon alpha response, allograft rejection and IL-6/JAK/STAT3 signaling were all downregulated significantly in patients with HS at day 28 relative to day 1 pre-dose (Fig. 3c). Complement showed a similar trend of downregulation but did not reach statistical significance. In patients with AD, there were no significantly downregulated pathways at day 28 relative to day 1. Select genes in the inflammatory response and complement pathways with a trend of downregulation in patients with HS at day 28 compared to day 1 pre-dose are shown in Fig. 3d,e. The levels of these genes in HVs are also shown as a reference. Genes that showed a trend of downregulation included classic inflammatory markers, such as IFN-γ (IFNG), IL-8 (CXCL8), IL-1β (IL1B) and NLRP3, as well as CXCL13 and TLR2. Markers of cytotoxicity upregulated in patients with HS, such as granzyme B (GZMB) and MMP12, also showed a trend toward downregulation with KT-474 treatment. Notably, these genes were most downregulated in patients who had the highest levels pre-treatment. Similar trends were seen for proinflammatory markers CXCL13, IL-1α (IL1A), IL-27 receptor alpha (IL27RA), COX2 (PTGS2) and OX40 (TNFRSF4) in AD patient biopsies (Fig. 3f).

### Patient outcomes

Clinical responses were assessed in patients with HS and patients with AD during the 28-d dosing period and subsequent 2-week follow-up through day 42.

**Fig. 4:**
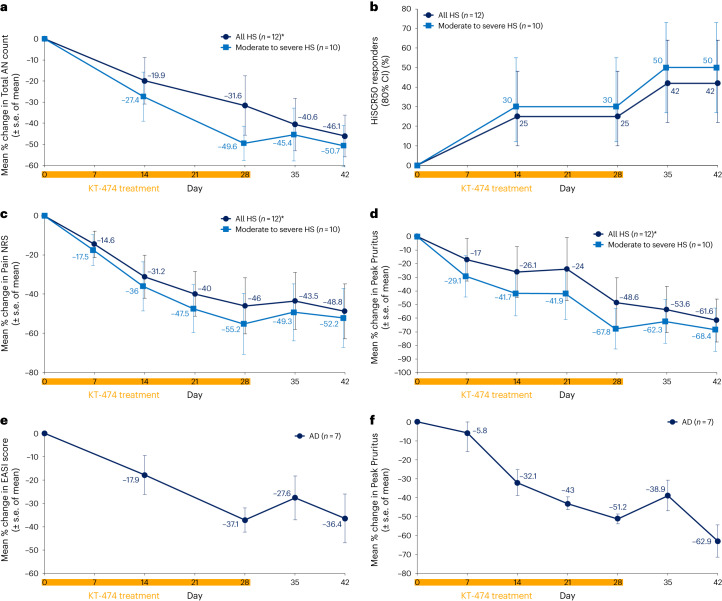
Clinical responses to KT-474 in patients with HS or AD. **a**–**f**, Improvement in skin lesions and symptoms in patients with HS (**a**–**d**) and patients with AD (**e**–**f**) treated with KT-474 for 28 d. CI, confidence interval. *One patient was censored for day 35 and day 42 because the patient was started on ustekinumab, steroids and antibiotics on day 34. Source data

**Extended Data Fig. 5:**
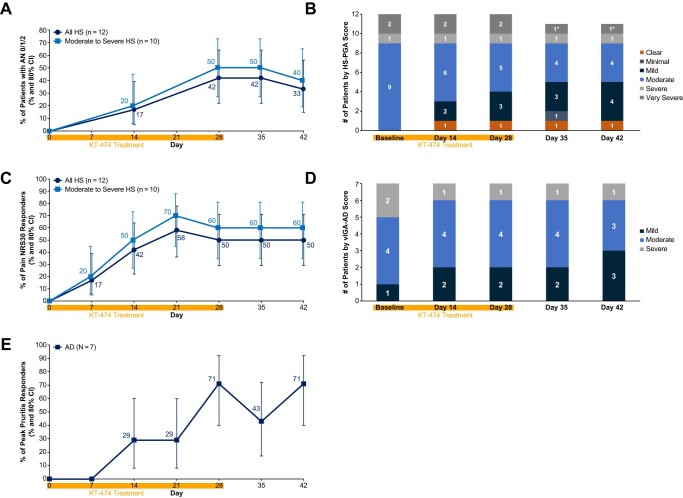
Clinical responses to KT-474 in hidradenitis suppurativa (HS) and atopic dermatitis (AD) patients. Improvement in skin lesions and symptoms in HS **(a-c)** and AD **(d-e)** patients treated with KT-474 for 28 days. Data are presented as % of responders and the 80% confidence interval of the % (a, c, and e). AN = abscess and nodule count; CI = confidence interval; HS-PGA = hidradenitis suppurativa Physician’s Global Assessment; NRS = Numerical Rating Scale; vIGA-AD = validated Investigator Global Assessment for AD. *One patient was censored for Day 35 and Day 42 because the patient started on ustekinumab, steroids and antibiotics on Day 34. Source data

For HS, the analysis was performed for all patients (*n* = 12), including two patients with very severe disease who had progressed after prior biologics, as well as for those patients with moderate to severe disease (*n* = 10). Overall, both analyses yielded similar results, with responses for the group gradually evolving over the 28 d of dosing before then being generally maintained or continuing to improve during the subsequent 2-week follow-up period. There was a 46.1–50.7% reduction in abscess and nodule (AN) count (maximum 100% reduction) and an HS Clinical Response 50% (HiSCR50) reduction rate of 42–50% (Fig. 4a,b) as well as an AN 0/1/2 rate of 42–50% (Extended Data Fig. 5a). This corresponded to an improvement in HS Physician’s Global Assessment (HS-PGA) in five of 12 patients, including complete clearance of lesions in a patient with moderate disease severity and stable HS-PGA in all remaining patients (Extended Data Fig. 5b). Pain is a major symptom in HS, impacting quality of life and how patients feel and function^28,29^. There was a 48.8–55.2% reduction in Pain Numerical Rating Scale (NRS) (Fig. 4c) and a 50–60% Pain NRS30 (at least a 30% reduction and one unit reduction from baseline) response rate (Extended Data Fig. 5c). Pruritus is a substantial problem in patients with HS^30^, and KT-474 also impacted this symptom with a 61.6–68.4% reduction in Peak Pruritus (Fig. 4d).

Patients with AD, like patients with HS, also showed a pattern of response characterized by evolution over the dosing period followed by maintenance or continued improvement during follow-up. There was a 37.1% reduction in Eczema Area and Severity Index (EASI) score (maximum 76% reduction; Fig. 4e), with validated Investigator Global Assessment for AD improving in two patients and remaining stable in the others (Extended Data Fig. 5d). Pruritus is one of the primary symptoms affecting quality of life in patients with AD^31,32^. Peak Pruritus declined by 62.9% (Fig. 4f), with a Peak Pruritus response rate of 71% (Extended Data Fig. 5e).

## Discussion

IRAK4 is a promising target for the treatment of TLR/IL-1R–driven skin, gastrointestinal and rheumatic autoimmune diseases, but the potential of interrupting this pathway has yet to be realized. We developed a potent, selective, orally administered heterobifunctional IRAK4 degrader, KT-474 (SAR444656), which demonstrated consistent effects on target expression, biomarkers of inflammation and clinical endpoints in a phase 1 placebo-controlled trial in HVs with an open-label cohort of patients with HS and patients with AD. To our knowledge, this is the first published clinical trial using a heterobifunctional degrader.

Single doses of KT-474 led to rapid, profound degradation of IRAK4 that persisted for days in PBMCs, consistent with its catalytic mechanism of action and potency. This persistent degradation enabled substantially lower doses of KT-474 to achieve more than 90% IRAK4 degradation with daily dosing; a steady-state plasma C_trough_ of only 3 ng ml^−1^ was required to maintain more than 80% IRAK4 reduction. Notably, there was strong fidelity of translation of the PK–pharmacodynamic (PD) relationship in blood from HVs to patients with HS and patients with AD. KT-474 also achieved 60–70% IRAK4 degradation in normal skin of HVs with multi-dosing, albeit with longer time to steady state (at least 14 d) compared to degradation in blood (7 d). This corresponded with increasing concentrations of drug in normal skin through 14 d of dosing, resulting in exposures in skin that were more than tenfold higher than what was achieved in plasma at steady state. Although basal levels of IRAK4 were approximately twofold higher in skin lesions in patients with HS and patients with AD compared to HV skin, the overall pattern of KT-474 exposure and IRAK4 knockdown in skin lesions was similar to HV skin. This demonstrated that the KT-474 PK–PD relationship in skin is distinct from that in blood but is similar between HVs and patients.

IRAK4 knockdown in blood and skin was generally well tolerated in HVs and patients. There were no drug-related infections during the 14–28 d of dosing when IRAK4 was maximally suppressed or during the subsequent 2 weeks of follow-up when IRAK4 levels were returning toward baseline. This was consistent with the phenotype of IRAK4 null humans, who, in infancy and early childhood, have a susceptibility to select bacterial infections that resolve once they reach adulthood^33,34^. The absence of drug-related infections in patients with HS, who have inflammatory abscesses and draining tunnels and may, therefore, be vulnerable to infections if immunosuppressed, was also encouraging. Headache AEs, although infrequently requiring treatment and not interfering with dosing, were observed in HVs more commonly in those getting KT-474 versus placebo and in KT-474–treated patients. Headaches have also been reported with the selective IRAK4 kinase inhibitor PF-06650833 (ref. ^24^). However, headaches are not a clinical manifestation in IRAK4 null individuals^33,34^, and the highly selective nature of IRAK4 degradation with KT-474 makes off-target effects as the cause of headaches unlikely. Gastrointestinal AEs were infrequent, as were heart palpitations. For those patients whose palpitations occurred while under observation in the phase 1 unit or clinic, there were no objective findings of arrhythmia on vital signs and/or electrocardiogram. The modest, subclinical QTcF prolongation seen only with multi-dosing was atypical due to its delayed onset and spontaneous resolution with continued dosing in patients beyond 14 d. This atypical pattern, along with human genetics^33,34^ and preclinical data with KT-474, suggest that the observed QTcF prolongation was unlikely to be related to IRAK4 targeting.

We have demonstrated that IRAK4 degradation functionally inhibits the TLR/IL-1R pathway in patients treated with KT-474. In HVs, we used ex vivo stimulation of whole blood with TLR4 and TLR7/8 agonists and showed broad inhibition of disease-relevant proinflammatory cytokine and chemokine production associated with doses of KT-474 achieving more than 90% IRAK4 knockdown in PBMCs. The HS and AD patient cohort demonstrated that IRAK4 degradation by KT-474 in blood and skin had a systemic anti-inflammatory effect. Although HS and AD are inflammatory diseases of the skin, plasma levels of proinflammatory cytokines and acute phase reactants involved in innate immunity, as well as Th1 and Th2 responses, have been shown to be elevated in these diseases^35–37^. We found that IL-6 and IL-1β were elevated in patients with HS and patients with AD, whereas CRP was increased only in patients with HS. IRAK4 degradation by KT-474 led to decreases of these inflammatory biomarkers in HS and AD, demonstrating in patients that IRAK4 targeting can impact inflammation in both diseases. RNA-seq analysis of skin lesions showed upregulation of multiple proinflammatory pathways in HS and AD relative to HV skin, with substantial overlap between the two diseases. This was consistent with previous publications reporting on the transcriptomic profile of AD and HS skin lesions^9,38^. The substantially stronger upregulation of proinflammatory gene expression that we observed with moderate to severe HS compared to AD, including genes associated with innate immunity, Th1 inflammation and humoral immune responses, is consistent with the generally greater inflammatory burden in HS associated with tissue destruction and pain and facilitated the demonstration that KT-474 treatment for 28 d could downregulate multiple different proinflammatory pathways. Specifically, the activity against IL-1β, IFN-γ, IL-8, NLRP3, TLR2 and CXCL13, GZMB and MMP12 showed that KT-474–mediated IRAK4 degradation could impact disease-relevant genes in HS that drive the activation and migration of neutrophils, macrophages, natural killer cells, T cells and B cells and lead to inflammation and tissue destruction. In AD, it was more challenging to show a significant effect of KT-474 on inflammatory biomarkers in the skin, which may have had more to do with the lower basal levels of expression in the enrolled patients than with the relative roles of IRAK4 in driving inflammation in HS versus AD. As already noted, KT-474 did reduce circulating inflammatory biomarkers in AD as well as in HS and impacted clinical endpoints in both diseases, pointing to a role for IRAK4 in the pathogenesis of inflammation in both diseases. In patients with AD, we did observe a trend in downregulation of IL-1α, COX2, OX40, CXCL13 and IL27RA, further supporting that the inflammation in AD is pleiotropic and that IRAK4 targeting may be capable of modifying the disease by impacting novel pathways.

As clinical trials with active biologics in HS (for example, anti-TNF-α and anti-IL-17 antibodies and JAK inhibitors) and AD (for example, cytokine-targeting antibodies and JAK inhibitors) have shown improvement in skin lesions and symptoms as early as 4 weeks into treatment^39–44^, we explored the clinical activity of KT-474 administered to patients for 4 weeks as part of the phase 1 study. In patients with predominantly moderate to severe disease, there was evidence of response in both HS and AD. The gradual improvement in skin lesions and symptoms over the 28 d of dosing is consistent with the kinetics of IRAK4 degradation that we observed in HVs and patients, where steady-state reduction occurs in blood by 7 d of dosing but in skin appears to require more than 14 d of dosing. It is, therefore, likely that maximum degradation in skin lesions, even if achieved over the 28 d of dosing, was only maintained for fewer than 2 weeks, making the observed clinical activity more remarkable. The fact that clinical responses were either maintained or continued to improve over the 2 weeks after the completion of dosing is consistent with the relatively slow kinetics of IRAK4 recovery demonstrated in both skin and blood when dosing was stopped.

In HS, the two patients with very severe disease, both of whom had progressed on prior biologics, did not show clinical response. It is possible that these patients with very severe, biologic-refractory disease either required a longer duration of KT-474 therapy to see response or had a greater disease burden that was less responsive to IRAK4 targeting. In AD, KT-474, on average, had a modest effect on EASI score at 4 weeks. However, there was a robust reduction of pruritus, suggesting that improvement in skin lesions may lag behind the effect on pruritus, and, therefore, a greater impact on EASI score may be achieved with longer duration of KT-474 dosing. The marked improvement in pain and/or pruritus that we observed in patients with HS and patients with AD treated with KT-474 for a relatively short period of time also raises the possibility that IRAK4 degradation could be modifying those symptoms via a neuro-immune mechanism in conjunction with its impact on skin lesion inflammation. The activation of TLRs and IL-1Rs on sensory nerves in skin and dorsal root ganglia has been implicated in both neuropathic pain and pruritus^45–49^, and IRAK4 targeting has been shown to reduce pain in animal models^50^.

Clinical proof of concept for heterobifunctional degraders has been limited to a small number of oncology programs where compounds targeting estrogen receptor (ARV-471) (refs. ^51,52^), androgen receptor (ARV-110) (ref. ^53^) or Bruton’s tyrosine kinase (NX-2127) (ref. ^54^) have demonstrated, in meeting presentations, varying degrees of target knockdown as well as anti-tumor activity with acceptable safety in phase 1 and phase 2 trials in breast cancer, prostate cancer and lymphoma, respectively. This phase 1 trial of KT-474 is important, in our view, in that it provides the first comprehensive assessment of heterobifunctional degrader safety, PK and PD in HVs and is the first such degrader trial in a non-oncology disease indication to extend those findings to patients and also demonstrate preliminary clinical proof of concept in HS and AD. Although the preliminary clinical activity of KT-474 in HS and AD in this study needs to be confirmed in future trials, it is notable that there was a consistent impact on objective, as well as subjective, clinical measures in both diseases with response kinetics that matched the PD effect on IRAK4 levels. This occurred in conjunction with a systemic anti-inflammatory effect, which included downregulation of disease-relevant gene transcripts in skin lesions.

The limitations of the patient cohort portion of the study include the small sample size, the absence of a placebo control group and the relatively short duration of therapy. The small sample size and lack of placebo control could have impacted the assessment of clinical efficacy for diseases such as HS and AD where the clinical course can wax and wane over time in some patients. Dosing with KT-474 for 28 d likely provided only a truncated view of the drug’s clinical activity, because it takes approximately 1 week to reach steady-state IRAK4 degradation in blood and at least 2 weeks to reach steady-state degradation in skin. The safety and efficacy of continuous long-term dosing with KT-474, which is anticipated for patients with HS and AD, was also not addressable in this trial.

Our study shows that an oral IRAK4 degrader in development for TLR/IL-1R–driven autoimmune diseases has robust in vivo activity against the target and pathway in HVs and patients, with a favorable safety and tolerability profile. The preliminary clinical activity in patients with moderate to severe HS and in patients with moderate to severe AD is encouraging and supports moving forward with confirmatory, placebo-controlled phase 2 trials.

## Methods

### Preclinical in vitro characterization of KT-474

#### Compounds

KT-474 was discovered through rational design and structure activity relationships exploration. PF-06550833 was purchased from Sigma-Aldrich (PZ0327).

#### Cell treatment for proteomics selectivity experiments

For selectivity analysis, isolated human PBMCs (AllCells) were treated with KT-474 at 300 nM in RPMI 1640 media supplemented with 10% FBS and penicillin–streptomycin for 24 h. Vehicle (DMSO)-treated cells at a final concentration of 0.05% (v/v) were used as controls.

#### Proteomics and data analysis

PBMCs were lysed using the iST sample preparation kit (PreOmics). After tryptic digestion, peptides were desalted and labeled using TMTpro reagents (Thermo Fisher Scientific). Pooled samples were fractioned offline using basic reversed-phase chromatography and recombined using a non-continuous pooling scheme as previously described^55^. Each peptide fraction was separated on an Easy nLC1200 nano HPLC over a 150-min gradient and analyzed online with an Orbitrap Eclipse (Thermo Fisher Scientific) mass spectrometer in data-dependent mode with MS2-based reporter quantification. Raw data were processed with MaxQuant^56^ (version 1.6.14.0) and searched with the Andromeda^57^ search engine against a comprehensive SwissProt database release for human. Peptide spectral matches were filtered for a precursor intensity fraction of >0.75 to be considered for quantification. Protein identifications were collapsed at the gene level, and at least two quantified razor or unique peptides were required for proteins to be reported. A paired statistical analysis was performed using the limma R package^58^. Significant degradation was determined by application of a weighted cutoff incorporating both negative logarithmic *P* value and log_2_ fold change using equation (1).

1 $$f(x)=-\,\log 10(0.05)+\frac{1}{|{x}^{2}-0.5|}$$

#### IRAK4 detection by flow methods in PBMCs

IRAK4 degradation was evaluated in PBMCs using flow methods. Frozen PBMCs were thawed into RPMI with 10% FBS, and 90 μl of the solution was plated per well. KT-474 was prepared at a 10 μM starting dose, followed by fivefold dilution and a 10-point dose curve and added at a final DMSO concentration of 0.1%. Cells and compound were incubated at 37 °C and 5% CO_2_ overnight (20 h). After incubation, cells were fixed with Cytofix Fixation Buffer from BD Biosciences and washed two times with PBS/2% FBS, and pellets were stored at −80 °C until further processing. On flow run day, cell pellets were thawed, and pre-permeabilization staining cocktail (CD3/CD14/CD56/CD19) was added. Samples were subsequently permeabilized with 60% methanol for 10 min at 4 °C, followed by incubation with post-permeabilization staining cocktail (CD16/IRAK4). Stained samples were run on the Attune NxT flow cytometer. Data were analyzed using FlowJo, and GraphPad Prism was used to generate 50% inhibitory concentrations (IC_50_s) using a four-parameter logistic regression curve, free-fit.

Percent IRAK4 signal was identified in B cells (CD3^−^/CD19^+^), monocytes (CD3^−^/CD19^−^CD14^+^) and lymphocytes (identified by side scatter size and CD14^−^).

#### Human cell cytokine release assay

Frozen PBMCs were thawed into RPMI with heat-inactivated 10% FBS/1% penicillin–streptomycin and the same day plated into 96-well flat-bottom plates at 200,000 cells per well in 190 μl of media. KT-474, PF-06550833 and DMSO controls were prepared in duplicate for each donor. All cells were dosed using the Tecan automated liquid handler, followed by incubation at 37 °C and 5% CO_2_ for 16 h, with final testing concentrations of 0.0064, 0.032, 0.16, 0.8, 4, 20, 100 and 500 nM. After 16 h of pre-treatment with the compounds, LPS (O55:B5) (Sigma-Aldrich, L2637) or R848 (Invivogen, tlrl-r848) were added at 100 ng ml^−1^ or 10 μg ml^−1^ final concentration, respectively. Cells were incubated an additional 5 h at 37 °C and 5% CO_2_. After assay completion, plates were centrifuged at 300*g* for 5 min. A total of 150 μl of supernatant was carefully removed and placed into a new 96-well V-bottom plate and stored at −80 °C until further analysis. Meso Scale Discovery (MSD) human U-plex or V-plex assays were used to measure cytokine levels. On the day of cytokine analysis, supernatant samples were thawed, diluted with MSD assay diluent and added to MSD plates. The assay was further completed per standard manufacturer’s protocol. Cytokine data were normalized to stimulated and unstimulated controls. The concentration of cytokines in supernatant was determined using MSD Discovery Workbench software. GraphPad Prism was used to generate IC_50_s using a four-parameter logistic regression curve, free-fit.

#### Human B cell phospho-flow assays

CD19^+^ B cells were purchased from BioIVT or isolated in-house using a negative selection kit (STEMCELL Technologies, 17954). Frozen B cells from *n* = 5 donors were thawed and plated at 450,000 cells per well in 190 μl of media in 96-well U-bottom plates. KT-474, PF-06550833 and DMSO controls were prepared in duplicate for each donor. All cells were dosed using the Tecan automated liquid handler, followed by incubation at 37 °C and 5% CO_2_ for 16 h, with final testing concentrations of 0.12, 0.489, 1.95, 7.81, 31.2, 125, 500 and 2,000 nM. After 16 h of compound pre-treatment, CpG-B (InvivoGen, tlrl-2006) was added at 2.5 μM final concentration for 60 min. After incubation, an equal volume of BD Cytofix Fixation Buffer was added to wells. Cells were washed with PBS + 2% FBS before being permeabilized on ice for 30 min using cold BD Perm Buffer III (BD Biosciences, 558050). Cells were washed again before being stained with fluorescently tagged antibody (phycoerythrin (PE) phospho-p65, clone K10-895.12.50 (BD Biosciences, 558423)) for 30 min at room temperature in the dark. Cells were then washed twice before being acquired using the Attune NxT flow cytometer, 10,000 events per well. Data were analyzed using FlowJo, and GraphPad Prism was used to generate IC_50_s using a four-parameter logistic regression curve, free-fit.

### Phase 1 trial design

The phase 1 trial of KT-474 consisted of randomized, placebo-controlled SAD and MAD studies in HVs, followed by an open-label study in patients with moderate to severe HS and patients with moderate to severe AD (ClinicalTrials.gov identifier: NCT04772885). The protocol is available as Supplementary Information. The trial was conducted according to the International Council on Harmonization Good Clinical Practice guidelines and the principles of the Declaration of Helsinki. The Advarra institutional review board (IRB) in Columbia, Maryland, reviewed and approved this study. All patients provided written informed consent before screening and enrollment.

Key eligibility criteria for HVs included males or females aged 18–55 years without clinically relevant medical histories or presence of clinically relevant medical disorders. Individuals who had used any prescribed medications other than hormonal contraceptives within 30 d or five half-lives (whichever was longer) of KT-474 administration were excluded. Randomization codes for associated treatment were prepared at the contract research organization using a validated computer program for both SAD and MAD portions of the study; a designated site staff member was able to unblind a study participant or cohort through individually sealed documents in case of emergency and was to be reported immediately by the investigator to the unblinded statistician, the Safety Review Committee (SRC) and other designated entities. Within each SAD cohort, eight patients were randomized 6:2 in a double-blind manner to receive a single dose of KT-474 or placebo after having fasted 10 h before and 4 h after the dose. Patients were confined on a phase 1 unit (in Hollywood, Florida, at Research Centers of America or in Fair Lawn, New Jersey, at TKL Research, Inc.) through day 5 and then followed on an outpatient basis through day 14. At each dose level, two sentinel patients were randomized to receive KT-474 or placebo. The remaining patients were dosed after the 24-h post-dose safety data from the sentinel patients had been reviewed by the investigator. Subsequent dose cohorts were initiated after the SRC had determined that all requirements for dose escalation were met based on a blinded review of the safety data through day 14 and PK data through day 5. The following KT-474 doses were assessed in SAD: 25, 75, 150, 300, 600, 1,000 and 1,600 mg. Within each MAD cohort, 12 patients were randomized 9:3 in a double-blind manner to receive KT-474 or placebo once daily (QD) from day 1 to day 14 after having fasted 10 h before and 4 h after the dose. The patients were confined through day 21 and then followed on an outpatient basis through day 28. Subsequent dose cohorts were initiated after the SRC had determined that all requirements for dose escalation were met based on a blinded review of the safety data through day 28, PK data through day 15 and PD data through day 7. The following KT-474 doses (mg daily × 14 d) were assessed in MAD: 25, 50, 100 and 200.

Key eligibility criteria for patients with AD or patients with HS included males or females aged 18–75 years with body mass index of 17.5–40.0 kg m^−^^2^ who weighed >45 kg and who were generally in good health. Patients with AD must have had involvement of ≥10% treatable body surface area excluding the scalp and designated venous access areas at screening or on admission, and patients with HS must have had a Total AN count ≥4 at baseline with fistula and tunnel count <20. Patients who had received prescription or non-prescription drugs for the treatment of AD or HS (including corticosteroids more potent than hydrocortisone 1% and vitamin and dietary supplements) within five half-lives or within 28 d (whichever is shorter) before the first dose of KT-474 were excluded. Patients were treated on an outpatient basis (at Encore Medical Research, LLC (Hollywood and Weston, Florida), Medical Dermatology Specialists (Phoenix, Arizona), Research Centers of America (Hollywood, Florida) and TKL Research, Inc. (Fair Lawn, New Jersey)) with KT-474 daily for 28 d at a dose of 75 mg per day in the fed state and followed through day 42. Dosing in the fed state was preferred over dosing in the fasted state to facilitate compliance with dosing at home. A modest increase in KT-474 plasma exposure was observed with food in additional SAD cohorts exploring food effect. As a result, a 75-mg dose in the fed state was chosen to simulate the plasma exposure observed in HVs treated with 100 mg QD in the fasted state (MAD cohort 3), which resulted in robust IRAK4 degradation in PBMCs and skin.

The primary objective of the trial was to determine the safety and tolerability of KT-474 as single and multiple daily doses in HVs and patients with HS and patients with AD, with endpoints including treatment-emergent AEs, treatment-emergent SAEs, concomitant medications, clinical laboratory tests (including hematology, coagulation, chemistry, urinalysis and urine microscopy), vital signs and safety electrocardiogram and Holter monitoring. The secondary objective was to characterize the PK profile of KT-474 in plasma and urine in HVs and patients. The main exploratory objectives were to characterize the PD profile and skin PK of KT-474 in HVs and patients and explore clinical efficacy in HS and AD. The PD endpoints included changes in IRAK4 levels and inflammatory biomarkers in blood and skin as well as changes in ex vivo whole blood cytokine induction. Efficacy endpoints in patients with HS included change from baseline in Total AN count, Pain NRS, Peak Pruritus NRS and HS-PGA. Additional endpoints derived from these included AN 0/1/2 (Total AN count of 0, 1 or 2), HiSCR50 (50% or greater reduction in AN count with no increase in abscesses or fistulas) and Pain NRS30. In patients with AD, efficacy endpoints included change from baseline in EASI, Peak Pruritus NRS and Investigator Global Assessment, with Peak Pruritus response derived from Peak Pruritus NRS (≥4 unit reduction from baseline).

### Plasma, urine and skin PK

Serial venous blood sampling (6 ml) and 24-h urine collections for quantification of KT-474 were performed on intensive PK sampling days. Skin biopsy samples were collected in MAD and open-label portions of the study and used for the quantification of KT-474 concentrations. Concentrations of KT-474 in plasma and urine were measured using validated liquid chromatography with tandem mass spectrometry (LC–MS/MS) assays with a lower limit of quantification (LLOQ) of 0.150 ng ml^−1^ and a quantitation range of 0.150–200 ng ml^−1^. KT-474 concentrations were determined in frozen skin biopsy samples using an LC–MS/MS method with an LLOQ of 1 ng ml^−1^ and a quantitation range of 1–1,000 ng ml^−1^.

PK parameters for KT-474 were estimated from plasma and urine concentration data via non-compartmental analysis using Phoenix WinNonlin (version 8.3.2, Certara).

### IRAK4 degradation in blood and skin by MS

Targeted mass spectrometry (LC–PRM/MS) was performed at Biognosys on isolated PBMCs collected from blood samples (6 ml of K2EDTA) or frozen skin biopsies. PBMCs were isolated at the clinical site using the Ficoll-paque filled Leucosep system, and, once purified, the PBMC pellets were frozen and shipped to Biognosys where the PBMC pellet was lysed and denatured using Biognosys Denature Buffer. The 5-mm skin biopsies were collected, trisected from the dermis to the epidermis and immediately frozen. One of the frozen aliquots was shipped to Biognosys where the tissue was homogenized with a Precellys homogenizer, and proteins were denatured using Biognosys Denature Buffer. Protein concentrations for both PBMCs and skin were determined by bicinchoninic acid (BCA) assay and then reduced and alkylated for 60 min at 37 °C, followed by overnight incubation with trypsin. C18 cleanup for MS was carried out using BioPureSPE MINI spin columns. Peptides were evaporated to complete dryness, and peptide concentrations were measured using the micro BCA assay. Before LC–PRM/MS analysis, sample preparations were spiked with the SIS peptide pool at a concentration of 30 fmol µg^−1^ tryptic peptides. The peptide pool contained three SIS peptides for IRAK4 and one SIS peptide for PARK7 as a control as well as additional control proteins PARK7, GAPDH and ACTB without SIS peptides. Values were reported as relative intensities between samples. For the LC–PRM/MS measurements, 1 µg of tryptic peptides per sample was injected in the reversed-phase column (PicoFrit emitter with 75-µm inner diameter, 60-cm length and 10-µm tip from New Objective, packed with 1.7-µm Charged Surface Hybrid C18 particles from Waters) on a Thermo Fisher Scientific EASY-nLC 1200 nano-liquid chromatography system connected to a Thermo Fisher Scientific Q Exactive HF mass spectrometer equipped with a Nanospray Flex Ion Source. A standard data-independent acquisition mode run was acquired for retention time-based scheduling using the high-precision normalized iRT concept. For analytical data acquisition, the scheduling window was 8 min per peptide. The normalized collision energy was 27 for all *m*/*z* values.

Signal processing and data analysis were carried out using SpectroDive software. Peak integration, retention time alignment and peak scoring were passed on mProphet^59^, and automated peak integration was verified by trained personnel for all experiments. A *q* value filter of 0.05 was applied. Absolute quantities of target peptides were determined using multi-point calibration. Calibration curves were carried out in tryptic peptide preparation of human PBMC and frozen skin background matrices with SIS peptides diluted in triplicate across a range of seven concentrations. The calibration curve was centered around the predicted endogenous concentration of each target peptide (C5), and two fourfold concentration steps above as well as four twofold concentration steps below were covered. Peptide calibration curves for each target peptide response determined empirical values for slope, linearity range, coefficient of correlation (R^2^) and LLOQ. For sample analysis, samples that yielded insufficient material for measurements were marked as fail, and, for individual values with no detectable peak, ‘ND’ was reported. All data post-processing was carried out in the R statistical environment, version 4.0.5. Of the three IRAK4 peptides, the IRAK4 peptide SANILLDEAFTAK was reported because this peptide had the greatest linear range and LLOQ at 0.025 fmol μg^−1^ protein in PBMCs and 0.012 fmol μg^−1^ protein in skin. IRAK4 percent change from baseline was calculated by subtracting the post-dose fmol μg^−1^ protein from the pre-dose values and then dividing by the pre-dose value. In study participants where the pre-dose value for fmol μg^−1^ peptide was below the LLOQ for IRAK4, and housekeeping genes were also noticeably low as compared to the participants’ other timepoints, the participant was excluded from assessment. In the situation where the post-dose was below limit of quantitation and housekeeping values were in line with other timepoints, the IRAK4 value reported was half the LLOQ value, such that, in PBMCs, that value was 0.0125 fmol μg^−1^ protein, and, in skin, it was 0.006 fmol μg^−1^ protein.

### IRAK4 degradation in blood by FLOW

FLOW cytometry was performed at CellCarta. Whole blood samples were collected in Na-Hep tubes at the clinical site and shipped on the day of collection at 4 °C. Upon receipt, samples were lysed/fixed and stored at −80 °C until processing. Lysed/fixed samples were divided into plates for unblocked and blocked conditions, stained with a pre-permeabilization antibody cocktail (CD14 1:25, CD56 1:50, CD19 1:100, CD3 1:200, CD4 1:200, CD8 1:200, CD45 1:200, CD15 1:200) for 30 min at room temperature, washed and permeabilized with 60% methanol at 4 °C for 10 min, followed by incubation with IRAK4 unconjugated antibody at 12.5ug ml^−1^ (blocking condition) or BSA (unblocked) for 30 min at room temperature. Finally, samples were stained with post-permeabilization antibody cocktail (CD16 1:200, IRAK4 1:20) for 30 min at room temperature, washed and processed on a BD LSRFortessa flow cytometer. Cytometer settings and compensations were performed as per CellCarta’s internal procedures. FLOW cytometry data were acquired using BD FACSDiva software and stored in the format of flow cytometry standard (*.fcs) files on a secured read-only ‘Raw Data’ server. Data were analyzed using the CellEngine templates established during development of the study. The assay lower limit was determined using a parallel blocking unconjugated antibody for each timepoint collected. To get the adjusted mean fluorescence intensity (MFI), the blocked MFI was subtracted from the unblocked MFI. Then, the adjusted MFI baseline was subtracted from the timepoint-adjusted MFI, which was then divided by the adjusted baseline MFI to get the IRAK4 percent change from baseline for each patient at each timepoint.

### Ex vivo cytokine induction in whole blood

Ex vivo cytokine induction in blood was evaluated using the Rules-Based Medicine (RBM) TruCulture system containing either of the TLR stimulants R848 or LPS. An unstimulated null tube was also collected at each timepoint to evaluate the effect of stimulation in each patient for each analyte. One milliliter of whole blood was collected directly into each of the three TruCulture tubes. Once blood was collected, TruCulture tubes were inverted 3–5 times and immediately placed on a 37 °C heat block and incubated overnight and up to 24 h. Cells were manually separated from the supernatant using the provided filter system, and the entire sample was then frozen at −20 °C and batch shipped to RBM for analysis. Samples were run on the OptiMAP 13 analyte panel to measure changes in cytokine levels. The percent change from baseline for each analyte was calculated by subtracting the post-dose from the pre-dose and then dividing by the pre-dose. Our internal criteria for response of cytokines for each analyte was ≥2× the value of the unstimulated (null) baseline value. The TruCulture system with OptiMAP detection is a commercially available and clinically validated system that was developed by RBM.

### Plasma inflammatory biomarkers

A total of 2 ml of whole blood was collected in a K2EDTA vial. Samples were inverted several times and centrifuged at 1,300*g* for 10 min, and then the plasma was collected and subsequently stored at −80 °C until shipment to RBM. Plasma samples were profiled over an immunoassay panel that included high-sensitivity CRP in addition to high-sensitivity single molecular immunoassays for IL-1β and IL-6. These assays are commercially available and were validated at RBM. The percent change was calculated by subtracting the protein concentration from pre-dose values and then dividing by the pre-dose.

### Skin biopsy RNA-seq sample processing and analysis

Skin biopsies were collected, submerged in RNALater within 30 min of collection and incubated overnight at 4 °C. Supernatant was then removed, and the sample was frozen at −20 °C. RNA was extracted from the frozen biopsies using the Qiagen RNeasy Plus Universal Mini Kit (cat. no. 73404). Ribosomal RNA depletion was performed using the Qiagen FastSelect HMR (cat. no. 334375). RNA-seq libraries were prepared using the NEBNext Ultra II RNA library prep kit (cat. no. E7770L). Libraries were sequenced on an Illumina NextSeq 500 sequencer.

Raw sequencing data were processed by the standard Illumina software package bcl2fastq to demultiplex the reads and perform base calling.

Sequencing reads were trimmed using cutadapt^60^ using the following command line parameters: cutadapt -a AGATCGGAAGAGCACACGTCTGAACTCCAGTCA -A AGATCGGAAGAGCGTCGTGTAGGGAAAGAGTGT -m 25 -j 4 -o <trimmed_FASTQ_read_1 > -p <trimmed_FASTQ_read_2 > <input_FASTQ_read_2 > <input_FASTQ_read_1>

After trimming, genes were quantified by pseudoalignment with kallisto^61^ using the Ensembl hg38 human genome reference and the command line parameters shown below: kallisto quant -i {input.index} -t 8 -b 100–bias -o <kallisto_output_directory > <trimmed_FASTQ_read_1 > <trimmed_FASTQ_read_2 > '

Differential expression analysis was performed using sleuth^62^. The estimated log fold changes (‘b’ values) were used to run GSEA^63,64^ in pre-ranked mode using the GSEA command line software package downloaded from https://www.gsea-msigdb.org/gsea/index.jsp. Normalized enrichment scores and false discovery rate (FDR) *q* values were extracted from the GSEA text output.

Analytical pipelines were implemented using snakemake^65^. R 4.2.0 (https://www.r-project.org) and the R tidyverse 2.0.0 libraries (https://www.tidyverse.org) were used to generate figures.

### Statistical analysis

For statistical analyses that do not specify the software used, they were conducted using SAS version 9.4. Sample size was based on practical considerations, as efficacy analyses were considered exploratory for this study. Data collected in this study were documented using summary tables and subject data listings. Continuous variables were summarized using descriptive statistics (number of subjects, mean, median, standard deviation, minimum and maximum). Categorical variables were summarized using frequencies and percentages. Data from different study parts (A, B and C) are presented in separate tables. For each study part, data are presented by dose cohort. Placebo patients from different cohorts in the same study part were combined in one column. For statistics of maximum values (Extended Data Fig. 3 and Extended Data Table 4), the individual maximum percentage change/reduction over the specified timeframe was identified for each patient, and descriptive statistics were determined for the maximum change/reduction for each dose group.

Formal statistical testing was not pre-specified. Post hoc testing was conducted for selected analyses, and nominal *P* values were presented to show strength of data. The *P* values for the comparisons between the treatment groups and placebo in IRAK4 degradation and cytokine production (Fig. 1b,d,f and Extended Data Fig. 4) were based on one-way ANOVA models with Tukey correction for multiple comparisons. The *P* values in Fig. 2b, for the skin IRAK4 levels of patients with AD and patients with HS on day 28 compared to baseline, were based on one-way ANOVA models.

Safety summaries were based on all study participants receiving at least one dose of KT-474 or placebo and included incidence and type of AEs, plus absolute values and changes in blood pressure, heart rate, temperature, clinical laboratory data, physical examination, neurological examination data and 12-lead electrocardiogram data from pre-dose to post-dose timepoints.

### Reporting summary

Further information on research design is available in the Nature Portfolio Reporting Summary linked to this article.

## Online content

Any methods, additional references, Nature Portfolio reporting summaries, source data, extended data, supplementary information, acknowledgements, peer review information; details of author contributions and competing interests; and statements of data and code availability are available at 10.1038/s41591-023-02635-7.

### Supplementary information

Supplementary Information Qualification report for FLOW assay, gating strategy and redacted protocol.

Reporting Summary

Supplementary Data 1 Source data for Tables 1 and 2.

### Source data

Source Data Fig. 1 Tables for number of patients screened, enrolled, treated and discontinued; Mean IRAK4 versus Time Data in PBMCs after single dosing; Individual IRAK4 Data in PBMCs at 48 h after single dosing; Mean IRAK4 Data in PBMCs at 48 h after single dosing; Mean IRAK4 versus. Time Data in PBMCs after multiple dosing; Individual IRAK4 Data in PBMCs at day 14 after multiple dosing; Mean IRAK4 Data in PBMCs at day 14 after multiple dosing; Mean IRAK4 versus Time Data in skin after multiple dosing; Individual IRAK4 Data in skin at day 14 after multiple dosing; Mean IRAK4 Data in skin at day 14 after multiple dosing.

Source Data Fig. 2 IRAK4 Reduction in PBMCs versus Plasma Conc data for Part C and Part B; Individual IRAK4 Data in skin in AD/HS patients and HVs; Mean IRAK4 Data in skin in AD/HS patients and HVs.

Source Data Fig. 3 Raw data are already provided in the NCBI Sequence Read Archive. Added processed data underlying the figures.

Source Data Fig. 4 Mean change and s.e.m. for each panel.

Source Data Extended Data Fig. 1 Discovery proteomics data of human PBMCs treated with KT-474 versus DMSO. log_2_ fold change, negative log_10_ *P* value and significance score are provided for each protein quantified; Mean percent IRAK4 values normalized to DMSO control; Mean percent cytokines normalized to stimulated control; Mean percent phospho-p65 normalized to stimulated control.

Source Data Extended Data Fig. 2 Mean ΔQTcF Data in MAD3 and AD/HS patients on days 7, 14, 21 and 28; Individual ΔQTcF Data in MAD3 and AD/HS patients on days 7, 14, 21 and 28.

Source Data Extended Data Fig. 3 Mean Plasma Concentration versus Time Data after single dosing in HVs; Mean Plasma Concentration versus Time Data after multiple dosing in HVs; Mean Skin Concentration versus Time Data after multiple dosing in HVs; Mean Plasma Concentration versus Time Data after multiple dosing in AD/HS patients; Mean Skin Concentration versus Time Data after multiple dosing in AD/HS patients.

Source Data Extended Data Fig. 4 Mean reduction in LPS-stimulated cytokines after single dosing; Individual reduction in LPS-stimulated cytokines after single dosing; Mean reduction in R848-stimulated cytokines after single dosing; Individual reduction in R848-stimulated cytokines after single dosing.

Source Data Extended Data Fig. 5 Mean change and 80% confidence interval; change over time.

Source Data Extended Data Table 1 Individual data points for counts in table.

Source Data Extended Data Table 2 Individual data points for counts in table.

Source Data Extended Data Table 3 Individual data points for counts in table.

Source Data Extended Data Table 4 Individual analyte readings, mean, standard deviations and *n*s for each group.

## Figures and Tables

**Extended Data Table 1:**
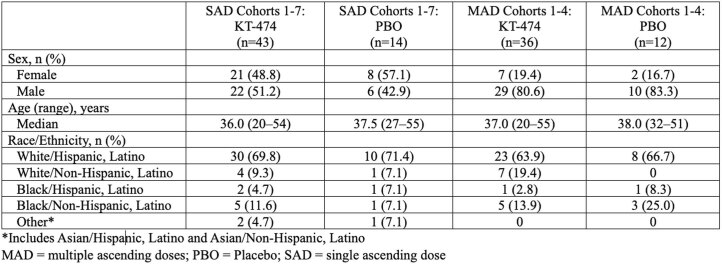
Baseline characteristics of HV SAD and MAD cohorts

*Includes Asian/Hispanic, Latino and Asian/Non-Hispanic, Latino. PBO, placebo.

Source data

**Extended Data Table 2:**
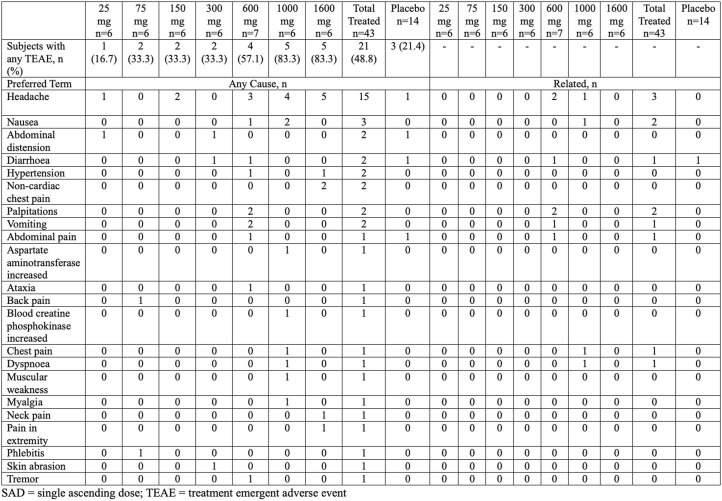
TEAEs by preferred term in SAD cohorts (individual and total) versus placebo

TEAE, treatment-emergent adverse event.

Source data

**Extended Data Table 3:**
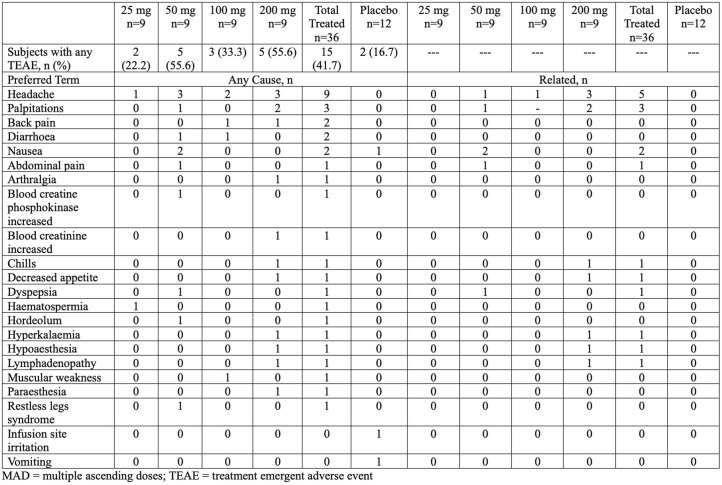
TEAEs by preferred term in HV MAD cohorts (individual and total) and in placebo

TEAE, treatment-emergent adverse event.

Source data

**Extended Data Table 4:**
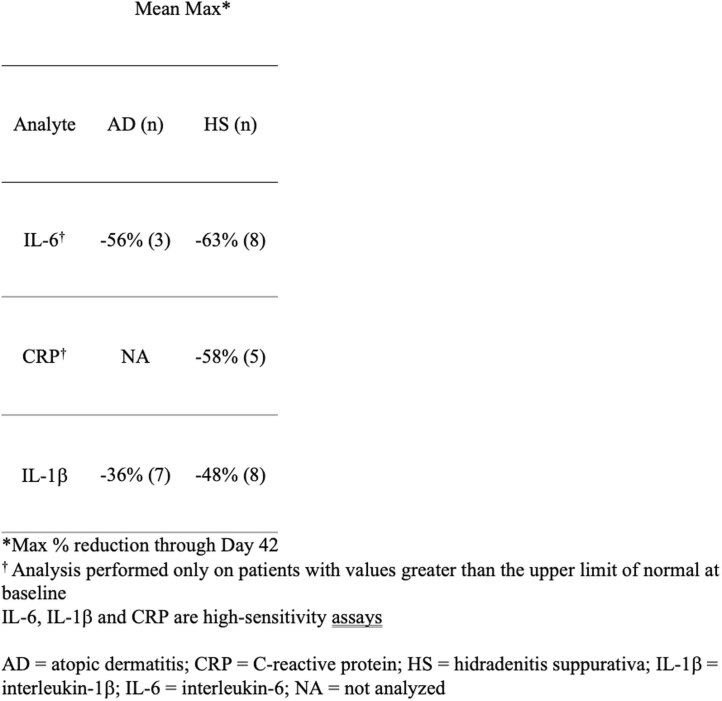
Effect of KT-474 on plasma cytokines and acute phase reactants in patients with HS and in patients with AD

*Maximum % reduction through day 42. ^†^ Analysis performed only on patients with values greater than the upper limit of normal at baseline. IL-6, IL-1β and CRP are high-sensitivity assays. NA, not analyzed.

Source data

## Data Availability

The clinical data that support the findings of this study are not openly available owing to reasons of sensitivity and are available from the corresponding author (jared@kymeratx.com) upon reasonable request. RNA-seq data are available at the National Center for Biotechnology Informationʼs Sequence Read Archive as BioProject number PRJNA1003129 (https://dataview.ncbi.nlm.nih.gov/object/PRJNA1003129?reviewer=o2jfesi1s6rtlgcsoofigfnft5). Requests will be responded to within 3 weeks of receipt. Source data are provided with this paper.

## Code availability

All code used for data collection and analysis is freely available and was not modified further. All sources of code are included in the Methods.
