# Supplementary material for: IRAK4 degrader in hidradenitis suppurativa and atopic dermatitis: a phase 1 trial
Source: Nat Med. 2023 Nov 13;29(12):3127–36. doi: 10.1038/s41591-023-02635-7 (PMC10719089; doi:10.1038/s41591-023-02635-7)
Supplement: Supplementary file 1 — Qualification report for FLOW assay, gating strategy and redacted protocol. [file 41591_2023_2635_MOESM1_ESM.pdf]

nature medicine

Article https://doi.org/10.1038/s41591-023-02635-7

IRAK4 degrader in hidradenitis suppurativa and atopic dermatitis: a phase 1 trial

In the format provided by the authors and unedited

DocuSign Envelope ID: 8ECCE282-5174-484C-8E06-7271AEF7B4F6

CAPRION

QUALIFICATION REPORT

Qualification of a Flow Cytometry Assay to Evaluate IRAK4 Assay Performance in Whole Blood

Testing Facility: **Caprion Biosciences, Inc.**
48383 Fremont Blvd, Suite 118
Fremont, CA
USA
94538

Hereinafter refered to as **"Caprion"**

Sponsor: **Kymera Therapeutics, Inc.**
400 Technology Square, 10th floor
Cambridge, MA 02139
USA
Hereinafter referred to as **"Sponsor"**

Caprion Biosciences Study Code: **6102**
Version: **FINAL**
Date: **17FEB2022**

ASSAY QUALIFICATION REPORT SIGNATURES

Caprion Biosciences

Name	Signature	Date
Juan Luis Callejas Valera, Ph.D.<br>Principal Scientist<br>Caprion Biosciences, Inc.	[signature]<br>Signature	17FEB2022<br>Date<br>(DDMMMYYYY)
Chenggang Jin, Ph.D.<br>Director Scientific Operations & Site Head<br>Caprion Biosciences, Inc.	[signature]<br>Signature	17FEB2022<br>Date<br>(DDMMMYYYY)

Kymera Therapeutics

Name	Signature	Date
Alice McDonald<br>Director, Translational Biology<br>Kymera Therapeutics	DocuSigned by:<br>Alice McDonald<br>1AC425378AA843E...<br>Signature	2/28/2022<br>Date<br>(DDMMMYYYY)
Veronica Campbell<br>Associate Director<br>Kymera Therapeutics	Veronica Campbell<br>Signature	25FEB2022<br>Date<br>(DDMMMYYYY)

CONTACT INFORMATION	
**Caprion Biosciences Contact Information**	
**Principal Scientist:**	
**Name:**<br>**Title:**<br>**Telephone:**<br>**Email:**	Juan Luis Callejas Valera, Ph.D.<br>Principal Scientist<br>+1-301-503-9760<br>jvalera@primitybio.com
**Management:**	
**Name:**<br>**Title:**<br>**Telephone:**<br>**Email:**	Chenggang Jin, Ph.D.<br>Director Scientific Operations & Site Head<br>408-220-5104<br>cjin@caprion.com
**Kymera Therapeutics. Contact Information**	
**Representative:**	
**Name:**<br>**Title:**<br>**Telephone:**<br>**Email:**	Alice McDonald<br>Director, Translational Biology<br>617-970-4879<br>alice@kymeratx.com
**Representative:**	
**Name:**<br>**Title:**<br>**Telephone:**<br>**Email:**	Veronica Campbell<br>Associate Director<br>978-376-4492<br>veronica@kymeratx.com

TABLE OF CONTENTS

Contact Information ... 3
Table of Contents ... 4
List of Abbreviations ... 5
1. Introduction and Objectives ... 6
2. Regulatory Compliance ... 6
2.1. GCP and GLP Compliance and Procedures ... 6
2.2. Ethics ... 6
2.3. Deviations ... 6
2.4. Amendments ... 6
3. Method Summary and Assay Readouts ... 7
3.1. Method Description ... 7
3.2. Reagents and Equipment ... 7
3.3. Samples Analyzed ... 9
4. Instrument and Software ... 9
5. Gating strategy ... 10
5.1. Populations assessed during analysis. ... 11
6. Qualifaction Parameters for Analysis ... 13
6.1. Assay Qualification Parameters ... 13
6.2. Recommended Assay Acceptance Criteria ... 14
7. Results and Discussion ... 14
7.1. Assay Precision ... 14
7.1.1. Intra-Operator Precision ... 14
7.1.2. Inter-Operator Precision ... 21
7.1.3. Inter-Instrument precision ... 27
7.1.4. Post-Draw Stability ... 31
7.1.5. Post-Freeze Stability ... 56
8. Summary ... 81
9. References ... 81
10. Appendix ... 81
10.1. Working instructions ... 81

LIST OF ABBREVIATIONS

**Abbreviations**	**Meaning**
BD	Becton Dickinson
BV	Brilliant Violet
BSA	Bovine Serum Abumin
CD	Cluster of Differentiation
CSP	Client Specific Procedure
CV	Coefficient of Variation
FACS	Fluorescence-Activated Cell Sorter
FBS	Fetal Bovine Serum
.fcs	Flow Cytometry Standard
FDA	Food and Drug Administration
FRQ	Frequency
GCP	Good Clinical Practice
GLP	Good Laboratory Practice
h	Hour
HD	Healthy Donor
ICH	International Council for Harmonization
IRB	Institutional Review Board
min	Minutes
MFI	Median Fluorescence Intensity
N/A	Not Applicable
QA	Quality Assurance
SOP	Standard Operating Procedure
WI	Working Instructions
µL	Microliter
°C	Degrees Celcius
CSV	Comma separated value
W%D	Weighted percent difference

1. INTRODUCTION AND OBJECTIVES

The objective of this study is to qualify an assay to evaluate the degradation of IRAK4 in whole blood in response to Kymera's degrader compound (KT-474). The qualified assay will be deployed later on whole blood subjects enrolled in a Phase 1 Clinical Trial.

2. REGULATORY COMPLIANCE

2.1. GCP AND GLP COMPLIANCE AND PROCEDURES

Applicable Standard Operating Procedures (SOP) in the form of Client Specific Procedure (CSP) (Table 1) were followed. The assay parameters tested in this assay transfer are in support of exploratory analysis of clinical samples.

Table 1. List of Applicable Working Instructions

WORKING INSTRUCTION NAME	WI NUMBER
Lyse/Fix Procedure (precision experiments)	D-CSP-02-6102-FIX-v1.0
Pre-Perm and Post-Perm Antibody Cocktail Preparation	CSP-01-6102-ABS-v1.0
Assay Form: Staining for IRAK4 Degradation Assay in Fixed/Frozen Human Whole Blood Samples (Precision Experiments)	CSP-02-6102-FRM-v1.0

2.2. ETHICS

For procurement of healthy donor (HD) specimens, blood collection from HD volunteers were conducted in accordance with the International Council for Harmonization (ICH) Guideline for Good Clinical Practice (GCP) E6(R1) following Institutional Review Board (IRB)-approved protocol for Blood Collection in Healthy Volunteers for Immunology and Proteomics Research (Protocol Number G522).

2.3. DEVIATIONS

No deviations were identified or investigations performed as part of this study.

2.4. AMENDMENTS

No amendments were submitted for the assay transfer plan.

3. METHOD SUMMARY AND ASSAY READOUTS

3.1. METHOD DESCRIPTION

100 µL of whole blood from HD Na-Hep tubes will be Lysed/Fixed for 10 min at room temperature in deep well plates followed by 2 washes with 2% FBS in PBS. After this step samples will be either directly stained or frozen at -80°C to evaluate samples stability.

Next, 45 µL of each Lysed/Fixed sample will be transfered to armadillo plate for each condition (unblock and blocking condition) and wash with 0.5% BSA in PBS. Then each well will be stained with Pre-permeabilization antibody cocktail for 30min at RT. Afterward, samples will be washed with 0.5% BSA in PBS twice and permeabilzed with 60% methanol for 10 min at 4°C.

After permeabilization, samples will be washed 3 times with 0.5% BSA in PBS and incubate with IRAK4 uncongugated antibody (blocking condition) or 0.5% BSA in PBS (unblocking condition) for 30min at RT. Finally, each well will be stainied with Post-perm antibody cocktail for 30min at RT following with 3 washes in 0.5% BSA in PBS and then samples will be resuspended in 50 µL of 0.5% BSA in PBS and storage at 4°C until analysis.

Key details are described in the following table:

Table 2. Method Summary

FEATURES	DESCRIPTION
**Matrix**	Human Whole Blood
**Blood Collection Tube**	Na-Hep Vacutainer
**Sample Volume Required per Test**	100 µL of Whole Blood
**Method Type**	Flow Cytometry
**Assay Control Condition**	WB treated with Hu IRAK4 unconjugated (blocking condition)
**Flow Cytometer Quality Control Sample**	Cytometer Setup and Tracking (CS&T) beads

3.2. REAGENTS AND EQUIPMENT

The antibodies used for the panel are presented in Table 3. Optimal antibody titers were previously established for all antibodies used for all assay transfer experiments described in this plan.

Table 3. List of Antibodies

ANTIBODY	MANUFACTURER	CLONE	CATALOG #*	RECOMMENDED CONCENTRATION / DILUTION	STAIN COCKTAIL
CD14 BUV395	BD	MφP9	563561	1/25	Pre-perm
CD16 PE	Biolegend	B73.1	360704	1/200	Post-perm
CD56 BV711	Biolegend	HCD56	318336	1/50	Pre-perm
CD19 BV786	BD	SJ25-C1	563325	1/100	Pre-perm
CD3 Pacific Blue	Biolegend	UCHT1	300417	1/200	Pre-perm
CD4 Ax700	Biolegend	RPA-T4	300526	1/200	Pre-perm
CD8 FITC	Biolegend	RPA-T8	301060	1/200	Pre-perm
CD45 BUV805	BD	HI30	564914	1/200	Pre-perm
IRAK4 Ax647	BD	L29-525	560315	1/20	Post-perm
CD15 PE-Cy7	Biolegend	W6D3	323030	1/200	Pre-perm

**Catalog number may vary depending on vial format*

Other reagents used in this study, such as buffers specific to the assay are shown in Table 4.

Table 4. List of Reagents

REAGENTS	SUPPLIER	SPECIFICATIONS*
Hu IRAK4 unconjugated (IRAK4 blocker)	BD	Catalog #624084
100% Methanol	Fisher Chemical	Catalog # A412-1
Lyse/Fix Buffer 5X	BD Biosciences	Catalog # 558049
Fetal Bovine Serum (FBS)	HyClone	Catalog # SH30071.03
BSA Stain Buffer	BD Biosciences	Catalog # 554657
KT-474 IRAK4 degrader	Provied by client (IRW-A-1384-019H)	

**Catalog number may vary depending on format*

3.3. SAMPLES ANALYZED

The following assay development study was performed using human WB collected in Na-Heparin tubes from healthy donors (HD) provided by Stanford blood donor center. Whole Blood samples were stored at 4°C until processing. The following donors were used for qualification experiments:

- Inter-Operator/Intra-Operator Precision
  - W070521010460 (Donor 1) drawn on 01FEB2021
  - W070521010463 (Donor 2) drawn on 01FEB2021
  - W070521000170 (Donor 3) drawn on 01FEB2021
- Inter-Instrument Precision
  - W070521000170 (Donor 3) drawn on 01FEB2021
- Post-Draw Stability
  - W0705215100223 (Donor 1) drawn on 08FEB2021
  - W0705215100224 (Donor 2) drawn on 08FEB2021
  - W0705215100225 (Donor 3) drawn on 08FEB2021
- Post-Freeze Stability
  - W070521010460 (Donor 1) drawn on 01FEB2021
  - W070521010463 (Donor 2)drawn on 01FEB2021
  - W070521000170 (Donor 3) drawn on 01FEB2021

4. INSTRUMENT AND SOFTWARE

The instrument and associated software that were used to perform the assay experiments are presented in Table 5.

Table 5: Equipment and Software

ITEMS	DESCRIPTION
**Primary Instrument**	LSR Fortessa
**Analytical System Software**	BD FACS Diva software version 8.0.1[A]
**Sample Analysis Software**	CellEngine

ITEMS	DESCRIPTION
**Backup Instrument**	LSR II flow cytometer (kappa)
**Analytical System Software**	BD FACS Diva software version 8.0.1[A]
**Sample Analysis Software**	CellEngine v1

[A]Version can be upgraded upon approval of a change control. Minor update to a version may be used with documented justification/rationale.

DocuSign Envelope ID: 8ECCE282-5174-484C-8E06-7271AEF7B4F6

5. GATING STRATEGY

For this panel the following gating strategy was followed (shown in Figure 1):

1. Review of sample collection over time (exclusion of fluidic instability);
2. Exclusion of doublets using FSC-A vs FSC-H, and SSC-A vs SSC-H;
3. WBC selection based on SSC-A and FSC-A;
4. Exclusion of Neutrophils using CD16 vs CD15 staining;
5. PBMC selection based on CD45 marker;
6. Exclusion of CD14+ cells
    a) Identification of NK cells (CD56+)
    b) Selection of Lymphocytes based on CD3 and CD16 markers
        i. Identification of T Cells (CD3+)
            - Selection of CD4+ T-cells based on CD4 vs CD8 staining
            - Selection of CD8+ T-cells based on CD4 vs CD8 staining
        ii. Selection of CD3 negative cells using CD3 marker
            - Identification of B-cells (CD19+)
    c) Exclusion of CD3+ cells
        i. Exclusion of CD56+ cells
        ii. Exclusion of CD19+ cells
            - Selection of Monocytes subsets based on CD16 vs CD14 markers

Furthermore, IRAK4 readouts for PBMC, Lymphocytes, Monocytes, Intermediate Monocytes, Classical Monocytes, Non-Classical Monocytes, NK cells, CD4+ T-Cells, CD8+ T-Cells, B-Cells were reported (shown in Figure 2)

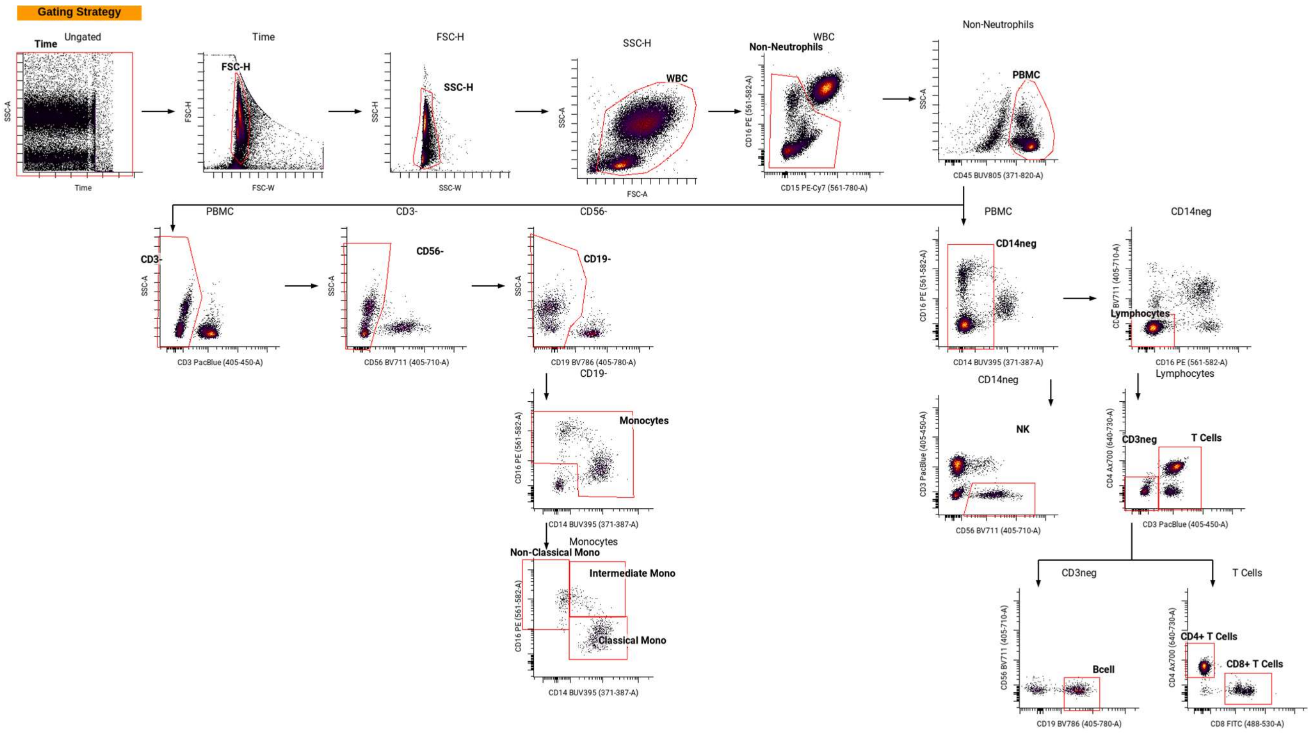

DocuSign Envelope ID: 8ECCE282-5174-484C-8E06-7271AEF7B4F6

Figure 1 . Major subset gating hierarchy for populations assessed in the Qualification Assays.

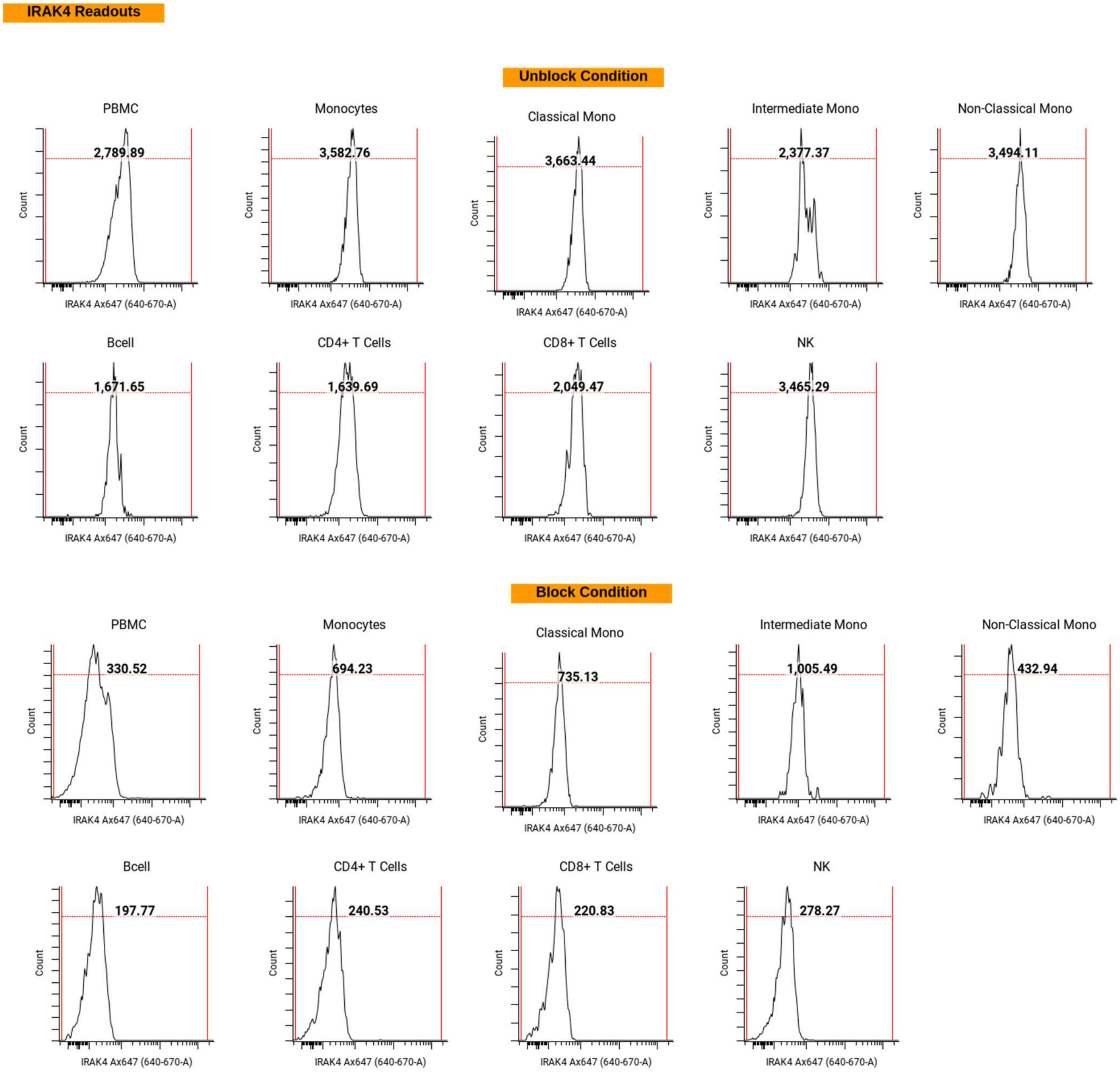

Figure 2. Readout markers assessed during qualification experiments for PBMC, Lymphocytes, Monocytes (including Classical, Intermediate and Non-Classical Monocytes), CD4+ T-Cells, CD8+ T-Cells, B-Cells and NK Cells for both conditions (block and unblock condition).

5.1. POPULATIONS ASSESSED DURING ANALYSIS.

The cell subsets and the readout markers evaluated for this study are shown in Table 6 and Table 7. From these populations, we evaluated each major cell subset/population as a function of percentage of PBMC except for PBMC subset that was evaluated as a function of WBC. For the readout populations, median fluorescence

intensity (MFI) was evaluated for the various levels of precision experiments. All the cell pupulations and readouts were assessed in presence/absence of IRAK4 blocker

Table 6. Major Cell Subsets Populations for Panel Assay.

Population	CD45	CD3	CD4	CD8	CD14	CD15	CD16	CD19	CD56	IRAK4	Metrics to be reported
PBMC	+	-	-	-	-	-	-	-	-	-	FRQ WBC
Lymphocytes	+	+	-	-	-	-	-	-	-	-	FRQ PBMC
B-Cells	+	-	-	-	-	-	-	+	-	-	FRQ PBMC
NK-Cells	+	-	-	-	-	-	+	-	+	-	FRQ PBMC
CD4 T-Cells	+	+	+	-	-	-	-	-	-	-	FRQ PBMC
CD8 T-Cells	+	+	-	+	-	-	-	-	-	-	FRQ PBMC
Monocytes	+	-	-	-	+/-	-	+/-	-	-	-	FRQ PBMC
Classical Monocytes (Exploratory_	+	-	-	-	+	-	-	-	-	-	FRQ PBMC
Intermediate Monocytes (Exploratory)	+	-	-	-	+	-	+	-	-	-	FRQ PBMC
Non Classical Monocytes (exploratory)	+	-	-	-	-	-	+	-	-	-	FRQ PBMC

Table 7. Readouts for Panel Assay.

Population	Subset	CD45	CD3	CD4	CD8	CD14	CD15	CD16	CD19	CD56	IRAK4	Metrics to be reported
IRAK4	PBMC	+	-	-	-	-	-	-	-	-	+	MFI
	Lymphocytes	+	+	-	-	-	-	-	-	-	+	MFI
	B Cells	+	-	-	-	-	-	-	+	-	+	MFI
	NK Cells	+	-	-	-	-	-	+	-	+	+	MFI
	CD4 T-Cells	+	+	+	-	-	-	-	-	-	+	MFI
	CD8 T-Cells	+	+	-	+	-	-	-	-	-	+	MFI
	Monocytes	+	-	-	-	+/-	-	+/-	-	-	+	MFI
	Classical Monocytes	+	-	-	-	+	-	-	-	-	+	MFI
	Intermediate Monocytes (exploratory)	+	-	-	-	+	-	+	-	-	+	MFI
	Non Classical Monocytes (exploratory)	+	-	-	-	-	-	+	-	-	+	MFI

6. QUALIFICATION PARAMETERS FOR ANALYSIS

6.1. ASSAY QUALIFICATION PARAMETERS

The following assay parmeters shown in Table 8 were evaluated for this study:

Table 8. Assay Qualification Parameters.

Qualification Parameter	Description	Number of Donors	Number of Replicates	Number of Runs or Days
Intra-Assay Precision	Precision across replicate samples processed and analyzed by a single operator in a single run	3	3	1
Inter-Operator Precision	Precision across replicate samples processed and analyzed by two different operators in a single run	3	3	1
Inter-Instrument Precision	Precision across replicate samples stained and acquired on two different instruments by the same operator	1	3	2
Post-Draw Stability	Stability of whole blood samples 1, 2, 3 and 4 days post draw at 4°C, processed, stimulated and stained by one operator	3	3	4
Post-Processing Stability	Longitudinal freeze-thaw stability of processed samples stored at -80°C were evaluated after Day 1, 9, 30 and 60	3	3	4

For Intra-Assay Precision experiments, the following calculation will be used to determine the %CV of experimental replicates for each donor:

$$\%CV = \frac{\text{Standard Deviation Triplicate}}{\text{Mean Triplicate}} \text{ x } 100$$

Next, Inter-Operator Precision will be calculated using the following equation by calculating the mean value of all replicates between operators as well as the standard deviation of replicates for each operator per sample:

$$\%CV = \frac{\text{Standard Deviation of Replicates (6)}}{\text{Mean of Replicates (6)}} \text{ x } 100$$

Inter-Instrument Precision will be determined by calculating the %CV of all replicates per sample between both instruments as similarly calculated for the inter-operator precision.

Lastly, for Post-Draw Stability and Post-Processing Stability, Weighted percent difference (W%D) will be calculated based on baseline (T0) and rest of the timepoints (Tn) with the following equation using Frequency for major subsets (% PBMC or WBC) or MFI for IRAK4 readouts.

$$W\%D = 100\ x\ (\frac{FRQ, MFI_{Tn} - FRQ, MFI_{T0}}{FRQ, MFI_{T0}})$$

6.2. RECOMMENDED ASSAY ACCEPTANCE CRITERIA

Table 9 shows the recommended acceptance criteria for the quantitative qualification parameters for each metric measured by the assay.

Table 9. Recommended Acceptance Criteria for Assay Qualification.

Qualification Parameter	Recommended Target Values
Intra-Assay Precision	%CV⩽25% across replicates for at least 2 of 3 donors for cellular subsets/readouts with abundance greater than 1% of total PBMC/WBC or greater than 250 MFI value
Inter-Operator Precision	%CV⩽25% across all samples analyzed by both operators for at least 2 of 3 donors for cellular subsets/readouts with abundance greater than 1% of total PBMC/WBC or greater than 250 MFI value
Inter-Instrument Precision	%CV⩽25% across all samples analyzed across two instruments by one operator for cellular subsets/readouts with abundance greater than 1% of total PBMC/WBC or greater than 250 MFI value
Post-Draw Stability	W%D of replicates for at least 2 of 3 donors each time point is ⩽ 25% for cellular subsets and readouts with percentage higer than 1% of total PBMC/WBC or greater than 250 MFI value
Post-Processing Stability	W%D of replicates for at least 2 of 3 donors each time point is ⩽ 25% for cellular subsets and readouts with percentage higer than 1% of total PBMC/WBC or greater than 250 MFI value

7. RESULTS AND DISCUSSION

7.1. ASSAY PRECISION

7.1.1. INTRA-OPERATOR PRECISION

For Intra-Assay precision, three (3) donor samples, in triplicate were stained by one operator for both block and unblock condition using IRAK4 blocker and the %CV for each donor was calculated for each cell subset as a percentage PBMC except for PBMC population where percentage was calculate as percentage of WBC. For readouts, key metric was median fluorescence intensity (MFI).

For major cell subsets assessed in the panel (Figure 3A-B and Table 10) Donor 2 and Donor 3 showed all the cellular subsets with a %CV below 25%. Donor1 displayed 17 of 20 cellular subsets with a %CV below. All cellular

subsets for Donor 3 and 18 cellular subsets for Donor 2 and Donor 1 demostrated an abundance of greater than 1% of total viable leukocytes.

Regarding readouts assessed in the Intra-Assay precision experiment, all readouts (20 in total) for Donor 1, Donor 2 and Donor 3 had a %CV below 25% (Figure 4A-B and Table 11).
Therefore, the results indicate that the assay has acceptable Intra-Assay Precision and notably, all populations minus one, greater than 1% of viable leukocytes showed %CV less than 25%.

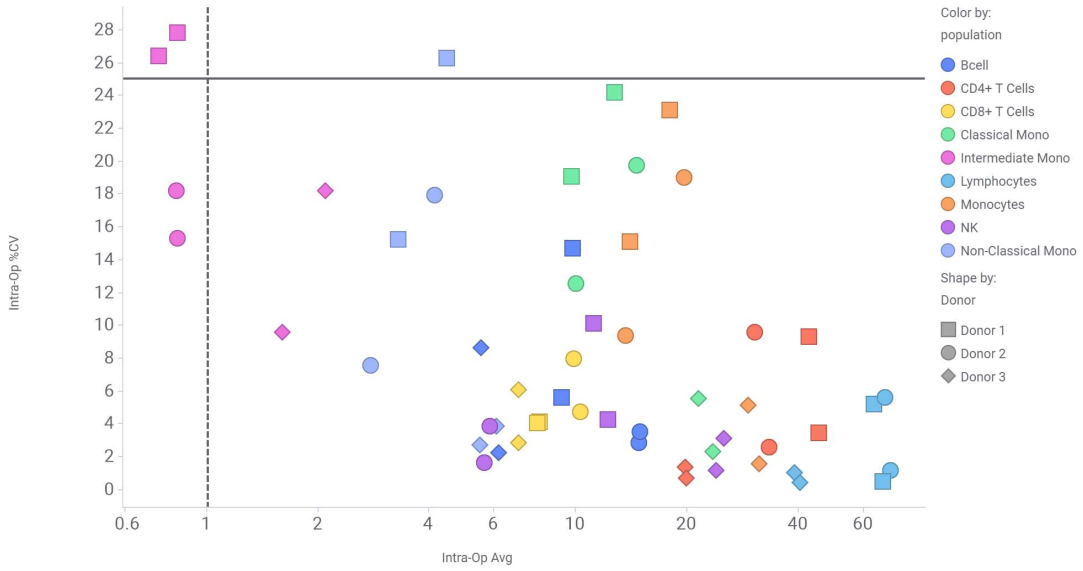

Figure 3A. Intra-Assay Precision for Major Subsets. %CV is plotted versus the average of the 3 replicates for each cellular subset refer to percent of PBMC. Each data point represents a unique cell subset (Table 6). Shapes represent the different blood donors analyzed. Reference lines at 1% on the x-axis and 25% CV on the y-axis are shown for guidance for the recommended criteria.

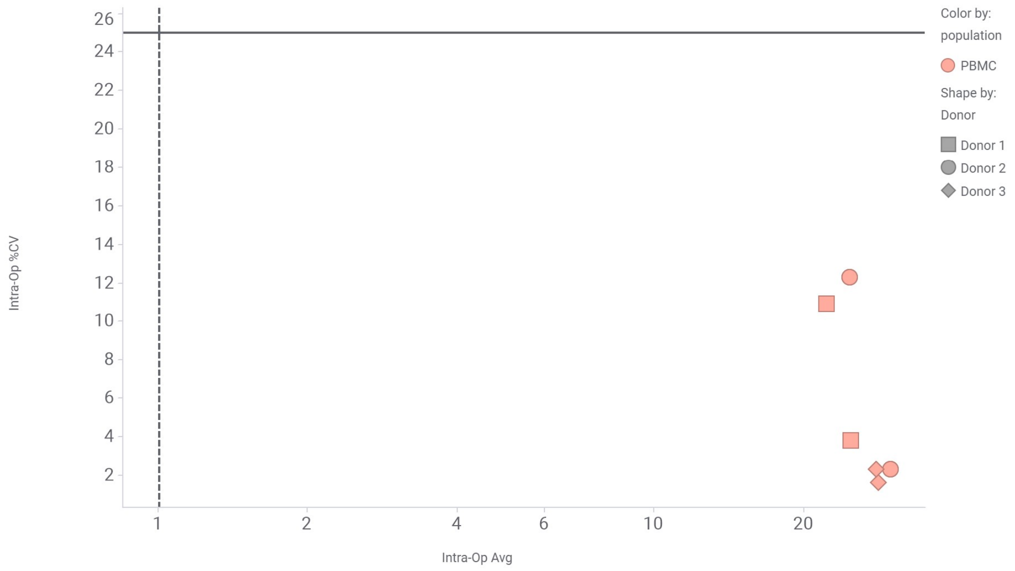

Figure 3B. Intra-Assay Precision for Major Subsets. %CV is plotted versus the average of the 3 replicates of PBMC refer the percent of WBC. Each data point represents a unique cell subset (Table 6). Shapes represent the different blood donors analyzed. Reference lines at 1% on the x-axis and 25% CV on the y-axis are shown for guidance for the recommended criteria.

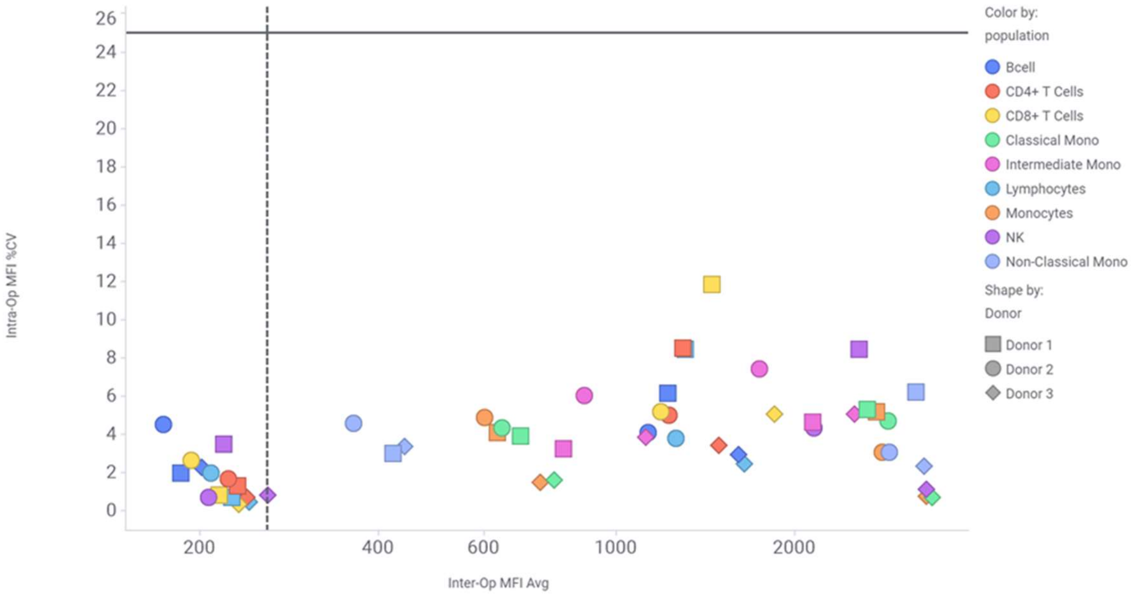

Figure 4A. Intra-Assay Precision for Readout Markers. %CV is plotted versus the average of the 3 replicates of IRAK4 Readouts for each subset refer to MFI values. Each data point represents a unique cell subset (Table 7). Shapes represent the different blood donors analyzed. Reference lines at 250 MFI on the x-axis and 25% CV on the y-axis are shown for guidance for the recommended criteria.

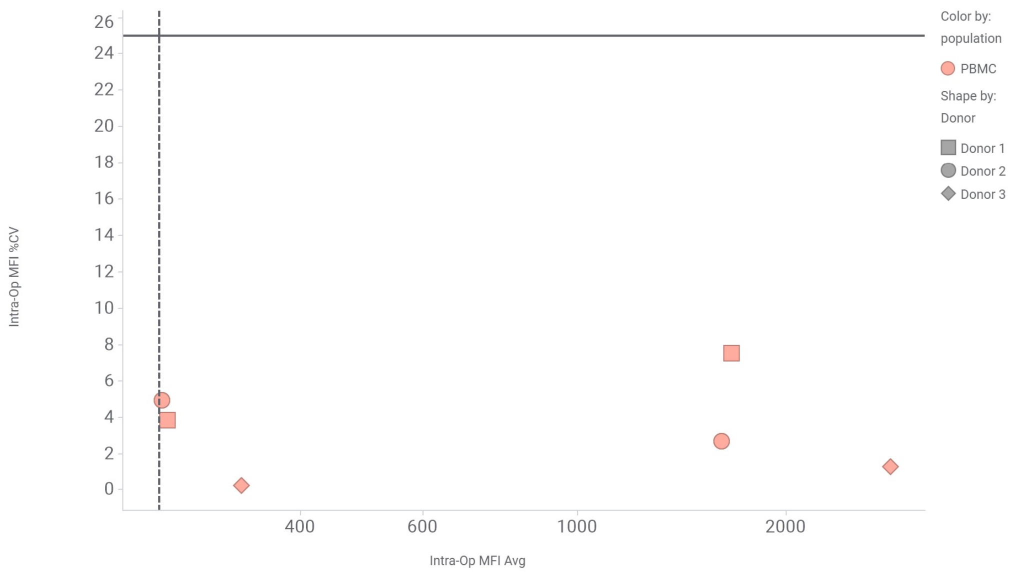

Figure 4B. Intra-Assay Precision for Readout Markers. %CV is plotted versus the average of the 3 replicates of IRAK4 Readout for PBMC subset refer to MFI values. Each data point represents a unique cell subset (Table 7). Shapes represent the different blood donors analyzed. Reference lines at 250 MFI on the x-axis and 25% CV on the y-axis are shown for guidance for the recommended criteria.

Table 10. Intra-Assay Precision for Major cell subsets

Population	Parent Population	Intra-Assay %CV	Intra-Assay Average	Donor	Condition	
Bcell[1]	CD3neg	5.62	9.11	Donor 1	Blocked	Qualified
CD4+ T Cells[1]	T Cells	9.34	42.46	Donor 1	Blocked	Qualified
CD8+ T Cells[1]	T Cells	4.09	7.83	Donor 1	Blocked	Qualified
Classical Mono[1]	Monocytes	24.19	12.68	Donor 1	Blocked	Qualified
Intermediate Mono[1]	Monocytes	26.46	0.74	Donor 1	Blocked	Non-Qualifed
Lymphocytes[1]	CD14neg	5.24	64.00	Donor 1	Blocked	Qualified
Monocytes[1]	CD19-	23.12	17.95	Donor 1	Blocked	Qualified
NK[1]	CD14neg	4.27	12.15	Donor 1	Blocked	Qualified
Non-Classical Mono[1]	Monocytes	26.31	4.46	Donor 1	Blocked	Non-Qualifed
PBMC[2]	Non-Neutrophils	10.97	22.15	Donor 1	Blocked	Qualified
Bcell[1]	CD3neg	14.70	9.78	Donor 1	Unblocked	Qualified
CD4+ T Cells[1]	T Cells	3.49	45.39	Donor 1	Unblocked	Qualified
CD8+ T Cells[1]	T Cells	4.15	7.95	Donor 1	Unblocked	Qualified
Classical Mono[1]	Monocytes	19.10	9.72	Donor 1	Unblocked	Qualified
Intermediate Mono[1]	Monocytes	27.86	0.83	Donor 1	Unblocked	Non-Qualifed

Lymphocytes[1]	CD14neg	0.49	67.42	Donor 1	Unblocked	Qualified
Monocytes[1]	CD19-	15.13	13.93	Donor 1	Unblocked	Qualified
NK[1]	CD14neg	10.18	11.15	Donor 1	Unblocked	Qualified
Non-Classical Mono[1]	Monocytes	15.28	3.30	Donor 1	Unblocked	Qualified
PBMC[2]	Non-Neutrophils	3.81	24.89	Donor 1	Unblocked	Qualified
Bcell[1]	CD3neg	3.53	14.88	Donor 2	Blocked	Qualified
CD4+ T Cells[1]	T Cells	9.59	30.37	Donor 2	Blocked	Qualified
CD8+ T Cells[1]	T Cells	8.01	9.82	Donor 2	Blocked	Qualified
Classical Mono[1]	Monocytes	19.79	14.55	Donor 2	Blocked	Qualified
Intermediate Mono[1]	Monocytes	18.21	0.83	Donor 2	Blocked	Qualified
Lymphocytes[1]	CD14neg	5.67	68.50	Donor 2	Blocked	Qualified
Monocytes[1]	CD19-	19.01	19.55	Donor 2	Blocked	Qualified
NK[1]	CD14neg	3.90	5.84	Donor 2	Blocked	Qualified
Non-Classical Mono[1]	Monocytes	17.98	4.12	Donor 2	Blocked	Qualified
PBMC[2]	Non-Neutrophils	12.29	24.70	Donor 2	Blocked	Qualified
Bcell[1]	CD3neg	2.90	14.75	Donor 2	Unblocked	Qualified
CD4+ T Cells[1]	T Cells	2.64	33.30	Donor 2	Unblocked	Qualified
CD8+ T Cells[1]	T Cells	4.75	10.21	Donor 2	Unblocked	Qualified
Classical Mono[1]	Monocytes	12.58	9.94	Donor 2	Unblocked	Qualified
Intermediate Mono[1]	Monocytes	15.33	0.83	Donor 2	Unblocked	Qualified
Lymphocytes[1]	CD14neg	1.20	70.85	Donor 2	Unblocked	Qualified
Monocytes[1]	CD19-	9.41	13.62	Donor 2	Unblocked	Qualified
NK[1]	CD14neg	1.70	5.64	Donor 2	Unblocked	Qualified
Non-Classical Mono[1]	Monocytes	7.60	2.76	Donor 2	Unblocked	Qualified
PBMC[2]	Non-Neutrophils	2.33	29.87	Donor 2	Unblocked	Qualified
Bcell[1]	CD3neg	2.29	6.15	Donor 3	Blocked	Qualified
CD4+ T Cells[1]	T Cells	0.72	19.85	Donor 3	Blocked	Qualified
CD8+ T Cells[1]	T Cells	2.85	6.95	Donor 3	Blocked	Qualified
Classical Mono[1]	Monocytes	2.32	23.39	Donor 3	Blocked	Qualified
Intermediate Mono[1]	Monocytes	9.61	1.60	Donor 3	Blocked	Qualified
Lymphocytes[1]	CD14neg	0.46	40.26	Donor 3	Blocked	Qualified
Monocytes[1]	CD19-	1.62	31.24	Donor 3	Blocked	Qualified
NK[1]	CD14neg	1.18	23.85	Donor 3	Blocked	Qualified
Non-Classical Mono[1]	Monocytes	3.91	6.07	Donor 3	Blocked	Qualified
PBMC[2]	Non-Neutrophils	2.34	28.00	Donor 3	Blocked	Qualified
Bcell[1]	CD3neg	8.68	5.51	Donor 3	Unblocked	Qualified
CD4+ T Cells[1]	T Cells	1.41	19.75	Donor 3	Unblocked	Qualified

CD8+ T Cells[1]	T Cells	6.13	6.99	Donor 3	Unblocked	Qualified
Classical Mono[1]	Monocytes	5.55	21.46	Donor 3	Unblocked	Qualified
Intermediate Mono[1]	Monocytes	18.26	2.09	Donor 3	Unblocked	Qualified
Lymphocytes[1]	CD14neg	1.07	38.93	Donor 3	Unblocked	Qualified
Monocytes[1]	CD19-	5.16	29.15	Donor 3	Unblocked	Qualified
NK[1]	CD14neg	3.14	25.14	Donor 3	Unblocked	Qualified
Non-Classical Mono[1]	Monocytes	2.73	5.46	Donor 3	Unblocked	Qualified
PBMC[2]	Non-Neutrophils	1.65	28.17	Donor 3	Unblocked	Qualified

[1]CV was determined based on percent of PBMC
[2]CV was determined based on percent of WBC

Table 11. Intra-Assay Precision for Readout Populations.

Population	Parent Population	Intra-Op %CV	Intra-Op Average	Donor	Condition	
Bcell[1]	CD3neg	2.02	180.26	Donor 1	Blocked	Qualified
CD4+ T Cells[1]	T Cells	1.35	225.05	Donor 1	Blocked	Qualified
CD8+ T Cells[1]	T Cells	0.83	206.45	Donor 1	Blocked	Qualified
Classical Mono[1]	Monocytes	3.94	690.08	Donor 1	Blocked	Qualified
Intermediate Mono[1]	Monocytes	3.28	820.96	Donor 1	Blocked	Qualified
Lymphocytes[1]	CD14neg	0.70	220.95	Donor 1	Blocked	Qualified
Monocytes[1]	CD19-	4.09	631.59	Donor 1	Blocked	Qualified
NK[1]	CD14neg	3.48	210.21	Donor 1	Blocked	Qualified
Non-Classical Mono[1]	Monocytes	3.05	418.54	Donor 1	Blocked	Qualified
PBMC[1]	Non-Neutrophils	3.83	257.83	Donor 1	Blocked	Qualified
Bcell[1]	CD3neg	6.15	1300.73	Donor 1	Unblocked	Qualified
CD4+ T Cells[1]	T Cells	8.53	1403.65	Donor 1	Unblocked	Qualified
CD8+ T Cells[1]	T Cells	11.85	1608.95	Donor 1	Unblocked	Qualified
Classical Mono[1]	Monocytes	5.34	2803.56	Donor 1	Unblocked	Qualified
Intermediate Mono[1]	Monocytes	4.67	2006.80	Donor 1	Unblocked	Qualified
Lymphocytes[1]	CD14neg	8.50	1418.69	Donor 1	Unblocked	Qualified
Monocytes[1]	CD19-	5.21	2894.16	Donor 1	Unblocked	Qualified
NK[1]	CD14neg	8.50	2783.74	Donor 1	Unblocked	Qualified
Non-Classical Mono[1]	Monocytes	6.23	3367.67	Donor 1	Unblocked	Qualified
PBMC[1]	Non-Neutrophils	7.59	1664.84	Donor 1	Unblocked	Qualified
Bcell[1]	CD3neg	4.51	173.85	Donor 2	Blocked	Qualified
CD4+ T Cells[1]	T Cells	1.69	223.76	Donor 2	Blocked	Qualified
CD8+ T Cells[1]	T Cells	2.69	192.64	Donor 2	Blocked	Qualified

Classical Mono[1]	Monocytes	4.35	662.16	Donor 2	Blocked	Qualified
Intermediate Mono[1]	Monocytes	6.06	900.26	Donor 2	Blocked	Qualified
Lymphocytes[1]	CD14neg	2.02	211.03	Donor 2	Blocked	Qualified
Monocytes[1]	CD19-	4.93	616.46	Donor 2	Blocked	Qualified
NK[1]	CD14neg	0.70	207.32	Donor 2	Blocked	Qualified
Non-Classical Mono[1]	Monocytes	4.60	362.38	Donor 2	Blocked	Qualified
PBMC[1]	Non-Neutrophils	4.96	252.95	Donor 2	Blocked	Qualified
Bcell[1]	CD3neg	4.11	1220.44	Donor 2	Unblocked	Qualified
CD4+ T Cells[1]	T Cells	5.03	1384.13	Donor 2	Unblocked	Qualified
CD8+ T Cells[1]	T Cells	5.20	1366.62	Donor 2	Unblocked	Qualified
Classical Mono[1]	Monocytes	4.69	3089.47	Donor 2	Unblocked	Qualified
Intermediate Mono[1]	Monocytes	7.43	1728.06	Donor 2	Unblocked	Qualified
Lymphocytes[1]	CD14neg	3.78	1409.98	Donor 2	Unblocked	Qualified
Monocytes[1]	CD19-	3.09	2983.42	Donor 2	Unblocked	Qualified
NK[1]	CD14neg	4.33	2314.17	Donor 2	Unblocked	Qualified
Non-Classical Mono[1]	Monocytes	3.07	2988.59	Donor 2	Unblocked	Qualified
PBMC[1]	Non-Neutrophils	2.72	1613.11	Donor 2	Unblocked	Qualified
Bcell[1]	CD3neg	2.31	192.91	Donor 3	Blocked	Qualified
CD4+ T Cells[1]	T Cells	0.74	238.41	Donor 3	Blocked	Qualified
CD8+ T Cells[1]	T Cells	0.36	221.76	Donor 3	Blocked	Qualified
Classical Mono[1]	Monocytes	1.63	749.17	Donor 3	Blocked	Qualified
Intermediate Mono[1]	Monocytes	3.86	1033.23	Donor 3	Blocked	Qualified
Lymphocytes[1]	CD14neg	0.50	238.91	Donor 3	Blocked	Qualified
Monocytes[1]	CD19-	1.53	706.61	Donor 3	Blocked	Qualified
NK[1]	CD14neg	0.82	251.47	Donor 3	Blocked	Qualified
Non-Classical Mono[1]	Monocytes	3.36	423.61	Donor 3	Blocked	Qualified
PBMC[1]	Non-Neutrophils	0.27	329.33	Donor 3	Blocked	Qualified
Bcell[1]	CD3neg	2.98	1666.95	Donor 3	Unblocked	Qualified
CD4+ T Cells[1]	T Cells	3.44	1681.25	Donor 3	Unblocked	Qualified
CD8+ T Cells[1]	T Cells	5.09	2083.50	Donor 3	Unblocked	Qualified
Classical Mono[1]	Monocytes	0.75	3674.42	Donor 3	Unblocked	Qualified
Intermediate Mono[1]	Monocytes	5.06	2516.14	Donor 3	Unblocked	Qualified
Lymphocytes[1]	CD14neg	2.47	1819.77	Donor 3	Unblocked	Qualified
Monocytes[1]	CD19-	0.81	3567.88	Donor 3	Unblocked	Qualified
NK[1]	CD14neg	1.18	3492.10	Donor 3	Unblocked	Qualified
Non-Classical Mono[1]	Monocytes	2.34	3487.54	Donor 3	Unblocked	Qualified

PBMC[1]	Non-Neutrophils	1.33	2815.90	Donor 3	Unblocked	Qualified

[1] *CV was determined based on MFI*

7.1.2. INTER-OPERATOR PRECISION

For inter-operator precision, three (3) donor samples, assassed in triplicate were stained by two operators (total number of replicates 6), and the %CV was calculated for the six replicates run by the two operators for both block and unblock condition using IRAK4 blocker and for each cell subset as a percentage PBMC except for PBMC population where percentage was calculate as percentage of WBC. For readouts key metric was median fluorescence intensity (MFI).

For major cell subsets assessed in the panel (Figure 5A-B and Table 12) Donor 2 and Donor 3 showed 18 fo 20 cellular subsets with a %CV below 25%. Donor 1 displayed 19 of 20 cellular subsets with a %CV below 25. All cellular subsets for Donor 3 and 18 cellular subsets for Donor 2 and Donor 1 demostrated an abundance of greater than 1% of total viable leukocytes.

Regarding readouts assessed in the intra-operator precision experiment, all readouts (20 in total) for Donor 1 and Donor 2 and had a %CV below 25%. For Donor 3 19 of 20 readouts showed a %CV lower than 25% (Figure 6A-B and Table 12).

Therefore, the results indicate that the assay has acceptable Inter-Operator Precision.

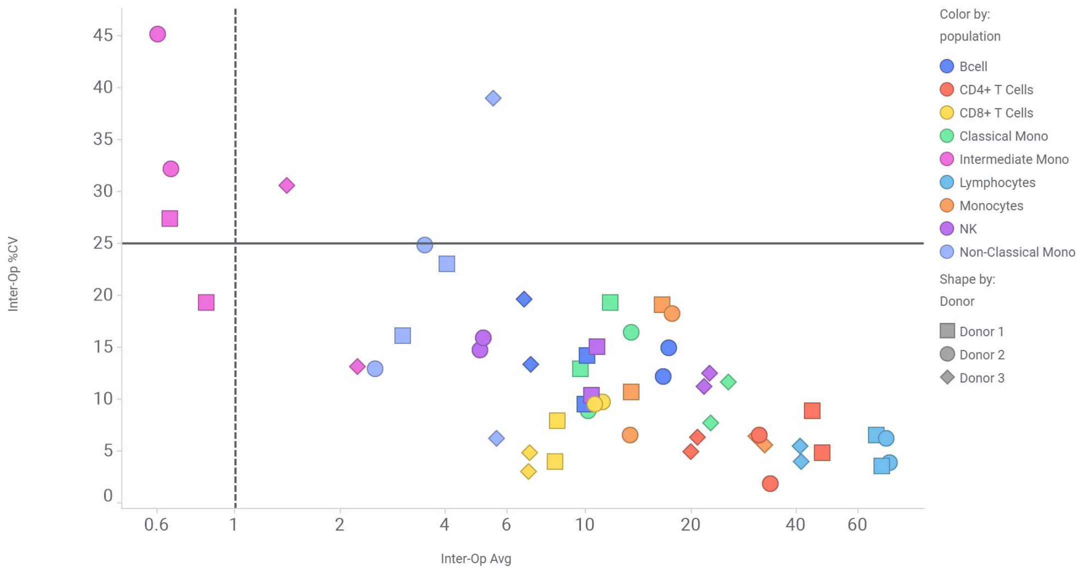

Figure 5A. Inter-Operator Precision for Major Subsets. %CV is plotted versus the average of the 6 replicates for each cellular subset refer to percent of PBMC. Each data point represents a unique cell subset (Table 6). Shapes represent the different blood donors analyzed. Reference lines at 1% on the x-axis and 25% CV on the y-axis are shown for guidance for the recommended criteria.

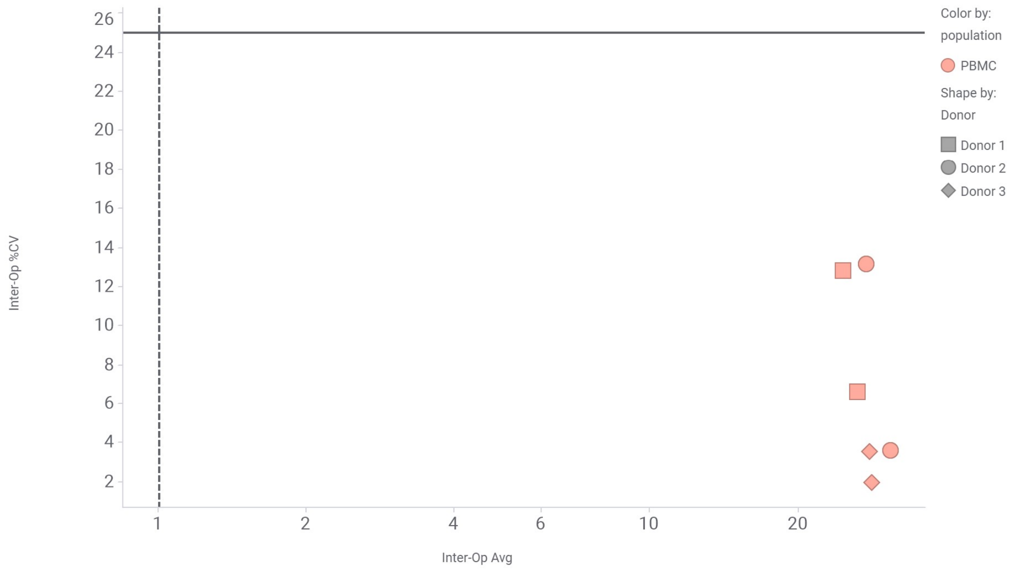

Figure 5B. Inter-Operator Precision for Major Subsets. %CV is plotted versus the average of the 6 replicates of PBMC refer the percent of WBC. Each data point represents a unique cell subset (Table 6). Shapes represent the different blood donors analyzed. Reference lines at 1% on the x-axis and 25% CV on the y-axis are shown for guidance for the recommended criteria.

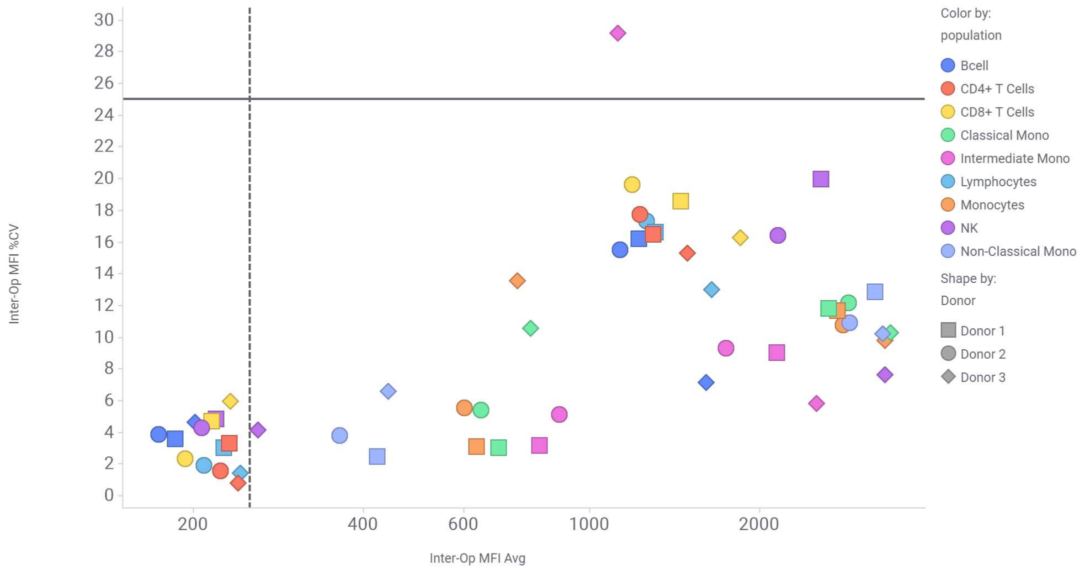

Figure 6A. Inter-Operator Precision for Readout Markers. %CV is plotted versus the average of the 6 replicates of IRAK4 Readouts for each subset refer to MFI values. Each data point represents a unique cell subset (Table 7). Shapes represent the different blood donors analyzed. Reference lines at 250 MFI on the x-axis and 25% CV on the y-axis are shown for guidance for the recommended criteria.

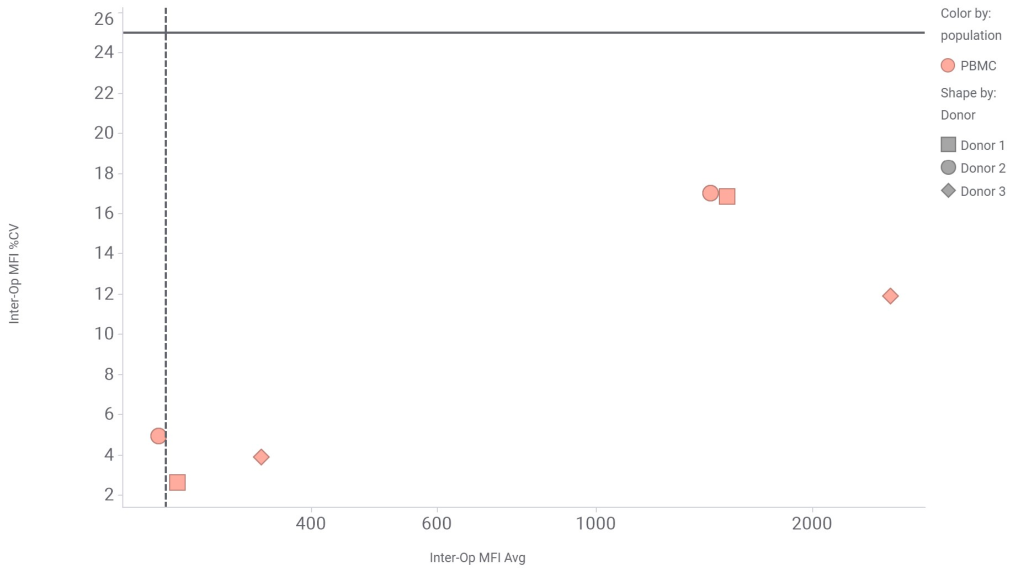

Figure 6B. Inter-Operator Precision for Readout Markers. %CV is plotted versus the average of the 6 replicates of IRAK4 Readout for PBMC subset refer to MFI values. Each data point represents a unique cell subset (Table 7). Shapes represent the different blood donors analyzed. Reference lines at 250 MFI on the x-axis and 25% CV on the y-axis are shown for guidance for the recommended criteria.

Table 12. Inter-Operator Precision for Major Cell Subsets

Population	Parent Population	Inter-Op %CV	Inter-Op Average	Donor	Condition	
Bcell[1]	CD3neg	14.27	10.09	Donor 1	Blocked	Qualified
CD4+ T Cells[1]	T Cells	8.87	44.10	Donor 1	Blocked	Qualified
CD8+ T Cells[1]	T Cells	7.98	8.31	Donor 1	Blocked	Qualified
Classical Mono[1]	Monocytes	19.31	11.74	Donor 1	Blocked	Qualified
Intermediate Mono[1]	Monocytes	27.47	0.65	Donor 1	Blocked	Non-Qualifed
Lymphocytes[1]	CD14neg	6.59	67.54	Donor 1	Blocked	Qualified
Monocytes[1]	CD19-	19.12	16.44	Donor 1	Blocked	Qualified
NK[1]	CD14neg	15.09	10.80	Donor 1	Blocked	Qualified
Non-Classical Mono[1]	Monocytes	23.04	4.00	Donor 1	Blocked	Qualified
PBMC[2]	Non-Neutrophils	12.81	24.60	Donor 1	Blocked	Qualified
Bcell[1]	CD3neg	9.52	9.96	Donor 1	Unblocked	Qualified
CD4+ T Cells[1]	T Cells	4.90	47.28	Donor 1	Unblocked	Qualified
CD8+ T Cells[1]	T Cells	4.02	8.18	Donor 1	Unblocked	Qualified
Classical Mono[1]	Monocytes	12.93	9.63	Donor 1	Unblocked	Qualified

Intermediate Mono[1]	Monocytes	19.32	0.83	Donor 1	Unblocked	Qualified
Lymphocytes[1]	CD14neg	3.62	69.67	Donor 1	Unblocked	Qualified
Monocytes[1]	CD19-	10.70	13.51	Donor 1	Unblocked	Qualified
NK[1]	CD14neg	10.43	10.41	Donor 1	Unblocked	Qualified
Non-Classical Mono[1]	Monocytes	16.14	3.00	Donor 1	Unblocked	Qualified
PBMC[2]	Non-Neutrophils	6.61	26.38	Donor 1	Unblocked	Qualified
Bcell[1]	CD3neg	14.96	17.18	Donor 2	Blocked	Qualified
CD4+ T Cells[1]	T Cells	6.60	31.19	Donor 2	Blocked	Qualified
CD8+ T Cells[1]	T Cells	9.59	10.62	Donor 2	Blocked	Qualified
Classical Mono[1]	Monocytes	16.44	13.43	Donor 2	Blocked	Qualified
Intermediate Mono[1]	Monocytes	45.25	0.60	Donor 2	Blocked	Non-Qualifed
Lymphocytes[1]	CD14neg	6.29	71.96	Donor 2	Blocked	Qualified
Monocytes[1]	CD19-	18.33	17.56	Donor 2	Blocked	Qualified
NK[1]	CD14neg	15.94	5.12	Donor 2	Blocked	Qualified
Non-Classical Mono[1]	Monocytes	24.90	3.47	Donor 2	Blocked	Qualified
PBMC[2]	Non-Neutrophils	13.18	27.49	Donor 2	Blocked	Qualified
Bcell[1]	CD3neg	12.21	16.57	Donor 2	Unblocked	Qualified
CD4+ T Cells[1]	T Cells	1.88	33.57	Donor 2	Unblocked	Qualified
CD8+ T Cells[1]	T Cells	9.72	11.15	Donor 2	Unblocked	Qualified
Classical Mono[1]	Monocytes	8.86	10.18	Donor 2	Unblocked	Qualified
Intermediate Mono[1]	Monocytes	32.26	0.66	Donor 2	Unblocked	Non-Qualifed
Lymphocytes[1]	CD14neg	3.91	73.42	Donor 2	Unblocked	Qualified
Monocytes[1]	CD19-	6.54	13.42	Donor 2	Unblocked	Qualified
NK[1]	CD14neg	14.74	4.99	Donor 2	Unblocked	Qualified
Non-Classical Mono[1]	Monocytes	12.93	2.52	Donor 2	Unblocked	Qualified
PBMC[2]	Non-Neutrophils	3.62	30.78	Donor 2	Unblocked	Qualified
Bcell[1]	CD3neg	13.36	6.97	Donor 3	Blocked	Qualified
CD4+ T Cells[1]	T Cells	5.02	19.93	Donor 3	Blocked	Qualified
CD8+ T Cells[1]	T Cells	3.09	6.87	Donor 3	Blocked	Qualified
Classical Mono[1]	Monocytes	11.67	25.49	Donor 3	Blocked	Qualified
Intermediate Mono[1]	Monocytes	30.68	1.40	Donor 3	Blocked	Non-Qualifed
Lymphocytes[1]	CD14neg	3.96	41.10	Donor 3	Blocked	Qualified
Monocytes[1]	CD19-	5.60	32.49	Donor 3	Blocked	Qualified
NK[1]	CD14neg	11.22	21.66	Donor 3	Blocked	Qualified
Non-Classical Mono[1]	Monocytes	39.04	5.47	Donor 3	Blocked	Non-Qualifed
PBMC[2]	Non-Neutrophils	3.56	27.86	Donor 3	Blocked	Qualified

Bcell[1]	CD3neg	19.69	6.66	Donor 3	Unblocked	Qualified
CD4+ T Cells[1]	T Cells	6.38	20.79	Donor 3	Unblocked	Qualified
CD8+ T Cells[1]	T Cells	4.83	6.90	Donor 3	Unblocked	Qualified
Classical Mono[1]	Monocytes	7.70	22.67	Donor 3	Unblocked	Qualified
Intermediate Mono[1]	Monocytes	13.13	2.23	Donor 3	Unblocked	Qualified
Lymphocytes[1]	CD14neg	5.47	40.87	Donor 3	Unblocked	Qualified
Monocytes[1]	CD19-	6.43	30.63	Donor 3	Unblocked	Qualified
NK[1]	CD14neg	12.58	22.60	Donor 3	Unblocked	Qualified
Non-Classical Mono[1]	Monocytes	6.21	5.57	Donor 3	Unblocked	Qualified
PBMC[2]	Non-Neutrophils	1.98	28.18	Donor 3	Unblocked	Qualified

[1]CV was determined based on percent of PBMC
[2]CV was determined based on percent of WBC

Table 13. Inter-Operator Precision for Readouts

Population	Parent Population	Inter-Op %CV	Inter-Op Average	Donor	Condition	
Bcell[1]	CD3neg	3.60	185.32	Donor 1	Blocked	Qualified
CD4+ T Cells[1]	T Cells	3.37	231.39	Donor 1	Blocked	Qualified
CD8+ T Cells[1]	T Cells	4.76	214.80	Donor 1	Blocked	Qualified
Classical Mono[1]	Monocytes	3.06	689.65	Donor 1	Blocked	Qualified
Intermediate Mono[1]	Monocytes	3.21	815.71	Donor 1	Blocked	Qualified
Lymphocytes[1]	CD14neg	3.04	226.37	Donor 1	Blocked	Qualified
Monocytes[1]	CD19-	3.16	629.86	Donor 1	Blocked	Qualified
NK[1]	CD14neg	4.91	218.68	Donor 1	Blocked	Qualified
Non-Classical Mono[1]	Monocytes	2.48	422.11	Donor 1	Blocked	Qualified
PBMC[1]	Non-Neutrophils	2.66	259.95	Donor 1	Blocked	Qualified
Bcell[1]	CD3neg	16.26	1217.89	Donor 1	Unblocked	Qualified
CD4+ T Cells[1]	T Cells	16.52	1293.50	Donor 1	Unblocked	Qualified
CD8+ T Cells[1]	T Cells	18.61	1444.37	Donor 1	Unblocked	Qualified
Classical Mono[1]	Monocytes	11.85	2637.08	Donor 1	Unblocked	Qualified
Intermediate Mono[1]	Monocytes	9.04	2138.69	Donor 1	Unblocked	Qualified
Lymphocytes[1]	CD14neg	16.65	1306.64	Donor 1	Unblocked	Qualified
Monocytes[1]	CD19-	11.70	2734.14	Donor 1	Unblocked	Qualified
NK[1]	CD14neg	20.00	2555.34	Donor 1	Unblocked	Qualified
Non-Classical Mono[1]	Monocytes	12.92	3187.51	Donor 1	Unblocked	Qualified
PBMC[1]	Non-Neutrophils	16.85	1520.15	Donor 1	Unblocked	Qualified
Bcell[1]	CD3neg	3.93	173.42	Donor 2	Blocked	Qualified

CD4+ T Cells[1]	T Cells	1.62	223.00	Donor 2	Blocked	Qualified
CD8+ T Cells[1]	T Cells	2.39	193.53	Donor 2	Blocked	Qualified
Classical Mono[1]	Monocytes	5.44	643.35	Donor 2	Blocked	Qualified
Intermediate Mono[1]	Monocytes	5.14	885.21	Donor 2	Blocked	Qualified
Lymphocytes[1]	CD14neg	1.98	208.82	Donor 2	Blocked	Qualified
Monocytes[1]	CD19-	5.55	599.33	Donor 2	Blocked	Qualified
NK[1]	CD14neg	4.33	206.43	Donor 2	Blocked	Qualified
Non-Classical Mono[1]	Monocytes	3.87	361.87	Donor 2	Blocked	Qualified
PBMC[1]	Non-Neutrophils	4.98	244.79	Donor 2	Blocked	Qualified
Bcell[1]	CD3neg	15.57	1130.76	Donor 2	Unblocked	Qualified
CD4+ T Cells[1]	T Cells	17.79	1227.27	Donor 2	Unblocked	Qualified
CD8+ T Cells[1]	T Cells	19.69	1184.84	Donor 2	Unblocked	Qualified
Classical Mono[1]	Monocytes	12.21	2857.39	Donor 2	Unblocked	Qualified
Intermediate Mono[1]	Monocytes	9.35	1737.82	Donor 2	Unblocked	Qualified
Lymphocytes[1]	CD14neg	17.36	1258.75	Donor 2	Unblocked	Qualified
Monocytes[1]	CD19-	10.80	2801.98	Donor 2	Unblocked	Qualified
NK[1]	CD14neg	16.48	2150.51	Donor 2	Unblocked	Qualified
Non-Classical Mono[1]	Monocytes	10.97	2866.98	Donor 2	Unblocked	Qualified
PBMC[1]	Non-Neutrophils	17.04	1441.85	Donor 2	Unblocked	Qualified
Bcell[1]	CD3neg	4.70	200.65	Donor 3	Blocked	Qualified
CD4+ T Cells[1]	T Cells	0.84	239.80	Donor 3	Blocked	Qualified
CD8+ T Cells[1]	T Cells	5.99	232.15	Donor 3	Blocked	Qualified
Classical Mono[1]	Monocytes	10.62	786.00	Donor 3	Blocked	Qualified
Intermediate Mono[1]	Monocytes	29.22	1120.05	Donor 3	Blocked	Non-Qualifed
Lymphocytes[1]	CD14neg	1.46	241.88	Donor 3	Blocked	Qualified
Monocytes[1]	CD19-	13.57	744.30	Donor 3	Blocked	Qualified
NK[1]	CD14neg	4.19	259.83	Donor 3	Blocked	Qualified
Non-Classical Mono[1]	Monocytes	6.66	440.08	Donor 3	Blocked	Qualified
PBMC[1]	Non-Neutrophils	3.94	340.08	Donor 3	Blocked	Qualified
Bcell[1]	CD3neg	7.15	1601.04	Donor 3	Unblocked	Qualified
CD4+ T Cells[1]	T Cells	15.37	1486.68	Donor 3	Unblocked	Qualified
CD8+ T Cells[1]	T Cells	16.32	1846.00	Donor 3	Unblocked	Qualified
Classical Mono[1]	Monocytes	10.34	3391.23	Donor 3	Unblocked	Qualified
Intermediate Mono[1]	Monocytes	5.86	2515.97	Donor 3	Unblocked	Qualified
Lymphocytes[1]	CD14neg	13.05	1640.81	Donor 3	Unblocked	Qualified
Monocytes[1]	CD19-	9.83	3315.86	Donor 3	Unblocked	Qualified
NK[1]	CD14neg	7.64	3312.13	Donor 3	Unblocked	Qualified

Non-Classical Mono[1]	Monocytes	10.25	3285.63	Donor 3	Unblocked	Qualified
PBMC[1]	Non-Neutrophils	11.96	2567.12	Donor 3	Unblocked	Qualified

[1]*CV was determined based on MFI*

7.1.3. INTER-INSTRUMENT PRECISION

For inter-instrument precision, one (1) donor sample was assassed in triplicate over two instruments by one operator and the %CV was calculated for the six replicates (one donor) tests across the two instruments and %CV was determined for each cell subset as a percentage of PBMC for all the subsets except for PBMC that was calculated as a percentage of WBC and for readouts key metric was median fluorescence intensity (MFI).

All major cell subsets assessed (20 of 20, Figure 7A-B and Table 14) had %CV below 25%. All cellular subsets demonstrated an abundance greater than 1% of total viable leukocytes.

Finally all readout populations assessed in the inter-instrument precision experiment as a function of MFI, all readouts (20 of 20) had a %CV less than 25% (Figure 8A-B and Table 15). Therefore, the results indicate that the assay has acceptable intra-operator Precision.

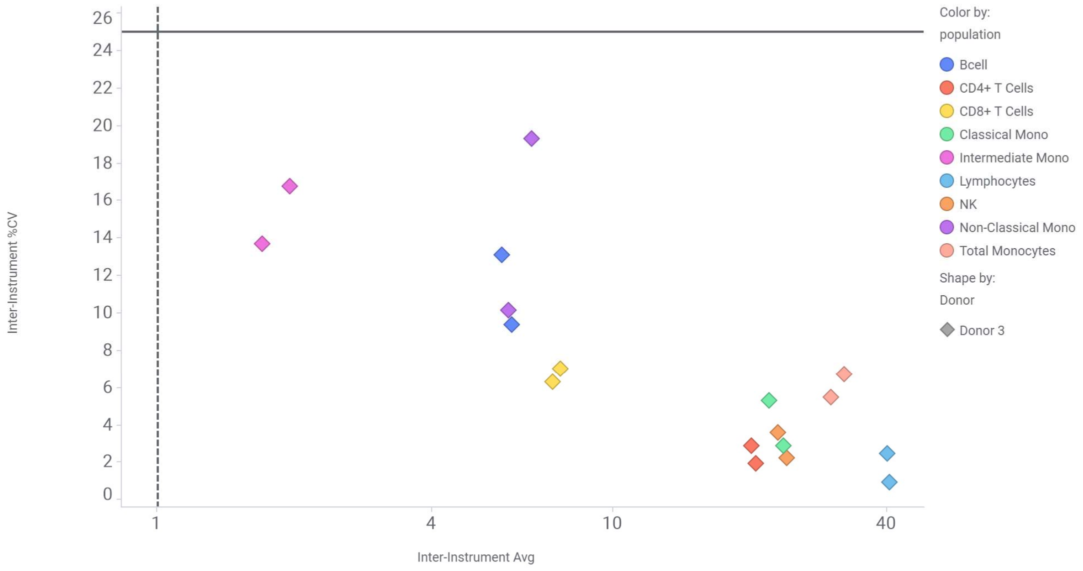

Figure 7A. Inter-Instrument Precision for Major Subsets. %CV is plotted versus the average of the 6 replicates for each cellular subset refer to percent of PBMC. Each data point represents a unique cell subset (Table 6). Shapes represent the different blood donors analyzed. Reference lines at 1% on the x-axis and 25% CV on the y-axis are shown for guidance for the recommended criteria.

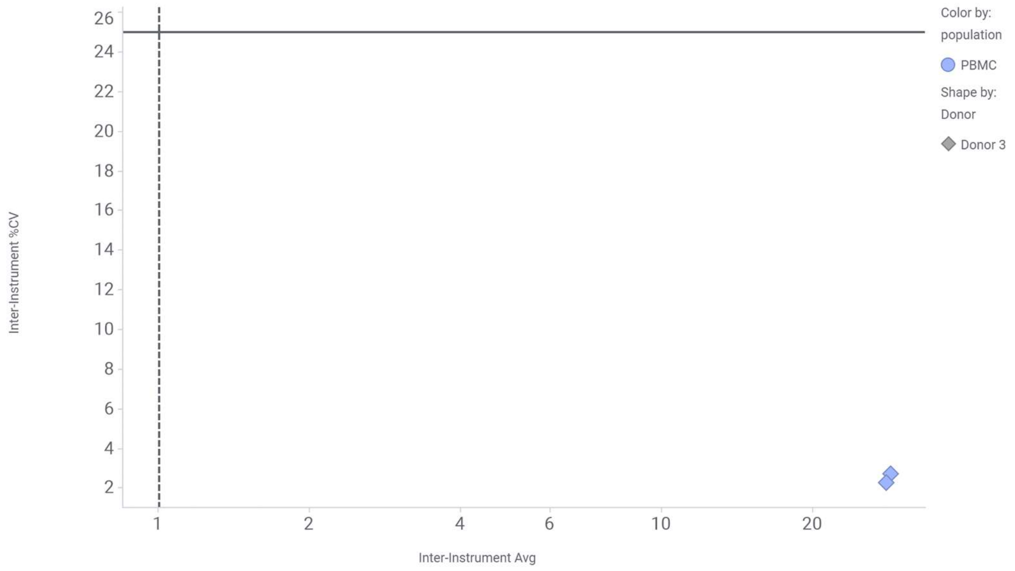

Figure 7B. Inter-Instrument Precision for Major Subsets. %CV is plotted versus the average of the 6 replicates of PBMC refer the percent of WBC. Each data point represents a unique cell subset (Table 6). Shapes represent the different blood donors analyzed. Reference lines at 1% on the x-axis and 25% CV on the y-axis are shown for guidance for the recommended criteria.

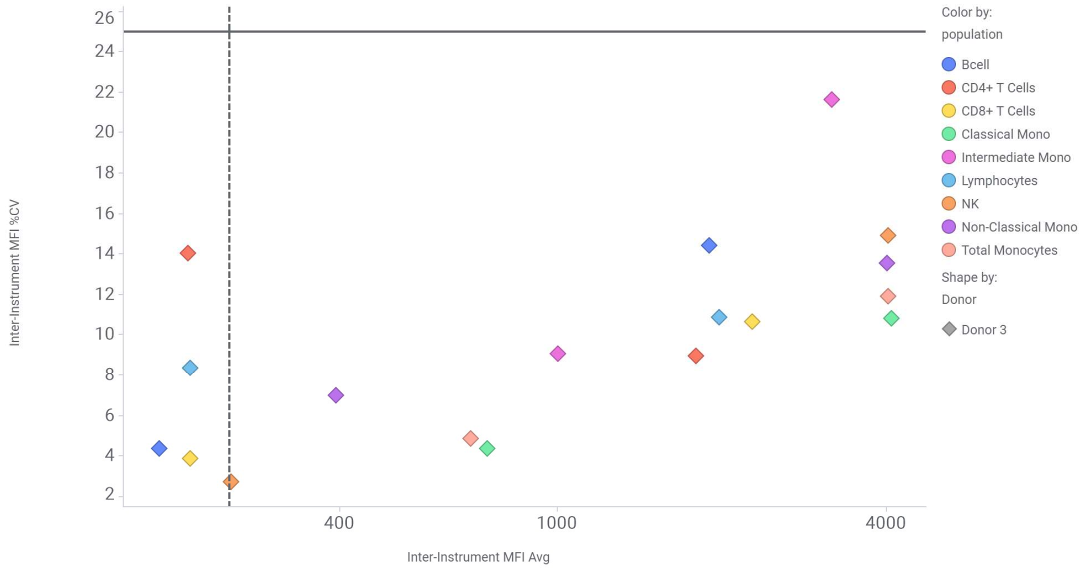

Figure 8A. Inter-Instrument Precision for Readout Markers. %CV is plotted versus the average of the 6 replicates of IRAK4 Readouts for each subset refer to MFI values. Each data point represents a unique cell subset (Table 7). Shapes represent the different blood donors analyzed. Reference lines at 250 MFI on the x-axis and 25% CV on the y-axis are shown for guidance for the recommended criteria.

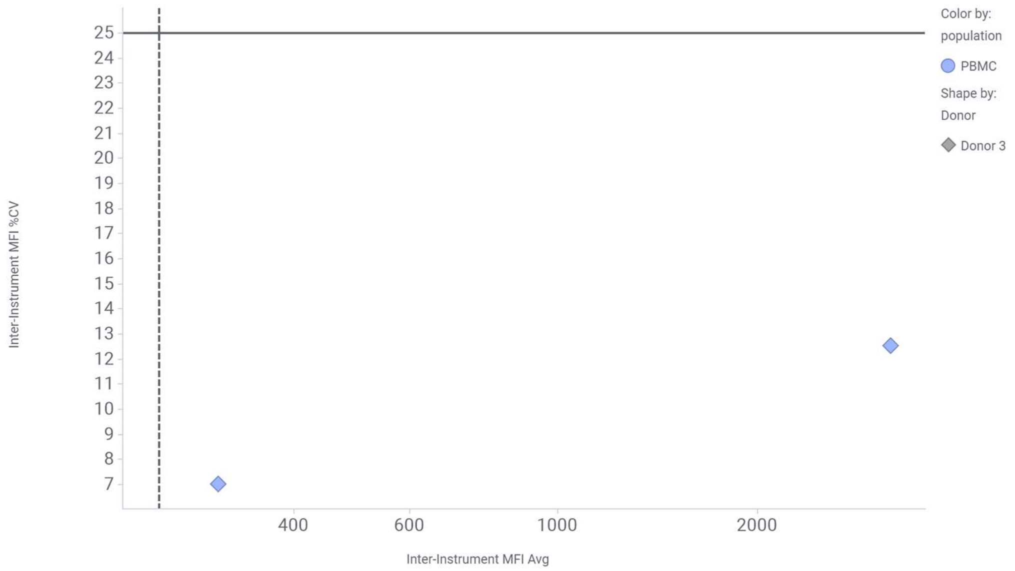

Figure 8B. Inter-Instrument Precision for Readout Markers. %CV is plotted versus the average of the 6 replicates of IRAK4 Readout for PBMC subset refer to MFI values. Each data point represents a unique cell subset (Table 7). Shapes represent the different blood donors analyzed. Reference lines at 250 MFI on the x-axis and 25% CV on the y-axis are shown for guidance for the recommended criteria.

Table 14. Inter-Instrument Precision for Major Cell Subsets

Population	Parent Population	Inter-Inst %CV	Inter-Inst Average	Donor	Condition	
Bcell[1]	CD3neg	9.38	5.98	Donor 3	Blocked	Qualified
CD4+ T Cells[1]	T Cells	2.89	20.14	Donor 3	Blocked	Qualified
CD8+ T Cells[1]	T Cells	6.35	7.35	Donor 3	Blocked	Qualified
Classical Mono[1]	Total Monocytes	2.94	23.62	Donor 3	Blocked	Qualified
Intermediate Mono[1]	Total Monocytes	13.70	1.70	Donor 3	Blocked	Qualified
Lymphocytes[1]	CD14neg	2.49	40.02	Donor 3	Blocked	Qualified
Monocytes[1]	CD19-	6.74	32.10	Donor 3	Blocked	Qualified
NK[1]	CD14neg	3.63	22.95	Donor 3	Blocked	Qualified
Non-Classical Mono[1]	Total Monocytes	19.32	6.61	Donor 3	Blocked	Qualified
PBMC[2]	Non-Neutrophils	2.31	27.88	Donor 3	Blocked	Qualified
Bcell[1]	CD3neg	13.15	5.69	Donor 3	Unblocked	Qualified

CD4+ T Cells[1]	T Cells	1.99	20.60	Donor 3	Unblocked	Qualified
CD8+ T Cells[1]	T Cells	7.02	7.67	Donor 3	Unblocked	Qualified
Classical Mono[1]	Total Monocytes	5.32	22.03	Donor 3	Unblocked	Qualified
Intermediate Mono[1]	Total Monocytes	16.79	1.95	Donor 3	Unblocked	Qualified
Lymphocytes[1]	CD14neg	0.97	40.37	Donor 3	Unblocked	Qualified
Monocytes[1]	CD19-	5.49	30.08	Donor 3	Unblocked	Qualified
NK[1]	CD14neg	2.28	24.00	Donor 3	Unblocked	Qualified
Non-Classical Mono[1]	Total Monocytes	10.15	5.90	Donor 3	Unblocked	Qualified
PBMC[2]	Non-Neutrophils	2.73	28.51	Donor 3	Unblocked	Qualified

[1]CV was determined based on percent of PBMC
[2]CV was determined based on percent of WBC

Table 15. Inter-Instrument Precision for Readouts

Population	Parent Population	Inter-Inst %CV	Inter-Inst Average	Donor	Condition	
Bcell[1]	CD3neg	4.40	186.37	Donor 3	Blocked	Qualified
CD4+ T Cells[1]	T Cells	14.09	210.32	Donor 3	Blocked	Qualified
CD8+ T Cells[1]	T Cells	3.91	212.05	Donor 3	Blocked	Qualified
Classical Mono[1]	Total Monocytes	4.38	742.95	Donor 3	Blocked	Qualified
Intermediate Mono[1]	Total Monocytes	9.11	996.86	Donor 3	Blocked	Qualified
Lymphocytes[1]	CD14neg	8.41	212.43	Donor 3	Blocked	Qualified
Monocytes[1]	CD19-	4.92	692.48	Donor 3	Blocked	Qualified
NK[1]	CD14neg	2.76	252.03	Donor 3	Blocked	Qualified
Non-Classical Mono[1]	Total Monocytes	7.04	392.58	Donor 3	Blocked	Qualified
PBMC[1]	Non-Neutrophils	7.05	307.58	Donor 3	Blocked	Qualified
Bcell[1]	CD3neg	14.47	1888.11	Donor 3	Unblocked	Qualified
CD4+ T Cells[1]	T Cells	8.98	1783.34	Donor 3	Unblocked	Qualified
CD8+ T Cells[1]	T Cells	10.68	2260.59	Donor 3	Unblocked	Qualified
Classical Mono[1]	Total Monocytes	10.82	4071.16	Donor 3	Unblocked	Qualified
Intermediate Mono[1]	Total Monocytes	21.67	3165.28	Donor 3	Unblocked	Qualified
Lymphocytes[1]	CD14neg	10.93	1972.81	Donor 3	Unblocked	Qualified
Monocytes[1]	CD19-	11.94	4007.28	Donor 3	Unblocked	Qualified
NK[1]	CD14neg	14.94	4010.51	Donor 3	Unblocked	Qualified
Non-Classical Mono[1]	Total Monocytes	13.57	3987.82	Donor 3	Unblocked	Qualified
PBMC[1]	Non-Neutrophils	12.55	3173.85	Donor 3	Unblocked	Qualified

[1]CV was determined based on MFI

DocuSign Envelope ID: 8ECCE282-5174-484C-8E06-7271AEF7B4F6

7.1.4. POST-DRAW STABILITY

For Post-Draw Stability, triplicate samples from three donors were assessed by one operator. Samples were stored at 4°C for 1, 2, 3 and 4 days post-drawn and then samples were lyse/fixed and storage at -80°C. Once processing was completed for all the timepoints samples were stained for block and unblock condition and aquired on the primary instrument. The average of the triplicates was calculated for each post-draw timepoint (1, 2, or 3 days post-draw) and compared to the average at Day 1 to determine the percent change from Day 1 (Figure 9, 10 and table 16 and 17)

Across the major subsets and the readout populations, nearly all populations showed changes less than 25% from Day 1 when analyzed at Day 3. However, by Day 4 Post-Draw some populations/readouts began to show larger changes in more than one donor.

Overall, the results suggest than running samples between 1 to 3 days Post-Draw is an acceptable window for dataacquisition.

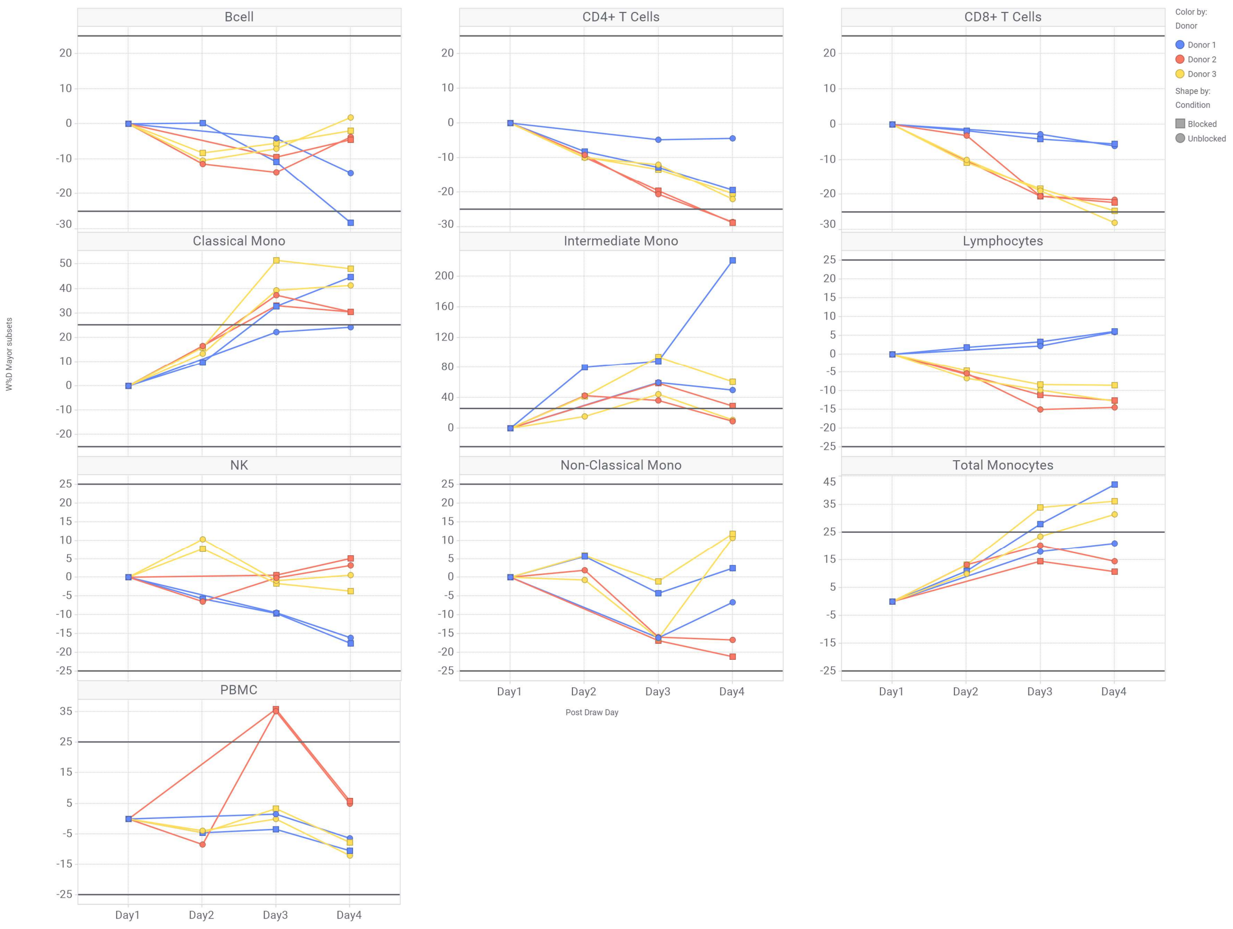

Figure 9. Post-Draw Stability. Major cell subset stability at Day 1, 2, 3 and 4. The percent change in the percent of PBMC/WBC from Day 1 was calculated. W%D Mayor subsets is plotted versus Days Post-Draw. Each graph represents a unique cell subset. Colors represents each donor for block and unblock condition. Reference lines 25% W%D on the y-axis are shown for guidance for the recommended criteria.

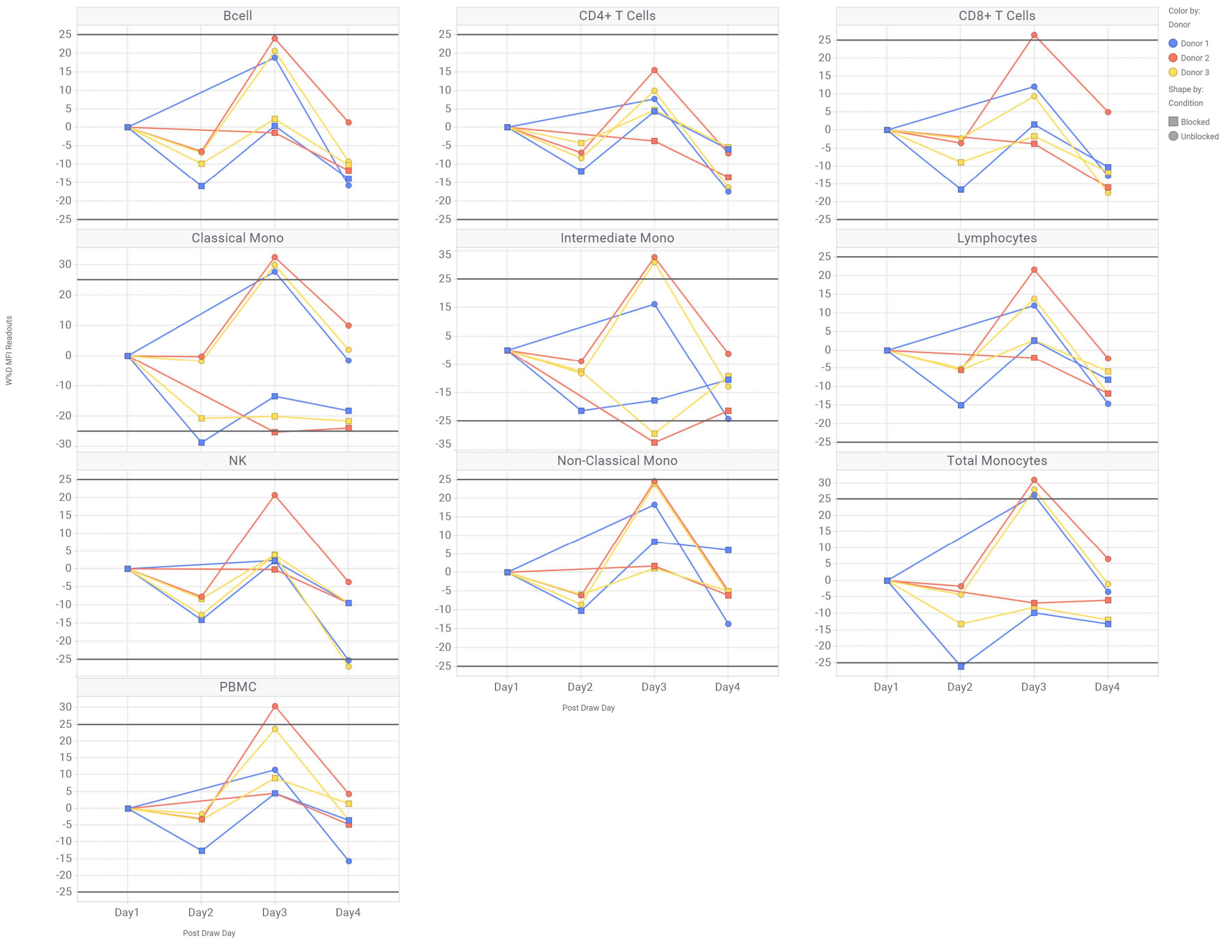

Figure 10. Post-Draw Stability. Readouts subset stability at Day 1, 2, 3 and 4. The percent change based on MFI for each readouts from Day 1 was calculated. W%D MFI Readouts is plotted versus Days Post-Draw. Each graph represents a unique cell subset. Colors represents each donor for block and unblock condition. Reference lines 25% W%D on the y-axis are shown for guidance for the recommended criteria.

Table 16. Post-Draw Stability for Major Cell Subsets

Population	Parent Population	W%D ChgD1	Average	Donor	Post-Draw Day	Condition	
Bcell[1]	CD3neg	0.00	2.49	Donor 1	Day1	Blocked	Qualified
CD4+ T Cells[1]	T Cells	0.00	26.64	Donor 1	Day1	Blocked	Qualified
CD8+ T Cells[1]	T Cells	0.00	27.86	Donor 1	Day1	Blocked	Qualified
Classical Mono[1]	Monocytes	0.00	12.25	Donor 1	Day1	Blocked	Qualified

Intermediate Mono[1]	Monocytes	0.00	0.33	Donor 1	Day1	Blocked	Qualified
Lymphocytes[1]	CD14neg	0.00	63.75	Donor 1	Day1	Blocked	Qualified
Monocytes[1]	CD19-	0.00	15.19	Donor 1	Day1	Blocked	Qualified
NK[1]	CD14neg	0.00	21.43	Donor 1	Day1	Blocked	Qualified
Non-Classical Mono[1]	Monocytes	0.00	2.53	Donor 1	Day1	Blocked	Qualified
PBMC[2]	Non-Neutrophils	0.00	30.66	Donor 1	Day1	Blocked	Qualified
Bcell[1]	CD3neg	0.31	2.50	Donor 1	Day2	Blocked	Qualified
CD4+ T Cells[1]	T Cells	-8.27	24.43	Donor 1	Day2	Blocked	Qualified
CD8+ T Cells[1]	T Cells	-1.74	27.37	Donor 1	Day2	Blocked	Qualified
Classical Mono[1]	Monocytes	9.69	13.44	Donor 1	Day2	Blocked	Qualified
Intermediate Mono[1]	Monocytes	80.12	0.60	Donor 1	Day2	Blocked	Non-Qualifed
Lymphocytes[1]	CD14neg	1.80	64.90	Donor 1	Day2	Blocked	Qualified
Monocytes[1]	CD19-	11.03	16.86	Donor 1	Day2	Blocked	Qualified
NK[1]	CD14neg	-5.62	20.23	Donor 1	Day2	Blocked	Qualified
Non-Classical Mono[1]	Monocytes	5.68	2.68	Donor 1	Day2	Blocked	Qualified
PBMC[2]	Non-Neutrophils	-4.68	29.22	Donor 1	Day2	Blocked	Qualified
Bcell[1]	CD3neg	-10.82	2.22	Donor 1	Day3	Blocked	Qualified
CD4+ T Cells[1]	T Cells	-12.76	23.24	Donor 1	Day3	Blocked	Qualified
CD8+ T Cells[1]	T Cells	-4.24	26.68	Donor 1	Day3	Blocked	Qualified
Classical Mono[1]	Monocytes	32.87	16.28	Donor 1	Day3	Blocked	Non-Qualifed
Intermediate Mono[1]	Monocytes	88.20	0.62	Donor 1	Day3	Blocked	Non-Qualifed
Lymphocytes[1]	CD14neg	3.21	65.79	Donor 1	Day3	Blocked	Qualified

Monocytes[1]	CD19-	28.01	19.44	Donor 1	Day3	Blocked	Non-Qualifed
NK[1]	CD14neg	-9.65	19.36	Donor 1	Day3	Blocked	Qualified
Non-Classical Mono[1]	Monocytes	-4.26	2.42	Donor 1	Day3	Blocked	Qualified
PBMC[2]	Non-Neutrophils	-3.57	29.56	Donor 1	Day3	Blocked	Qualified
Bcell[1]	CD3neg	-28.26	1.79	Donor 1	Day4	Blocked	Non-Qualifed
CD4+ T Cells[1]	T Cells	-19.38	21.47	Donor 1	Day4	Blocked	Qualified
CD8+ T Cells[1]	T Cells	-5.58	26.30	Donor 1	Day4	Blocked	Qualified
Classical Mono[1]	Monocytes	44.83	17.74	Donor 1	Day4	Blocked	Non-Qualifed
Intermediate Mono[1]	Monocytes	221.46	1.07	Donor 1	Day4	Blocked	Non-Qualifed
Lymphocytes[1]	CD14neg	6.03	67.59	Donor 1	Day4	Blocked	Qualified
Monocytes[1]	CD19-	42.12	21.58	Donor 1	Day4	Blocked	Non-Qualifed
NK[1]	CD14neg	-17.54	17.67	Donor 1	Day4	Blocked	Qualified
Non-Classical Mono[1]	Monocytes	2.58	2.60	Donor 1	Day4	Blocked	Qualified
PBMC[2]	Non-Neutrophils	-10.43	27.46	Donor 1	Day4	Blocked	Qualified
Bcell[1]	CD3neg	0.00	8.31	Donor 2	Day1	Blocked	Qualified
CD4+ T Cells[1]	T Cells	0.00	18.81	Donor 2	Day1	Blocked	Qualified
CD8+ T Cells[1]	T Cells	0.00	4.63	Donor 2	Day1	Blocked	Qualified
Classical Mono[1]	Monocytes	0.00	18.79	Donor 2	Day1	Blocked	Qualified
Intermediate Mono[1]	Monocytes	0.00	0.86	Donor 2	Day1	Blocked	Qualified
Lymphocytes[1]	CD14neg	0.00	38.20	Donor 2	Day1	Blocked	Qualified

Monocytes[1]	CD19-	0.00	32.40	Donor 2	Day1	Blocked	Qualified
NK[1]	CD14neg	0.00	28.03	Donor 2	Day1	Blocked	Qualified
Non-Classical Mono[1]	Monocytes	0.00	12.64	Donor 2	Day1	Blocked	Qualified
PBMC[2]	Non-Neutrophils	0.00	21.55	Donor 2	Day1	Blocked	Qualified
Bcell[1]	CD3neg	-11.03	7.40	Donor 2	Day2	Blocked	Qualified
CD4+ T Cells[1]	T Cells	-8.29	17.25	Donor 2	Day2	Blocked	Qualified
CD8+ T Cells[1]	T Cells	-1.57	4.55	Donor 2	Day2	Blocked	Qualified
Classical Mono[1]	Monocytes	18.00	22.18	Donor 2	Day2	Blocked	Qualified
Intermediate Mono[1]	Monocytes	80.65	1.56	Donor 2	Day2	Blocked	Non-Qualifed
Lymphocytes[1]	CD14neg	-3.61	36.82	Donor 2	Day2	Blocked	Qualified
Monocytes[1]	CD19-	12.03	36.30	Donor 2	Day2	Blocked	Qualified
NK[1]	CD14neg	-9.28	25.43	Donor 2	Day2	Blocked	Qualified
Non-Classical Mono[1]	Monocytes	-2.05	12.38	Donor 2	Day2	Blocked	Qualified
PBMC[2]	Non-Neutrophils	-5.15	20.44	Donor 2	Day2	Blocked	Qualified
Bcell[1]	CD3neg	-9.42	7.53	Donor 2	Day3	Blocked	Qualified
CD4+ T Cells[1]	T Cells	-19.73	15.10	Donor 2	Day3	Blocked	Qualified
CD8+ T Cells[1]	T Cells	-20.57	3.68	Donor 2	Day3	Blocked	Qualified
Classical Mono[1]	Monocytes	32.94	24.98	Donor 2	Day3	Blocked	Non-Qualifed
Intermediate Mono[1]	Monocytes	59.28	1.37	Donor 2	Day3	Blocked	Non-Qualifed
Lymphocytes[1]	CD14neg	-11.14	33.94	Donor 2	Day3	Blocked	Qualified
Monocytes[1]	CD19-	14.34	37.05	Donor 2	Day3	Blocked	Qualified
NK[1]	CD14neg	0.67	28.21	Donor 2	Day3	Blocked	Qualified

Non-Classical Mono[1]	Monocytes	-16.74	10.53	Donor 2	Day3	Blocked	Qualified
PBMC[2]	Non-Neutrophils	35.94	29.30	Donor 2	Day3	Blocked	Non-Qualifed
Bcell[1]	CD3neg	-4.42	7.94	Donor 2	Day4	Blocked	Qualified
CD4+ T Cells[1]	T Cells	-28.87	13.38	Donor 2	Day4	Blocked	Non-Qualifed
CD8+ T Cells[1]	T Cells	-22.36	3.59	Donor 2	Day4	Blocked	Qualified
Classical Mono[1]	Monocytes	30.45	24.52	Donor 2	Day4	Blocked	Non-Qualifed
Intermediate Mono[1]	Monocytes	28.86	1.11	Donor 2	Day4	Blocked	Non-Qualifed
Lymphocytes[1]	CD14neg	-12.62	33.38	Donor 2	Day4	Blocked	Qualified
Monocytes[1]	CD19-	10.71	35.87	Donor 2	Day4	Blocked	Qualified
NK[1]	CD14neg	5.16	29.47	Donor 2	Day4	Blocked	Qualified
Non-Classical Mono[1]	Monocytes	-21.18	9.96	Donor 2	Day4	Blocked	Qualified
PBMC[2]	Non-Neutrophils	5.91	22.83	Donor 2	Day4	Blocked	Qualified
Bcell[1]	CD3neg	0.00	6.33	Donor 3	Day1	Blocked	Qualified
CD4+ T Cells[1]	T Cells	0.00	33.71	Donor 3	Day1	Blocked	Qualified
CD8+ T Cells[1]	T Cells	0.00	13.30	Donor 3	Day1	Blocked	Qualified
Classical Mono[1]	Monocytes	0.00	14.10	Donor 3	Day1	Blocked	Qualified
Intermediate Mono[1]	Monocytes	0.00	0.68	Donor 3	Day1	Blocked	Qualified
Lymphocytes[1]	CD14neg	0.00	62.15	Donor 3	Day1	Blocked	Qualified
Monocytes[1]	CD19-	0.00	23.09	Donor 3	Day1	Blocked	Qualified
NK[1]	CD14neg	0.00	13.04	Donor 3	Day1	Blocked	Qualified
Non-Classical Mono[1]	Monocytes	0.00	8.10	Donor 3	Day1	Blocked	Qualified

PBMC[2]	Non-Neutrophils	0.00	21.42	Donor 3	Day1	Blocked	Qualified
Bcell[1]	CD3neg	-8.30	5.81	Donor 3	Day2	Blocked	Qualified
CD4+ T Cells[1]	T Cells	-9.77	30.42	Donor 3	Day2	Blocked	Qualified
CD8+ T Cells[1]	T Cells	-10.95	11.85	Donor 3	Day2	Blocked	Qualified
Classical Mono[1]	Monocytes	15.96	16.36	Donor 3	Day2	Blocked	Qualified
Intermediate Mono[1]	Monocytes	41.88	0.97	Donor 3	Day2	Blocked	Non-Qualifed
Lymphocytes[1]	CD14neg	-4.58	59.30	Donor 3	Day2	Blocked	Qualified
Monocytes[1]	CD19-	13.20	26.13	Donor 3	Day2	Blocked	Qualified
NK[1]	CD14neg	7.69	14.05	Donor 3	Day2	Blocked	Qualified
Non-Classical Mono[1]	Monocytes	5.82	8.57	Donor 3	Day2	Blocked	Qualified
PBMC[2]	Non-Neutrophils	-4.59	20.44	Donor 3	Day2	Blocked	Qualified
Bcell[1]	CD3neg	-5.48	5.99	Donor 3	Day3	Blocked	Qualified
CD4+ T Cells[1]	T Cells	-13.45	29.18	Donor 3	Day3	Blocked	Qualified
CD8+ T Cells[1]	T Cells	-18.46	10.85	Donor 3	Day3	Blocked	Qualified
Classical Mono[1]	Monocytes	51.54	21.37	Donor 3	Day3	Blocked	Non-Qualifed
Intermediate Mono[1]	Monocytes	94.29	1.33	Donor 3	Day3	Blocked	Non-Qualifed
Lymphocytes[1]	CD14neg	-8.18	57.07	Donor 3	Day3	Blocked	Qualified
Monocytes[1]	CD19-	33.80	30.89	Donor 3	Day3	Blocked	Non-Qualifed
NK[1]	CD14neg	-1.57	12.84	Donor 3	Day3	Blocked	Qualified
Non-Classical Mono[1]	Monocytes	-0.94	8.03	Donor 3	Day3	Blocked	Qualified
PBMC[2]	Non-Neutrophils	3.35	22.14	Donor 3	Day3	Blocked	Qualified

Bcell[1]	CD3neg	-1.85	6.22	Donor 3	Day4	Blocked	Qualified
CD4+ T Cells[1]	T Cells	-20.38	26.84	Donor 3	Day4	Blocked	Qualified
CD8+ T Cells[1]	T Cells	-24.67	10.02	Donor 3	Day4	Blocked	Qualified
Classical Mono[1]	Monocytes	48.26	20.91	Donor 3	Day4	Blocked	Non-Qualifed
Intermediate Mono[1]	Monocytes	60.69	1.10	Donor 3	Day4	Blocked	Non-Qualifed
Lymphocytes[1]	CD14neg	-8.47	56.89	Donor 3	Day4	Blocked	Qualified
Monocytes[1]	CD19-	36.22	31.45	Donor 3	Day4	Blocked	
NK[1]	CD14neg	-3.61	12.57	Donor 3	Day4	Blocked	Qualified
Non-Classical Mono[1]	Monocytes	11.76	9.06	Donor 3	Day4	Blocked	Qualified
PBMC[2]	Non-Neutrophils	-7.88	19.73	Donor 3	Day4	Blocked	Qualified
Bcell[1]	CD3neg	0.00	2.55	Donor 1	Day1	Unblocked	Qualified
CD4+ T Cells[1]	T Cells	0.00	26.70	Donor 1	Day1	Unblocked	Qualified
CD8+ T Cells[1]	T Cells	0.00	27.18	Donor 1	Day1	Unblocked	Qualified
Classical Mono[1]	Monocytes	0.00	12.79	Donor 1	Day1	Unblocked	Qualified
Intermediate Mono[1]	Monocytes	0.00	0.35	Donor 1	Day1	Unblocked	Qualified
Lymphocytes[1]	CD14neg	0.00	62.41	Donor 1	Day1	Unblocked	Qualified
Monocytes[1]	CD19-	0.00	15.22	Donor 1	Day1	Unblocked	Qualified
NK[1]	CD14neg	0.00	21.58	Donor 1	Day1	Unblocked	Qualified
Non-Classical Mono[1]	Monocytes	0.00	2.04	Donor 1	Day1	Unblocked	Qualified
PBMC[2]	Non-Neutrophils	0.00	30.26	Donor 1	Day1	Unblocked	Qualified
Bcell[1]	CD3neg	-8.42	2.33	Donor 1	Day2	Unblocked	Qualified
CD4+ T Cells[1]	T Cells	-5.38	25.27	Donor 1	Day2	Unblocked	Qualified
CD8+ T Cells[1]	T Cells	2.69	27.91	Donor 1	Day2	Unblocked	Qualified

Classical Mono[1]	Monocytes	4.50	13.37	Donor 1	Day2	Unblocked	Qualified
Intermediate Mono[1]	Monocytes	29.14	0.45	Donor 1	Day2	Unblocked	Non-Qualifed
Lymphocytes[1]	CD14neg	1.73	63.49	Donor 1	Day2	Unblocked	Qualified
Monocytes[1]	CD19-	3.51	15.76	Donor 1	Day2	Unblocked	Qualified
NK[1]	CD14neg	-4.78	20.55	Donor 1	Day2	Unblocked	Qualified
Non-Classical Mono[1]	Monocytes	-8.21	1.87	Donor 1	Day2	Unblocked	Qualified
PBMC[2]	Non-Neutrophils	0.91	30.54	Donor 1	Day2	Unblocked	Qualified
Bcell[1]	CD3neg	-4.17	2.44	Donor 1	Day3	Unblocked	Qualified
CD4+ T Cells[1]	T Cells	-4.78	25.42	Donor 1	Day3	Unblocked	Qualified
CD8+ T Cells[1]	T Cells	-2.71	26.45	Donor 1	Day3	Unblocked	Qualified
Classical Mono[1]	Monocytes	22.18	15.63	Donor 1	Day3	Unblocked	Qualified
Intermediate Mono[1]	Monocytes	60.55	0.56	Donor 1	Day3	Unblocked	Non-Qualifed
Lymphocytes[1]	CD14neg	2.15	63.75	Donor 1	Day3	Unblocked	Qualified
Monocytes[1]	CD19-	17.93	17.95	Donor 1	Day3	Unblocked	Qualified
NK[1]	CD14neg	-9.46	19.54	Donor 1	Day3	Unblocked	Qualified
Non-Classical Mono[1]	Monocytes	-16.00	1.72	Donor 1	Day3	Unblocked	Qualified
PBMC[2]	Non-Neutrophils	1.56	30.73	Donor 1	Day3	Unblocked	Qualified
Bcell[1]	CD3neg	-13.99	2.19	Donor 1	Day4	Unblocked	Qualified
CD4+ T Cells[1]	T Cells	-4.36	25.54	Donor 1	Day4	Unblocked	Qualified
CD8+ T Cells[1]	T Cells	-6.22	25.49	Donor 1	Day4	Unblocked	Qualified
Classical Mono[1]	Monocytes	24.09	15.87	Donor 1	Day4	Unblocked	Qualified

Intermediate Mono[1]	Monocytes	50.30	0.52	Donor 1	Day4	Unblocked	Non-Qualifed
Lymphocytes[1]	CD14neg	5.77	66.01	Donor 1	Day4	Unblocked	Qualified
Monocytes[1]	CD19-	20.87	18.40	Donor 1	Day4	Unblocked	Qualified
NK[1]	CD14neg	-16.07	18.11	Donor 1	Day4	Unblocked	Qualified
Non-Classical Mono[1]	Monocytes	-6.65	1.91	Donor 1	Day4	Unblocked	Qualified
PBMC[2]	Non-Neutrophils	-6.42	28.32	Donor 1	Day4	Unblocked	Qualified
Bcell[1]	CD3neg	0.00	8.80	Donor 2	Day1	Unblocked	Qualified
CD4+ T Cells[1]	T Cells	0.00	19.81	Donor 2	Day1	Unblocked	Qualified
CD8+ T Cells[1]	T Cells	0.00	4.73	Donor 2	Day1	Unblocked	Qualified
Classical Mono[1]	Monocytes	0.00	18.53	Donor 2	Day1	Unblocked	Qualified
Intermediate Mono[1]	Monocytes	0.00	1.38	Donor 2	Day1	Unblocked	Qualified
Lymphocytes[1]	CD14neg	0.00	39.54	Donor 2	Day1	Unblocked	Qualified
Monocytes[1]	CD19-	0.00	29.48	Donor 2	Day1	Unblocked	Qualified
NK[1]	CD14neg	0.00	29.78	Donor 2	Day1	Unblocked	Qualified
Non-Classical Mono[1]	Monocytes	0.00	9.48	Donor 2	Day1	Unblocked	Qualified
PBMC[2]	Non-Neutrophils	0.00	21.63	Donor 2	Day1	Unblocked	Qualified
Bcell[1]	CD3neg	-11.33	7.80	Donor 2	Day2	Unblocked	Qualified
CD4+ T Cells[1]	T Cells	-9.25	17.98	Donor 2	Day2	Unblocked	Qualified
CD8+ T Cells[1]	T Cells	-3.26	4.58	Donor 2	Day2	Unblocked	Qualified
Classical Mono[1]	Monocytes	16.55	21.60	Donor 2	Day2	Unblocked	Qualified
Intermediate Mono[1]	Monocytes	42.63	1.97	Donor 2	Day2	Unblocked	Non-Qualifed
Lymphocytes[1]	CD14neg	-5.34	37.43	Donor 2	Day2	Unblocked	Qualified

Monocytes[1]	CD19-	13.06	33.33	Donor 2	Day2	Unblocked	Qualified
NK[1]	CD14neg	-6.47	27.85	Donor 2	Day2	Unblocked	Qualified
Non-Classical Mono[1]	Monocytes	1.97	9.67	Donor 2	Day2	Unblocked	Qualified
PBMC[2]	Non-Neutrophils	-8.46	19.80	Donor 2	Day2	Unblocked	Qualified
Bcell[1]	CD3neg	-13.75	7.59	Donor 2	Day3	Unblocked	Qualified
CD4+ T Cells[1]	T Cells	-20.64	15.72	Donor 2	Day3	Unblocked	Qualified
CD8+ T Cells[1]	T Cells	-20.50	3.76	Donor 2	Day3	Unblocked	Qualified
Classical Mono[1]	Monocytes	37.43	25.47	Donor 2	Day3	Unblocked	Non-Qualifed
Intermediate Mono[1]	Monocytes	36.58	1.89	Donor 2	Day3	Unblocked	Non-Qualifed
Lymphocytes[1]	CD14neg	-14.91	33.64	Donor 2	Day3	Unblocked	Qualified
Monocytes[1]	CD19-	20.18	35.43	Donor 2	Day3	Unblocked	Qualified
NK[1]	CD14neg	-0.16	29.73	Donor 2	Day3	Unblocked	Qualified
Non-Classical Mono[1]	Monocytes	-15.85	7.98	Donor 2	Day3	Unblocked	Qualified
PBMC[2]	Non-Neutrophils	35.32	29.27	Donor 2	Day3	Unblocked	Non-Qualifed
Bcell[1]	CD3neg	-3.79	8.46	Donor 2	Day4	Unblocked	Qualified
CD4+ T Cells[1]	T Cells	-28.59	14.15	Donor 2	Day4	Unblocked	Non-Qualifed
CD8+ T Cells[1]	T Cells	-21.59	3.71	Donor 2	Day4	Unblocked	Qualified
Classical Mono[1]	Monocytes	30.59	24.20	Donor 2	Day4	Unblocked	Non-Qualifed
Intermediate Mono[1]	Monocytes	8.54	1.50	Donor 2	Day4	Unblocked	Qualified
Lymphocytes[1]	CD14neg	-14.42	33.84	Donor 2	Day4	Unblocked	Qualified
Monocytes[1]	CD19-	14.47	33.75	Donor 2	Day4	Unblocked	Qualified
NK[1]	CD14neg	3.27	30.75	Donor 2	Day4	Unblocked	Qualified

Non-Classical Mono[1]	Monocytes	-16.63	7.91	Donor 2	Day4	Unblocked	Qualified
PBMC[2]	Non-Neutrophils	4.81	22.67	Donor 2	Day4	Unblocked	Qualified
Bcell[1]	CD3neg	0.00	5.99	Donor 3	Day1	Unblocked	Qualified
CD4+ T Cells[1]	T Cells	0.00	33.86	Donor 3	Day1	Unblocked	Qualified
CD8+ T Cells[1]	T Cells	0.00	13.30	Donor 3	Day1	Unblocked	Qualified
Classical Mono[1]	Monocytes	0.00	15.04	Donor 3	Day1	Unblocked	Qualified
Intermediate Mono[1]	Monocytes	0.00	1.31	Donor 3	Day1	Unblocked	Qualified
Lymphocytes[1]	CD14neg	0.00	61.03	Donor 3	Day1	Unblocked	Qualified
Monocytes[1]	CD19-	0.00	23.21	Donor 3	Day1	Unblocked	Qualified
NK[1]	CD14neg	0.00	13.27	Donor 3	Day1	Unblocked	Qualified
Non-Classical Mono[1]	Monocytes	0.00	6.84	Donor 3	Day1	Unblocked	Qualified
PBMC[2]	Non-Neutrophils	0.00	21.17	Donor 3	Day1	Unblocked	Qualified
Bcell[1]	CD3neg	-10.43	5.36	Donor 3	Day2	Unblocked	Qualified
CD4+ T Cells[1]	T Cells	-10.14	30.43	Donor 3	Day2	Unblocked	Qualified
CD8+ T Cells[1]	T Cells	-10.11	11.96	Donor 3	Day2	Unblocked	Qualified
Classical Mono[1]	Monocytes	13.45	17.06	Donor 3	Day2	Unblocked	Qualified
Intermediate Mono[1]	Monocytes	15.13	1.50	Donor 3	Day2	Unblocked	Qualified
Lymphocytes[1]	CD14neg	-6.61	57.00	Donor 3	Day2	Unblocked	Qualified
Monocytes[1]	CD19-	9.85	25.50	Donor 3	Day2	Unblocked	Qualified
NK[1]	CD14neg	10.27	14.63	Donor 3	Day2	Unblocked	Qualified
Non-Classical Mono[1]	Monocytes	-0.67	6.79	Donor 3	Day2	Unblocked	Qualified

PBMC[2]	Non-Neutrophils	-3.90	20.34	Donor 3	Day2	Unblocked	Qualified
Bcell[1]	CD3neg	-7.09	5.56	Donor 3	Day3	Unblocked	Qualified
CD4+ T Cells[1]	T Cells	-12.01	29.80	Donor 3	Day3	Unblocked	Qualified
CD8+ T Cells[1]	T Cells	-18.95	10.78	Donor 3	Day3	Unblocked	Qualified
Classical Mono[1]	Monocytes	39.33	20.95	Donor 3	Day3	Unblocked	Non-Qualifed
Intermediate Mono[1]	Monocytes	44.39	1.88	Donor 3	Day3	Unblocked	Non-Qualifed
Lymphocytes[1]	CD14neg	-9.69	55.12	Donor 3	Day3	Unblocked	Qualified
Monocytes[1]	CD19-	23.44	28.65	Donor 3	Day3	Unblocked	Qualified
NK[1]	CD14neg	-0.89	13.15	Donor 3	Day3	Unblocked	Qualified
Non-Classical Mono[1]	Monocytes	-16.19	5.73	Donor 3	Day3	Unblocked	Qualified
PBMC[2]	Non-Neutrophils	-0.09	21.15	Donor 3	Day3	Unblocked	Qualified
Bcell[1]	CD3neg	1.77	6.09	Donor 3	Day4	Unblocked	Qualified
CD4+ T Cells[1]	T Cells	-21.97	26.42	Donor 3	Day4	Unblocked	Qualified
CD8+ T Cells[1]	T Cells	-28.03	9.57	Donor 3	Day4	Unblocked	Non-Qualifed
Classical Mono[1]	Monocytes	41.43	21.27	Donor 3	Day4	Unblocked	Non-Qualifed
Intermediate Mono[1]	Monocytes	11.04	1.45	Donor 3	Day4	Unblocked	Qualified
Lymphocytes[1]	CD14neg	-12.73	53.27	Donor 3	Day4	Unblocked	Qualified
Monocytes[1]	CD19-	31.38	30.50	Donor 3	Day4	Unblocked	Non-Qualifed
NK[1]	CD14neg	0.71	13.36	Donor 3	Day4	Unblocked	Qualified
Non-Classical Mono[1]	Monocytes	10.70	7.57	Donor 3	Day4	Unblocked	Qualified

PBMC[2]	Non-Neutrophils	-11.99	18.63	Donor 3	Day4	Unblocked	Qualified

[1]W%D was determined based on percent of PBMC
[2]W%D was determined based on percent of WBC

Table 17. Post-Draw Stability for Readouts Subsets

Population	Parent Population	W%D ChgD1	Average	Donor	Post-Draw Day	Condition	
Bcell[1]	CD3neg	0.00	196.52	Donor 1	Day1	Blocked	Qualified
CD4+ T Cells[1]	T Cells	0.00	166.54	Donor 1	Day1	Blocked	Qualified
CD8+ T Cells[1]	T Cells	0.00	204.52	Donor 1	Day1	Blocked	Qualified
Classical Mono[1]	Monocytes	0.00	734.39	Donor 1	Day1	Blocked	Qualified
Intermediate Mono[1]	Monocytes	0.00	1078.98	Donor 1	Day1	Blocked	Qualified
Lymphocytes[1]	CD14neg	0.00	188.81	Donor 1	Day1	Blocked	Qualified
Monocytes[1]	CD19-	0.00	666.76	Donor 1	Day1	Blocked	Qualified
NK[1]	CD14neg	0.00	201.77	Donor 1	Day1	Blocked	Qualified
Non-Classical Mono[1]	Monocytes	0.00	332.03	Donor 1	Day1	Blocked	Qualified
PBMC[1]	Non-Neutrophils	0.00	213.85	Donor 1	Day1	Blocked	Qualified
Bcell[1]	CD3neg	-15.81	165.46	Donor 1	Day2	Blocked	Qualified
CD4+ T Cells[1]	T Cells	-11.85	146.81	Donor 1	Day2	Blocked	Qualified
CD8+ T Cells[1]	T Cells	-16.49	170.79	Donor 1	Day2	Blocked	Qualified
Classical Mono[1]	Monocytes	-28.66	523.91	Donor 1	Day2	Blocked	Non-Qualifed
Intermediate Mono[1]	Monocytes	-21.18	850.48	Donor 1	Day2	Blocked	Qualified
Lymphocytes[1]	CD14neg	-14.92	160.65	Donor 1	Day2	Blocked	Qualified
Monocytes[1]	CD19-	-26.11	492.66	Donor 1	Day2	Blocked	Non-Qualifed

NK[1]	CD14neg	-14.08	173.36	Donor 1	Day2	Blocked	Qualified
Non-Classical Mono[1]	Monocytes	-10.12	298.44	Donor 1	Day2	Blocked	Qualified
PBMC[1]	Non-Neutrophils	-12.71	186.67	Donor 1	Day2	Blocked	Qualified
Bcell[1]	CD3neg	0.43	197.35	Donor 1	Day3	Blocked	Qualified
CD4+ T Cells[1]	T Cells	4.28	173.67	Donor 1	Day3	Blocked	Qualified
CD8+ T Cells[1]	T Cells	1.60	207.80	Donor 1	Day3	Blocked	Qualified
Classical Mono[1]	Monocytes	-13.33	636.49	Donor 1	Day3	Blocked	Qualified
Intermediate Mono[1]	Monocytes	-17.64	888.69	Donor 1	Day3	Blocked	Qualified
Lymphocytes[1]	CD14neg	2.44	193.43	Donor 1	Day3	Blocked	Qualified
Monocytes[1]	CD19-	-9.81	601.33	Donor 1	Day3	Blocked	Qualified
NK[1]	CD14neg	2.16	206.13	Donor 1	Day3	Blocked	Qualified
Non-Classical Mono[1]	Monocytes	8.30	359.58	Donor 1	Day3	Blocked	Qualified
PBMC[1]	Non-Neutrophils	4.58	223.65	Donor 1	Day3	Blocked	Qualified
Bcell[1]	CD3neg	-13.84	169.32	Donor 1	Day4	Blocked	Qualified
CD4+ T Cells[1]	T Cells	-5.79	156.89	Donor 1	Day4	Blocked	Qualified
CD8+ T Cells[1]	T Cells	-10.22	183.63	Donor 1	Day4	Blocked	Qualified
Classical Mono[1]	Monocytes	-18.26	600.29	Donor 1	Day4	Blocked	Qualified
Intermediate Mono[1]	Monocytes	-10.35	967.32	Donor 1	Day4	Blocked	Qualified
Lymphocytes[1]	CD14neg	-8.13	173.46	Donor 1	Day4	Blocked	Qualified
Monocytes[1]	CD19-	-13.07	579.64	Donor 1	Day4	Blocked	Qualified
NK[1]	CD14neg	-9.39	182.83	Donor 1	Day4	Blocked	Qualified
Non-Classical Mono[1]	Monocytes	6.04	352.08	Donor 1	Day4	Blocked	Qualified

PBMC[1]	Non-Neutrophils	-3.54	206.29	Donor 1	Day4	Blocked	Qualified
Bcell[1]	CD3neg	0.00	170.68	Donor 2	Day1	Blocked	Qualified
CD4+ T Cells[1]	T Cells	0.00	183.96	Donor 2	Day1	Blocked	Qualified
CD8+ T Cells[1]	T Cells	0.00	210.47	Donor 2	Day1	Blocked	Qualified
Classical Mono[1]	Monocytes	0.00	914.81	Donor 2	Day1	Blocked	Qualified
Intermediate Mono[1]	Monocytes	0.00	1300.57	Donor 2	Day1	Blocked	Qualified
Lymphocytes[1]	CD14neg	0.00	189.55	Donor 2	Day1	Blocked	Qualified
Monocytes[1]	CD19-	0.00	639.72	Donor 2	Day1	Blocked	Qualified
NK[1]	CD14neg	0.00	234.83	Donor 2	Day1	Blocked	Qualified
Non-Classical Mono[1]	Monocytes	0.00	327.31	Donor 2	Day1	Blocked	Qualified
PBMC[1]	Non-Neutrophils	0.00	261.07	Donor 2	Day1	Blocked	Qualified
Bcell[1]	CD3neg	-12.58	149.22	Donor 2	Day2	Blocked	Qualified
CD4+ T Cells[1]	T Cells	-8.79	167.78	Donor 2	Day2	Blocked	Qualified
CD8+ T Cells[1]	T Cells	-11.52	186.23	Donor 2	Day2	Blocked	Qualified
Classical Mono[1]	Monocytes	-19.45	736.84	Donor 2	Day2	Blocked	Qualified
Intermediate Mono[1]	Monocytes	-13.34	1127.07	Donor 2	Day2	Blocked	Qualified
Lymphocytes[1]	CD14neg	-9.80	170.99	Donor 2	Day2	Blocked	Qualified
Monocytes[1]	CD19-	-8.48	585.49	Donor 2	Day2	Blocked	Qualified
NK[1]	CD14neg	-13.19	203.85	Donor 2	Day2	Blocked	Qualified
Non-Classical Mono[1]	Monocytes	-8.65	298.99	Donor 2	Day2	Blocked	Qualified
PBMC[1]	Non-Neutrophils	-7.00	242.79	Donor 2	Day2	Blocked	Qualified
Bcell[1]	CD3neg	-1.44	168.23	Donor 2	Day3	Blocked	Qualified

CD4+ T Cells[1]	T Cells	-3.59	177.36	Donor 2	Day3	Blocked	Qualified
CD8+ T Cells[1]	T Cells	-3.83	202.41	Donor 2	Day3	Blocked	Qualified
Classical Mono[1]	Monocytes	-25.14	684.81	Donor 2	Day3	Blocked	Non-Qualifed
Intermediate Mono[1]	Monocytes	-32.41	879.04	Donor 2	Day3	Blocked	Non-Qualifed
Lymphocytes[1]	CD14neg	-2.05	185.67	Donor 2	Day3	Blocked	Qualified
Monocytes[1]	CD19-	-6.83	596.03	Donor 2	Day3	Blocked	Qualified
NK[1]	CD14neg	-0.01	234.81	Donor 2	Day3	Blocked	Qualified
Non-Classical Mono[1]	Monocytes	1.85	333.35	Donor 2	Day3	Blocked	Qualified
PBMC[1]	Non-Neutrophils	4.48	272.75	Donor 2	Day3	Blocked	Qualified
Bcell[1]	CD3neg	-11.55	150.98	Donor 2	Day4	Blocked	Qualified
CD4+ T Cells[1]	T Cells	-13.55	159.03	Donor 2	Day4	Blocked	Qualified
CD8+ T Cells[1]	T Cells	-15.97	176.85	Donor 2	Day4	Blocked	Qualified
Classical Mono[1]	Monocytes	-23.94	695.85	Donor 2	Day4	Blocked	Qualified
Intermediate Mono[1]	Monocytes	-21.22	1024.63	Donor 2	Day4	Blocked	Qualified
Lymphocytes[1]	CD14neg	-11.73	167.32	Donor 2	Day4	Blocked	Qualified
Monocytes[1]	CD19-	-6.02	601.19	Donor 2	Day4	Blocked	Qualified
NK[1]	CD14neg	-9.44	212.66	Donor 2	Day4	Blocked	Qualified
Non-Classical Mono[1]	Monocytes	-6.12	307.28	Donor 2	Day4	Blocked	Qualified
PBMC[1]	Non-Neutrophils	-4.87	248.35	Donor 2	Day4	Blocked	Qualified
Bcell[1]	CD3neg	0.00	158.98	Donor 3	Day1	Blocked	Qualified
CD4+ T Cells[1]	T Cells	0.00	156.49	Donor 3	Day1	Blocked	Qualified
CD8+ T Cells[1]	T Cells	0.00	174.86	Donor 3	Day1	Blocked	Qualified

Classical Mono[1]	Monocytes	0.00	626.33	Donor 3	Day1	Blocked	Qualified
Intermediate Mono[1]	Monocytes	0.00	812.38	Donor 3	Day1	Blocked	Qualified
Lymphocytes[1]	CD14neg	0.00	169.80	Donor 3	Day1	Blocked	Qualified
Monocytes[1]	CD19-	0.00	498.36	Donor 3	Day1	Blocked	Qualified
NK[1]	CD14neg	0.00	218.64	Donor 3	Day1	Blocked	Qualified
Non-Classical Mono[1]	Monocytes	0.00	343.18	Donor 3	Day1	Blocked	Qualified
PBMC[1]	Non-Neutrophils	0.00	217.63	Donor 3	Day1	Blocked	Qualified
Bcell[1]	CD3neg	-9.80	143.40	Donor 3	Day2	Blocked	Qualified
CD4+ T Cells[1]	T Cells	-4.13	150.02	Donor 3	Day2	Blocked	Qualified
CD8+ T Cells[1]	T Cells	-8.88	159.34	Donor 3	Day2	Blocked	Qualified
Classical Mono[1]	Monocytes	-20.75	496.37	Donor 3	Day2	Blocked	Qualified
Intermediate Mono[1]	Monocytes	-7.36	752.61	Donor 3	Day2	Blocked	Qualified
Lymphocytes[1]	CD14neg	-5.57	160.34	Donor 3	Day2	Blocked	Qualified
Monocytes[1]	CD19-	-13.08	433.19	Donor 3	Day2	Blocked	Qualified
NK[1]	CD14neg	-8.14	200.85	Donor 3	Day2	Blocked	Qualified
Non-Classical Mono[1]	Monocytes	-5.91	322.90	Donor 3	Day2	Blocked	Qualified
PBMC[1]	Non-Neutrophils	-3.23	210.59	Donor 3	Day2	Blocked	Qualified
Bcell[1]	CD3neg	2.37	162.75	Donor 3	Day3	Blocked	Qualified
CD4+ T Cells[1]	T Cells	4.79	163.98	Donor 3	Day3	Blocked	Qualified
CD8+ T Cells[1]	T Cells	-1.56	172.13	Donor 3	Day3	Blocked	Qualified
Classical Mono[1]	Monocytes	-20.03	500.85	Donor 3	Day3	Blocked	Qualified

Intermediate Mono[1]	Monocytes	-29.22	575.00	Donor 3	Day3	Blocked	Non-Qualifed
Lymphocytes[1]	CD14neg	2.73	174.43	Donor 3	Day3	Blocked	Qualified
Monocytes[1]	CD19-	-7.98	458.61	Donor 3	Day3	Blocked	Qualified
NK[1]	CD14neg	4.01	227.40	Donor 3	Day3	Blocked	Qualified
Non-Classical Mono[1]	Monocytes	1.28	347.56	Donor 3	Day3	Blocked	Qualified
PBMC[1]	Non-Neutrophils	9.07	237.38	Donor 3	Day3	Blocked	Qualified
Bcell[1]	CD3neg	-10.04	143.01	Donor 3	Day4	Blocked	Qualified
CD4+ T Cells[1]	T Cells	-5.36	148.10	Donor 3	Day4	Blocked	Qualified
CD8+ T Cells[1]	T Cells	-11.63	154.54	Donor 3	Day4	Blocked	Qualified
Classical Mono[1]	Monocytes	-21.69	490.45	Donor 3	Day4	Blocked	Qualified
Intermediate Mono[1]	Monocytes	-9.07	738.73	Donor 3	Day4	Blocked	Qualified
Lymphocytes[1]	CD14neg	-5.91	159.77	Donor 3	Day4	Blocked	Qualified
Monocytes[1]	CD19-	-11.86	439.25	Donor 3	Day4	Blocked	Qualified
NK[1]	CD14neg	-9.32	198.25	Donor 3	Day4	Blocked	Qualified
Non-Classical Mono[1]	Monocytes	-4.86	326.48	Donor 3	Day4	Blocked	Qualified
PBMC[1]	Non-Neutrophils	1.48	220.86	Donor 3	Day4	Blocked	Qualified
Bcell[1]	CD3neg	0.00	1171.35	Donor 1	Day1	Unblocked	Qualified
CD4+ T Cells[1]	T Cells	0.00	975.76	Donor 1	Day1	Unblocked	Qualified
CD8+ T Cells[1]	T Cells	0.00	1457.74	Donor 1	Day1	Unblocked	Qualified
Classical Mono[1]	Monocytes	0.00	2149.46	Donor 1	Day1	Unblocked	Qualified
Intermediate Mono[1]	Monocytes	0.00	2277.94	Donor 1	Day1	Unblocked	Qualified
Lymphocytes[1]	CD14neg	0.00	1235.52	Donor 1	Day1	Unblocked	Qualified

Monocytes[1]	CD19-	0.00	2175.44	Donor 1	Day1	Unblocked	Qualified
NK[1]	CD14neg	0.00	1783.34	Donor 1	Day1	Unblocked	Qualified
Non-Classical Mono[1]	Monocytes	0.00	2395.12	Donor 1	Day1	Unblocked	Qualified
PBMC[1]	Non-Neutrophils	0.00	1486.95	Donor 1	Day1	Unblocked	Qualified
Bcell[1]	CD3neg	-5.46	1107.40	Donor 1	Day2	Unblocked	Qualified
CD4+ T Cells[1]	T Cells	-6.80	909.41	Donor 1	Day2	Unblocked	Qualified
CD8+ T Cells[1]	T Cells	-8.12	1339.30	Donor 1	Day2	Unblocked	Qualified
Classical Mono[1]	Monocytes	-6.54	2008.99	Donor 1	Day2	Unblocked	Qualified
Intermediate Mono[1]	Monocytes	-26.86	1666.00	Donor 1	Day2	Unblocked	Non-Qualifed
Lymphocytes[1]	CD14neg	-6.11	1160.09	Donor 1	Day2	Unblocked	Qualified
Monocytes[1]	CD19-	-7.17	2019.53	Donor 1	Day2	Unblocked	Qualified
NK[1]	CD14neg	-11.18	1583.90	Donor 1	Day2	Unblocked	Qualified
Non-Classical Mono[1]	Monocytes	-9.10	2177.23	Donor 1	Day2	Unblocked	Qualified
PBMC[1]	Non-Neutrophils	-8.02	1367.72	Donor 1	Day2	Unblocked	Qualified
Bcell[1]	CD3neg	18.78	1391.38	Donor 1	Day3	Unblocked	Qualified
CD4+ T Cells[1]	T Cells	7.78	1051.65	Donor 1	Day3	Unblocked	Qualified
CD8+ T Cells[1]	T Cells	12.12	1634.45	Donor 1	Day3	Unblocked	Qualified
Classical Mono[1]	Monocytes	27.81	2747.13	Donor 1	Day3	Unblocked	Non-Qualifed
Intermediate Mono[1]	Monocytes	16.29	2649.01	Donor 1	Day3	Unblocked	Qualified
Lymphocytes[1]	CD14neg	11.95	1383.16	Donor 1	Day3	Unblocked	Qualified
Monocytes[1]	CD19-	26.37	2749.03	Donor 1	Day3	Unblocked	
NK[1]	CD14neg	2.50	1827.95	Donor 1	Day3	Unblocked	Qualified

Non-Classical Mono[1]	Monocytes	18.34	2834.32	Donor 1	Day3	Unblocked	Qualified
PBMC[1]	Non-Neutrophils	11.52	1658.23	Donor 1	Day3	Unblocked	Qualified
Bcell[1]	CD3neg	-15.70	987.45	Donor 1	Day4	Unblocked	Qualified
CD4+ T Cells[1]	T Cells	-17.40	805.99	Donor 1	Day4	Unblocked	Qualified
CD8+ T Cells[1]	T Cells	-12.75	1271.90	Donor 1	Day4	Unblocked	Qualified
Classical Mono[1]	Monocytes	-1.64	2114.26	Donor 1	Day4	Unblocked	Qualified
Intermediate Mono[1]	Monocytes	-24.21	1726.34	Donor 1	Day4	Unblocked	Qualified
Lymphocytes[1]	CD14neg	-14.64	1054.69	Donor 1	Day4	Unblocked	Qualified
Monocytes[1]	CD19-	-3.48	2099.66	Donor 1	Day4	Unblocked	Qualified
NK[1]	CD14neg	-25.14	1335.02	Donor 1	Day4	Unblocked	Non-Qualifed
Non-Classical Mono[1]	Monocytes	-13.70	2066.97	Donor 1	Day4	Unblocked	Qualified
PBMC[1]	Non-Neutrophils	-15.63	1254.56	Donor 1	Day4	Unblocked	Qualified
Bcell[1]	CD3neg	0.00	752.20	Donor 2	Day1	Unblocked	Qualified
CD4+ T Cells[1]	T Cells	0.00	760.96	Donor 2	Day1	Unblocked	Qualified
CD8+ T Cells[1]	T Cells	0.00	901.59	Donor 2	Day1	Unblocked	Qualified
Classical Mono[1]	Monocytes	0.00	1816.00	Donor 2	Day1	Unblocked	Qualified
Intermediate Mono[1]	Monocytes	0.00	1846.36	Donor 2	Day1	Unblocked	Qualified
Lymphocytes[1]	CD14neg	0.00	808.59	Donor 2	Day1	Unblocked	Qualified
Monocytes[1]	CD19-	0.00	1811.29	Donor 2	Day1	Unblocked	Qualified
NK[1]	CD14neg	0.00	1544.27	Donor 2	Day1	Unblocked	Qualified
Non-Classical Mono[1]	Monocytes	0.00	1798.69	Donor 2	Day1	Unblocked	Qualified

PBMC[1]	Non-Neutrophils	0.00	1345.69	Donor 2	Day1	Unblocked	Qualified
Bcell[1]	CD3neg	-6.34	704.51	Donor 2	Day2	Unblocked	Qualified
CD4+ T Cells[1]	T Cells	-6.81	709.15	Donor 2	Day2	Unblocked	Qualified
CD8+ T Cells[1]	T Cells	-3.53	869.81	Donor 2	Day2	Unblocked	Qualified
Classical Mono[1]	Monocytes	-0.19	1812.52	Donor 2	Day2	Unblocked	Qualified
Intermediate Mono[1]	Monocytes	-3.78	1776.66	Donor 2	Day2	Unblocked	Qualified
Lymphocytes[1]	CD14neg	-5.41	764.84	Donor 2	Day2	Unblocked	Qualified
Monocytes[1]	CD19-	-1.72	1780.14	Donor 2	Day2	Unblocked	Qualified
NK[1]	CD14neg	-7.56	1427.48	Donor 2	Day2	Unblocked	Qualified
Non-Classical Mono[1]	Monocytes	-6.06	1689.78	Donor 2	Day2	Unblocked	Qualified
PBMC[1]	Non-Neutrophils	-3.23	1302.19	Donor 2	Day2	Unblocked	Qualified
Bcell[1]	CD3neg	24.08	933.31	Donor 2	Day3	Unblocked	Qualified
CD4+ T Cells[1]	T Cells	15.60	879.70	Donor 2	Day3	Unblocked	Qualified
CD8+ T Cells[1]	T Cells	26.52	1140.67	Donor 2	Day3	Unblocked	Non-Qualifed
Classical Mono[1]	Monocytes	32.48	2405.79	Donor 2	Day3	Unblocked	Non-Qualifed
Intermediate Mono[1]	Monocytes	32.80	2452.00	Donor 2	Day3	Unblocked	Non-Qualifed
Lymphocytes[1]	CD14neg	21.58	983.12	Donor 2	Day3	Unblocked	Qualified
Monocytes[1]	CD19-	30.96	2372.08	Donor 2	Day3	Unblocked	
NK[1]	CD14neg	20.76	1864.81	Donor 2	Day3	Unblocked	Qualified
Non-Classical Mono[1]	Monocytes	24.63	2241.67	Donor 2	Day3	Unblocked	Qualified
PBMC[1]	Non-Neutrophils	30.50	1756.15	Donor 2	Day3	Unblocked	Non-Qualifed

Bcell[1]	CD3neg	1.43	762.99	Donor 2	Day4	Unblocked	Qualified
CD4+ T Cells[1]	T Cells	-7.04	707.36	Donor 2	Day4	Unblocked	Qualified
CD8+ T Cells[1]	T Cells	5.11	947.62	Donor 2	Day4	Unblocked	Qualified
Classical Mono[1]	Monocytes	9.87	1995.25	Donor 2	Day4	Unblocked	Qualified
Intermediate Mono[1]	Monocytes	-1.06	1826.71	Donor 2	Day4	Unblocked	Qualified
Lymphocytes[1]	CD14neg	-2.38	789.33	Donor 2	Day4	Unblocked	Qualified
Monocytes[1]	CD19-	6.66	1931.87	Donor 2	Day4	Unblocked	Qualified
NK[1]	CD14neg	-3.67	1487.55	Donor 2	Day4	Unblocked	Qualified
Non-Classical Mono[1]	Monocytes	-5.02	1708.43	Donor 2	Day4	Unblocked	Qualified
PBMC[1]	Non-Neutrophils	4.29	1403.41	Donor 2	Day4	Unblocked	Qualified
Bcell[1]	CD3neg	0.00	912.85	Donor 3	Day1	Unblocked	Qualified
CD4+ T Cells[1]	T Cells	0.00	840.86	Donor 3	Day1	Unblocked	Qualified
CD8+ T Cells[1]	T Cells	0.00	900.15	Donor 3	Day1	Unblocked	Qualified
Classical Mono[1]	Monocytes	0.00	2090.02	Donor 3	Day1	Unblocked	Qualified
Intermediate Mono[1]	Monocytes	0.00	2115.09	Donor 3	Day1	Unblocked	Qualified
Lymphocytes[1]	CD14neg	0.00	905.34	Donor 3	Day1	Unblocked	Qualified
Monocytes[1]	CD19-	0.00	2114.59	Donor 3	Day1	Unblocked	Qualified
NK[1]	CD14neg	0.00	1882.76	Donor 3	Day1	Unblocked	Qualified
Non-Classical Mono[1]	Monocytes	0.00	2158.65	Donor 3	Day1	Unblocked	Qualified
PBMC[2]	Non-Neutrophils	0.00	1225.26	Donor 3	Day1	Unblocked	Qualified
Bcell[1]	CD3neg	-6.86	850.23	Donor 3	Day2	Unblocked	Qualified
CD4+ T Cells[1]	T Cells	-8.32	770.94	Donor 3	Day2	Unblocked	Qualified
CD8+ T Cells[1]	T Cells	-2.12	881.02	Donor 3	Day2	Unblocked	Qualified

Classical Mono[1]	Monocytes	-1.81	2052.24	Donor 3	Day2	Unblocked	Qualified
Intermediate Mono[1]	Monocytes	-8.25	1940.67	Donor 3	Day2	Unblocked	Qualified
Lymphocytes[1]	CD14neg	-5.06	859.51	Donor 3	Day2	Unblocked	Qualified
Monocytes[1]	CD19-	-4.18	2026.29	Donor 3	Day2	Unblocked	Qualified
NK[1]	CD14neg	-12.71	1643.40	Donor 3	Day2	Unblocked	Qualified
Non-Classical Mono[1]	Monocytes	-8.40	1977.37	Donor 3	Day2	Unblocked	Qualified
PBMC[1]	Non-Neutrophils	-1.74	1203.91	Donor 3	Day2	Unblocked	Qualified
Bcell[1]	CD3neg	20.78	1102.53	Donor 3	Day3	Unblocked	Qualified
CD4+ T Cells[1]	T Cells	9.96	924.62	Donor 3	Day3	Unblocked	Qualified
CD8+ T Cells[1]	T Cells	9.54	985.99	Donor 3	Day3	Unblocked	Qualified
Classical Mono[1]	Monocytes	30.03	2717.70	Donor 3	Day3	Unblocked	Non-Qualifed
Intermediate Mono[1]	Monocytes	31.06	2772.08	Donor 3	Day3	Unblocked	Non-Qualifed
Lymphocytes[1]	CD14neg	13.86	1030.85	Donor 3	Day3	Unblocked	Qualified
Monocytes[1]	CD19-	27.93	2705.17	Donor 3	Day3	Unblocked	Non-Qualifed
NK[1]	CD14neg	3.90	1956.20	Donor 3	Day3	Unblocked	Qualified
Non-Classical Mono[1]	Monocytes	23.81	2672.58	Donor 3	Day3	Unblocked	Qualified
PBMC[1]	Non-Neutrophils	23.66	1515.21	Donor 3	Day3	Unblocked	Qualified
Bcell[1]	CD3neg	-9.28	828.10	Donor 3	Day4	Unblocked	Qualified
CD4+ T Cells[1]	T Cells	-16.35	703.38	Donor 3	Day4	Unblocked	Qualified
CD8+ T Cells[1]	T Cells	-17.62	741.53	Donor 3	Day4	Unblocked	Qualified
Classical Mono[1]	Monocytes	1.94	2130.47	Donor 3	Day4	Unblocked	Qualified

Intermediate Mono[1]	Monocytes	-12.79	1844.67	Donor 3	Day4	Unblocked	Qualified
Lymphocytes[1]	CD14neg	-11.78	798.74	Donor 3	Day4	Unblocked	Qualified
Monocytes[1]	CD19-	-0.99	2093.69	Donor 3	Day4	Unblocked	Qualified
NK[1]	CD14neg	-26.94	1375.56	Donor 3	Day4	Unblocked	Non-Qualifed
Non-Classical Mono[1]	Monocytes	-5.76	2034.32	Donor 3	Day4	Unblocked	Qualified
PBMC[1]	Non-Neutrophils	-3.55	1181.71	Donor 3	Day4	Unblocked	Qualified

[1]W%D was determined based on MFI

7.1.5. POST-PROCESSING STABILITY

For Post-Processing Stability, triplicate samples from three donors were lyse/fixed by one operator and storage at -80 °C for 1, 9, 30, or 60 days, then stained for block and unblock condition and adquired on the primary instrument. The average of the triplicates was calculated for each run day. The average at Day 9, Day 30 and Day 60 was compared to the average at Day 1 to determine the percent change from Day 1 (Figure 10 and 11 and tables 18 and 19)

Overall, the results suggest that running samples between 1 to 30 days Post-Freeze is an acceptable window for data acquisition.

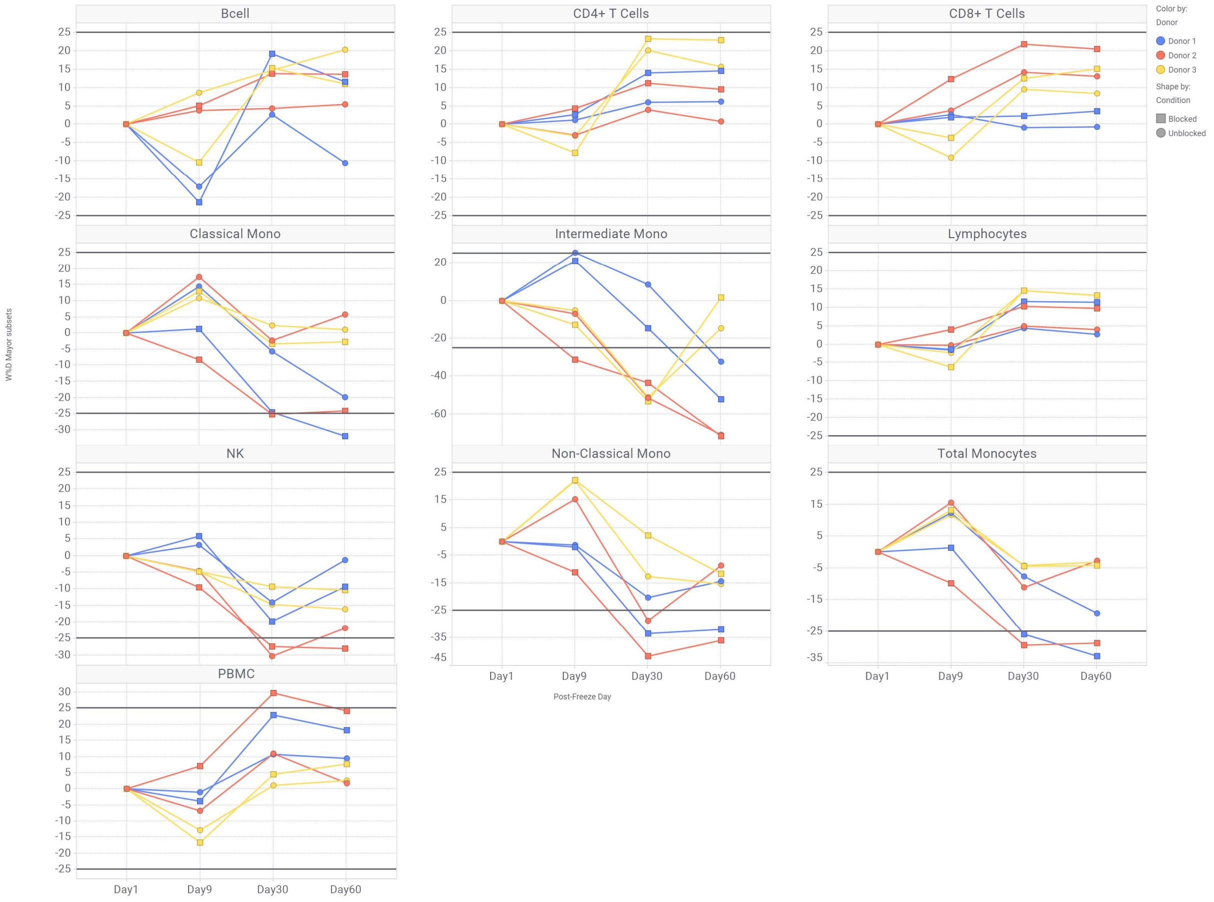

Figure 11. Post-Processing Stability. Major cell subset stability at Day 1, 9, 30 and 60. The percent change in the percent of PBMC/WBC from Day 1 was calculated. W%D Mayor subsets is plotted versus Days Post-Freeze. Each graph represents a unique cell subset. Colors represents each donor for block and unblock condition. Reference lines ±25% W%D on the y-axis are shown for guidance for the recommended criteria.

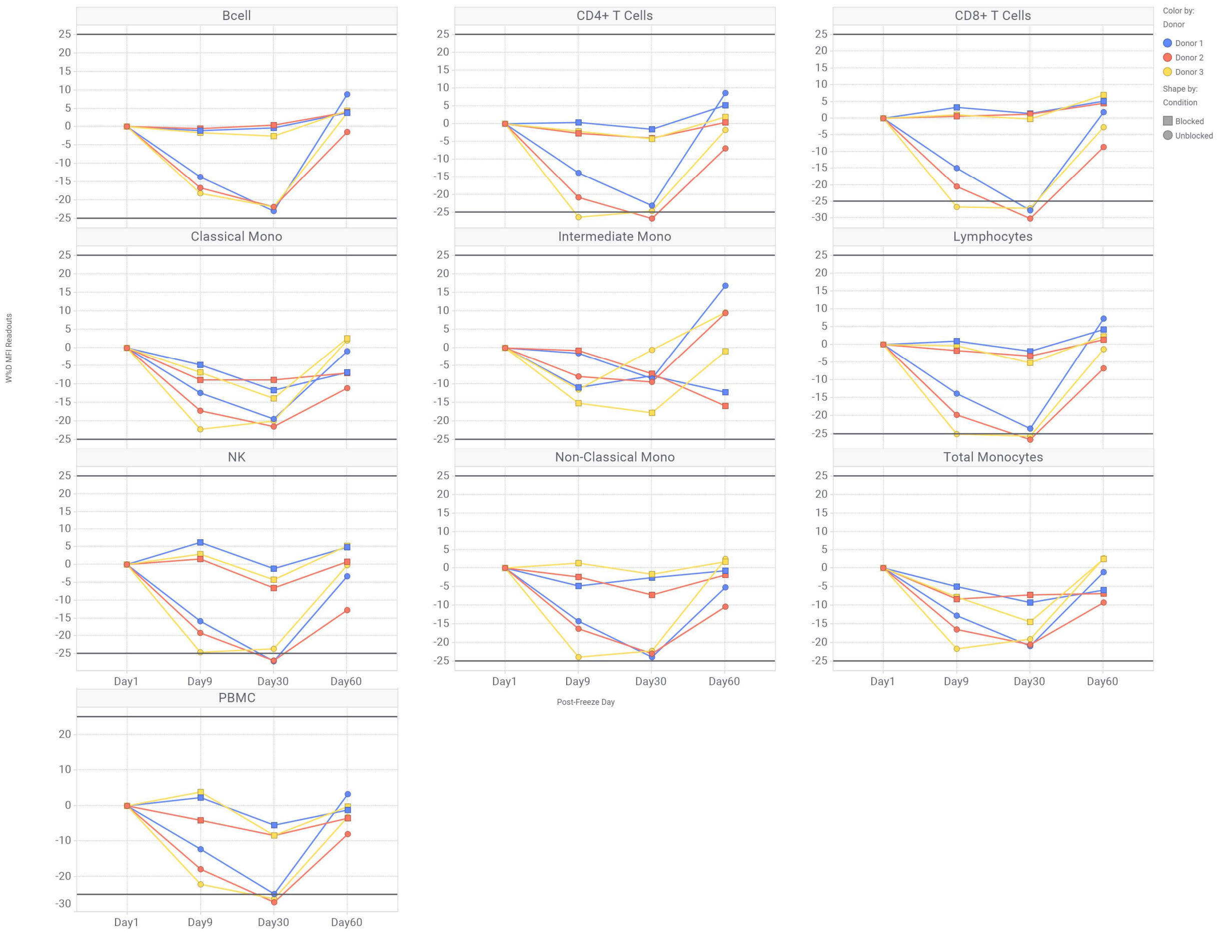

Figure 12. Post-Processing Stability. Readouts subset stability at Day 1, 9, 30 and 60. The percent change based on MFI for each readouts from Day 1 was calculated. W%D MFI Readouts is plotted versus Days Post-Freeeze. Each graph represents a unique cell subset. Colors represents each donor for block and unblock condition. Reference lines ±25% W%D on the y-axis are shown for guidance for the recommended criteria.

Table 18. Post-Processing Stability for Major Cell Subsets

Population	Parent Population	W%D ChgD1	Average	Donor	Post-Processing Day	Condition	
Bcell[1]	CD3neg	0.00	9.07	Donor 1	Day1	Blocked	Qualified
CD4+ T Cells[1]	T Cells	0.00	43.23	Donor 1	Day1	Blocked	Qualified
CD8+ T Cells[1]	T Cells	0.00	8.22	Donor 1	Day1	Blocked	Qualified
Classical Mono[1]	Monocytes	0.00	12.49	Donor 1	Day1	Blocked	Qualified
Intermediate Mono[1]	Monocytes	0.00	0.78	Donor 1	Day1	Blocked	Qualified
Lymphocytes[1]	CD14neg	0.00	65.45	Donor 1	Day1	Blocked	Qualified
Monocytes[1]	CD19-	0.00	17.54	Donor 1	Day1	Blocked	Qualified
NK[1]	CD14neg	0.00	11.98	Donor 1	Day1	Blocked	Qualified
Non-Classical Mono[1]	Monocytes	0.00	4.27	Donor 1	Day1	Blocked	Qualified
PBMC[2]	Non-Neutrophils	0.00	22.28	Donor 1	Day1	Blocked	Qualified
Bcell[1]	CD3neg	-21.32	7.14	Donor 1	Day9	Blocked	Qualified
CD4+ T Cells[1]	T Cells	2.68	44.39	Donor 1	Day9	Blocked	Qualified
CD8+ T Cells[1]	T Cells	1.98	8.38	Donor 1	Day9	Blocked	Qualified
Classical Mono[1]	Monocytes	1.22	12.64	Donor 1	Day9	Blocked	Qualified
Intermediate Mono[1]	Monocytes	21.34	0.94	Donor 1	Day9	Blocked	Qualified
Lymphocytes[1]	CD14neg	-1.47	64.48	Donor 1	Day9	Blocked	Qualified
Monocytes[1]	CD19-	1.29	17.76	Donor 1	Day9	Blocked	Qualified
NK[1]	CD14neg	5.95	12.69	Donor 1	Day9	Blocked	Qualified
Non-Classical Mono[1]	Monocytes	-2.02	4.19	Donor 1	Day9	Blocked	Qualified

PBMC[2]	Non-Neutrophils	-3.83	21.43	Donor 1	Day9	Blocked	Qualified
Bcell[1]	CD3neg	19.22	10.82	Donor 1	Day30	Blocked	Qualified
CD4+ T Cells[1]	T Cells	14.00	49.28	Donor 1	Day30	Blocked	Qualified
CD8+ T Cells[1]	T Cells	2.28	8.40	Donor 1	Day30	Blocked	Qualified
Classical Mono[1]	Monocytes	-24.45	9.44	Donor 1	Day30	Blocked	Qualified
Intermediate Mono[1]	Monocytes	-14.50	0.66	Donor 1	Day30	Blocked	Qualified
Lymphocytes[1]	CD14neg	11.63	73.06	Donor 1	Day30	Blocked	Qualified
Monocytes[1]	CD19-	-25.90	12.99	Donor 1	Day30	Blocked	Qualified
NK[1]	CD14neg	-19.67	9.62	Donor 1	Day30	Blocked	Qualified
Non-Classical Mono[1]	Monocytes	-33.53	2.84	Donor 1	Day30	Blocked	Non-Qualifed
PBMC[2]	Non-Neutrophils	22.88	27.38	Donor 1	Day30	Blocked	Qualified
Bcell[1]	CD3neg	11.68	10.13	Donor 1	Day60	Blocked	Qualified
CD4+ T Cells[1]	T Cells	14.66	49.57	Donor 1	Day60	Blocked	Qualified
CD8+ T Cells[1]	T Cells	3.64	8.52	Donor 1	Day60	Blocked	Qualified
Classical Mono[1]	Monocytes	-31.91	8.51	Donor 1	Day60	Blocked	Non-Qualifed
Intermediate Mono[1]	Monocytes	-52.12	0.37	Donor 1	Day60	Blocked	Non-Qualifed
Lymphocytes[1]	CD14neg	11.39	72.90	Donor 1	Day60	Blocked	Qualified
Monocytes[1]	CD19-	-32.82	11.78	Donor 1	Day60	Blocked	Non-Qualifed
NK[1]	CD14neg	-9.20	10.87	Donor 1	Day60	Blocked	Qualified
Non-Classical Mono[1]	Monocytes	-31.94	2.91	Donor 1	Day60	Blocked	Non-Qualifed
PBMC[2]	Non-Neutrophils	18.28	26.35	Donor 1	Day60	Blocked	Qualified

Bcell[1]	CD3neg	0.00	14.30	Donor 2	Day1	Blocked	Qualified
CD4+ T Cells[1]	T Cells	0.00	31.87	Donor 2	Day1	Blocked	Qualified
CD8+ T Cells[1]	T Cells	0.00	10.46	Donor 2	Day1	Blocked	Qualified
Classical Mono[1]	Monocytes	0.00	13.89	Donor 2	Day1	Blocked	Qualified
Intermediate Mono[1]	Monocytes	0.00	0.86	Donor 2	Day1	Blocked	Qualified
Lymphocytes[1]	CD14neg	0.00	71.40	Donor 2	Day1	Blocked	Qualified
Monocytes[1]	CD19-	0.00	18.59	Donor 2	Day1	Blocked	Qualified
NK[1]	CD14neg	0.00	5.50	Donor 2	Day1	Blocked	Qualified
Non-Classical Mono[1]	Monocytes	0.00	3.84	Donor 2	Day1	Blocked	Qualified
PBMC[2]	Non-Neutrophils	0.00	25.05	Donor 2	Day1	Blocked	Qualified
Bcell[1]	CD3neg	5.08	15.03	Donor 2	Day9	Blocked	Qualified
CD4+ T Cells[1]	T Cells	4.31	33.25	Donor 2	Day9	Blocked	Qualified
CD8+ T Cells[1]	T Cells	12.28	11.74	Donor 2	Day9	Blocked	Qualified
Classical Mono[1]	Monocytes	-8.24	12.75	Donor 2	Day9	Blocked	Qualified
Intermediate Mono[1]	Monocytes	-31.22	0.59	Donor 2	Day9	Blocked	Non-Qualifed
Lymphocytes[1]	CD14neg	3.92	74.20	Donor 2	Day9	Blocked	Qualified
Monocytes[1]	CD19-	-9.87	16.76	Donor 2	Day9	Blocked	Qualified
NK[1]	CD14neg	-9.54	4.98	Donor 2	Day9	Blocked	Qualified
Non-Classical Mono[1]	Monocytes	-11.15	3.41	Donor 2	Day9	Blocked	Qualified
PBMC[2]	Non-Neutrophils	7.20	26.85	Donor 2	Day9	Blocked	Qualified
Bcell[1]	CD3neg	13.84	16.28	Donor 2	Day30	Blocked	Qualified
CD4+ T Cells[1]	T Cells	11.31	35.48	Donor 2	Day30	Blocked	Qualified
CD8+ T Cells[1]	T Cells	21.86	12.74	Donor 2	Day30	Blocked	Qualified

Classical Mono[1]	Monocytes	-25.22	10.39	Donor 2	Day30	Blocked	Non-Qualifed
Intermediate Mono[1]	Monocytes	-43.31	0.49	Donor 2	Day30	Blocked	Non-Qualifed
Lymphocytes[1]	CD14neg	10.23	78.70	Donor 2	Day30	Blocked	Qualified
Monocytes[1]	CD19-	-29.28	13.15	Donor 2	Day30	Blocked	Non-Qualifed
NK[1]	CD14neg	-27.38	4.00	Donor 2	Day30	Blocked	Non-Qualifed
Non-Classical Mono[1]	Monocytes	-41.59	2.24	Donor 2	Day30	Blocked	Non-Qualifed
PBMC[2]	Non-Neutrophils	29.81	32.51	Donor 2	Day30	Blocked	Non-Qualifed
Bcell[1]	CD3neg	13.61	16.25	Donor 2	Day60	Blocked	Qualified
CD4+ T Cells[1]	T Cells	9.49	34.90	Donor 2	Day60	Blocked	Qualified
CD8+ T Cells[1]	T Cells	20.61	12.61	Donor 2	Day60	Blocked	Qualified
Classical Mono[1]	Monocytes	-24.16	10.54	Donor 2	Day60	Blocked	Qualified
Intermediate Mono[1]	Monocytes	-71.59	0.24	Donor 2	Day60	Blocked	Non-Qualifed
Lymphocytes[1]	CD14neg	9.74	78.35	Donor 2	Day60	Blocked	Qualified
Monocytes[1]	CD19-	-28.75	13.25	Donor 2	Day60	Blocked	Non-Qualifed
NK[1]	CD14neg	-28.04	3.96	Donor 2	Day60	Blocked	Non-Qualifed
Non-Classical Mono[1]	Monocytes	-35.83	2.46	Donor 2	Day60	Blocked	Non-Qualifed
PBMC[2]	Non-Neutrophils	24.26	31.12	Donor 2	Day60	Blocked	Qualified
Bcell[1]	CD3neg	0.00	5.43	Donor 3	Day1	Blocked	Qualified
CD4+ T Cells[1]	T Cells	0.00	17.65	Donor 3	Day1	Blocked	Qualified

CD8+ T Cells[1]	T Cells	0.00	6.87	Donor 3	Day1	Blocked	Qualified
Classical Mono[1]	Monocytes	0.00	24.37	Donor 3	Day1	Blocked	Qualified
Intermediate Mono[1]	Monocytes	0.00	1.82	Donor 3	Day1	Blocked	Qualified
Lymphocytes[1]	CD14neg	0.00	38.37	Donor 3	Day1	Blocked	Qualified
Monocytes[1]	CD19-	0.00	32.03	Donor 3	Day1	Blocked	Qualified
NK[1]	CD14neg	0.00	24.64	Donor 3	Day1	Blocked	Qualified
Non-Classical Mono[1]	Monocytes	0.00	5.85	Donor 3	Day1	Blocked	Qualified
PBMC[2]	Non-Neutrophils	0.00	26.46	Donor 3	Day1	Blocked	Qualified
Bcell[1]	CD3neg	-10.28	4.87	Donor 3	Day9	Blocked	Qualified
CD4+ T Cells[1]	T Cells	-7.67	16.30	Donor 3	Day9	Blocked	Qualified
CD8+ T Cells[1]	T Cells	-3.61	6.62	Donor 3	Day9	Blocked	Qualified
Classical Mono[1]	Monocytes	12.98	27.54	Donor 3	Day9	Blocked	Qualified
Intermediate Mono[1]	Monocytes	-12.62	1.59	Donor 3	Day9	Blocked	Qualified
Lymphocytes[1]	CD14neg	-6.21	35.99	Donor 3	Day9	Blocked	Qualified
Monocytes[1]	CD19-	13.20	36.26	Donor 3	Day9	Blocked	Qualified
NK[1]	CD14neg	-4.70	23.48	Donor 3	Day9	Blocked	Qualified
Non-Classical Mono[1]	Monocytes	22.22	7.15	Donor 3	Day9	Blocked	Qualified
PBMC[2]	Non-Neutrophils	-16.56	22.08	Donor 3	Day9	Blocked	Qualified
Bcell[1]	CD3neg	15.26	6.26	Donor 3	Day30	Blocked	Qualified
CD4+ T Cells[1]	T Cells	23.31	21.77	Donor 3	Day30	Blocked	Qualified
CD8+ T Cells[1]	T Cells	12.60	7.73	Donor 3	Day30	Blocked	Qualified
Classical Mono[1]	Monocytes	-3.30	23.57	Donor 3	Day30	Blocked	Qualified

Intermediate Mono[1]	Monocytes	-53.21	0.85	Donor 3	Day30	Blocked	Non-Qualifed
Lymphocytes[1]	CD14neg	14.68	44.01	Donor 3	Day30	Blocked	Qualified
Monocytes[1]	CD19-	-4.44	30.61	Donor 3	Day30	Blocked	Qualified
NK[1]	CD14neg	-9.28	22.35	Donor 3	Day30	Blocked	Qualified
Non-Classical Mono[1]	Monocytes	2.21	5.98	Donor 3	Day30	Blocked	Qualified
PBMC[2]	Non-Neutrophils	4.62	27.69	Donor 3	Day30	Blocked	Qualified
Bcell[1]	CD3neg	10.99	6.02	Donor 3	Day60	Blocked	Qualified
CD4+ T Cells[1]	T Cells	22.98	21.71	Donor 3	Day60	Blocked	Qualified
CD8+ T Cells[1]	T Cells	15.19	7.91	Donor 3	Day60	Blocked	Qualified
Classical Mono[1]	Monocytes	-2.78	23.70	Donor 3	Day60	Blocked	Qualified
Intermediate Mono[1]	Monocytes	1.81	1.85	Donor 3	Day60	Blocked	Qualified
Lymphocytes[1]	CD14neg	13.26	43.46	Donor 3	Day60	Blocked	Qualified
Monocytes[1]	CD19-	-4.15	30.70	Donor 3	Day60	Blocked	Qualified
NK[1]	CD14neg	-10.36	22.08	Donor 3	Day60	Blocked	Qualified
Non-Classical Mono[1]	Monocytes	-11.62	5.17	Donor 3	Day60	Blocked	Qualified
PBMC[2]	Non-Neutrophils	7.78	28.52	Donor 3	Day60	Blocked	Qualified
Bcell[1]	CD3neg	0.00	9.96	Donor 1	Day1	Unblocked	Qualified
CD4+ T Cells[1]	T Cells	0.00	46.62	Donor 1	Day1	Unblocked	Qualified
CD8+ T Cells[1]	T Cells	0.00	8.37	Donor 1	Day1	Unblocked	Qualified
Classical Mono[1]	Monocytes	0.00	9.67	Donor 1	Day1	Unblocked	Qualified
Intermediate Mono[1]	Monocytes	0.00	0.92	Donor 1	Day1	Unblocked	Qualified
Lymphocytes[1]	CD14neg	0.00	69.35	Donor 1	Day1	Unblocked	Qualified

Monocytes[1]	CD19-	0.00	13.90	Donor 1	Day1	Unblocked	Qualified
NK[1]	CD14neg	0.00	11.11	Donor 1	Day1	Unblocked	Qualified
Non-Classical Mono[1]	Monocytes	0.00	3.30	Donor 1	Day1	Unblocked	Qualified
PBMC[2]	Non-Neutrophils	0.00	25.01	Donor 1	Day1	Unblocked	Qualified
Bcell[1]	CD3neg	-16.97	8.27	Donor 1	Day9	Unblocked	Qualified
CD4+ T Cells[1]	T Cells	1.17	47.16	Donor 1	Day9	Unblocked	Qualified
CD8+ T Cells[1]	T Cells	2.79	8.60	Donor 1	Day9	Unblocked	Qualified
Classical Mono[1]	Monocytes	14.39	11.07	Donor 1	Day9	Unblocked	Qualified
Intermediate Mono[1]	Monocytes	25.50	1.16	Donor 1	Day9	Unblocked	Qualified
Lymphocytes[1]	CD14neg	-1.50	68.31	Donor 1	Day9	Unblocked	Qualified
Monocytes[1]	CD19-	12.28	15.61	Donor 1	Day9	Unblocked	Qualified
NK[1]	CD14neg	3.19	11.46	Donor 1	Day9	Unblocked	Qualified
Non-Classical Mono[1]	Monocytes	-1.18	3.26	Donor 1	Day9	Unblocked	Qualified
PBMC[2]	Non-Neutrophils	-1.07	24.74	Donor 1	Day9	Unblocked	Qualified
Bcell[1]	CD3neg	2.66	10.22	Donor 1	Day30	Unblocked	Qualified
CD4+ T Cells[1]	T Cells	6.00	49.41	Donor 1	Day30	Unblocked	Qualified
CD8+ T Cells[1]	T Cells	-0.78	8.30	Donor 1	Day30	Unblocked	Qualified
Classical Mono[1]	Monocytes	-5.74	9.12	Donor 1	Day30	Unblocked	Qualified
Intermediate Mono[1]	Monocytes	8.64	1.00	Donor 1	Day30	Unblocked	Qualified
Lymphocytes[1]	CD14neg	4.45	72.44	Donor 1	Day30	Unblocked	Qualified
Monocytes[1]	CD19-	-7.77	12.82	Donor 1	Day30	Unblocked	Qualified
NK[1]	CD14neg	-14.04	9.55	Donor 1	Day30	Unblocked	Qualified

Non-Classical Mono[1]	Monocytes	-20.34	2.63	Donor 1	Day30	Unblocked	Qualified
PBMC[2]	Non-Neutrophils	10.77	27.70	Donor 1	Day30	Unblocked	Qualified
Bcell[1]	CD3neg	-10.52	8.91	Donor 1	Day60	Unblocked	Qualified
CD4+ T Cells[1]	T Cells	6.14	49.48	Donor 1	Day60	Unblocked	Qualified
CD8+ T Cells[1]	T Cells	-0.61	8.32	Donor 1	Day60	Unblocked	Qualified
Classical Mono[1]	Monocytes	-19.76	7.76	Donor 1	Day60	Unblocked	Qualified
Intermediate Mono[1]	Monocytes	-32.17	0.63	Donor 1	Day60	Unblocked	Non-Qualifed
Lymphocytes[1]	CD14neg	2.60	71.16	Donor 1	Day60	Unblocked	Qualified
Monocytes[1]	CD19-	-19.34	11.21	Donor 1	Day60	Unblocked	Qualified
NK[1]	CD14neg	-1.22	10.97	Donor 1	Day60	Unblocked	Qualified
Non-Classical Mono[1]	Monocytes	-14.40	2.82	Donor 1	Day60	Unblocked	Qualified
PBMC[2]	Non-Neutrophils	9.51	27.39	Donor 1	Day60	Unblocked	Qualified
Bcell[1]	CD3neg	0.00	15.02	Donor 2	Day1	Unblocked	Qualified
CD4+ T Cells[1]	T Cells	0.00	34.95	Donor 2	Day1	Unblocked	Qualified
CD8+ T Cells[1]	T Cells	0.00	11.14	Donor 2	Day1	Unblocked	Qualified
Classical Mono[1]	Monocytes	0.00	9.77	Donor 2	Day1	Unblocked	Qualified
Intermediate Mono[1]	Monocytes	0.00	0.97	Donor 2	Day1	Unblocked	Qualified
Lymphocytes[1]	CD14neg	0.00	74.79	Donor 2	Day1	Unblocked	Qualified
Monocytes[1]	CD19-	0.00	13.53	Donor 2	Day1	Unblocked	Qualified
NK[1]	CD14neg	0.00	5.41	Donor 2	Day1	Unblocked	Qualified
Non-Classical Mono[1]	Monocytes	0.00	2.79	Donor 2	Day1	Unblocked	Qualified

PBMC[2]	Non-Neutrophils	0.00	30.11	Donor 2	Day1	Unblocked	Qualified
Bcell[1]	CD3neg	3.89	15.61	Donor 2	Day9	Unblocked	Qualified
CD4+ T Cells[1]	T Cells	-2.89	33.94	Donor 2	Day9	Unblocked	Qualified
CD8+ T Cells[1]	T Cells	3.83	11.56	Donor 2	Day9	Unblocked	Qualified
Classical Mono[1]	Monocytes	17.35	11.47	Donor 2	Day9	Unblocked	Qualified
Intermediate Mono[1]	Monocytes	-7.10	0.90	Donor 2	Day9	Unblocked	Qualified
Lymphocytes[1]	CD14neg	-0.19	74.64	Donor 2	Day9	Unblocked	Qualified
Monocytes[1]	CD19-	15.45	15.63	Donor 2	Day9	Unblocked	Qualified
NK[1]	CD14neg	-4.65	5.16	Donor 2	Day9	Unblocked	Qualified
Non-Classical Mono[1]	Monocytes	15.31	3.22	Donor 2	Day9	Unblocked	Qualified
PBMC[2]	Non-Neutrophils	-6.71	28.09	Donor 2	Day9	Unblocked	Qualified
Bcell[1]	CD3neg	4.36	15.68	Donor 2	Day30	Unblocked	Qualified
CD4+ T Cells[1]	T Cells	4.05	36.37	Donor 2	Day30	Unblocked	Qualified
CD8+ T Cells[1]	T Cells	14.29	12.73	Donor 2	Day30	Unblocked	Qualified
Classical Mono[1]	Monocytes	-2.36	9.54	Donor 2	Day30	Unblocked	Qualified
Intermediate Mono[1]	Monocytes	-51.32	0.47	Donor 2	Day30	Unblocked	Qualified
Lymphocytes[1]	CD14neg	4.99	78.52	Donor 2	Day30	Unblocked	Qualified
Monocytes[1]	CD19-	-11.12	12.03	Donor 2	Day30	Unblocked	Qualified
NK[1]	CD14neg	-30.25	3.78	Donor 2	Day30	Unblocked	Non-Qualifed
Non-Classical Mono[1]	Monocytes	-28.71	1.99	Donor 2	Day30	Unblocked	Non-Qualifed
PBMC[2]	Non-Neutrophils	11.07	33.44	Donor 2	Day30	Unblocked	Qualified

Bcell[1]	CD3neg	5.40	15.83	Donor 2	Day60	Unblocked	Qualified
CD4+ T Cells[1]	T Cells	0.86	35.25	Donor 2	Day60	Unblocked	Qualified
CD8+ T Cells[1]	T Cells	13.04	12.59	Donor 2	Day60	Unblocked	Qualified
Classical Mono[1]	Monocytes	5.74	10.34	Donor 2	Day60	Unblocked	Qualified
Intermediate Mono[1]	Monocytes	-70.78	0.28	Donor 2	Day60	Unblocked	Non-Qualifed
Lymphocytes[1]	CD14neg	4.00	77.78	Donor 2	Day60	Unblocked	Qualified
Monocytes[1]	CD19-	-2.69	13.17	Donor 2	Day60	Unblocked	Qualified
NK[1]	CD14neg	-21.78	4.23	Donor 2	Day60	Unblocked	Qualified
Non-Classical Mono[1]	Monocytes	-8.59	2.55	Donor 2	Day60	Unblocked	Qualified
PBMC[2]	Non-Neutrophils	1.67	30.61	Donor 2	Day60	Unblocked	Qualified
Bcell[1]	CD3neg	0.00	5.41	Donor 3	Day1	Unblocked	Qualified
CD4+ T Cells[1]	T Cells	0.00	20.55	Donor 3	Day1	Unblocked	Qualified
CD8+ T Cells[1]	T Cells	0.00	7.42	Donor 3	Day1	Unblocked	Qualified
Classical Mono[1]	Monocytes	0.00	21.44	Donor 3	Day1	Unblocked	Qualified
Intermediate Mono[1]	Monocytes	0.00	2.27	Donor 3	Day1	Unblocked	Qualified
Lymphocytes[1]	CD14neg	0.00	40.92	Donor 3	Day1	Unblocked	Qualified
Monocytes[1]	CD19-	0.00	29.13	Donor 3	Day1	Unblocked	Qualified
NK[1]	CD14neg	0.00	24.93	Donor 3	Day1	Unblocked	Qualified
Non-Classical Mono[1]	Monocytes	0.00	5.43	Donor 3	Day1	Unblocked	Qualified
PBMC[2]	Non-Neutrophils	0.00	28.00	Donor 3	Day1	Unblocked	Qualified
Bcell[1]	CD3neg	8.71	5.88	Donor 3	Day9	Unblocked	Qualified
CD4+ T Cells[1]	T Cells	-3.10	19.91	Donor 3	Day9	Unblocked	Qualified
CD8+ T Cells[1]	T Cells	-9.08	6.75	Donor 3	Day9	Unblocked	Qualified

Classical Mono[1]	Monocytes	10.82	23.76	Donor 3	Day9	Unblocked	Qualified
Intermediate Mono[1]	Monocytes	-5.11	2.15	Donor 3	Day9	Unblocked	Qualified
Lymphocytes[1]	CD14neg	-2.34	39.97	Donor 3	Day9	Unblocked	Qualified
Monocytes[1]	CD19-	11.76	32.56	Donor 3	Day9	Unblocked	Qualified
NK[1]	CD14neg	-4.80	23.74	Donor 3	Day9	Unblocked	Qualified
Non-Classical Mono[1]	Monocytes	22.05	6.63	Donor 3	Day9	Unblocked	Qualified
PBMC[2]	Non-Neutrophils	-12.88	24.40	Donor 3	Day9	Unblocked	Qualified
Bcell[1]	CD3neg	14.70	6.20	Donor 3	Day30	Unblocked	Qualified
CD4+ T Cells[1]	T Cells	20.21	24.70	Donor 3	Day30	Unblocked	Qualified
CD8+ T Cells[1]	T Cells	9.57	8.13	Donor 3	Day30	Unblocked	Qualified
Classical Mono[1]	Monocytes	2.31	21.93	Donor 3	Day30	Unblocked	Qualified
Intermediate Mono[1]	Monocytes	-51.03	1.11	Donor 3	Day30	Unblocked	Non-Qualifed
Lymphocytes[1]	CD14neg	14.58	46.89	Donor 3	Day30	Unblocked	Qualified
Monocytes[1]	CD19-	-14.73	21.26	Donor 3	Day30	Unblocked	Qualified
NK[1]	CD14neg	-12.66	4.74	Donor 3	Day30	Unblocked	Qualified
Non-Classical Mono[1]	Monocytes	1.15	28.32	Donor 3	Day30	Unblocked	Qualified
PBMC[2]	Non-Neutrophils	-4.26	27.89	Donor 3	Day30	Unblocked	Qualified
Bcell[1]	CD3neg	20.27	6.50	Donor 3	Day60	Unblocked	Qualified
CD4+ T Cells[1]	T Cells	15.69	23.77	Donor 3	Day60	Unblocked	Qualified
CD8+ T Cells[1]	T Cells	8.39	8.05	Donor 3	Day60	Unblocked	Qualified
Classical Mono[1]	Monocytes	1.13	21.68	Donor 3	Day60	Unblocked	Qualified

Intermediate Mono[1]	Monocytes	-14.41	1.94	Donor 3	Day60	Unblocked	Qualified
Lymphocytes[1]	CD14neg	13.38	46.40	Donor 3	Day60	Unblocked	Qualified
Monocytes[1]	CD19-	-3.15	28.21	Donor 3	Day60	Unblocked	Qualified
NK[1]	CD14neg	-16.08	20.93	Donor 3	Day60	Unblocked	Qualified
Non-Classical Mono[1]	Monocytes	-15.35	4.60	Donor 3	Day60	Unblocked	Qualified
PBMC[2]	Non-Neutrophils	2.66	28.75	Donor 3	Day60	Unblocked	Qualified

[1]W%D was determined based on percent of PBMC
[2]W%D was determined based on percent of WBC

Table 19. Post-Processing Stability for Readouts Subsets

Population	Parent Population	W%D ChgD1	Average	Donor	Post-Processing Day	Condition	
Bcell[1]	CD3neg	0.00	179.77	Donor 1	Day1	Blocked	Qualified
CD4+ T Cells[1]	T Cells	0.00	224.68	Donor 1	Day1	Blocked	Qualified
CD8+ T Cells[1]	T Cells	0.00	206.38	Donor 1	Day1	Blocked	Qualified
Classical Mono[1]	Monocytes	0.00	689.55	Donor 1	Day1	Blocked	Qualified
Intermediate Mono[1]	Monocytes	0.00	812.95	Donor 1	Day1	Blocked	Qualified
Lymphocytes[1]	CD14neg	0.00	220.49	Donor 1	Day1	Blocked	Qualified
Monocytes[1]	CD19-	0.00	631.59	Donor 1	Day1	Blocked	Qualified
NK[1]	CD14neg	0.00	207.78	Donor 1	Day1	Blocked	Qualified
Non-Classical Mono[1]	Monocytes	0.00	421.89	Donor 1	Day1	Blocked	Qualified
PBMC[1]	Non-Neutrophils	0.00	256.60	Donor 1	Day1	Blocked	Qualified
Bcell[1]	CD3neg	-1.11	177.78	Donor 1	Day9	Blocked	Qualified
CD4+ T Cells[1]	T Cells	0.45	225.70	Donor 1	Day9	Blocked	Qualified

CD8+ T Cells[1]	T Cells	3.18	212.93	Donor 1	Day9	Blocked	Qualified
Classical Mono[1]	Monocytes	-4.83	656.27	Donor 1	Day9	Blocked	Qualified
Intermediate Mono[1]	Monocytes	-10.82	724.99	Donor 1	Day9	Blocked	Qualified
Lymphocytes[1]	CD14neg	0.95	222.59	Donor 1	Day9	Blocked	Qualified
Monocytes[1]	CD19-	-4.86	600.90	Donor 1	Day9	Blocked	Qualified
NK[1]	CD14neg	6.27	220.80	Donor 1	Day9	Blocked	Qualified
Non-Classical Mono[1]	Monocytes	-4.65	402.28	Donor 1	Day9	Blocked	Qualified
PBMC[1]	Non-Neutrophils	2.27	262.43	Donor 1	Day9	Blocked	Qualified
Bcell[1]	CD3neg	-0.35	179.14	Donor 1	Day30	Blocked	Qualified
CD4+ T Cells[1]	T Cells	-1.55	221.19	Donor 1	Day30	Blocked	Qualified
CD8+ T Cells[1]	T Cells	1.36	209.19	Donor 1	Day30	Blocked	Qualified
Classical Mono[1]	Monocytes	-11.63	609.32	Donor 1	Day30	Blocked	Qualified
Intermediate Mono[1]	Monocytes	-7.68	750.50	Donor 1	Day30	Blocked	Qualified
Lymphocytes[1]	CD14neg	-1.91	216.29	Donor 1	Day30	Blocked	Qualified
Monocytes[1]	CD19-	-9.27	573.03	Donor 1	Day30	Blocked	Qualified
NK[1]	CD14neg	-1.18	205.33	Donor 1	Day30	Blocked	Qualified
Non-Classical Mono[1]	Monocytes	-2.60	410.94	Donor 1	Day30	Blocked	Qualified
PBMC[1]	Non-Neutrophils	-5.45	242.63	Donor 1	Day30	Blocked	Qualified
Bcell[1]	CD3neg	3.76	186.54	Donor 1	Day60	Blocked	Qualified
CD4+ T Cells[1]	T Cells	5.29	236.57	Donor 1	Day60	Blocked	Qualified
CD8+ T Cells[1]	T Cells	5.09	216.89	Donor 1	Day60	Blocked	Qualified
Classical Mono[1]	Monocytes	-6.78	642.80	Donor 1	Day60	Blocked	Qualified

Intermediate Mono[1]	Monocytes	-12.18	713.97	Donor 1	Day60	Blocked	Qualified
Lymphocytes[1]	CD14neg	4.28	229.92	Donor 1	Day60	Blocked	Qualified
Monocytes[1]	CD19-	-5.90	594.30	Donor 1	Day60	Blocked	Qualified
NK[1]	CD14neg	4.86	217.88	Donor 1	Day60	Blocked	Qualified
Non-Classical Mono[1]	Monocytes	-0.66	419.11	Donor 1	Day60	Blocked	Qualified
PBMC[1]	Non-Neutrophils	-1.11	253.7387	Donor 1	Day60	Blocked	Qualified
Bcell[1]	CD3neg	0.00	168.81	Donor 2	Day1	Blocked	Qualified
CD4+ T Cells[1]	T Cells	0.00	221.29	Donor 2	Day1	Blocked	Qualified
CD8+ T Cells[1]	T Cells	0.00	188.82	Donor 2	Day1	Blocked	Qualified
Classical Mono[1]	Monocytes	0.00	662.11	Donor 2	Day1	Blocked	Qualified
Intermediate Mono[1]	Monocytes	0.00	901.29	Donor 2	Day1	Blocked	Qualified
Lymphocytes[1]	CD14neg	0.00	207.98	Donor 2	Day1	Blocked	Qualified
Monocytes[1]	CD19-	0.00	615.11	Donor 2	Day1	Blocked	Qualified
NK[1]	CD14neg	0.00	202.08	Donor 2	Day1	Blocked	Qualified
Non-Classical Mono[1]	Monocytes	0.00	360.35	Donor 2	Day1	Blocked	Qualified
PBMC[1]	Non-Neutrophils	0.00	247.23	Donor 2	Day1	Blocked	Qualified
Bcell[1]	CD3neg	-0.44	168.07	Donor 2	Day9	Blocked	Qualified
CD4+ T Cells[1]	T Cells	-2.68	215.36	Donor 2	Day9	Blocked	Qualified
CD8+ T Cells[1]	T Cells	0.69	190.11	Donor 2	Day9	Blocked	Qualified
Classical Mono[1]	Monocytes	-8.78	603.98	Donor 2	Day9	Blocked	Qualified
Intermediate Mono[1]	Monocytes	-0.87	893.46	Donor 2	Day9	Blocked	Qualified
Lymphocytes[1]	CD14neg	-1.65	204.55	Donor 2	Day9	Blocked	Qualified

Monocytes[1]	CD19-	-8.27	564.25	Donor 2	Day9	Blocked	Qualified
NK[1]	CD14neg	1.60	205.31	Donor 2	Day9	Blocked	Qualified
Non-Classical Mono[1]	Monocytes	-2.33	351.96	Donor 2	Day9	Blocked	Qualified
PBMC[1]	Non-Neutrophils	-4.18	236.88	Donor 2	Day9	Blocked	Qualified
Bcell[1]	CD3neg	0.48	169.62	Donor 2	Day30	Blocked	Qualified
CD4+ T Cells[1]	T Cells	-3.99	212.46	Donor 2	Day30	Blocked	Qualified
CD8+ T Cells[1]	T Cells	1.16	191.00	Donor 2	Day30	Blocked	Qualified
Classical Mono[1]	Monocytes	-8.75	604.16	Donor 2	Day30	Blocked	Qualified
Intermediate Mono[1]	Monocytes	-7.17	836.70	Donor 2	Day30	Blocked	Qualified
Lymphocytes[1]	CD14neg	-3.22	201.28	Donor 2	Day30	Blocked	Qualified
Monocytes[1]	CD19-	-7.21	570.79	Donor 2	Day30	Blocked	Qualified
NK[1]	CD14neg	-6.53	188.88	Donor 2	Day30	Blocked	Qualified
Non-Classical Mono[1]	Monocytes	-7.10	334.78	Donor 2	Day30	Blocked	Qualified
PBMC[1]	Non-Neutrophils	-8.44	226.36	Donor 2	Day30	Blocked	Qualified
Bcell[1]	CD3neg	3.96	175.50	Donor 2	Day60	Blocked	Qualified
CD4+ T Cells[1]	T Cells	0.50	222.39	Donor 2	Day60	Blocked	Qualified
CD8+ T Cells[1]	T Cells	4.54	197.39	Donor 2	Day60	Blocked	Qualified
Classical Mono[1]	Monocytes	-7.05	615.41	Donor 2	Day60	Blocked	Qualified
Intermediate Mono[1]	Monocytes	-15.98	757.25	Donor 2	Day60	Blocked	Qualified
Lymphocytes[1]	CD14neg	1.40	210.89	Donor 2	Day60	Blocked	Qualified
Monocytes[1]	CD19-	-6.78	573.39	Donor 2	Day60	Blocked	Qualified
NK[1]	CD14neg	0.81	203.72	Donor 2	Day60	Blocked	Qualified

Non-Classical Mono[1]	Monocytes	-1.68	354.31	Donor 2	Day60	Blocked	Qualified
PBMC[1]	Non-Neutrophils	-3.62	238.2643	Donor 2	Day60	Blocked	Qualified
Bcell[1]	CD3neg	0.00	193.93	Donor 3	Day1	Blocked	Qualified
CD4+ T Cells[1]	T Cells	0.00	243.83	Donor 3	Day1	Blocked	Qualified
CD8+ T Cells[1]	T Cells	0.00	222.04	Donor 3	Day1	Blocked	Qualified
Classical Mono[1]	Monocytes	0.00	748.74	Donor 3	Day1	Blocked	Qualified
Intermediate Mono[1]	Monocytes	0.00	1009.71	Donor 3	Day1	Blocked	Qualified
Lymphocytes[1]	CD14neg	0.00	244.02	Donor 3	Day1	Blocked	Qualified
Monocytes[1]	CD19-	0.00	709.23	Donor 3	Day1	Blocked	Qualified
NK[1]	CD14neg	0.00	249.54	Donor 3	Day1	Blocked	Qualified
Non-Classical Mono[1]	Monocytes	0.00	412.10	Donor 3	Day1	Blocked	Qualified
PBMC[1]	Non-Neutrophils	0.00	337.89	Donor 3	Day1	Blocked	Qualified
Bcell[1]	CD3neg	-1.64	190.75	Donor 3	Day9	Blocked	Qualified
CD4+ T Cells[1]	T Cells	-2.11	238.69	Donor 3	Day9	Blocked	Qualified
CD8+ T Cells[1]	T Cells	1.09	224.46	Donor 3	Day9	Blocked	Qualified
Classical Mono[1]	Monocytes	-6.79	697.92	Donor 3	Day9	Blocked	Qualified
Intermediate Mono[1]	Monocytes	-15.15	856.74	Donor 3	Day9	Blocked	Qualified
Lymphocytes[1]	CD14neg	-0.36	243.15	Donor 3	Day9	Blocked	Qualified
Monocytes[1]	CD19-	-7.73	654.42	Donor 3	Day9	Blocked	Qualified
NK[1]	CD14neg	3.01	257.05	Donor 3	Day9	Blocked	Qualified
Non-Classical Mono[1]	Monocytes	1.46	418.13	Donor 3	Day9	Blocked	Qualified

PBMC[1]	Non-Neutrophils	3.84	350.87	Donor 3	Day9	Blocked	Qualified
Bcell[1]	CD3neg	-2.54	189.00	Donor 3	Day30	Blocked	Qualified
CD4+ T Cells[1]	T Cells	-4.13	233.76	Donor 3	Day30	Blocked	Qualified
CD8+ T Cells[1]	T Cells	-0.30	221.37	Donor 3	Day30	Blocked	Qualified
Classical Mono[1]	Monocytes	-13.78	645.54	Donor 3	Day30	Blocked	Qualified
Intermediate Mono[1]	Monocytes	-17.83	829.72	Donor 3	Day30	Blocked	Qualified
Lymphocytes[1]	CD14neg	-5.17	231.41	Donor 3	Day30	Blocked	Qualified
Monocytes[1]	CD19-	-14.35	607.48	Donor 3	Day30	Blocked	Qualified
NK[1]	CD14neg	-4.15	239.19	Donor 3	Day30	Blocked	Qualified
Non-Classical Mono[1]	Monocytes	-1.49	405.94	Donor 3	Day30	Blocked	Qualified
PBMC[1]	Non-Neutrophils	-8.43	309.41	Donor 3	Day30	Blocked	Qualified
Bcell[1]	CD3neg	4.40	202.45	Donor 3	Day60	Blocked	Qualified
CD4+ T Cells[1]	T Cells	2.02	248.76	Donor 3	Day60	Blocked	Qualified
CD8+ T Cells[1]	T Cells	6.92	237.40	Donor 3	Day60	Blocked	Qualified
Classical Mono[1]	Monocytes	2.44	767.02	Donor 3	Day60	Blocked	Qualified
Intermediate Mono[1]	Monocytes	-1.06	998.98	Donor 3	Day60	Blocked	Qualified
Lymphocytes[1]	CD14neg	2.38	249.82	Donor 3	Day60	Blocked	Qualified
Monocytes[1]	CD19-	2.48	726.81	Donor 3	Day60	Blocked	Qualified
NK[1]	CD14neg	5.28	262.71	Donor 3	Day60	Blocked	Qualified
Non-Classical Mono[1]	Monocytes	1.69	419.08	Donor 3	Day60	Blocked	Qualified
PBMC[1]	Non-Neutrophils	-0.28	336.94	Donor 3	Day60	Blocked	Qualified
Bcell[1]	CD3neg	0.00	1302.13	Donor 1	Day1	Unblocked	Qualified

CD4+ T Cells[1]	T Cells	0.00	1400.51	Donor 1	Day1	Unblocked	Qualified
CD8+ T Cells[1]	T Cells	0.00	1604.07	Donor 1	Day1	Unblocked	Qualified
Classical Mono[1]	Monocytes	0.00	2806.45	Donor 1	Day1	Unblocked	Qualified
Intermediate Mono[1]	Monocytes	0.00	2045.52	Donor 1	Day1	Unblocked	Qualified
Lymphocytes[1]	CD14neg	0.00	1417.51	Donor 1	Day1	Unblocked	Qualified
Monocytes[1]	CD19-	0.00	2904.58	Donor 1	Day1	Unblocked	Qualified
NK[1]	CD14neg	0.00	2774.19	Donor 1	Day1	Unblocked	Qualified
Non-Classical Mono[1]	Monocytes	0.00	3392.89	Donor 1	Day1	Unblocked	Qualified
PBMC[1]	Non-Neutrophils	0.00	1666.22	Donor 1	Day1	Unblocked	Qualified
Bcell[1]	CD3neg	-13.64	1124.51	Donor 1	Day9	Unblocked	Qualified
CD4+ T Cells[1]	T Cells	-13.77	1207.71	Donor 1	Day9	Unblocked	Qualified
CD8+ T Cells[1]	T Cells	-14.86	1365.66	Donor 1	Day9	Unblocked	Qualified
Classical Mono[1]	Monocytes	-12.29	2461.55	Donor 1	Day9	Unblocked	Qualified
Intermediate Mono[1]	Monocytes	-1.63	2012.27	Donor 1	Day9	Unblocked	Qualified
Lymphocytes[1]	CD14neg	-13.79	1222.03	Donor 1	Day9	Unblocked	Qualified
Monocytes[1]	CD19-	-12.71	2535.38	Donor 1	Day9	Unblocked	Qualified
NK[1]	CD14neg	-15.79	2336.16	Donor 1	Day9	Unblocked	Qualified
Non-Classical Mono[1]	Monocytes	-14.22	2910.38	Donor 1	Day9	Unblocked	Qualified
PBMC[1]	Non-Neutrophils	-12.32	1460.94	Donor 1	Day9	Unblocked	Qualified
Bcell[1]	CD3neg	-22.95	1003.31	Donor 1	Day30	Unblocked	Qualified
CD4+ T Cells[1]	T Cells	-23.02	1078.14	Donor 1	Day30	Unblocked	Qualified
CD8+ T Cells[1]	T Cells	-27.64	1160.63	Donor 1	Day30	Unblocked	Non-Qualifed

Classical Mono[1]	Monocytes	-19.49	2259.40	Donor 1	Day30	Unblocked	Qualified
Intermediate Mono[1]	Monocytes	-8.42	1873.34	Donor 1	Day30	Unblocked	Qualified
Lymphocytes[1]	CD14neg	-23.63	1082.58	Donor 1	Day30	Unblocked	Qualified
Monocytes[1]	CD19-	-21.00	2294.73	Donor 1	Day30	Unblocked	Qualified
NK[1]	CD14neg	-27.10	2022.49	Donor 1	Day30	Unblocked	Non-Qualifed
Non-Classical Mono[1]	Monocytes	-23.93	2580.89	Donor 1	Day30	Unblocked	Qualified
PBMC[1]	Non-Neutrophils	-24.96	1250.31	Donor 1	Day30	Unblocked	Qualified
Bcell[1]	CD3neg	8.82	1417.00	Donor 1	Day60	Unblocked	Qualified
CD4+ T Cells[1]	T Cells	8.64	1521.46	Donor 1	Day60	Unblocked	Qualified
CD8+ T Cells[1]	T Cells	1.82	1633.23	Donor 1	Day60	Unblocked	Qualified
Classical Mono[1]	Monocytes	-1.07	2776.52	Donor 1	Day60	Unblocked	Qualified
Intermediate Mono[1]	Monocytes	16.86	2390.38	Donor 1	Day60	Unblocked	Qualified
Lymphocytes[1]	CD14neg	7.32	1521.32	Donor 1	Day60	Unblocked	Qualified
Monocytes[1]	CD19-	-1.02	2875.04	Donor 1	Day60	Unblocked	Qualified
NK[1]	CD14neg	-3.18	2685.88	Donor 1	Day60	Unblocked	Qualified
Non-Classical Mono[1]	Monocytes	-5.10	3219.96	Donor 1	Day60	Unblocked	Qualified
PBMC[1]	Non-Neutrophils	3.32	1721.47	Donor 1	Day60	Unblocked	Qualified
Bcell[1]	CD3neg	0.00	1214.19	Donor 2	Day1	Unblocked	Qualified
CD4+ T Cells[1]	T Cells	0.00	1386.55	Donor 2	Day1	Unblocked	Qualified
CD8+ T Cells[1]	T Cells	0.00	1353.35	Donor 2	Day1	Unblocked	Qualified
Classical Mono[1]	Monocytes	0.00	3109.62	Donor 2	Day1	Unblocked	Qualified

Intermediate Mono[1]	Monocytes	0.00	1838.51	Donor 2	Day1	Unblocked	Qualified
Lymphocytes[1]	CD14neg	0.00	1411.92	Donor 2	Day1	Unblocked	Qualified
Monocytes[1]	CD19-	0.00	3012.74	Donor 2	Day1	Unblocked	Qualified
NK[1]	CD14neg	0.00	2305.85	Donor 2	Day1	Unblocked	Qualified
Non-Classical Mono[1]	Monocytes	0.00	3006.77	Donor 2	Day1	Unblocked	Qualified
PBMC[1]	Non-Neutrophils	0.00	1617.53	Donor 2	Day1	Unblocked	Qualified
Bcell[1]	CD3neg	-16.59	1012.77	Donor 2	Day9	Unblocked	Qualified
CD4+ T Cells[1]	T Cells	-20.72	1099.22	Donor 2	Day9	Unblocked	Qualified
CD8+ T Cells[1]	T Cells	-20.43	1076.93	Donor 2	Day9	Unblocked	Qualified
Classical Mono[1]	Monocytes	-17.20	2574.84	Donor 2	Day9	Unblocked	Qualified
Intermediate Mono[1]	Monocytes	-7.88	1693.66	Donor 2	Day9	Unblocked	Qualified
Lymphocytes[1]	CD14neg	-19.67	1134.13	Donor 2	Day9	Unblocked	Qualified
Monocytes[1]	CD19-	-16.45	2517.00	Donor 2	Day9	Unblocked	Qualified
NK[1]	CD14neg	-19.19	1863.41	Donor 2	Day9	Unblocked	Qualified
Non-Classical Mono[1]	Monocytes	-16.28	2517.35	Donor 2	Day9	Unblocked	Qualified
PBMC[1]	Non-Neutrophils	-17.93	1327.51	Donor 2	Day9	Unblocked	Qualified
Bcell[1]	CD3neg	-21.83	949.10	Donor 2	Day30	Unblocked	Qualified
CD4+ T Cells[1]	T Cells	-26.66	1016.96	Donor 2	Day30	Unblocked	Non-Qualifed
CD8+ T Cells[1]	T Cells	-30.12	945.68	Donor 2	Day30	Unblocked	Non-Qualifed
Classical Mono[1]	Monocytes	-21.44	2442.82	Donor 2	Day30	Unblocked	Qualified

Intermediate Mono[1]	Monocytes	-9.41	1665.58	Donor 2	Day30	Unblocked	Qualified
Lymphocytes[1]	CD14neg	-26.62	1036.02	Donor 2	Day30	Unblocked	Non-Qualifed
Monocytes[1]	CD19-	-20.47	2395.92	Donor 2	Day30	Unblocked	Qualified
NK[1]	CD14neg	-26.89	1685.72	Donor 2	Day30	Unblocked	Non-Qualifed
Non-Classical Mono[1]	Monocytes	-22.88	2318.73	Donor 2	Day30	Unblocked	Qualified
PBMC[1]	Non-Neutrophils	-27.22	1177.22	Donor 2	Day30	Unblocked	Non-Qualifed
Bcell[1]	CD3neg	-1.46	1196.51	Donor 2	Day60	Unblocked	Qualified
CD4+ T Cells[1]	T Cells	-6.96	1290.07	Donor 2	Day60	Unblocked	Qualified
CD8+ T Cells[1]	T Cells	-8.60	1236.96	Donor 2	Day60	Unblocked	Qualified
Classical Mono[1]	Monocytes	-11.00	2767.66	Donor 2	Day60	Unblocked	Qualified
Intermediate Mono[1]	Monocytes	9.41	2011.59	Donor 2	Day60	Unblocked	Qualified
Lymphocytes[1]	CD14neg	-6.73	1316.93	Donor 2	Day60	Unblocked	Qualified
Monocytes[1]	CD19-	-9.11	2738.33	Donor 2	Day60	Unblocked	Qualified
NK[1]	CD14neg	-12.73	2012.27	Donor 2	Day60	Unblocked	Qualified
Non-Classical Mono[1]	Monocytes	-10.33	2696.16	Donor 2	Day60	Unblocked	Qualified
PBMC[1]	Non-Neutrophils	-8.01	1487.92	Donor 2	Day60	Unblocked	Qualified
Bcell[1]	CD3neg	0.00	1669.70	Donor 3	Day1	Unblocked	Qualified
CD4+ T Cells[1]	T Cells	0.00	1673.62	Donor 3	Day1	Unblocked	Qualified
CD8+ T Cells[1]	T Cells	0.00	2066.26	Donor 3	Day1	Unblocked	Qualified
Classical Mono[1]	Monocytes	0.00	3678.12	Donor 3	Day1	Unblocked	Qualified

Intermediate Mono[1]	Monocytes	0.00	2527.82	Donor 3	Day1	Unblocked	Qualified
Lymphocytes[1]	CD14neg	0.00	1823.23	Donor 3	Day1	Unblocked	Qualified
Monocytes[1]	CD19-	0.00	3571.08	Donor 3	Day1	Unblocked	Qualified
NK[1]	CD14neg	0.00	3493.54	Donor 3	Day1	Unblocked	Qualified
Non-Classical Mono[1]	Monocytes	0.00	3508.19	Donor 3	Day1	Unblocked	Qualified
PBMC[2]	Non-Neutrophils	0.00	2824.89	Donor 3	Day1	Unblocked	Qualified
Bcell[1]	CD3neg	-18.07	1368.00	Donor 3	Day9	Unblocked	Qualified
CD4+ T Cells[1]	T Cells	-26.22	1234.86	Donor 3	Day9	Unblocked	Non-Qualifed
CD8+ T Cells[1]	T Cells	-26.65	1515.57	Donor 3	Day9	Unblocked	Non-Qualifed
Classical Mono[1]	Monocytes	-22.18	2862.34	Donor 3	Day9	Unblocked	Qualified
Intermediate Mono[1]	Monocytes	-11.49	2237.40	Donor 3	Day9	Unblocked	Qualified
Lymphocytes[1]	CD14neg	-25.09	1365.82	Donor 3	Day9	Unblocked	Qualified
Monocytes[1]	CD19-	-21.70	2795.99	Donor 3	Day9	Unblocked	Qualified
NK[1]	CD14neg	-24.55	2635.75	Donor 3	Day9	Unblocked	Qualified
Non-Classical Mono[1]	Monocytes	-23.81	2672.82	Donor 3	Day9	Unblocked	Qualified
PBMC[1]	Non-Neutrophils	-22.27	2195.81	Donor 3	Day9	Unblocked	Qualified
Bcell[1]	CD3neg	-21.92	1303.74	Donor 3	Day30	Unblocked	Qualified
CD4+ T Cells[1]	T Cells	-24.55	1262.78	Donor 3	Day30	Unblocked	Qualified
CD8+ T Cells[1]	T Cells	-27.11	1506.01	Donor 3	Day30	Unblocked	Non-Qualifed
Classical Mono[1]	Monocytes	-19.98	2943.36	Donor 3	Day30	Unblocked	Qualified

Intermediate Mono[1]	Monocytes	-0.59	2513.00	Donor 3	Day30	Unblocked	Qualified
Lymphocytes[1]	CD14neg	-25.61	1356.38	Donor 3	Day30	Unblocked	Non-Qualifed
Monocytes[1]	CD19-	-19.07	2890.18	Donor 3	Day30	Unblocked	Qualified
NK[1]	CD14neg	-23.59	2669.29	Donor 3	Day30	Unblocked	Qualified
Non-Classical Mono[1]	Monocytes	-22.25	2727.59	Donor 3	Day30	Unblocked	Qualified
PBMC[1]	Non-Neutrophils	-26.31	2081.55	Donor 3	Day30	Unblocked	Non-Qualifed
Bcell[1]	CD3neg	3.85	1733.94	Donor 3	Day60	Unblocked	Qualified
CD4+ T Cells[1]	T Cells	-1.78	1643.87	Donor 3	Day60	Unblocked	Qualified
CD8+ T Cells[1]	T Cells	-2.63	2011.91	Donor 3	Day60	Unblocked	Qualified
Classical Mono[1]	Monocytes	1.93	3748.94	Donor 3	Day60	Unblocked	Qualified
Intermediate Mono[1]	Monocytes	9.66	2771.99	Donor 3	Day60	Unblocked	Qualified
Lymphocytes[1]	CD14neg	-1.35	1798.63	Donor 3	Day60	Unblocked	Qualified
Monocytes[1]	CD19-	2.76	3669.74	Donor 3	Day60	Unblocked	Qualified
NK[1]	CD14neg	-0.15	3488.26	Donor 3	Day60	Unblocked	Qualified
Non-Classical Mono[1]	Monocytes	2.54	3597.14	Donor 3	Day60	Unblocked	Qualified
PBMC[1]	Non-Neutrophils	-3.17	2735.37	Donor 3	Day60	Unblocked	Qualified

[1]*W%D was determined based on MFI*

8. SUMMARY

This Qualification Report describes the flow cytometry assay that was qualified and reports on the precision of the assay. Based on the data observed, the assay performs reprodicibly in terms of intra-assay, inter-operator and inter-instrument comparisons across the cell populations and metrics assessed where the abundance of population is greater than 1% of viable leukocytes. Also cut-off time point was established based on precision experiments for blood sample processing after draw (no more than 3 days post draw) and for -80°C sample storage (no more than 30 days post freeze). The assay is therefore suitable for monitoring the phenotype of immune cell subsets for exploratory analyses of clinical trial samples.

9. REFERENCES

- E6 (R2) Good Clinical Practice: Integrated Addendum to ICH E6(R1) Guidance for Industry. March 2018

10. APPENDIX

10.1. WORKING INSTRUCTIONS

(Starting on next page)

CAPRION	*Document No.*	*Applicable SOP*
	**D-CSP-02-6102-FIX-v1.0**	**CSP-02-6102-v1.0**
	Lyse/Fix Procedure (precision experiments)	

**STUDY CODE:**	**6102**	**RUN ID:**	
**RUN OBJECTIVE:**	Precision experiment to describe how IRAK4 degradation assay performs		
**RUN ID/PROCESSING DATE (DDMMMYYYY)**			
**OPERATOR /ANALYST (print)**			

EQUIPMENT TYPE	EQUIPMENT ID	PIPETTES USED	PIPETTE ID
Biological Safety Cabinet		MULTI/P1000	
Centrifuge		MULTI/P200	
ELX Plate Aspirator		MULTI/P20	
Water Bath		Other	
VIAFLO			

REAGENTS AND PREPARATION OF WORKING SOLUTIONS:

ID	ASSIGNED INTERNAL ID (6102-YYMMDD-XX)	DONOR/SUBJECT ID	COLLECTION DATE
**D1**			
**D2**			
**D3**			

REAGENTS AND SOLUTIONS	LOT/BATCH NUMBER	EXPIRY DATE
2% FBS		
HyClone Water, Cell Culture Grade		
Lyse/Fix Buffer 5X		

Authenticated by (Initial/Date): ____________________

CAPRION	*Document No.*	*Applicable SOP*
	**D-CSP-02-6102-FIX-v1.0**	**CSP-02-6102-v1.0**
	Lyse/Fix Procedure (precision experiments)	

REAGENT	VOLUME PER SAMPLE (µL)	☐ 20 SAMPLES (PLAN FOR 25) (mL)	☐ 40 SAMPLES (PLAN FOR 50) (mL)	☐ # OF TUBES PREPARED
5X Lyse Fix	300	5 mL	10 mL	
Hyclone Water	1200	20 mL	40 mL	
**TOTAL:**	1500	25 mL	50 mL	

**PROCEDURE**		
**1X LYSE/FIX WORKING SOLUTION**		**Add information or Check only when/after step performed**
**STEP #**		
1	Mix by inversion and pre-warm for at least 30 minutes in a water batch set at 37°C	Start Time:<br><br>End Time:
**STEP #**	**SAMPLE STAINING**	**Check only when/after step performed**
1	Prepare equipment, required working solutions and retrieve samples as necessary.	**Start Time:**
2	Pipette **<u>100µL</u>** of whole blood sample in **<u>6 wells</u>** for a total of **<u>600µL per sample</u>** in a 96-deepwell conical (V) bottom plate or equivalent.<br>• Refer to worksheet for plate layout	☐
3	Lyse/Fix Samples<br>• Using a P1000 multichannel, pipette 1mL of 1x Lyse/Fix Working solution to each well<br>• Re-suspend gently by pipetting up and down one row at a time	☐
4	Seal and Incubate the plate at RT for 10 minutes	**Actual Incubation Time:** __________
5	Centrifuge Sample:<br>• Centrifuge samples at 400g (1500 RPM) for 5 minutes at RT;	☐

Authenticated by (Initial/Date): ____________________

6	Aspirate Cells<br>• Using a VIAFLO equipped with a 1250µL head, aspirate the supernatant into another deep well block using “WASTE” program.	☐
7	Pipette 1mL of 2% FBS per well<br>• Using a P1000 multichannel, pipette 1mL of 2% FBS to each well. Gently pipette to mix.	☐
8	Centrifuge Sample:<br>• Seal sample and centrifuge at 400g (1500 RPM) for 5 minutes at RT;	☐
9	Aspirate Cells:<br>• Using a VIAFLO equipped with a 1250µL head, aspirate the supernatant into another deep well block using “XXX” program.	☐
10	Transfer cells to Armadillo PCR plate:<br>• Using a P200 Multichannel, transfer 3 full wells to the “Primary” plate<br>• Use a P200 Multichannel to transfer the 3 remaining cells to the “Backup Plate”;<br>• Use a P200 Multichannel to transfer the 3 remaining cells to the “Backup Plate”;	☐
11	Centrifuge Samples:<br>• Centrifuge the **two** armadillo plates at 400g (1500 RPM) for 5 minutes at RT;	☐
12	Aspirate Cells:<br>• Use PCR ELX aspirator equipped with “PCR HI” to aspirate cells;	☐
13	Fill In Plate Layout on Page 4<br>• Fill in Subject + Timepoint (or	☐
14	Label Each Plate:<br>• Use a label maker to label each plate;<br>• Primary Plate: **6102-YYMMDD-PRIMARY**<br>• Backup Plate: **6102-YYMMDD-BACKUP**	☐<br>**End Time:**
15	Freeze immediately at -80°C and record location	**Freezer:**<br>**Location:**

Authenticated by (Initial/Date): ____________________

DocuSign Envelope ID: 8ECCE282-5174-484C-8E06-7271AEF7B4F6

PLATE LAYOUT: TABLE REPRESENTS BOTH PRIMARY AND BACKUP PLATES

	1	2	3	4	5	6	7	8	9	10	11	12
A												
B												
C												
D												
E												
F												
G												
H												

Notes

Reviewed by / Date:____

Authenticated by (Initial/Date): ____________________

CAPRION	*Document No.*	*Applicable SOP*
	**CSP-01-6102-ABS-v1.0**	**CSP-01-6102-v1.0**
	Pre-Perm and Post-Perm Antibody Cocktail Preparation	

**RUN ID/PROCESSING DATE (DDMMMYYYY)**	
**OPERATOR /ANALYST (print)**	

PREPARATION OF "PRE-PERM" STAINING COCKTAIL

**Reagent**	**Volume per Stain (µL)**	**Antibody Lot**	**Antibody Expiry Date**	☐ **11 Stained Samples (Plan for 13) (µL)**	☐ **_____ Stained Samples (Plan for _____) (µL)**
BD BSA	71.5µL			929.5	
eBio BV Buffer	4µL			52	
CD14 BUV395	4µL	0335456	30SEP2022	52	
CD56 BV711	2µL	B327219	10NOV2023	26	
CD19 BV786	1µL	0335061	30NOV2021	13	
CD4 Ax700	0.5µL	B296061	31JUL2024	6.5	
CD3 Pacific Blue	0.5µL	B284971	31MAR2024	6.5	
CD8 FITC	0.5µL	B279938	31JAN2026	6.5	
CD15 PE-Cy7	0.5µL	B280000	31JAN2023	6.5	
CD45 BUV805	0.5µL	0290065	31MAR2022	6.5	
**Total Volume**	**85**			**1105**	

PREPARATION OF "POST-PERM" STAINING COCKTAIL

**Reagent**	**Volume per Stain (µL)**	**Antibody Lot**	**Antibody Expiry Date**	☐ **11 Stained Samples (Plan for 13) (µL)**	☐ **_____ Stained Samples (Plan for _____) (µL)**
BD BSA	39.5µL			513.5µL	
IRAK4	5µL	9140819	31DEC2022	65µL	
CD16 PE	0.5µL	B317475	18MAY2025	6.5µL	
**Total Volume**	**45µL**			**585µL**	

Authenticated by / Date:_______________________________________

Reviewed by / Date:____

CAPRION	*Document No.*	*Applicable SOP*
	**CSP-02-6102-FRM-v1.0**	**CSP-02-6102-v1.0**
	Assay Form: Staining for IRAK4 Degradation Assay in Fixed/Frozen Human Whole Blood Samples (Precision Experiments)	

**STUDY CODE:**	**6102**
**RUN OBJECTIVE:**	Precision experiment to evaluate performance of the IRAK4 degradation assay
**RUN ID/PROCESSING DATE (DDMMMYYYY)**	
**OPERATOR /ANALYST (print)**	

SAMPLE INFORMATION:

PLATE ID OF THAWED SAMPLES	-80°C FREEZER + LOCATION

Clinical samples will be processed according to the following plan:

SAMPLE TYPE	STAINING CONDITIONS	TOTAL NUMBER OF SAMPLES
Clinical Lyse/Fixed Frozen Blood Samples	Full panel staining for each Lysed/Fixed donor sample	
Clinical Lyse/Fixed Frozen Blood Sample	Additional “Blocked” Condition for each Lysed/Fixed donor sample	
	**TOTAL STAINS**	

EQUIPMENT USED:

EQUIPMENT TYPE	EQUIPMENT ID	PIPETTES USED	PIPETTE ID
Flow Cytometer	☐ FORTESSA ☐ KAPPA	MULTI/P1000	
Centrifuge, refrigerated		MULTI/P200	
Laboratory Refrigerator 2-8°C		MULTI/P20	
Plate Shaker		MULTI/P2	
ELX Plate Aspirator			

REAGENTS AND PREPARATION OF WORKING SOLUTIONS:

Authenticated by (Initial/Date): ___________________

REAGENTS AND SOLUTIONS	LOT/BATCH NUMBER	EXPIRY DATE
BD BSA		
100% Methanol		
HyClone Water, Cell Culture Grade		
Brillant Stain Buffer (BSB)		
1X Lyse/Fix		
Hu IRAK4 Unconjugated Antibody		

60% MeOH Solution: Dilute the 100% Methanol stock by preparing a working solution in a conical tube. Store at 4°C until use.

REAGENT	VOLUME PER SAMPLE (µL)	☐ 40 SAMPLES (PLAN FOR 50) (mL)	☐ 80 SAMPLES (PLAN FOR 100) (mL)	☐ _____ SAMPLES (µL)
100% Methanol	90	4.5 mL	9 mL	
Hyclone Water	60	3 mL	6 mL	
**TOTAL:**	150	7.5 mL	15 mL	

PROCEDURE

STEP #	PREPARATION OF WORKING SOLUTIONS, ANTIBODY COCKTAIL, AND COMPENSATION CONTROLS	Check only when/after step performed
1	Prepare required working solution (60% Methanol).	**Start Time:**
2	Prepare Full Panel Pre-perm Staining Cocktail as listed in CSP-01-6102-ABS.	☐
**STEP #**	**SAMPLE STAINING**	**Check only when/after step performed**
1	Remove Armadillo PCR plate samples from -80°C freezer and thaw at RT for 5 minutes.	**Start Time:**<br>**Actual Thaw Time (min):**
2	Resuspend samples in BD BSA:<br>• Use a P200 multi-channel to resuspend samples in 85µl of BD BSA.<br>• Pipette to mix.	☐

Authenticated by (Initial/Date): ____________________

3	Transfer cells to a new PCR plate for staining Blocked and Unblocked conditions (**one well per condition**).<br>• Transfer 45µL of cells to a 96-well PCR plate for staining "**Blocked**" samples.<br>• Transfer 45µL of cells to a 96-well PCR plate for staining **"Unblocked**" samples.<br>• Add 100µL of BD BSA to all wells.	☐
4	Centrifuge Samples:<br>• Centrifuge samples at 400g (1500 RPM) for 5 minutes at RT.<br>• Use PCR ELX aspirator equipped with "PCR HI" to aspirate supernatant.<br>• Use a plate shaker to resuspend cells in residual volume (1800rpm x 15 seconds).	☐
5	Stain cells with Pre-perm Antibody Cocktail:<br>• Using a P200 multichannel, pipette 85µL/well of antibody cocktail to each row of samples and pipette to mix.	☐
6	Seal plate and incubate at RT <u>in the dark</u> for 30 minutes.	**Actual Incubation Time:**
7	Centrifuge Samples:<br>• Centrifuge samples at 400g (1500 RPM) for 5 minutes at RT.<br>• Use PCR ELX aspirator equipped with "PCR HI" to aspirate supernatant.<br>• Use a plate shaker to resuspend cells in residual volume (1800rpm x 15 seconds).	☐
8	Wash cells in BD BSA<br>• Using a P200 multi-channel, add 150µL / well.	☐
9	Centrifuge Samples:<br>• Centrifuge samples at 400g (1500 RPM) for 5 minutes at RT.<br>• Use PCR ELX aspirator equipped with "PCR HI" to aspirate supernatant.<br>• Use a plate shaker to resuspend cells in residual volume (1800rpm x 15 seconds).	☐
10	Wash cells in BD BSA<br>• Using a P200 multi-channel, add 150µL / well.	☐
11	Centrifuge Samples:<br>• Centrifuge samples at 400g (1500 RPM) for 5 minutes at RT.<br>• Use PCR ELX aspirator equipped with "PCR HI" to aspirate supernatant.<br>• Use a plate shaker to resuspend cells in residual volume (1800rpm x 15 seconds).	☐
12	Permeabilize cells in 60% MeOH solution:<br>• Use a P200 multi-channel to add 150µL of 60% MeOH.<br>• Pipette to mix.	☐
13	Incubate in 4°C refrigerator for 10 minutes.	**Actual Incubation Time:**
14	Prepare Post-perm Staining Cocktail as listed in CSP-01-6102-ABS.	☐

Authenticated by (Initial/Date): ____________________

15	Centrifuge Samples:<br>• Centrifuge samples at 400g (1500 RPM) for 5 minutes at RT.<br>• Use PCR ELX aspirator equipped with "PCR HI" to aspirate supernatant.<br>• Use a plate shaker to resuspend cells in residual volume (1800rpm x 15 seconds).	☐
16	Wash cells in BD BSA<br>• Using a P200 multi-channel, add 150µL / well.	☐
17	Centrifuge Samples:<br>• Centrifuge samples at 400g (1500 RPM) for 5 minutes at RT.<br>• Use PCR ELX aspirator equipped with "PCR HI" to aspirate supernatant.<br>• Use a plate shaker to resuspend cells in residual volume (1800rpm x 15 seconds).	☐
18	Wash cells in BD BSA<br>• Using a P200 multi-channel, add 150µL / well.	☐
19	Centrifuge Samples:<br>• Centrifuge samples at 400g (1500 RPM) for 5 minutes at RT.<br>• Use PCR ELX aspirator equipped with "PCR HI" to aspirate supernatant.<br>• Use a plate shaker to resuspend cells in residual volume (1800rpm x 15 seconds).	☐
20	Wash cells in BD BSA<br>• Using a P200 multi-channel, add 150µL / well.	☐
21	Centrifuge Samples:<br>• Centrifuge samples at 400g (1500 RPM) for 5 minutes at RT.<br>• Use PCR ELX aspirator equipped with "PCR HI" to aspirate supernatant.<br>• Use a plate shaker to resuspend cells in residual volume (1800rpm x 15 seconds).	☐
22	Add the IRAK4 blocking antibody:<br>• Dispense 40µL/well of Hu IRAK4 unconjugated antibody (undiluted stock) and pipette to mix in each **"Blocked"** well.<br>• Dispense 40µL/well of BD BSA and pipette to mix in each **"Unblocked"** well.	☒
23	Incubate at RT <u>in the dark</u> for 30 minutes.	**Actual Incubation Time:**
24	Stain Cells with Post-perm antibody cocktail:<br>• Use a P200 multi-channel to add 45µL/well of post-perm antibody cocktail to all wells.	☐
25	Incubate at RT <u>in the dark</u> for 30 minutes.	**Actual Incubation Time:**
26	Centrifuge Samples:<br>• Centrifuge samples at 400g (1500 RPM) for 5 minutes at RT.<br>• Use PCR ELX aspirator equipped with "PCR HI" to aspirate supernatant.<br>• Use a plate shaker to resuspend cells in residual volume (1800rpm x 15 seconds).	☐
27	Wash cells in BD BSA<br>• Using a P200 multi-channel, add 150µL / well.	☐

Authenticated by (Initial/Date): ____________________

DocuSign Envelope ID: 8ECCE282-5174-484C-8E06-7271AEF7B4F6

28	Centrifuge Samples:<br>• Centrifuge samples at 400g (1500 RPM) for 5 minutes at RT.<br>• Use PCR ELX aspirator equipped with "PCR HI" to aspirate supernatant.<br>• Use a plate shaker to resuspend cells in residual volume (1800rpm x 15 seconds).	☐
29	Wash cells in BD BSA<br>• Using a P200 multi-channel, add 150µL / well.	☐
30	Centrifuge Samples:<br>• Centrifuge samples at 400g (1500 RPM) for 5 minutes at RT.<br>• Use PCR ELX aspirator equipped with "PCR HI" to aspirate supernatant.<br>• Use a plate shaker to resuspend cells in residual volume (1800rpm x 15 seconds).	☐
31	Sample Resuspension:<br>• Use a multi-channel to add 50µL of BD BSA to each well.<br>• Pipette to mix, transfer the entire contents of each well to corresponding alpha-numeric printed cluster tube.	☐
32	Store samples in 4°C refrigerator until analysis.	**Stain Completion Time:**
33	Fill in plate layout on Page 6.	
**STEP #**	**DATA ACQUISITION**	**Check only when/after step performed**
1	Set up flow cytometer for data acquisition:<br>• Use appropriate analysis template in BD FACSDiva.<br>• Confirm that CS&T passed, and apply "6102_IRAK4_v210128" application settings.<br>• Click "No" on FSC Area Scaling overwrite pop-up window.<br>• Log out and in, and click "Use CST Settings" on pop-up window.<br>• Check Laser Settings against daily CS&T Cytometer Performance Report.	☐
2	Name experiment, and label specimens and tubes for samples in Diva experiment layout. Ensure that labels are provided for all variables (e.g., donors, block conditions, replicates, and cytometers).	☐
3	Acquire data:<br>• *IMPORTANT*: Vortex to resuspend each sample prior to acquisition;<br>• Acquire full volume from each sample unless otherwise instructed by Technical Supervisor.	**Data Acquisition Start Time:**
**STEP #**	**DATA EXPORT AND UPLOAD**	**Check only when/after step performed**
1	Export data:<br>• Export FCS files including all parameters to the Clinical Samples folder, under the appropriate Cytometer_Raw_Data directory.	☐

Authenticated by (Initial/Date): ____________________

PLATE LAYOUT FOR DONORS

	1	2	3	4	5	6	7	8	9	10	11	12
A												
B												
C												
D												
E												
F												
G												
H												

PLATE LAYOUT FOR BLOCKED + UNBLOCKED CONDITIONS

	1	2	3	4	5	6	7	8	9	10	11	12
A												
B												
C												
D												
E												
F												
G												
H												

Authenticated by (Initial/Date): ____________________

**Notes**

Reviewed by / Date:____

Authenticated by (Initial/Date): ____________________

Gating Strategy for PBMC population from whole blood samples

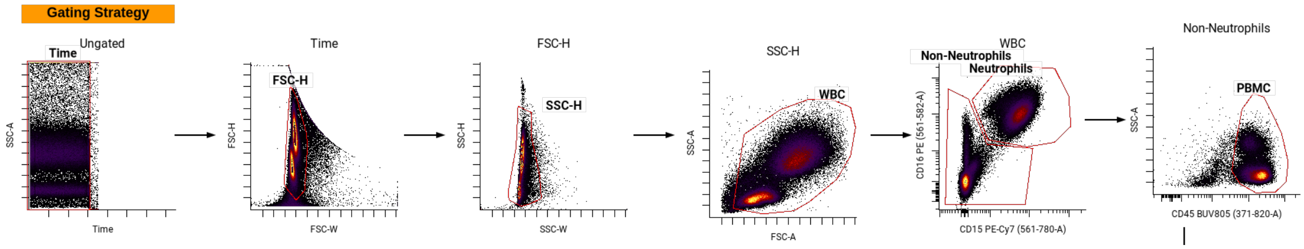

CLINICAL TRIAL PROTOCOL

A Phase 1 randomized, placebo-controlled, single and multiple ascending dose trial to evaluate the safety, tolerability, pharmacokinetics, and pharmacodynamics of orally administered KT-474 in healthy adult volunteers and patients with atopic dermatitis (AD) or hidradenitis suppurativa (HS)

Investigational Product: KT-474

Protocol Number: KT474-HV-101

Original Protocol:	Version 1.0, 11 December 2020
	Version 2.0, 18 February 2021
	Version 3.0, 15 March 2021
	Version 4.0, 20 May 2021
	Version 5.0, 30 July 2021
	Version 6.0, 13 September 2021
	Version 7.0, 04 April 2022
	Version 8.0, 10 June 2022
	**Version 9.0, 05 August 2022**

Sponsor:

Kymera Therapeutics, Inc.
200 Arsenal Yards Blvd., Suite 230
Watertown, MA 02472

US IND Number: IND 149184

Confidentiality Statement

The information in this document is confidential and is not to be disclosed without the written consent of Kymera Therapeutics, Inc. except to the extent that disclosure would be required by law and for the purpose of evaluating and/or conducting a clinical trial for Kymera Therapeutics, Inc. You are allowed to disclose the contents of this document only to your Institutional Review Board or Ethics Committee and study personnel directly involved with conducting this protocol. Persons to whom the information is disclosed must be informed that the information is confidential and proprietary to Kymera Therapeutics, Inc. and that it may not be further disclosed to third parties.

SIGNATURE PAGE

Study Title: A Phase 1 randomized, placebo-controlled, single and multiple ascending dose trial to evaluate the safety, tolerability, pharmacokinetics, and pharmacodynamics of orally administered KT-474 in healthy adult volunteers and patients with atopic dermatitis (AD) or hidradenitis suppurativa (HS)

Sponsor Signatory:

[redacted]

Medical Monitor name and contact information can be found in Appendix 2.

TABLE OF CONTENTS

TABLE OF TABLES.....7

TABLE OF FIGURES.....8

1.0 PROTOCOL SUMMARY.....9
1.1 Synopsis.....9
1.2 Schema.....15
1.3 Schedule of Planned Assessments.....16

2.0 INTRODUCTION.....37
2.1 Heterobifunctional Small Molecule Protein Degraders.....37
2.2 Interleukin-1 Receptor-associated Kinase-4 (IRAK4).....37
2.2.1 Biology and Role in Inflammation.....37
2.2.2 Phenotype of IRAK4 Deficiency and Inhibition.....38
2.3 Rationale for an IRAK4 Degrader for the Treatment of Inflammatory Cutaneous Diseases.....38
2.3.1 Hidradenitis Suppurativa.....38
2.3.2 Atopic Dermatitis.....39
2.4 KT-474.....40
2.4.1 Nonclinical Pharmacology Studies with KT-474.....40
2.4.2 Nonclinical Pharmacokinetic Studies with KT-474.....41
2.4.3 Nonclinical Toxicity and Safety Studies with KT-474.....42
2.5 Benefit/Risk Assessment.....43

3.0 OBJECTIVES AND ENDPOINTS.....45
3.1 Study Objectives.....45
3.1.1 Primary Objective:.....45
3.1.2 Secondary Objectives:.....45
3.1.3 Exploratory Objectives:.....45
3.2 Study Endpoints.....45
3.2.1 Primary Endpoints:.....45
3.2.2 Secondary Endpoints.....46
3.2.3 Exploratory Endpoints:.....46

4.0 STUDY DESIGN.....48
4.1 Part A.....48
4.1.1 Food-effect.....49
4.1.2 Additional Non-Randomized Fed-State Cohorts.....49
4.2 Part B.....50
4.2.1 Additional Intermittent Dosing Cohorts.....51
4.3 Part C.....52
4.4 Selection of Doses.....52

4.4.1 Selection of First in Human Starting Dose 52
4.4.2 Part A 53
4.4.3 Part B 54
4.4.4 Part C 55
4.5 Study Procedures During Special Circumstances 55
4.6 Dose Escalation Criteria 56
4.7 Safety Review Committee, Dose Escalation Procedures and Stopping Criteria (SAD and MAD) 56
4.7.1 Data Required for Dose Escalation Review 56
4.8 Stopping Criteria 57
4.8.1 Individual Study Participant Stopping Criteria 57
4.8.2 Dose Escalation Stopping Criteria 57
4.8.3 Criteria for Stopping the Study (Parts A, B, and C) 58
4.9 End of Study Definition 58

5.0 STUDY POPULATION 59
5.1 Inclusion Criteria 59
5.1.1 For Healthy Volunteers (Parts A and B) 59
5.1.2 For Patients (Part C) 60
5.2 Exclusion Criteria 61
5.2.1 For Healthy Volunteers (Parts A and B) 61
5.2.2 For Patients (Part C) 63
5.3 Lifestyle Considerations 66
5.3.1 Meals and Dietary Restrictions 66
5.3.2 Caffeine and Alcohol 68
5.3.3 Activity 68
5.4 Screen Failures 68

6.0 STUDY ASSESSMENTS AND PROCEDURES 69
6.1 Efficacy Assessments 69
6.1.1 HS Patients 69
6.1.2 AD Patients 70
6.2 Safety Assessments 71
6.2.1 Physical Examinations 71
6.2.2 Vital Signs 71
6.2.3 Holter Monitoring 71
6.2.4 Telemetry 72
6.2.5 Electrocardiograms in Parts A and B 72
6.2.6 Electrocardiogram Monitoring and Dose Modifications in the Intermittent Dosing Cohorts and in Part C 73
6.2.7 Clinical Safety Laboratory Assessments 75
6.3 Adverse Events 76
6.3.1 Time Period and Frequency for Collecting Adverse Event and Serious Adverse Event Information 76
6.3.2 Method of Detecting Adverse Events and Serious Adverse Events 77

6.3.3 Follow-up of Adverse Events and Serious Adverse Events.............77
6.3.4 Regulatory Reporting Requirements for Serious Adverse Events.............77
6.3.5 Adverse Events of Special Interest.............78
6.3.6 Pregnancy.............78
6.4 Treatment of Overdose.............78
6.5 Pharmacokinetics.............79
6.5.1 Collection of Samples.............79
6.5.2 Determination of Drug Concentration.............80
6.5.3 Calculation of Derivation of Pharmacokinetic Variables.............80
6.6 Pharmacodynamics.............84
6.7 Tissue Pharmacokinetics.............86
6.8 Genetics.............87
6.9 Health Economics OR Medical Resource Utilization and Health Economics.............87

7.0 TREATMENT AND RESTRICTIONS.............88
7.1 Treatment Regimen, Dosing, Duration.............88
7.1.1 SAD Cohorts (Part A).............88
7.1.2 Food Effect Cohorts (Part A).............88
7.1.3 Additional Non-randomized Cohorts (Part A).............88
7.1.4 MAD Cohorts (Part B).............89
7.1.5 Additional Intermittent Dosing Cohorts (Part B).............89
7.1.6 Patient Cohort (Part C).............89
7.2 Measures to Minimize Bias: Randomization and Blinding.............89
7.3 Investigational Product Compliance.............90
7.3.1 Treatment Strategy.............91
7.4 Study Restrictions.............91
7.4.1 Concomitant Therapy.............91

8.0 INVESTIGATIONAL PRODUCTS.............93
8.1 Clinical Trial Material.............93
8.2 Pharmaceutical Formulations.............93
8.3 Labeling and Packaging.............93
8.4 Dispensing Procedures and Storage Conditions.............94
8.4.1 Dispensing Procedures.............94
8.4.2 Storage Conditions.............94

9.0 DISCONTINUATION OF INVESTIGATIONAL PRODUCT AND DISCONTINUATION/WITHDRAWAL OF THE STUDY PARTICIPANT.............95
9.1 Discontinuation of Investigational product.............95
9.2 Discontinuation/Withdrawal of Study Participant from the Study.............96
9.3 Lost to Follow-up.............96

10.0 STATISTICAL CONSIDERATIONS 97
10.1 Sample Size Determination 97
10.2 Populations for Analyses 97
10.3 Statistical Analyses....97
10.3.1 Efficacy Analyses....98
10.3.2 Pharmacokinetic Analyses....98
10.3.3 Pharmacodynamic Analyses....99
10.3.4 Tissue Pharmacokinetic Analysis....99
10.3.5 Safety Analyses 100
10.3.6 Missing Data....100
10.4 Interim Analyses 100

11.0 REFERENCES....101

12.0 APPENDICES 103
Appendix 1 Abbreviations....104
Appendix 2 Regulatory, Ethical, and Study Oversight Considerations....108
Regulatory and Ethical Considerations....108
Adequate Resources....108
Financial Disclosure....109
Insurance 109
Informed Consent Process 109
Data Protection....109
Administrative Structure....110
Medical Monitor 110
Dissemination of Clinical Study Data....110
Data Quality Assurance 110
Source Documents 111
Study and Study Center Closure....111
Publication Policy 111
Appendix 3 Clinical Laboratory Tests 112
Appendix 4 Adverse Events: Definitions and Procedures for Recording, Evaluating, Follow-up, and Reporting....114
Appendix 5 Excluded Medications/Therapy 120
Appendix 6 Contraceptive Guidance and Collection of Pregnancy Information....122
Appendix 7 Food Effect Meal Composition....125
Appendix 8 Signature of Investigator 126

TABLE OF TABLES

Table 1 Schedule of Planned Assessments for Part A-Single Ascending Dose Cohorts 16
Table 2 Schedule of Planned Assessments for Part A- Single Ascending Dose (Food Effect) Cohorts 19
Table 3 Schedule of Planned Assessments for Part A – Additional Non-Randomized Fed-State Cohorts 22
Table 4 Schedule of Planned Assessments for Part B-Multiple Ascending Dose Cohorts 25
Table 5 Schedule of Planned Assessments for Part C AD/HS Cohort 28
Table 6 Schedule of Planned Assessments for Part B - Intermittent Dosing – Every other day (QOD) 31
Table 7 Schedule of Planned Assessments for Part B - Intermittent Dosing – Twice Weekly (BIW) 34
Table 8 Pharmacologically Active Concentrations of KT-474 in *In Vitro* and *In Vivo* Studies 53
Table 9 Exposure Ratios Predicted for KT-474 in Human Relative to Rats and Dogs NOAELs from 28-day Toxicology Studies 54
Table 10 Dose Modifications for Part C Patients for QTcF Prolongation as Determined by the Core ECG Laboratory 74
Table 11 Plasma Pharmacokinetic Parameters 82
Table 12 Urine Pharmacokinetic Parameters 83
Table 13 Details Related to Blood Samples - Part A (Single Ascending Dose and Food Effect- Healthy Volunteers) 84
Table 14 Details Related to Blood and Skin Samples - Part B (Multiple Ascending Dose - Healthy Volunteers) 84
Table 15 Details Related to Blood and Skin Samples - Part B (QOD Intermittent Dosing - Healthy Volunteers) 85
Table 16 Details Related to Blood and Skin Samples - Part B (BIW Intermittent Dosing - Healthy Volunteers) 85
Table 17 Details Related to Blood and Skin Samples - Part C (Multiple Ascending Dose – Patients with HS or AD) 86
Table 18 Analysis Sets 97
Table 19 Protocol-required Safety Laboratory Assessments 112
Table 20 Allowed and Excluded Medications 121

TABLE OF FIGURES

Figure 1 Study Schema..15

1.0 PROTOCOL SUMMARY

1.1 Synopsis

**Protocol Title**: A Phase 1 randomized, placebo-controlled, single and multiple ascending dose trial to evaluate the safety, tolerability, pharmacokinetics, and pharmacodynamics of orally administered KT-474 in healthy adult volunteers and patients with atopic dermatitis (AD) or hidradenitis suppurativa (HS)
**Protocol Number**: KT474-HV-101
**Investigational Product**: KT-474
**Phase**: 1
**Objectives**: To assess the safety, tolerability, pharmacokinetics (PK), and pharmacodynamics (PD) of KT-474 after administering single and multiple oral doses at escalating dose levels in healthy volunteers (HVs) and following multiple doses in patients with AD or HS.
**Overview of Study Design:** This is a first in human (FIH), Phase 1 study of KT-474 that will characterize the safety, PK, and PD of KT-474 after a single dose and after repeated dosing in adult HVs and in patients with HS or AD. Initially, a dose range of KT-474 in single ascending dose (SAD) escalation cohorts will be explored in adult HVs (Part A). To understand food effects (FE) on the PK and PD of KT-474 in HVs, up to 2 SAD cohorts will be designated in Part A where HVs will return for a second treatment period and will receive the same treatment which was originally allocated, but in the fed state. Safety and PK data from at least 3 completed SAD cohorts will determine initiation of and appropriate doses for the 14-day multiple ascending dose (MAD) portion of the study (Part B). A single cohort of up to 30 patients with AD or HS will be subsequently enrolled (Part C) and KT-474 will be administered to these patients for 28 days, at a dose and schedule selected by the Safety Review Committee (SRC) following review of the safety, PK, and PD data after completion of the dose escalation in Part B.<br><u>Part A</u><br>Part A is a double-blind, randomized, placebo-controlled, SAD, sequential group study in approximately 64 adult HVs, divided in 8 cohorts of eight HVs each. Seven ascending single doses (1 dose level per cohort) will be investigated. One or more additional cohorts may be added in the presence of food or in the fasting state, as needed, after SRC review of all relevant clinical safety, and PK data. Within each cohort, 6 HVs will be randomized to receive KT-474 and 2 HVs will be randomized to receive placebo.<br>In the SAD part, the planned KT-474 doses are 25, 75, 150, 300, 600, 1000, and 1400 mg. Pharmacokinetic parameters at the no-observed-adverse-effect level (NOAELs) from the 28-day KT-474 toxicokinetic studies in rats and dogs were used to calculate exposure ratios relative to predicted human AUC and C_{max} for KT-474. These data indicate 79- to 159-fold exposure safety margin for the starting dose of 25 mg based on the AUCs of rat and dog NOAELs, respectively. The safety margin decreases as the dose increases. Following review of safety and PK data from HVs in the 25 mg dose cohort, dose levels of subsequent SAD cohorts may be adjusted from those proposed but will not exceed the designated fold increase of exposure indicated for each dose level. The mean exposure in SAD will not exceed the mean AUC of 11,700 ng*hr/mL, which is the AUCτ on Day-28 at the NOAEL of 60 mg/kg/day in dogs, the most sensitive species.<br>At each dose level, 2 sentinel HVs (1 receiving KT-474 and 1 receiving placebo) will be administered the investigational product first. The safety data up to 24-hours post-dose for these sentinel HVs will be reviewed by the Investigator to ensure acceptable tolerability before commencing administration of the

investigational product to the remaining HVs in the cohort. Sequential dosing of HVs within a cohort will be staggered so that there will be at least a gap of 10 minutes between dosing of individual HVs.

After the completion of each dose level, the blinded interim PK data through Day 5 and safety data through Day 14 will be reviewed by the SRC before proceeding to the next dose level. Each subsequent dose administration will be performed, if in the judgment of the Investigator and Safety Physician, the results of the safety analyses of the preceding dose administration are satisfactory.

In addition, the effect of food intake on the PK of KT-474 will also be explored by selecting up to 2 SAD cohorts who will return for a second treatment period and will receive the same treatment allocation, in the fed state (within 30 minutes of completion of the FDA standard high-fat breakfast). The washout period between the first treatment and second treatment will be 14 days or 5 times of KT-474 half-life, whichever is longer. Selection of cohorts will be based on the emerging safety and PK data from previous cohorts in Part A. The anticipated exposures in the FE study will not exceed the highest anticipated exposures in the next planned SAD study cohort where safety and tolerability of KT-474 was established (eg, SAD 5 exposures in a fed state will not exceed SAD 6 projected exposures in the fasted state).

Following completion of Part B, up to 3 additional non-randomized cohorts may be initiated to establish the Part C dose in a fed state. In these additional cohorts, all 8 HVs will receive a single dose of KT-474 in the fed state. The doses of KT-474 to be evaluated in the additional cohorts will be selected such that predicted exposures in the fed state do not exceed the highest exposure achieved in Part A.

HVs in all cohorts will be screened for eligibility to participate in the study up to 26 days (Day −28) prior to admission to the study center on Day −2. Eligible HVs will be admitted to the study center on Day −2 and will be discharged on Day 5 after all scheduled assessments have been completed. Following discharge, HVs will return to the study center for follow-up visits on Days 7, 10, and 14.

Part B

Part B is a double-blind, randomized, placebo-controlled, MAD, sequential group study in approximately 48 adult HVs, divided in up to 4 cohorts of 12 adult HVs each. One or more additional cohorts may be added, as needed.

The MAD portion of the study will evaluate up to 4 dose levels of KT-474 continuous once daily dosing for 14 days. The selection of KT-474 doses will be guided by the safety, tolerability, and PK data in humans from the SAD portions of the study. The initial dose level of the first MAD cohort will be 25 mg. This dose has been selected, based on the PK observed in the first 3 SAD cohorts and is predicted to result in mean $ssAUC_{\tau}$ and ssC_{max} that are lower than the mean exposure observed in SAD cohort 3 (150 mg) where KT-474 was confirmed to be safe and tolerable. Increasing dose levels in subsequent MAD cohorts are planned to be 50, 100, 200, and 400 mg, and will be confirmed/modified based on the safety and PK observed in the previous SAD and MAD cohorts. Dose escalation between each MAD cohort will not exceed 100% of the mean exposure observed in the preceding MAD cohort. Doses greater than 400 mg may be evaluated provided the predicted mean daily exposure at the highest dose MAD cohort does not exceed the highest mean exposure in the SAD study where safety and tolerability of KT-474 was established.

Within each cohort, 9 HVs will be randomized to receive KT-474 and 3 HVs will be randomized to receive placebo.

It is planned that KT-474 or placebo will be administered orally once a day following an overnight fast for 10 hours, from Day 1 to Day 14, inclusive. However, the dosing interval and the duration of dosing may change following review of the safety, PK, and PD data from Part A.

Up to 3 additional cohorts may be initiated to evaluate a KT-474 dose administered once every other day (QOD) for a total of 7 doses over 14 days, and/or twice weekly (BIW) for a total of 5 doses over 15 days. In these cohorts, KT-474 or placebo will be administered in the fasted state and the dose(s) will

be selected such that predicted exposures do not exceed the highest exposure previously achieved in the study.

Within each cohort, 9 HVs will be randomized to receive KT-474 and 3 HVs will be randomized to receive placebo.

As a precaution, Part A of the study will utilize a sentinel dosing strategy. This strategy will not be utilized in Part B, unless the safety and PK data from Part A indicates otherwise (eg, safety issue). After the completion of each MAD dose level, PD data through day 7, PK data through Day 15 and safety data through Day 28 will be reviewed by the SRC before proceeding to the next dose level. Following review of the emerging safety, PK, and PD data from the first 2 MAD cohorts, this period for review may change either way, subject to a protocol amendment.

The HVs will be screened for eligibility to participate in the study up to 26 days (Day −28) prior to admission to the study center on Day −2. Eligible HVs will be admitted to the study center on Day −2 and will be discharged on Day 21 after all scheduled assessments have been completed. Following discharge on Day 21, HVs will return to the study center for a follow-up visit on Day 28. Additional visits may be planned following review of the emerging safety, PK, and PD data.

<u>Part C</u>

Part C is an open-label, multiple dose study of KT-474 administered daily for 28 days in a single cohort of up to 30 patients with AD or HS and will commence once the SRC has recommended a dose based on the review of Part B data. Part C may be conducted on an outpatient basis and patients will continue to be followed for safety through Day 42. The dose will be selected by the SRC following review of the safety, PK, and PD data after completion of Part B and based on evaluation of the PK of KT-474 in fed-state in Part A, and will result in plasma exposure that is approximately equivalent to that achieved with the 100 mg QD dose in the fasted state in Part B.

It is currently planned that the patients will be screened for eligibility from Day −28 and those eligible to participate will be treated on Day 1. Patients will return to the clinic on Days 4, 7, 11, 14, 21, 28, 31, 35, and 42. At the discretion of the investigator, patients will be dispensed study drug to be administered at home between visits or patients will be asked to return to the clinic to receive their study medication. After the last planned dose on Day 28, patients will be followed for safety until Day 42.

Stopping rules based primarily on safety with considerations of emerging PK and PD findings are defined for individual study participants, individual dose cohorts, and the entire study.

Number of Investigators and Study Centers: Approximately 8 Investigators and study centers are expected to participate in this study.

Study Population and Number of Study Participants: The total number of study participants is dependent on the number of cohorts required to determine the minimum and maximum effective doses.

<u>Part A</u>: Approximately 220 HVs will be screened to achieve 64 HVs assigned to the investigational product in the randomized cohorts.

Following completion of Part B, up to 24 HVs will be enrolled in up to 3 additional non-randomized cohorts to establish the Part C dose in the fed state.

<u>Part B</u>: Approximately 200 HVs will be screened to achieve 48 HVs assigned to the investigational product in the MAD cohorts.

Approximately 150 HVs will be screened to achieve up to 36 HVs assigned to the investigational product in the additional intermittent dosing cohorts.

<u>Part C</u>: Approximately 90 patients with AD or HS will be screened to enroll up to 30 patients assigned to the investigational product.

Treatment Groups and Duration of Study: The 2 treatment groups were KT-474 group and the placebo group.

Part A: Screening (26 days), Confinement before treatment (2 days), Treatment (1 day), Confinement after treatment (5 days), and follow-up (13 days).

Part B: Screening (26 days), Confinement before treatment (2 days), Treatment (14 days), Confinement after treatment (7 days) and follow-up (7 days). Duration of treatment for Part B intermittent dosing cohorts will be 7 doses (over 14 days) for the QOD schedule or 5 doses (over 15 days) for the BIW schedule.

Part C: Screening (28 days), Treatment (28 days) and follow-up (14 days).

Study Objectives:

Primary Objective:

- To determine the safety and tolerability of KT-474 when administered as single and multiple oral doses at escalating dose levels in HVs and following multiple doses in patients with AD or HS

Secondary Objective:

- To characterize the PK profile of KT-474 and its diastereomers KT-5481 and KT-5482, following single and multiple doses of KT-474 in HVs and following multiple doses in patients with AD or HS

Exploratory Objectives:

- To characterize the PD profile of KT-474 following single and multiple doses in HVs and following multiple doses in patients with AD or HS.
- To characterize the concentration of KT-474 in skin following multiple doses in HVs and patients with AD or HS.
- [redacted]
- To evaluate the metabolite profile of KT-474 following multiple doses of KT-474 in HVs.
- To assess blood and skin for messenger ribonucleic acid (mRNA) for candidate biomarkers following multiple doses of KT-474 in HVs and patients with AD or HS.
- To assess preliminary efficacy in patients with AD and HS.

Study Endpoints:

Primary Endpoints:

- Treatment-emergent (serious) adverse events ([S]AEs)
- Concomitant medication
- Clinical laboratory tests
    - Hematology
    - Coagulation
    - Chemistry
    - Urinalysis and urine microscopy
- Vital signs
    - Pulse Rate (bpm)
    - Systolic blood pressure (BP) (mm Hg)

  - Diastolic BP (mm Hg)
  - Respiratory rate
  - Temperature
- Safety electrocardiogram and Holter monitoring
  - Heart Rate (bpm), PR, QRS, QT, QTcF

Secondary Endpoints:

- Pharmacokinetic evaluations in HVs and patients with AD or HS

  The following (but not limited to) plasma PK parameters of KT-474, KT-5481, and KT-5482 will be calculated as appropriate:
  - Area under the plasma concentration-time curve from time zero to infinity [$AUC_{(0-\infty)}$] (single dose only), area under the plasma concentration-time curve from time zero to last measurable concentration [$AUC_{(0-last)}$], area under the concentration-time curve during a dosing interval [$AUC_{(0-tau)}$], maximum observed concentration (C_{max}), time to C_{max} (t_{max}), apparent clearance (CL/F), apparent volume of distribution (Vz/F), terminal half-life ($t_{1/2}$), mean residence time (MRT), and dose-normalized AUC and C_{max}
  - Following repeat dosing only, accumulation ratios (RAUC, RC_{max}), average concentration over the dosing interval (C_{avg}), and concentration at the end of dose interval (C_{trough})
  - [redacted]

  The following (but not limited to) urine PK parameters of KT-474, KT-5481, and KT-5482 in SAD and MAD cohorts will be calculated as appropriate:
  - By-collection-interval and cumulative amount: of unchanged drug excreted in urine [$Ae_{(t1-t2)}$, $Ae_{(0-t)}$], fraction of unchanged drug [$fe_{(t1-t2)}$, $fe_{(0-t)}$]
  - Renal clearance (CL_R)

Exploratory Endpoints:

- Pharmacodynamic Endpoints
  - IRAK4 levels in whole blood by FLOW (Parts A, B, and C)
  - IRAK4 levels in peripheral blood mononuclear cells by mass spectrometry (MS) (Parts A, B, and C)
  - IRAK4 levels in skin punch biopsies by MS and immunofluorescence (Parts B and C)
  - Proinflammatory cytokines and chemokines in skin punch biopsies by MS and gene expression profiling (GEP) (Part B and C)
  - Proinflammatory cytokine and chemokine production following ex vivo stimulation of whole blood by Luminex (Parts A, B and C)
  - Plasma high-sensitivity C-reactive protein levels by Luminex (Parts B [not in intermittent dosing cohorts] and C)
  - Plasma serum amyloid A and proinflammatory cytokines which may include but are not limited to tumor necrosis factor-α, interleukin (IL)-6, IL-1β, IL-4 and IL-5 by Luminex and enzyme-linked immunosorbent assay (Part C only)
  - Changes in mRNA levels by RNAseq in whole blood (Parts B and C)
- Pharmacokinetic Endpoints

- [redacted].
- [redacted]
- KT-474 concentration in skin punch biopsies (Parts B and C)

- Efficacy Endpoints
  - Change from baseline in Total Abscess and Inflammatory Nodule (AN) Count, Pain Numerical Rating Scale (NRS), Peak pruritis NRS and HS Physician's Global Assessment (HS-PGA) at 2, 4, 5 and 6 weeks in HS patients.
  - Change from baseline in Eczema Area and Severity Index (EASI), Peak Pruritus Numerical Rating Scale (NRS) and Investigator Global Assessment (IGA) at 2, 4, 5 and 6 weeks in AD patients.

Statistical Methods:

Efficacy

Preliminary efficacy will be assessed in an exploratory manner in Part C.

Safety and Tolerability

All data will be fully listed. The reporting of the safety data of all study participants receiving at least 1 dose of KT-474 or placebo will include the incidence and type of AEs, plus absolute values and changes in BP, heart rate, temperature, clinical laboratory data, physical examination, neurological examination data, and 12-lead electrocardiogram data from pre-dose to post-dose timepoints.

Pharmacokinetics

Analysis of the PK data will be performed for all study participants receiving a dose of KT-474. Pharmacokinetic parameters of KT-474, KT-5481, and KT-5482 will be summarized, and descriptive statistics (including mean, median, standard deviation and coefficient of variation) will be generated for each dose group. The graphical assessment of dose proportionality will be performed for AUC and C_{max}. Relative bioavailability of food effect will be assessed based on AUC and C_{max}.

Pharmacodynamics

Pharmacodynamic analyses will be performed for all study participants receiving at least one dose of KT-474 or placebo. The analysis of IRAK4 levels and modulation of proinflammatory cytokine and chemokine assessments will be considered exploratory. A mixed effects Analysis of Variance model will be used to compare the on-treatment IRAK4 levels of active versus placebo. The baseline IRAK4 levels will be used as a covariate in the model. The placebo-treated study participants will be pooled across cohorts and used as a single treatment group for comparison to each active treatment group.

Safety Review Committee: Yes

1.2 Schema

Figure 1 Study Schema

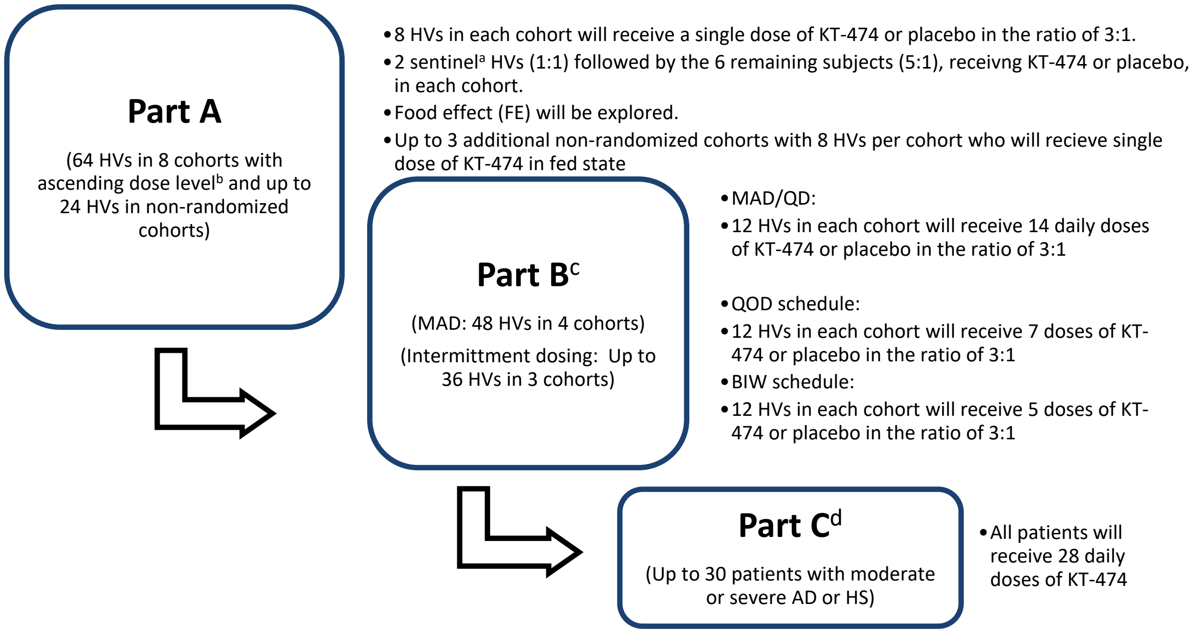

Abbreviations: AD = atopic dermatitis, BIW = twice weekly, HS = hidradenitis suppurativa, HV = healthy volunteer, MAD = multiple ascending dose, PK = pharmacokinetics, QD = daily dosing, QOD = every other day, SAD = single ascending dose, SRC = Safety Review Committee

a: The safety data up to 24-hours postdose for the sentinel study participants will be reviewed by the Investigator, before commencing administration of the investigational product to the remaining study participants in the cohort.

b: After the completion of each dose level, the safety data through Day 14 and PK data of KT-474 through Day 5 will be reviewed by an SRC before proceeding to the next dose level.

c: Safety and PK data from at least three completed SAD cohorts will determine initiation of and appropriate doses for Part B. After the completion of each dose level, the safety data through Day 28 and PK data of KT-474 through Day 15 will be reviewed by an SRC before proceeding to the next dose level. Following review of the emerging safety, PK and PD data from the first 2 MAD cohorts, this period for review may change either way subject to a protocol amendment.

d: Part C will commence once the SRC has recommended a dose based on the review of the Part B data.

1.3 Schedule of Planned Assessments

[redacted]

[redacted]	[redacted]	[redacted]	[redacted]	[redacted]	[redacted]	[redacted]	[redacted]	[redacted]	[redacted]	[redacted]	[redacted]
[redacted]	[redacted]										
[redacted]	[redacted]	[redacted]									
[redacted]	[redacted]										
[redacted]	[redacted]	[redacted]									
[redacted]	[redacted]										
[redacted]	[redacted]	[redacted]						[redacted]			[redacted]
[redacted]	[redacted]	[redacted]									[redacted]
[redacted]	[redacted]										
[redacted]	[redacted]	[redacted]		[redacted]	[redacted]			[redacted]			[redacted]
[redacted]	[redacted]	[redacted]		[redacted]	[redacted]			[redacted]			[redacted]
[redacted]	[redacted]										
[redacted]	[redacted]										
[redacted]	[redacted]	[redacted]									[redacted]
[redacted]	[redacted]	[redacted]									
[redacted]	[redacted]	[redacted]		[redacted]	[redacted]	[redacted]	[redacted]	[redacted]	[redacted]	[redacted]	[redacted]
[redacted]			[redacted]	[redacted]							
[redacted]				[redacted]							
[redacted]	[redacted]	[redacted]	[redacted]	[redacted]	[redacted]	[redacted]	[redacted]	[redacted]	[redacted]	[redacted]	[redacted]
[redacted]	[redacted]	[redacted]	[redacted]	[redacted]	[redacted]	[redacted]	[redacted]	[redacted]	[redacted]	[redacted]	[redacted]
[redacted]				[redacted]	[redacted]	[redacted]	[redacted]	[redacted]	[redacted]	[redacted]	[redacted]
[redacted]				[redacted]							
[redacted]				[redacted]							
[redacted]				[redacted]	[redacted]	[redacted]	[redacted]	[redacted]	[redacted]	[redacted]	[redacted]
[redacted]			[redacted]	[redacted]	[redacted]	[redacted]	[redacted]	[redacted]			

Time point [redacted]	[redacted]	[redacted]	[redacted]	[redacted]	[redacted]	[redacted]	[redacted]	[redacted]	[redacted]	[redacted]	[redacted]
[redacted]				[redacted]	[redacted]	[redacted]	[redacted]		[redacted]		[redacted]
[redacted]				[redacted]	[redacted]	[redacted]	[redacted]		[redacted]		[redacted]
[redacted]				[redacted]	[redacted]	[redacted]			[redacted]		[redacted]
[redacted]		[redacted]	[redacted]	[redacted]	[redacted]	[redacted]	[redacted]	[redacted]			
[redacted]		[redacted]									
[redacted]		[redacted]									
[redacted]		[redacted]									

[redacted]

j[redacted]

[redacted]

[redacted]		[redacted]									
	[redacted]	[redacted]	[redacted]	[redacted]	[redacted]	[redacted]	[redacted]	[redacted]	[redacted]	[redacted]	[redacted]
[redacted]	[redacted]										
[redacted]	[redacted]	[redacted]									
[redacted]	[redacted]										
[redacted]	[redacted]	[redacted]									
[redacted]	[redacted]										
[redacted]	[redacted]	[redacted]					[redacted]				[redacted]
[redacted]	[redacted]	[redacted]									[redacted]
[redacted]	[redacted]										
[redacted]	[redacted]	[redacted]		[redacted]	[redacted]			[redacted]			[redacted]
[redacted]	[redacted]	[redacted]		[redacted]	[redacted]			[redacted]			[redacted]
[redacted]	[redacted]										
[redacted]	[redacted]										
[redacted]	[redacted]	[redacted]									[redacted]
[redacted]	[redacted]	[redacted]									
[redacted]	[redacted]	[redacted]		[redacted]	[redacted]	[redacted]	[redacted]	[redacted]	[redacted]	[redacted]	[redacted]
[redacted]			[redacted]	[redacted]							
[redacted]				[redacted]							
[redacted]	[redacted]	[redacted]	[redacted]	[redacted]	[redacted]	[redacted]	[redacted]	[redacted]	[redacted]	[redacted]	[redacted]
[redacted]	[redacted]	[redacted]	[redacted]	[redacted]	[redacted]	[redacted]	[redacted]	[redacted]	[redacted]	[redacted]	[redacted]
[redacted]				[redacted]	[redacted]	[redacted]	[redacted]	[redacted]	[redacted]	[redacted]	[redacted]
[redacted]				[redacted]							
[redacted]				[redacted]							
[redacted]				[redacted]	[redacted]	[redacted]	[redacted]	[redacted]	[redacted]	[redacted]	[redacted]

[redacted]		[redacted]									
	[redacted]	[redacted]	[redacted]	[redacted]	[redacted]	[redacted]	[redacted]	[redacted]	[redacted]	[redacted]	[redacted]
[redacted]			[redacted]	[redacted]	[redacted]	[redacted]	[redacted]	[redacted]			
[redacted]				[redacted]	[redacted]	[redacted]	[redacted]		[redacted]		[redacted]
[redacted]				[redacted]	[redacted]	[redacted]	[redacted]		[redacted]		[redacted]
[redacted]				[redacted]	[redacted]	[redacted]			[redacted]		[redacted]
[redacted]		[redacted]	[redacted]	[redacted]	[redacted]	[redacted]	[redacted]	[redacted]			
[redacted]		[redacted]									
[redacted]		[redacted]									
[redacted]		[redacted]									

[redacted]

[redacted]

[redacted] [redacted]

[redacted]	[redacted]										
	[redacted]	[redacted]	[redacted]	[redacted]	[redacted]	[redacted]	[redacted]	[redacted]	[redacted]	[redacted]	[redacted]
[redacted]	[redacted]	[redacted]	[redacted]	[redacted]	[redacted]	[redacted]	[redacted]	[redacted]	[redacted]	[redacted]	[redacted]
[redacted]	[redacted]										
[redacted]	[redacted]	[redacted]									
[redacted]	[redacted]										
[redacted]	[redacted]	[redacted]									
[redacted]	[redacted]										
[redacted]	[redacted]	[redacted]							[redacted]		[redacted]
[redacted]	[redacted]	[redacted]							[redacted]		[redacted]
[redacted]	[redacted]										
[redacted]	[redacted]	[redacted]		[redacted]	[redacted]			[redacted]	[redacted]		[redacted]
[redacted]	[redacted]	[redacted]		[redacted]	[redacted]			[redacted]	[redacted]		[redacted]
[redacted]	[redacted]										
[redacted]	[redacted]										
[redacted]	[redacted]	[redacted]							[redacted]		[redacted]
[redacted]	[redacted]	[redacted]									
[redacted]	[redacted]	[redacted]		[redacted]	[redacted]	[redacted]	[redacted]	[redacted]	[redacted]	[redacted]	[redacted]
[redacted]				[redacted]							
[redacted]	[redacted]	[redacted]	[redacted]	[redacted]	[redacted]	[redacted]	[redacted]	[redacted]	[redacted]	[redacted]	[redacted]
[redacted]	[redacted]	[redacted]	[redacted]	[redacted]	[redacted]	[redacted]	[redacted]	[redacted]	[redacted]	[redacted]	[redacted]
[redacted]			[redacted]	[redacted]	[redacted]	[redacted]	[redacted]	[redacted]	[redacted]	[redacted]	[redacted]
[redacted]				[redacted]							
[redacted]				[redacted]							
[redacted]				[redacted]	[redacted]	[redacted]	[redacted]	[redacted]	[redacted]	[redacted]	

Time point	[redacted]										
	[redacted]	[redacted]	[redacted]	[redacted]	[redacted]	[redacted]	[redacted]	[redacted]	[redacted]	[redacted]	[redacted]
[redacted]	[redacted]	[redacted]	[redacted]	[redacted]	[redacted]	[redacted]	[redacted]	[redacted]	[redacted]	[redacted]	[redacted]
[redacted]				[redacted]	[redacted]	[redacted]	[redacted]		[redacted]		
[redacted]				[redacted]	[redacted]	[redacted]	[redacted]		[redacted]		
[redacted]				[redacted]	[redacted]	[redacted]			[redacted]		
[redacted]		[redacted]	[redacted]	[redacted]	[redacted]	[redacted]	[redacted]	[redacted]	[redacted]		
[redacted]		[redacted]									
[redacted]		[redacted]									
[redacted]		[redacted]									

[redacted]

[redacted]

[redacted] [redacted]

[redacted]	[redacted]	[redacted]	[redacted]	[redacted]	[redacted]	[redacted]	[redacted]	[redacted]	[redacted]	[redacted]	[redacted]	[redacted]	[redacted]	[redacted]	[redacted]	[redacted]	[redacted]	[redacted]	[redacted]	[redacted]	[redacted]	[redacted]
[redacted]	[redacted]																					
[redacted]		[redacted]																				
[redacted]																						
[redacted]	[redacted]																					
[redacted]		[redacted]																				
[redacted]	[redacted]																					
[redacted]		[redacted]															[redacted]				[redacted]	[redacted]
[redacted]	[redacted]	[redacted]																				[redacted]
[redacted]	[redacted]	[redacted]		[redacted]	[redacted]		[redacted]			[redacted]			[redacted]				[redacted]			[redacted]	[redacted]	[redacted]
[redacted]	[redacted]	[redacted]		[redacted]	[redacted]		[redacted]			[redacted]			[redacted]				[redacted]			[redacted]	[redacted]	[redacted]
[redacted]																						
[redacted]																						
[redacted]	[redacted]																					[redacted]
[redacted]	[redacted]	[redacted]																				
[redacted]																						[redacted]
[redacted]	[redacted]	[redacted]		[redacted]	[redacted]					[redacted]							[redacted]	[redacted]			[redacted]	[redacted]
[redacted]			[redacted]	[redacted]													[redacted]					
[redacted]	[redacted]	[redacted]	[redacted]	[redacted]	[redacted]	[redacted]	[redacted]	[redacted]	[redacted]	[redacted]	[redacted]	[redacted]	[redacted]	[redacted]	[redacted]	[redacted]	[redacted]	[redacted]	[redacted]	[redacted]	[redacted]	[redacted]
[redacted]	[redacted]	[redacted]	[redacted]	[redacted]	[redacted]	[redacted]	[redacted]	[redacted]	[redacted]	[redacted]	[redacted]	[redacted]	[redacted]	[redacted]	[redacted]	[redacted]	[redacted]	[redacted]	[redacted]	[redacted]	[redacted]	[redacted]
[redacted]				[redacted]																		
[redacted]				[redacted]	[redacted]	[redacted]	[redacted]	[redacted]	[redacted]	[redacted]	[redacted]	[redacted]	[redacted]	[redacted]	[redacted]	[redacted]	[redacted]					
[redacted]				[redacted]	[redacted]	[redacted]	[redacted]			[redacted]				[redacted]	[redacted]	[redacted]	[redacted]	[redacted]		[redacted]	[redacted]	[redacted]
[redacted]			[redacted]	[redacted]													[redacted]					

[redacted]	[redacted]	[redacted]	[redacted]	[redacted]	[redacted]	[redacted]	[redacted]	[redacted]	[redacted]	[redacted]	[redacted]	[redacted]	[redacted]	[redacted]	[redacted]	[redacted]	[redacted]	[redacted]	[redacted]	[redacted]	[redacted]	[redacted]
[redacted]				[redacted]	[redacted]	[redacted]	[redacted]			[redacted]							[redacted]			[redacted]	[redacted]	[redacted]
[redacted]				[redacted]	[redacted]	[redacted]	[redacted]			[redacted]							[redacted]			[redacted]	[redacted]	[redacted]
[redacted]				[redacted]	[redacted]					[redacted]							[redacted]				[redacted]	[redacted]
[redacted]				[redacted]						[redacted]							[redacted]					[redacted]
[redacted]				[redacted]	[redacted]					[redacted]							[redacted]				[redacted]	[redacted]
[redacted]				[redacted]													[redacted]					
[redacted]		[redacted]																				
[redacted]		[redacted]																				
[redacted]		[redacted]																				

[redacted]

[redacted]

[redacted] [redacted]

[redacted]	[redacted]	[redacted]	[redacted]	[redacted]	[redacted]	[redacted]	[redacted]	[redacted]	[redacted]	[redacted]	[redacted]	[redacted]
[redacted]	[redacted]											
[redacted]	[redacted]											
[redacted]	[redacted]											
[redacted]	[redacted]											
[redacted]	[redacted]	[redacted]		[redacted]		[redacted]	[redacted]	[redacted]		[redacted]	[redacted]	
[redacted]	[redacted]										[redacted]	
[redacted]	[redacted]	[redacted]										
[redacted]	[redacted]	[redacted]		[redacted]		[redacted]	[redacted]	[redacted]		[redacted]	[redacted]	
[redacted]	[redacted]	[redacted]		[redacted]		[redacted]	[redacted]	[redacted]		[redacted]	[redacted]	
[redacted]	[redacted]											
[redacted]	[redacted]											
[redacted]	[redacted]										[redacted]	
[redacted]	[redacted]	[redacted]									[redacted]	
[redacted]	[redacted]	[redacted]	[redacted]	[redacted]	[redacted]	[redacted]	[redacted]	[redacted]	[redacted]	[redacted]	[redacted]	
[redacted]	[redacted]	[redacted]	[redacted]	[redacted]	[redacted]	[redacted]	[redacted]	[redacted]	[redacted]	[redacted]	[redacted]	
[redacted]	[redacted]	[redacted]		[redacted]		[redacted]	[redacted]	[redacted]		[redacted]	[redacted]	
[redacted]		[redacted]	[redacted]	[redacted]	[redacted]	[redacted]	[redacted]	[redacted]				[redacted]
[redacted]		[redacted]	[redacted]	[redacted]	[redacted]	[redacted]	[redacted]	[redacted]	[redacted]	[redacted]	[redacted]	
[redacted]		[redacted]		[redacted]		[redacted]		[redacted]		[redacted]	[redacted]	
[redacted]		[redacted]		[redacted]		[redacted]		[redacted]		[redacted]	[redacted]	
[redacted]		[redacted]						[redacted]			[redacted]	
[redacted]		[redacted]		[redacted]		[redacted]		[redacted]		[redacted]	[redacted]	
[redacted]		[redacted]				[redacted]		[redacted]		[redacted]	[redacted]	

Time point [n] [redacted]	[redacted]	[redacted]	[redacted]	[redacted]	[redacted]	[redacted]	[redacted]	[redacted]	[redacted]	[redacted]	[redacted]	[redacted]
[redacted]		[redacted]				[redacted]						
[redacted]						[redacted]						[redacted]
[redacted]						[redacted]						[redacted]
[redacted]	[redacted]	[redacted]				[redacted]		[redacted]		[redacted]	[redacted]	[redacted]
[redacted]		[redacted]	[redacted]	[redacted]	[redacted]	[redacted]	[redacted]	[redacted]	[redacted]	[redacted]	[redacted]	
[redacted]		[redacted]				[redacted]		[redacted]		[redacted]	[redacted]	[redacted]
[redacted]		[redacted]	[redacted]	[redacted]	[redacted]	[redacted]	[redacted]	[redacted]	[redacted]	[redacted]	[redacted]	

[redacted]

[redacted]

[redacted] [redacted]

[redacted]	[redacted]	[redacted]	[redacted]	[redacted]	[redacted]	[redacted]	[redacted]	[redacted]	[redacted]	[redacted]	[redacted]	[redacted]	[redacted]	[redacted]	[redacted]	[redacted]	[redacted]	[redacted]	[redacted]	[redacted]	[redacted]	[redacted]
[redacted]	[redacted]																					
[redacted]		[redacted]																				
[redacted]																						
[redacted]	[redacted]																					
[redacted]		[redacted]																				
[redacted]	[redacted]																					
[redacted]		[redacted]														[redacted]					[redacted]	[redacted]
[redacted]	[redacted]	[redacted]																				[redacted]
[redacted]	[redacted]	[redacted]		[redacted]		[redacted]		[redacted]		[redacted]		[redacted]		[redacted]		[redacted]	[redacted]			[redacted]	[redacted]	[redacted]
[redacted]	[redacted]	[redacted]		[redacted]		[redacted]		[redacted]		[redacted]		[redacted]		[redacted]		[redacted]	[redacted]			[redacted]	[redacted]	[redacted]
[redacted]																						
[redacted]																						
[redacted]																						[redacted]
[redacted]	[redacted]	[redacted]																				
[redacted]																						[redacted]
[redacted]	[redacted]	[redacted]		[redacted]	[redacted]	[redacted]	[redacted]	[redacted]	[redacted]	[redacted]	[redacted]	[redacted]	[redacted]	[redacted]	[redacted]	[redacted]	[redacted]	[redacted]			[redacted]	[redacted]
[redacted]			[redacted]	[redacted]	[redacted]					[redacted]	[redacted]					[redacted]	[redacted]					
[redacted]	[redacted]	[redacted]	[redacted]	[redacted]	[redacted]	[redacted]	[redacted]	[redacted]	[redacted]	[redacted]	[redacted]	[redacted]	[redacted]	[redacted]	[redacted]	[redacted]	[redacted]	[redacted]	[redacted]	[redacted]	[redacted]	[redacted]
[redacted]	[redacted]	[redacted]	[redacted]	[redacted]	[redacted]	[redacted]	[redacted]	[redacted]	[redacted]	[redacted]	[redacted]	[redacted]	[redacted]	[redacted]	[redacted]	[redacted]	[redacted]	[redacted]	[redacted]	[redacted]	[redacted]	[redacted]
[redacted]																						
[redacted]				[redacted]		[redacted]		[redacted]		[redacted]		[redacted]		[redacted]		[redacted]						
[redacted]				[redacted]	[redacted]	[redacted]		[redacted]		[redacted]	[redacted]	[redacted]		[redacted]		[redacted]	[redacted]	[redacted]	[redacted]	[redacted]	[redacted]	[redacted]

[redacted]	[redacted]	[redacted]	[redacted]	[redacted]	[redacted]	[redacted]	[redacted]	[redacted]	[redacted]	[redacted]	[redacted]	[redacted]	[redacted]	[redacted]	[redacted]	[redacted]	[redacted]	[redacted]	[redacted]	[redacted]	[redacted]	[redacted]
[redacted]				[redacted]	[redacted]	[redacted]				[redacted]	[redacted]	[redacted]				[redacted]	[redacted]	[redacted]			[redacted]	[redacted]
[redacted]				[redacted]	[redacted]	[redacted]				[redacted]	[redacted]	[redacted]				[redacted]	[redacted]	[redacted]			[redacted]	[redacted]
[redacted]				[redacted]	[redacted]					[redacted]	[redacted]					[redacted]	[redacted]				[redacted]	[redacted]
[redacted]				[redacted]							[redacted]						[redacted]					[redacted]
[redacted]				[redacted]													[redacted]					
[redacted]		[redacted]																				
[redacted]																						
[redacted]		[redacted]																				

[redacted]

[redacted]

[redacted] [redacted]

[redacted]	[redacted]	[redacted]	[redacted]	[redacted]	[redacted]	[redacted]	[redacted]	[redacted]	[redacted]	[redacted]	[redacted]	[redacted]	[redacted]	[redacted]	[redacted]	[redacted]	[redacted]	[redacted]	[redacted]	[redacted]	[redacted]	[redacted]
[redacted]	[redacted]																					
[redacted]	[redacted]	[redacted]																				
[redacted]		[redacted]																				
[redacted]	[redacted]																					
[redacted]	[redacted]	[redacted]																				
[redacted]	[redacted]																					
[redacted]	[redacted]	[redacted]																[redacted]			[redacted]	[redacted]
[redacted]	[redacted]	[redacted]																				[redacted]
[redacted]	[redacted]	[redacted]		[redacted]	[redacted]		[redacted]				[redacted]			[redacted]				[redacted]		[redacted]	[redacted]	[redacted]
[redacted]	[redacted]	[redacted]		[redacted]	[redacted]		[redacted]				[redacted]			[redacted]				[redacted]		[redacted]	[redacted]	[redacted]
[redacted]	[redacted]																					
[redacted]	[redacted]																					
[redacted]	[redacted]																					[redacted]
[redacted]	[redacted]	[redacted]																				
[redacted]	[redacted]	[redacted]																				[redacted]
[redacted]	[redacted]	[redacted]		[redacted]	[redacted]	[redacted]	[redacted]	[redacted]	[redacted]	[redacted]	[redacted]	[redacted]	[redacted]	[redacted]	[redacted]	[redacted]	[redacted]	[redacted]	[redacted]	[redacted]	[redacted]	[redacted]
[redacted]			[redacted]	[redacted]	[redacted]						[redacted]	[redacted]						[redacted]	[redacted]			
[redacted]	[redacted]	[redacted]	[redacted]	[redacted]	[redacted]	[redacted]	[redacted]	[redacted]	[redacted]	[redacted]	[redacted]	[redacted]	[redacted]	[redacted]	[redacted]	[redacted]	[redacted]	[redacted]	[redacted]	[redacted]	[redacted]	[redacted]
[redacted]	[redacted]	[redacted]	[redacted]	[redacted]	[redacted]	[redacted]	[redacted]	[redacted]	[redacted]	[redacted]	[redacted]	[redacted]	[redacted]	[redacted]	[redacted]	[redacted]	[redacted]	[redacted]	[redacted]	[redacted]	[redacted]	[redacted]
[redacted]				[redacted]																		

Time point [redacted]	[redacted]	[redacted]	[redacted]	[redacted]	[redacted]	[redacted]	[redacted]	[redacted]	[redacted]	[redacted]	[redacted]	[redacted]	[redacted]	[redacted]	[redacted]	[redacted]	[redacted]	[redacted]	[redacted]	[redacted]	[redacted]	[redacted]
[redacted]				[redacted]			[redacted]				[redacted]			[redacted]				[redacted]				
[redacted]				[redacted]	[redacted]		[redacted]				[redacted]	[redacted]	[redacted]	[redacted]	[redacted]	[redacted]	[redacted]	[redacted]	[redacted]	[redacted]	[redacted]	[redacted]
[redacted]				[redacted]	[redacted]		[redacted]				[redacted]	[redacted]	[redacted]					[redacted]	[redacted]	[redacted]	[redacted]	[redacted]
[redacted]				[redacted]	[redacted]		[redacted]				[redacted]	[redacted]	[redacted]					[redacted]	[redacted]	[redacted]	[redacted]	[redacted]
[redacted]				[redacted]	[redacted]						[redacted]	[redacted]						[redacted]	[redacted]		[redacted]	[redacted]
[redacted]				[redacted]								[redacted]							[redacted]			[redacted]
[redacted]				[redacted]															[redacted]			
[redacted]		[redacted]																				
[redacted]		[redacted]																				
[redacted]		[redacted]																				

[redacted]

[redacted]

2.0 INTRODUCTION

2.1 Heterobifunctional Small Molecule Protein Degraders

The ubiquitin-proteasome system is the endogenous intracellular mechanism for maintaining protein homeostasis through protein degradation and turnover (Nandi, 2006).

Protein ubiquitination is controlled by E3 ligases, which mediate the polyubiquitination of lysine residues upon recognition and non-covalent binding to the target protein. There are over 600 different E3 ligases, each of which is characterized by its own tissue distribution and protein substrate specificity (Schapira, 2019). Examples of E3 ligases include cereblon (CRBN), VHL, XIAP and MDM2. Proteins that have become ubiquitinated by E3 ligases are marked for subsequent degradation by the proteasome, a protein complex that uses proteases to mediate proteolysis.

Heterobifunctional small molecules are a new class of compounds that enable the development of degraders specific for a target protein of interest. Whereas immunomodulatory drugs redirect CRBN toward neosubstrates and SERDs bind to the ER and target it for degradation, heterobifunctional molecules are comprised of 2 ligands mediating binding to both an E3 ligase and target protein of interest, respectively, joined together by a chemical linker (Schapira, 2019; Neklesa, 2017). The heterobifunctional molecule and 2 proteins form a ternary complex, bringing the E3 ligase in close proximity with a protein that now serves as its neosubstrate, leading to its ubiquitination and degradation and release of the small molecule which then mediates additional rounds of ternary complex formation and protein degradation. The requirements for stable and productive ternary complex formation contribute to the high level of selectivity that can be achieved with this approach, while the catalytic nature of the mechanism is responsible for its potency (Bondeson, 2018).

2.2 Interleukin-1 Receptor-associated Kinase-4 (IRAK4)

2.2.1 Biology and Role in Inflammation

Interleukin-1 receptor-associated kinase-4 (IRAK4) is a key component of the myddosome, a multiprotein complex involved in innate immunity that mediates signaling through toll-like receptors (TLRs) and interleukin (IL)-1 receptors (Patra, 2016). The IRAK4 protein is ubiquitously expressed across multiple different tissue types, including skin, lymphoid tissue, bone marrow, gastrointestinal (GI) tract and lung. The function of IRAK4 is dependent both on its kinase activity and on its scaffolding properties, which is required for the assembly of the myddosome complex following TLR or IL-1R engagement and myeloid differentiation factor 88 (MyD88) activation (De Nardo, 2018; Cushing, 2014; De Nardo, 2018). The NF-kB activation is particularly dependent on the scaffolding function of IRAK4 and is a key driver of cellular proliferation and proinflammatory cytokine and chemokine production mediated by myddosome activation.

The IL-1 family cytokines, including IL-1α, IL-1β, IL-18, IL-36, and IL-33, have been implicated in a variety of different autoinflammatory and autoimmune diseases, such as hidradenitis suppurativa (HS), atopic dermatitis (AD), rheumatoid arthritis (RA), systemic lupus erythematosus, and inflammatory bowel disease (Dinarello, 2018). As both TLRs and IL-1 receptors are involved in the production and/or response to all of these IL-1 family cytokines (Dinarello, 2018), IRAK4 targeting with a single small molecule degrader could have pleiotropic effects on multiple different cytokines and chemokines and thereby provide a transformative approach to the treatment of TLR/IL-1R-driven diseases.

2.2.2 Phenotype of IRAK4 Deficiency and Inhibition

Patients with IRAK4 deficiency or those who have been treated with IRAK4 kinase inhibitors provide insights into the tolerability of IRAK4 knockdown. Patients with either IRAK4 or MyD88 deficiency have been followed longitudinally, with the most comprehensive series including descriptions of 52 patients with IRAK4 deficiency and 24 with MyD88 deficiency (von Bernuth, 2012; Picard, 2011). During infancy and early childhood, these patients are susceptible to serious streptococcus, staphylococcus and pseudomonas bacterial infections but not to mycobacterial, viral, fungal or parasitic infections. After adolescence, there is no increased risk of infection, suggesting that adaptive immunity and/or further maturation of innate immunity has eliminated the dependency on MyD88-IRAK4 signaling for protection against these bacterial pathogens in adults.

Several different IRAK4 kinase inhibitors are in development for autoimmune/autoinflammatory diseases or hematologic malignancies. In a randomized Phase 2b study in RA, the Pfizer IRAK4 inhibitor PF-06650833 showed improvements in disease activity without any apparent drug-related increase in infections (Danto, 2019). In an ongoing Phase 1 oncology study of the Curis IRAK4 inhibitor CA-4948, antitumor responses were observed in lymphoma patients with no reports of infectious adverse events (AEs) (Younes, 2019).

2.3 Rationale for an IRAK4 Degrader for the Treatment of Inflammatory Cutaneous Diseases

There are numerous cutaneous, rheumatic, and GI autoinflammatory/autoimmune disease indications whose pathogenesis involves IL-1 family cytokines as well as TLR stimulation and where the pleiotropic effects of an IRAK4 degrader on these pathways can provide a significant advantage over current treatment options. There are multiple cutaneous indications, including HS and AD, where there is clinical proof of concept for targeting the IL-1R/TLR pathway but continued high unmet need for more effective therapeutics.

2.3.1 Hidradenitis Suppurativa

Hidradenitis suppurativa (HS) is a chronic, destructive, painful and debilitating inflammatory skin disease affecting up to 1% of the US population (Sabat, 2020). The disease is usually diagnosed in the 3rd and 4th decades of life, affects women more than men (3:1 ratio), and is more

common in African Americans (Alikhan, 2019). Patients with HS have numerous painful, draining nodules and abscesses, usually within skin folds, that are characterized by inflammation and bacterial colonization (Sabat, 2020; Alikhan, 2019). This is accompanied by signs and symptoms of systemic inflammation, including fatigue, depression and elevation of inflammatory biomarkers in the blood. HS is treated palliatively with corticosteroids, antibiotics and surgery (Sabat, 2020; Alikhan, 2019). The only approved drug is the anti-TNF antibody Humira, which benefits approximately 50% of patients with moderate-to-severe disease but is not curative; thus, there is a high unmet need for better therapies for HS.

Bacterial activation of TLRs, as well as the production of IL-1α, IL-1β and IL-36 by keratinocytes and inflammatory cells leading to the generation of Th1 and Th17 cell mediated inflammation characterized by high levels of TNF-α, IL-6 and IL-17, are central to the pathogenesis of HS and the reason why targeting IRAK4 with a degrader holds considerable promise for the treatment of this disease (Lowe, 2020). Monoclonal antibodies targeting IL-1α (bermekimab) and the IL-1α/β receptor (anakinra) have shown activity in HS, as have antibodies targeting IL-17 (secukinumab and bimekizumab), which is derived from Th17 cells generated through IL-1β and IL-36 stimulation. While these results provide clinical validation of the TLR/IL-1R pathway in HS, it is not yet known whether these drugs targeting single cytokines will be as effective or more effective than the anti-TNF approach, underscoring the potential advantage in HS of hitting multiple cytokines as well as TLRs involved in disease pathogenesis with an IRAK4 degrader (Lowe, 2020).

An ongoing non-interventional study evaluating IRAK4 expression in HS patients showed increased IRAK4 expression in active lesions and perilesional skin compared to unaffected skin, further supporting the role of IRAK4 in the pathogenesis of HS.

2.3.2 Atopic Dermatitis

Atopic dermatitis is a chronic, pruritic inflammatory skin disease that occurs most frequently in children but also affects adults. In the US, the prevalence of AD is approximately 11-15%. AD follows a chronic relapsing course over month to years, with dry skin and severe pruritus as the cardinal signs, sometimes accompanied by skin thickening from chronic scratching and fissuring. AD is treated palliatively with topical therapies, including emollients, corticosteroids, and phosphodiesterase inhibitors (Weidinger, 2018).

The pathogenesis of AD involves the production of Th2 and Th22 cells producing multiple cytokines (IL-4, IL-13, IL-31 and IL-22) which drive the skin inflammation and contribute to the severe pruritus. Dupilumab, a monoclonal antibody targeting IL-4Rα that blocks IL-4 and IL-13 signaling, is the only approved systemic therapy for AD patients, benefiting approximately 40% of patients with moderate-to-severe disease (Simpson, 2016; Gooderham, 2018). There remains a high unmet need for more effective therapies.

There is evidence that both IL-33 and IL-1 are involved in the generation of Th2 cell mediated inflammation in both AD and other allergic diseases (e.g. eosinophilic asthma and chronic

rhinosinusitis), and monoclonal antibodies against IL-33 (etokimab) and IL-1α (bermekimab) have shown preliminary activity in AD (Uppal, 2020). The ability of an IRAK4 degrader to impact the production of IL-33 and IL-1 (through TLR inhibition) and the cellular response to both cytokines (through IL-1R inhibition) provides a compelling mechanistic rationale for development in AD that has been de-risked through early clinical validation of the pathway and is differentiated from the targeting of single cytokines (Uppal, 2020).

2.4 KT-474

KT-474 is a potent, highly selective, orally administered heterobifunctional small molecule therapeutic targeting IRAK4 and the E3 ligase CRBN to mediate the selective degradation of IRAK4 via the ubiquitin-proteasome system.

KT-474 is composed of a CRBN-targeting ligand and an IRAK4-targeting ligand joined by a chemical linker. KT-474 forms a ternary complex through non-covalent binding to both CRBN and IRAK4, bringing the E3 ligase (CRBN) in close proximity to IRAK4, that now serves as its neosubstrate. This proximity leads to IRAK4 ubiquitination and proteosomal degradation and eventual release of KT-474, which is then free to mediate additional rounds of ternary complex formation and IRAK4 degradation.

2.4.1 Nonclinical Pharmacology Studies with KT-474

In vitro and *in vivo* studies confirmed the ability of KT-474 to selectively degrade its intended target, IRAK4, and to inhibit downstream production of disease relevant proinflammatory cytokines and chemokines. *In vitro*, KT-474's ability to degrade IRAK4 across species was confirmed in a study of mouse and rat splenocytes and dog, monkey, and human PBMCs, where similar DC_{50} values were observed across all species (<10 nM). Across a series of *in vitro* studies in human peripheral blood mononuclear cells (PBMCs), whole blood, and OCI-LY10 cells, KT-474 robustly reduced IRAK4 levels, with DC_{50} values consistently in the low nM range. Multiple *in vitro* cytokine release assays confirmed KT-474's ability to inhibit TLR agonist (lipopolysaccharide and R848) and IL-1β-induced proinflammatory cytokine production (including IL-6, TNF-α, granulocyte-macrophage colony-stimulating factor, and IL-8) in PBMCs with IC_{50} values also in the low nM range. Lastly, mass spectrometry (MS) proteomic analysis of PBMCs treated with KT-474 demonstrated the compound's selectivity for its target, with IRAK4 being the only protein degraded of more than 9,000 proteins sampled.

In vivo, murine models of inflammation demonstrated the ability of KT-474-induced IRAK4 degradation to impact TLR- and IL-1β-mediated Th1 and Th17 inflammation as well as neutrophil migration. In the mouse air pouch model of MSU-crystal induced (TLR 2/4-dependent) inflammation, 3 days twice daily administration of KT-474 at doses ranging from 30 to 100 mg/kg not only significantly reduced IRAK4 levels in the spleen, but also significantly reduced the inflammatory exudate, including reduction of neutrophils and IL-1β. Similar findings were observed in the imiquimod psoriasis model (TLR 7/8-dependent), where administration of KT-474 resulted in dose-dependent degradation of IRAK4 in the spleen and

skin associated with reduction in skin thickness as well as significant reduction of IL-1β ($p<0.0001$) and IL-6 ($p<0.05$; 300 mg/kg only) in the skin. Overall, efficacy was associated with achieving at least 80% or more IRAK4 knockdown in associated tissues in the model systems.

In vivo pharmacokinetics (PK)/ pharmacodynamics (PD) studies in mice and dogs demonstrated potent IRAK4 degradation by KT-474. In wild-type mice, a single oral dose of KT-474 at 300 mg/kg resulted in nearly 100% degradation of IRAK4 in the skin and approximately 66% degradation in the spleen, which was sustained for at least 48-hour post-dose. In both the skin and spleen, maximal PD effects were achieved after t_{max} at each dose level. In dogs, 7 days of oral administration at doses up to 10 mg/kg/day also led to marked reduction of IRAK4 in the skin and in PBMCs, with KT-474 trough plasma concentration levels as low as 3 nM inducing >85% degradation of IRAK4 in the PBMCs and degradation below the limit of quantitation in the skin. Recovery of IRAK4 levels was noted by 96 to 168 hr following last dose in dogs, demonstrating the reversible nature of KT-474 induced degradation.

Together, these studies point to the potent, on-target, and reversible effects of KT-474 against IRAK4.

2.4.2 Nonclinical Pharmacokinetic Studies with KT-474

In *in vivo* pharmacokinetic (PK) studies conducted in rats, dogs, and monkeys, KT-474 PK was characterized by moderate to high clearance, high volume of distribution at steady state, a moderate terminal half-life, and low to moderate bioavailability. [redacted]

[redacted]

[redacted]

2.4.3 Nonclinical Toxicity and Safety Studies with KT-474

[redacted]

[redacted]

[redacted]

[redacted]

2.5 Benefit/Risk Assessment

Results from the nonclinical testing program support the development of KT-474 for the treatment of moderate to severe HS and AD, starting with the assessment of the safety, tolerability, PK, and PD of orally administered KT-474 in this Phase 1 single and multiple ascending dose, randomized, double-blind, placebo-controlled trial in adult healthy volunteers (HVs) and patients with HS or AD.

While there are no IRAK4 protein degraders currently in clinical development, several IRAK4 inhibitors have been evaluated in human clinical trials to date for the treatment of inflammatory conditions, such as RA, as well as hematological malignancies, such as non-Hodgkin lymphoma (Danto, 2019; Younes, 2019; Weise, 2020). In a Phase 1 single ascending and multiple ascending dose (SAD/MAD) study in healthy subjects, no dose limiting toxicities were observed and the most common treatment-emergent AEs (TEAEs) were headache, GI disorders, and acne with no significant effects on vital signs, ECGs, or laboratory data (Danto, 2019). There was evidence of increased number of atypical crystals in the urine; however, these were not associated with any findings of renal injury (Weise, 2020). As a precaution urine microscopy has been included as an assessment in this study with KT-474. In a Phase 2 randomized, placebo-controlled study in subjects with RA, the most commonly reported TEAE was infections and infestations, which was reported in 24% of treated individuals; however, the severity of these effects has not been reported. In a Phase 1 study with a different IRAK4 inhibitor in subjects with non-Hodgkin lymphoma, the most frequent TEAEs were fatigue, reduced neutrophil count, hypercalcemia, nausea, constipation, dizziness, and decreased white blood cell count (Younes, 2019). It is unclear whether the hematological side effects observed were related to the patient population or are the result of a more aggressive dosing schedule rather than a class specific side effect; however, hematological effects were not a frequently observed finding in studies conducted with different IRAK4 inhibitors but nevertheless will be closely monitored in this study with KT-474. In summary, based on the literature available, treatment with IRAK4 inhibitors appears to be well tolerated, with only mild to moderate GI TEAEs being frequently reported across multiple studies (Weise, 2020).

The principle KT-474-related effects identified in the oral GLP repeat-dose toxicity studies in rats and dogs involved multiorgan phospholipidosis. Overall, in the repeat-dose studies, these findings were not associated with any functional correlates, were non-adverse, and did not drive the selection of the NOAEL. [redacted]

[redacted]

- [redacted]

[redacted]

The overall risk to study participants of the present study is deemed to be acceptable, as supported by the results of the nonclinical toxicology studies detailed in the Investigator's Brochure. There is no medical benefit for study participants in this study.

3.0 OBJECTIVES AND ENDPOINTS

3.1 Study Objectives

3.1.1 Primary Objective:

- To determine the safety and tolerability of KT-474 when administered as single and multiple oral doses at escalating dose levels in HVs and as multiple doses in patients with AD or HS

3.1.2 Secondary Objectives:

- To characterize the PK profile of KT-474 and its diastereomers KT-5481 and KT 5482, following single and multiple doses of KT-474 in HVs and following multiple doses in patients with AD or HS.

3.1.3 Exploratory Objectives:

- [redacted]

3.2 Study Endpoints

3.2.1 Primary Endpoints:

- Treatment-emergent (serious) adverse events ([S]AEs)
- Concomitant medication
- Clinical laboratory tests
  - Hematology
  - Coagulation
  - Chemistry
  - Urinalysis and urine microscopy
- Vital signs
  - Pulse Rate (bpm)
  - Systolic blood pressure (BP) (mm Hg)
  - Diastolic BP (mm Hg)

- Respiratory rate
- Temperature

- Safety ECG and Holter monitoring
  - Heart Rate (HR) (bpm), PR, QRS, QT, QTcF

3.2.2 Secondary Endpoints

3.2.2.1 Pharmacokinetic Evaluations in Healthy Volunteers and Patients with AD or HS

- The following (but not limited to) plasma PK parameters of KT-474, KT-5481, and KT-5482 will be calculated as appropriate:
  - Area under the curve from time zero to infinity [$AUC_{(0-\infty)}$] (single dose only), area under the curve from time zero to last measurable concentration [$AUC_{(0-last)}$], area under the concentration-time curve during a dosing interval [$AUC_{(0-tau)}$], maximum observed concentration (C_{max}), time to C_{max} (t_{max}), apparent clearance (CL/F), apparent volume of distribution (Vz/F), terminal half-life ($t_{1/2}$), mean residence time (MRT), and dose-normalized AUC and C_{max}
  - Following repeat dosing only, accumulation ratios (RAUC, RC_{max}), average concentration over the dosing interval (C_{avg}), and concentration at the end of dose interval (C_{trough})
  - Diastereomer Ratio: ratios of the diastereomers KT-5481versus KT-5482 (C_{max}, AUC, and concentration for each sampling time)

- The following (but not limited to) urine PK parameters of KT-474, KT-5481, and KT-5482 will be calculated as appropriate:
  - By-collection-interval and cumulative amount: of unchanged drug excreted in urine [$Ae_{(t1-t2)}$, $Ae_{(0-t)}$], fraction of unchanged drug [$fe_{(t1-t2)}$, $fe_{(0-t)}$]
  - Renal clearance (CLR)

3.2.3 Exploratory Endpoints:

3.2.3.1 Pharmacodynamic Endpoints

- IRAK4 levels in whole blood by FLOW (Parts A, B and C)
- IRAK4 levels in PBMC by MS (Parts A, B and C)
- IRAK4 levels in skin punch biopsies by MS and immunofluorescence (Parts B and C)
- Proinflammatory cytokines and chemokines in skin punch biopsies by MS and GEP (Parts B and C)
- Proinflammatory cytokine and chemokine production following ex vivo stimulation of whole blood by Luminex (Parts A, B, and C)

- Plasma high-sensitivity C-reactive protein levels by Luminex (Parts B [not in intermittent dosing cohorts] and C)
- Plasma Serum amyloid A and proinflammatory cytokines which may include but are not limited to TNF-α, IL-6, IL-1β, IL-4 and IL-5 by Luminex and enzyme-linked immunosorbent assay (Part C only)
- Identification of KT-474 candidate biomarkers in whole blood by RNAseq (Parts B and C)

3.2.3.2 Pharmacokinetics Endpoints

- [redacted]
- [redacted]
- KT-474 concentrations in skin punch biopsies (Parts B and C)

3.2.3.3 Efficacy Endpoints

- Change from baseline in Total Abscess and Inflammatory Nodule (AN) Count, Skin Pain Numerical Rating Scale (NRS), Peak pruritis NRS, and HS Physician's Global Assessment (HS-PGA) at 2, 4, 5 and 6 weeks in HS patients
- Change from baseline in Eczema Area and Severity Index (EASI), Peak Pruritus Numerical Rating Scale (NRS) and Investigator Global Assessment (IGA) at 2, 4, 5 and 6 weeks in AD patients

4.0 STUDY DESIGN

This is a FIH, Phase 1 study of KT-474 that will characterize the safety, PK, and PD of KT-474 after a single dose and after repeated dosing in adult HVs and in patients with HS or AD. Initially, a dose range of KT-474 in SAD escalation cohorts will be explored in adult HVs (Part A). To understand the FE on the PK and PD of KT-474 in the HVs, up to 2 SAD cohorts will be designated in Part A where the HVs will return for a second treatment period and will receive the same treatment which was originally allocated, but in the fed state. Additional cohorts at or below previously evaluated dose levels may be added to further understand the PK of KT-474 (see Section 4.1.2).

A series of MAD cohorts of HVs will evaluate the safety, PK, and PD of daily doses of KT-474 for 14 days (Part B) with doses based on the safety and PK collected in Part A. Additional multiple dose cohorts evaluating alternate dosing schedules may be added to further understand the PK of KT-474 (see Section 4.2.1). Finally, a single cohort of up to 30 patients with AD or HS will be enrolled as a single multiple dose cohort (Part C) where KT-474 will be administered for 28 days, at a dose and schedule selected by the SRC following review of the safety, PK, and PD data after completion of the dose escalation in Part B.

4.1 Part A

Part A is a double-blind, randomized, placebo-controlled, SAD, sequential group study in approximately 64 HVs, divided in 8 cohorts of eight HVs. Seven ascending single doses (1 dose level per cohort) will be investigated. One or more additional SAD cohorts may be added in the presence of food or in the fasting state, as needed, after SRC review of all relevant clinical, PK and PD data. This decision will be documented in writing and provided to the site(s) prior to Day -2. Within each cohort, 6 HVs will be randomized to receive KT-474 and 2 HVs will be randomized to receive placebo.

For the SAD cohorts, at each dose level, 2 sentinel HVs (1 receiving KT-474 and 1 receiving placebo) will be administered the investigational product first. The safety data up to 24-hours post-dose for these sentinel HVs will be reviewed by the Investigator before commencing administration of the investigational product to the remaining HVs in the cohort. Sequential dosing of HVs within a cohort will be staggered so that there will be at least a gap of 10 minutes between dosing of individual HVs.

Following completion of Part B, up to 3 additional non-randomized cohorts may be added to establish the KT-474 dose for Part C. In these additional cohorts, all HVs will receive a single dose of KT-474 in the fed state. The doses of KT-474 for these cohorts will be selected such that predicted exposures in the fed state will not exceed the highest exposures achieved in the previously completed SAD cohorts. Since exposures in these additional cohorts have already been evaluated in the study, all 8 HVs will receive KT-474 and a sentinel dosing strategy will not be utilized.

After the completion of each dose level, the blinded interim PK data through Day 5 and safety data through Day 14 will be reviewed by the SRC before proceeding to the next dose level. Each subsequence dose administration will be performed if in the judgment of the Investigator and Safety Physician, the results of the safety analyses of the preceding dose administration are satisfactory. The details of the SRC, the data to be reviewed, and the requirements for dose escalation are provided in Section 4.7.

In Part A, HVs will be screened for eligibility to participate in the study up to 26 days (Day −28) prior to admission to the study center on Day -2. Eligible HVs will be admitted to the study center on Day −2 and will be discharged on Day 5 after all scheduled assessments have been completed. Following discharge, HVs will return to the study center for a follow-up visit on Days 7, 10, and 14. In SAD Cohorts 1-7, KT-474 or placebo will be administered orally on Day 1 at 0 hours following an overnight fast (10 hours) with 240 mL of water. Safety, PK, and PD assessments will be performed throughout the treatment period (Section 1.3, Table 1). Lunch and dinner will be served on Day 1 at approximately 4 and 10 hours post-dose.

In the additional SAD cohort(s), if it is decided that KT-474 will be administered in the fed state, subjects will fast overnight (10 hrs) following which they will be provided an FDA standard high-fat breakfast 30 minutes prior to dosing which has to be completed at least 5 minutes prior to dosing. KT-474 or placebo will be administered orally on Day 1 with 240 mL of water. Lunch and dinner will be served on Day 1 at approximately 4 and 10 hours post-dose.

On other study days, during confinement in the unit, meals will be served at standard times. No other food or beverage may be consumed. The HVs would take the dose with 240 mL of water. Water will be permitted ad libitum, 1-hour post-dose. The HVs need to consume at least 2 L of water within 24-hours post-dose.

4.1.1 Food-effect

In addition, the effect of food intake on the PK of KT-474 will also be explored by selecting up to 2 SAD cohorts who will return for a second treatment period and will receive the same treatment allocation in the fed state (within 30 minutes of completion of the FDA standard high-fat breakfast). The washout period between the first treatment and second treatment will be 14 days or 5 times of KT-474 half-life, whichever is longer. Selection of cohorts will be based on the emerging safety and PK data from previous cohorts in Part A.

Following discharge, HVs will return to the study center for a follow-up visit on Days 7, 10, and 14 (Section 1.3, Table 2).

4.1.2 Additional Non-Randomized Fed-State Cohorts

Following completion of Part B, additional non-randomized cohorts may be added at or below previously evaluated dose levels to establish the dose in the fed state for Part C. Each additional cohort will consist of 8 HVs who will all receive KT-474. As the predicted exposures in these

additional cohorts have already been evaluated in the SAD cohorts, a sentinel dosing strategy will not be utilized and all HVs will be dosed on the same day.

HVs will be screened for eligibility to participate in the study up to 26 days (Day −28) prior to admission to the study center on Day -2. HVs will remain at the study center from Day – 2 through Day 5 and will be discharged following completion of all Day 5 study procedures. All HVs will return to the study center on Study Days 7, 10 and 14 for follow-up visits. Safety, PK, and PD assessments will be performed throughout the treatment period (Section 1.3,Table 3).

Subjects will fast overnight (10 hrs) after which they will be provided a standard breakfast 30 minutes prior to dosing which has to be completed at least 5 minutes prior to dosing. KT-474 will be administered orally on Day 1 with 240 mL of water. Lunch and dinner will be served on Day 1 at approximately 4 and 10 hours post-dose, respectively. Selection of the KT-474 dose will be based on the emerging safety and PK data from previous cohorts such that expected exposure of KT-474 in presence of food will not exceed the highest exposures observed in Part A.

Details of all scheduled assessments are provided in Section 1.3, Table 3.

4.2 Part B

Part B is a double-blind, randomized, placebo-controlled, MAD, sequential group study in approximately 48 HVs, divided in up to 4 cohorts of 12 HVs each. One or more additional cohorts may be added, as needed.

Within each cohort, 9 HVs will be randomized to receive KT-474 and 3 HVs will be randomized to receive placebo.

The first cohort of Part B may commence after a satisfactory review of safety, PK, and PD data from at least the first 3 cohorts in Part A. The starting dose of Part B will be 25 mg, based on the observed safety, PK, and PD in SAD Cohorts 1-3 and represents a dose where the predicted mean $ssAUC_{\tau}$ and ssC_{max} is 40% below the mean exposure observed in the SAD cohort 3, where KT-474 was confirmed to be safe and tolerable.

Target dose levels for subsequent MAD cohorts are 50, 100, 200 and 400 mg once daily for 14 days. These dose levels may be adjusted based on the safety and PK observed in the previous SAD and MAD cohorts. Dose escalation between each MAD cohort will not exceed 100% of the mean exposure observed in the preceding MAD cohort. Doses greater than 400 mg may be evaluated provided the mean daily exposure at the highest dose MAD cohort is not predicted to exceed the highest mean exposure in the SAD study where safety and tolerability of KT-474 was established.

It is planned that KT-474 or placebo will be administered orally once a day following an overnight fast for 10 hours, from Day 1 to Day 14, inclusive. However, the dosing interval and the duration of dosing may change following review of the safety, PK, and PD data from Part A.

As a precaution, Part A of the study will utilize a sentinel dosing strategy. This strategy will not be utilized in Part B unless the safety and PK data from Part A indicates otherwise (eg, safety issue). After the completion of each MAD dose level, PD data through Day 7, PK data through Day 15 and safety data through Day 28 will be reviewed by the SRC before proceeding to the next dose level. Following review of the emerging safety, PK, and PD data from the first two MAD cohorts, this period for review may change either way subject to a protocol amendment.

The details of the data required for review are provided in Section 4.7.1.2.

In Part B, HVs will be screened for eligibility to participate in the study up to 26 days (Day −28) prior to admission to the study center on Day −2. Eligible HVs will be admitted to the study center on Day −2 and will be discharged on Day 21 after all scheduled assessments have been completed.

Safety, PK, and PD assessments will be performed throughout the treatment period (Section 1.3, Table 4). Standard meals will be served at appropriate times relative to dosing as indicated in Table 4. No other food or beverage may be consumed. The HVs will take the dose with 240 mL of water. Water will be permitted ad libitum, 1-hour post-dose. The HVs will have to consume at least 2 L of water within 24-hours post-dose.

Following discharge on Day 21, HVs will return to the study center for a follow-up visit on Day 28. Additional visits may be planned following review of the emerging safety, PK, and PD data.

4.2.1 Additional Intermittent Dosing Cohorts

Up to 3 additional cohorts may be initiated to evaluate intermittent dosing schedules where KT-474 is dosed every other day (QOD; i.e., 7 doses over 14 days) and/or twice weekly (BIW; i.e., 5 doses over 15 days).

In these intermittent dosing cohorts, KT-474 will be administered in the fasted state and the dose(s) will be selected such that predicted exposures do not exceed the highest exposure previously achieved in the study.

Details of all scheduled assessments for these cohorts are provided in Section 1.3, Table 6 and Table 7.

4.3 Part C

Part C is an open-label, multiple dose study in a single cohort of up to 30 patients with AD or HS and will commence after the completion of Part B. KT-474 will be dosed daily for 28 days.

Part C will be conducted on an outpatient basis and patients will be continued to be followed for safety through Day 42. The dose will be selected by the SRC (Section 4.7) following review of the safety, PK and PD data after completion of Part B and based on evaluation of PK of KT-474 in fed state in Part A and will result in plasma exposure that is approximately equivalent to that achieved with the 100 mg QD dose in the fasted state in Part B.

KT-474 will be administered every morning within 30 minutes of starting a breakfast (i.e. in the fed state). KT-474 should be administered with approximately 240 mL (i.e., ~ 8 fluid ounces) of water. Every effort should be made to administer the dose at the same time every morning.

It is currently planned that the patients will be screened for eligibility from Day −28 and those eligible to participate will treated on Day 1. Patients will return to the clinic on Days 4, 7, 11, 14, 21, 28, 31, 35, and 42. At the discretion of the investigator, patients will be dispensed study drug to be administered at home between visits or patients will be asked to return to the clinic to receive their study medication. After the last planned dose on Day 28, patients will be followed for safety until Day 42.

Details of all scheduled assessments are provided in Section 1.3, Table 5.

4.4 Selection of Doses

4.4.1 Selection of First in Human Starting Dose

[redacted]

Table 8 [redacted]

[redacted]	[redacted]
[redacted]	[redacted]
[redacted]	[redacted]
[redacted]	[redacted]
[redacted]	[redacted]

[redacted]

4.4.2 Part A

In the SAD studies, the planned KT-474 doses are 25, 75, 150, 300, 600, 1000, and 1400 mg. Pharmacokinetic parameters at the NOAELs from the 28-day KT-474 toxicokinetic studies in rats and dogs were used to calculate exposure ratios relative to predicted human AUC and C_{max} for KT-474 (Table 9). These data indicate 79- to 159-fold exposure safety margin for the starting dose of 25 mg based on the AUCs of rat and dog NOAELs, respectively. The safety margin decreases as the dose increases. Following review of safety and PK data from HVs in the 25 mg dose cohort, dose levels of subsequent SAD cohorts may be adjusted from those proposed below but will not exceed the designated fold increase of exposure outlined for each dose level below.

Additionally, dose levels above 1400 mg may be administered in a fasted state or a previously evaluated dose level may be administered in a fed state in an optional cohort provided that the predicted mean exposure does not exceed 2-fold of the highest mean exposure observed in a previously tested SAD cohort that was determined to be safe and tolerable. The mean exposure in any given SAD cohort in the study will not exceed mean AUC of 11,700 ng*hr/mL, which is the $AUC\tau$ on Day-28 at the NOAEL of 60 mg/kg/day in dogs, the most sensitive species.

Table 9 Exposure Ratios Predicted for KT-474 in Human Relative to Rats and Dogs NOAELs from 28-day Toxicology Studies

SAD[a]	Fold Increase in Dose or Exposure	Predicted AUC	Exposure Ratio[b]		Predicted C_{max}	Exposure Ratio[c]	
Dose (mg)		ng*hr/mL	Dogs	Rats	ng/mL	Dogs	Rats
25	--	74	159	79	5	109	58
75	3	221	53	26	15	36	19
150	2	441	27	13	31	18	10
300	2	882	13	7	62	9	4.9
600	2	1765	6.6	3.3	124	4.5	2.4
1000	1.7	2941	4.0	2.0	206	2.7	1.5
1400	1.4	4118	2.8	1.4	288	1.9	1.0

[a] Dose and tablet strength may be modified based on the safety and pharmacokinetic profile of KT-474
[b] Exposure ratio = NOAEL $AUC\tau$/Predicted AUC in human
[c] Exposure ratio = NOAEL C_{max}/Predicted C_{max} in human

Following the completion of Part B, up to 3 additional non-randomized cohorts may be added to evaluate the PK of KT-474 in the fed state and establish the dose for Part C. For these additional cohorts, the KT-474 dose will be selected such that the expected exposure in presence of food does not exceed the highest exposure tested in Part A that was found to be safe and well tolerated.

4.4.3 Part B

The MAD portion of the study will evaluate up to 4 dose levels of KT-474 continuous once daily dosing for 14 days. The selection of KT-474 doses in the MAD study has been guided by the safety, tolerability, and PK data in humans from the SAD portion of the study. The initial dose level of the first MAD cohort will be 25 mg. This dose has been selected based on analysis of the PK data from SAD cohorts 1 to 3 and is predicted to result in mean $ssAUC_\tau$ and ssC_{max} ~40% lower than the mean exposure observed in the SAD cohort 3 where KT-474 was confirmed to be safe and tolerable.

The planned dose levels for subsequent MAD cohorts are 50, 100, 200 and 400 mg once daily for 14 days. These dose levels may be adjusted based on the safety and PK observed in the previous SAD and MAD cohorts. Dose escalation between each MAD cohort will not exceed 100% of the mean exposure observed in the preceding MAD cohort. Doses greater than 400 mg may be evaluated provided the predicted mean daily exposure at the highest dose MAD cohort does not exceed the highest mean exposure in the SAD study where safety and tolerability of KT-474 was established. As outlined in Section 4.2, after the completion of each MAD dose level, PD data through Day 7, PK data through Day 15 and safety data through Day 28 will be reviewed by the SRC before proceeding to the next dose level. Following review of the emerging

safety, PK, and PD data from the first two MAD cohorts, this period for review may be amended.

4.4.3.1 Additional Intermittent Dosing Cohorts

Intermittent dosing including QOD and/or BIW schedules may be evaluated in up to 3 additional cohorts. The dose levels for these subsequent cohorts will be selected based on the safety and PK observed in the previous SAD and MAD cohorts. Specifically, doses in the intermittent dosing cohorts will be selected such that the predicted mean daily exposure does not exceed the highest mean exposure observed in previous SAD and MAD cohorts in which the safety and tolerability of KT-474 have been established.

4.4.4 Part C

Part C will evaluate a single dose level of KT-474 in patients with AD or HS for safety, PK and PD in order to compare to the results observed in HVs. Part C will commence after the completion of Part B. The selection of KT-474 dose will be guided by the safety, tolerability, PK and PD data in humans from Part A (SAD) and Part B (MAD) and will be a dose in the fed state where the predicted mean $ssAUC_{\tau}$ and ssC_{max} are approximately equivalent to that observed at the 100 mg QD dose in Part B.

4.5 Study Procedures During Special Circumstances

During special circumstances (eg, COVID-19 pandemic), the specific guidance from local public health and other competent authorities regarding the protection of individuals' welfare must be applied. For the duration of such special circumstances, the following measures may be implemented for enrolled study participants:

- Safety follow-up may be made by a telephone call, other means of virtual contact, or home visit, if appropriate.
- Biological samples may be collected at a different location* other than the study site or at study participant's home. Biological samples should not be collected if they cannot be processed in a timely manner or appropriately stored until the intended use.
- A limited number of study participants may be recruited, should there be a high rate of missed visit and/or visit outside of planned interval and/or withdrawal due to the current exceptional and unpredictable circumstances.

*It is the Investigator's responsibility to identify an alternate location. The Investigator should ensure that this alternate location meets International Council for Harmonisation Guidelines for Good Clinical Practices (ICH GCP) requirements, such as adequate facilities to perform study procedures, appropriate training of the staff, and documented delegation of responsibilities in this location. This alternate location should be covered by proper insurance for the study conduct by Investigator and staff at a site other than the designated study site. Refer to Food and Drug

Administration Guidance on Conduct of Clinical Trials of Medical Products during COVID-19 Public Health Emergency (Current Version) for further details.

4.6 Dose Escalation Criteria

In Parts A and B, an appropriate interval of at least 7 days from discharge will separate the investigation of dose levels to permit a timely review and evaluation of safety, PK, and preliminary PD data prior to proceeding to a higher dose level.

All data for dose escalation decisions will be reviewed by SRC (Section 4.7). The data to be reviewed for Parts A and B are described in Section 4.7.1. The SRC meeting will take place at least 48 hours before the administration of the next dose level.

4.7 Safety Review Committee, Dose Escalation Procedures and Stopping Criteria (SAD and MAD)

The SRC will be assembled to review the blinded study data for each of the cohorts in Parts A and B and, if needed, unblinded data in Part C. The role of the SRC and its modus operandi will be defined in a written charter. The SRC will be comprised at a minimum of the Investigator(s), IQVIA Medical Monitor, and a Sponsor Medical Representative. The data to be reviewed for Parts A and B will be subjected to a quality control review and should be available to SRC members in the required format at least 24 hours prior to the scheduled SRC meeting. The SRC meeting should take place at least 48 hours before the administration of the next dose level and will be formally documented in accordance with the SRC charter. The decision by the SRC to dose escalate as planned, to dose escalate to an intermediate dose, to repeat a dose if scientifically justified, or to stop dose escalation will be documented in writing. The data required for the SRC review for Parts A and B is provided in Section 4.7.1.

4.7.1 Data Required for Dose Escalation Review

4.7.1.1 Part A

Safety and PK data from all HVs in the current dose cohort as well as any preceding cohort to identify any emerging trends will be required for review.

Safety data for review up to and including Day 14 will include AEs, concomitant medication. vital signs (BP, HR, and temperature), 12-lead ECGs for safety, and clinical laboratory safety tests (hematology, biochemistry, coagulation, urinalysis, and urine microscopy).

The PK data for review up to and including Day 5 will include KT-474 plasma concentrations, maximum plasma concentration (C_{max}), time to maximum plasma concentration (t_{max}), area under the plasma concentration-time curve (AUC), derived terminal half-life ($t_{1/2}$).

The PD data from Part A will not be included in the safety review for dose escalation.

4.7.1.2 Part B

Safety, PK, and PD data from all HVs in the current dose cohort as well as any preceding cohort to identify any emerging trends will be required for review.

Safety data for review up to and including Day 28 will include AEs, concomitant medication, vital signs (BP, HR, and temperature), 12-lead ECGs for safety, and clinical laboratory safety tests (hematology, biochemistry, coagulation, urinalysis, and urine microscopy).

The PK data for review up to and including Day 15 will include KT-474 plasma concentrations/time profiles on Day 1 and Day 14, maximum plasma concentration (C_{max}) on Day 1 and Day 14, t_{max} on Day 1 and Day 14, AUC_{tau} on Day 1 and Day 14 terminal half-life ($t_{1/2}$) on Day 1 and Day 14, if estimable.

The PD data for review up to and including Day 7 will include IRAK4 levels in skin, whole blood, and isolated PBMCs.

Following review of the emerging safety, PK, and PD data from the first two MAD cohorts, this period for review may change either way subject to a protocol amendment.

4.8 Stopping Criteria

4.8.1 Individual Study Participant Stopping Criteria

Dosing for any individual study participant will be stopped if the study participant experiences a SAE or a clinically significant possibly drug-related related AE, which in the opinion of the study physician, Investigator, or Sponsor’s medical representative warrants discontinuation from the study for that study participant’s well-being, or in case of pregnancy.

4.8.2 Dose Escalation Stopping Criteria

In Parts A and B, dose escalation for an individual protocol part may be stopped if any of the study stopping rules are met (Section 4.8.3).

In Parts A and B, dose escalation will be stopped if the mean exposure of KT-474, C_{max} and AUC_{inf} or AUC_{τ}, of a given cohort reaches predefined maximum exposure level of C_{max} (561 ng/mL) or AUC (11,700 ng*hr/mL). If the predefined exposure limits are reached and there are no safety or tolerability concerns, a lower dose may be investigated, if scientifically justified.

When it is not appropriate to escalate the dose then the same dose, a previous dose or an intermediate dose may be given following discussion between the Investigator and Sponsor. A dose level will not be repeated if any of the dose escalation stopping criteria has been met for that cohort.

If any of the criteria for stopping the study (Section 4.8.3) is met in Part A (SAD), then progression to Part B (MAD) will occur only at dose levels deemed to be safe and well tolerated

in Part A. Similarly, progression to Part C will only occur at a dose level deemed to be safe and well tolerated in Parts A and B.

If the study stopping rules (Section 4.8.3) have not been met, additional dose levels may be investigated in an additional cohort(s) in either Part A or Part B.

4.8.3 Criteria for Stopping the Study (Parts A, B, and C)

If any of the following scenarios occur within an individual study part and with a reasonable possibility of a causal relationship with the investigational product, the impacted study part will be stopped:

- ≥1 study participant experiences SAEs considered to have a reasonable possibility of relationship to the investigational product.
- ≥2 study participants experience non-tolerable AEs that are considered to have a reasonable possibility of relationship to the investigational product.
- ≥2 study participants with neutrophil counts of 1.5 x 10^9/L or below on repeat testing.
- ≥1 study participant receiving KT-474 fulfills Hy's law defined as alanine aminotransferase (ALT) >3 × the upper limit of normal (ULN) and bilirubin >2 × ULN, in the absence of significant increase in alkaline phosphatase and in the absence of an alternative diagnosis that explains the increase in total bilirubin, to be assessed from the first administration of investigational product up to and including follow-up.
- ≥2 study participants who receive KT-474 have >2 × ULN of either ALT, bilirubin, or alkaline phosphatase.
- ≥2 study participants who receive KT-474 have a QTc prolongation defined as QTcF >500 msec, or an increase of QTcF >75 msec above baseline on the 12-lead ECG, confirmed (persistent for >5 minutes) on repeated 12-lead ECGs, and not due to a new bundle branch block.

If the study stopping rules have been met but it is deemed by the SRC scientifically and ethically justifiable to investigate a new lower interim dose, then a protocol amendment will be prepared and will be subject to Institutional Review Boards (IRB) approval before it can be implemented.

4.9 End of Study Definition

The last protocol-specified visit/assessment (including any telephone contact) for the last study participant in the study will be considered the formal "end of study". The study will be fully completed after final statistical analysis.

5.0 STUDY POPULATION

Prospective approval of protocol deviations to recruitment and enrollment criteria, also known as protocol waivers or exemptions, is not permitted.

5.1 Inclusion Criteria

5.1.1 For Healthy Volunteers (Parts A and B)

1. Male HVs or female HVs aged 18 to 55 years (inclusive), at the time of consent with weight at least 50 kg and a body mass index (BMI) between 18.0 and 30.0 kg/m^2 (inclusive), at Screening.
2. Healthy volunteers must be confirmed as negative in severe acute respiratory syndrome coronavirus 2 (SARS-CoV-2) infection test at Screening and on Day −2.
3. Evidence of a personally signed and dated informed consent document indicating that the HV has been informed of all pertinent aspects of the study.
4. Male HVs and their partners of childbearing potential must agree to use a highly effective method of contraception or 2 acceptable methods of contraception until 90 days after the investigational product administration. A man or woman is of childbearing potential if he or she is biologically capable of having children in the opinion of the Investigator and is sexually active. The HVs and their partners who have been surgically sterilized for less than 6 months prior to the date of informed consent must agree to use any medically acceptable methods of contraception.
5. Female HVs of nonchildbearing potential must meet at least 1 of the following criteria:

    a. Achieved postmenopausal status, defined as follows: cessation of regular menses for at least 12 consecutive months with no alternative pathological or physiological cause; and have a serum follicle-stimulating hormone (FSH) level confirming the postmenopausal state;
    b. Have undergone a documented hysterectomy and/or bilateral oophorectomy;
    c. Have medically confirmed ovarian failure.

6. Female HVs of childbearing potential must agree to a combination of TWO of the following from 14 days prior to Day 1 until 90 days after the investigational product administration:

    a. Barrier method of contraception: condoms (male or female) with or without a spermicidal agent, diaphragm or cervical cap with spermicide
    b. IUD
    c. Hormone-based contraceptive

7. Female subjects may not be pregnant, lactating, or breast-feeding or plan to become pregnant (including ova donation) within 90 days of last study drug administration.

8. Female subjects must have a negative result for pregnancy test at Screening and on Admission.
9. HVs must be willing and able to comply with scheduled visits, treatment plan, laboratory tests, and other study procedures.

5.1.2 For Patients (Part C)

Patients must meet all of the following inclusion criteria to be eligible for enrollment in the study:

1. Male or female patients aged 18 years to 75 years (inclusive) at the time of Screening, and in generally good health, except for AD or HS.
    a. Good health is defined as no clinically relevant abnormalities based on clinical judgement of the Investigator.
2. Patients must be confirmed as negative in SARS-CoV-2 infection test at Screening and on Day 1.
3. Male patients and their partners of childbearing potential must agree to use a highly effective method of contraception or 2 acceptable methods of contraception until 90 days after the investigational product administration. A man or woman is of childbearing potential if he or she is biologically capable of having children in the opinion of the Investigator and is sexually active. The patients and their partners who have been surgically sterilized for less than 6 months prior to the date of informed consent must agree to use any medically acceptable methods of contraception.
4. Female patients of nonchildbearing potential must meet at least 1 of the following criteria:
    a. Achieved postmenopausal status, defined as follows: cessation of regular menses for at least 12 consecutive months with no alternative pathological or physiological cause; and have a serum FSH level confirming the postmenopausal state;
    b. Have undergone a documented hysterectomy and/or bilateral oophorectomy;
    c. Have medically confirmed ovarian failure.
5. Female patients of childbearing potential must agree to a combination of TWO of the following from 14 days prior to Day 1 until 90 days after the investigational product administration:

    a. Barrier method of contraception: condoms (male or female) with or without a spermicidal agent, diaphragm or cervical cap with spermicide
    b. IUD
    c. Hormone-based contraceptive

6. Female patients may not be pregnant, lactating, or breast-feeding or plan to become pregnant (including ova donation) within 90 days of last study drug administration.
7. Female patients must have a negative result for the pregnancy test at Screening Visit and on Admission.

8. Diagnosis of AD or HS for at least 6 months prior to Day 1.
9. Patients with AD: having at least 10% treatable percentage body surface area at Screening or on Admission (excluding the scalp and designated venous access areas).
10. Patients with HS: A total Abscess and Inflammatory Nodule count of ≥4 at baseline.
11. Has a BMI of 17.5 to 40.0 kg/m^2; and a total body weight >45 kg (100 lb).
12. Evidence of a personally signed and dated informed consent document indicating that the patient has been informed of all pertinent aspects of the study.
13. Patients who are willing and able to comply with scheduled visits, treatment plan, laboratory tests, and other study procedures.
14. Has adequate venous access with venous access sites having AD-unaffected, non-infected skin to permit repeated PK sampling.

5.2 Exclusion Criteria

5.2.1 For Healthy Volunteers (Parts A and B)

Healthy volunteers meeting any of the following criteria will be excluded from the study-

1. Healthy volunteers who do not conform to the above inclusion criteria.
2. Healthy volunteers with a predisposition to keloid scarring (excluded in Part B only).
3. Female HVs who are pregnant, trying to become pregnant or lactating.
4. Healthy volunteers who have a clinically relevant history or presence of respiratory, GI, renal, hepatic, hematological, lymphatic, neurological, cardiovascular, psychiatric, musculoskeletal, genitourinary, immunological, dermatological, or connective tissue diseases or disorders.
5. Healthy volunteers who have a clinically relevant surgical history (prior appendectomy or cholecystectomy is not exclusionary).
6. Healthy volunteers who have a clinically relevant family history.
7. Healthy volunteers who have a history of cardiac arrhythmia or family history of sudden cardiac death.
8. Healthy volunteers who have a history of relevant atopy including any confirmed significant allergic reactions (urticaria or anaphylaxis) against any drug, or multiple drug allergies (non-active hay fever is acceptable).
9. Healthy volunteers who have a history of relevant drug hypersensitivity.
10. Healthy volunteers who have a history of alcoholism.
11. Healthy volunteers who have a history of drug abuse.
12. Healthy volunteers who have any known factor, condition, or disease that might interfere with treatment compliance, study conduct or interpretation of the results such as drug or alcohol dependence or psychiatric disease.
13. Healthy volunteers who test positive for alcohol and drugs of abuse at Screening and on each admission. Note Alcohol will not be allowed from at least 48 hours before Screening and prior to every return visit;

14. Healthy volunteers who consume more than 14 units of alcohol a week. (unit = 1 glass of wine (125 mL) = 1 measure of spirits = ½ pint of beer)
15. Healthy volunteers who smoke, or have smoked cigarettes (or equivalent) and/or using or have used nicotine-based products within 6 months prior to admission
16. Healthy volunteers who demonstrate excess in xanthine consumption (more than 8 cups of coffee or equivalent per day).
17. Healthy volunteers who have a significant infection or known inflammatory process on Screening.
18. Healthy volunteers who have acute GI symptoms at the time of Screening or admission (eg, nausea, vomiting, diarrhea, heartburn).
19. Healthy volunteers who have an acute infection such as influenza at the time of Screening or admission.
20. Healthy volunteers who do not agree to use highly effective medically acceptable methods of contraception (as defined in Appendix 6).
21. Healthy volunteers whose results from clinical laboratory safety tests are outside the local reference range at Screening and on admission.
    Note: Subjects with results outside of the local reference range that are deemed not clinically significant by the site PI, medical monitor and the Sponsor may be enrolled
22. Healthy volunteers who have a positive hepatitis B surface antigen, hepatitis B core antibody, hepatitis C antibody, or human immunodeficiency virus (HIV) antibody, SARS-CoV-2 infection at any time or other known infection requiring antibiotic therapy within the last 3 months prior to the study.
23. Healthy volunteers who have a positive QuantiFERON gold test and/or a tuberculosis history.
24. Healthy volunteers whose Screening supine BP ≥140 mm Hg (systolic) or ≥90 mm Hg (diastolic), following at least 5 minutes of supine rest. If BP is ≥140 mm Hg (systolic) or ≥90 mm Hg (diastolic), the BP should be repeated 2 more times and the average of the 3 BP values should be used to determine the HVs eligibility.
25. Healthy volunteers whose Screening supine 12-lead ECG demonstrating a QTcF interval >450msec or a QRS interval >120msec. If QTcF exceeds 450msec, or QRS exceeds 110msec, the ECG should be repeated 2 more times and the average of the 3QTcF or QRS values should be used to determine the HV's eligibility.
26. Healthy volunteers who have used any prescribed medications other than hormonal contraceptives within 30 days of investigational product administration, or less than 5 half-lives (whichever is longer). Female subjects using oral contraceptives may enroll.
27. Healthy volunteers who have taken non-steroidal anti-inflammatory drugs within 30 days of investigational product administration, or less than 5 half-lives (whichever is longer).
28. Healthy volunteers who have used over the counter medication excluding routine vitamins and acetaminophen but including megadose (intake of 20 to 600 times the recommended daily dose) vitamin therapy within 7 days of first dosing.

29. Healthy volunteers who have been dosed with any investigational drug or device in a clinical study within 30 days of KT-474/Placebo administration, or less than 5 half-lives (whichever is longer).
30. Healthy volunteers who have previously participated in a study with an investigational product or device involving the dosing of a biological targeted at any immune pathway within 1 year prior to Screening.
31. Healthy volunteers who have received the last dose of investigational product greater than 30 days ago but who are on extended follow-up.
32. Healthy volunteers who have previously received KT-474 in either another study or another cohort in this study.
33. Healthy volunteers who have lost or donated of blood over 500 mL within 3 months prior to Screening or intention to donate blood or blood products during the study.
34. Healthy volunteers who have consumed grapefruit, grapefruit juice, Seville oranges, Seville orange marmalade, and Seville orange juice or other products containing grapefruit or Seville oranges from 7 days prior to admission to the study center and for the duration of the residential period.
35. Healthy volunteers who are Investigator site staff members directly involved in the conduct of the study and their family members, site staff members otherwise supervised by the Investigator, or study participants who are Kymera employees, including their family members, directly involved in the conduct of the study.
36. Healthy volunteers who are vegans or have medical dietary restrictions.
37. Healthy volunteers who cannot communicate reliably with the Investigator.
38. Healthy volunteers who are unlikely to co-operate with the requirements of the study.
39. Healthy volunteers who have received a vaccine (including a COVID-19 vaccine) less than 30 days prior to first dose of drug on study.

5.2.2 For Patients (Part C)

Patients meeting any of the following criteria will be excluded from the study-

1. Has any clinically significant medical disorder, condition, disease (including active or potentially recurrent dermatological conditions other than AD or HS), significant physical examination or laboratory findings that may interfere with study objectives, in the Investigator’s opinion (eg, conditions or findings that may expose a patient to unacceptable risk by study participation, confound the evaluation of treatment response or adverse events, or otherwise interfere with a patient’s ability to complete the study).
2. Patients with HS: Fistula and Tunnel count of >20 at baseline.
3. Patients with AD: Active herpes infection or history of eczema herpeticum.
4. Has an active systemic or soft tissue infection, including known actively-infected AD or HS skin lesion.

5. Has a history or evidence of clinically significant or severe allergies (eg, seasonal, pet-dander, environmental, food) requiring acute or chronic treatment (patients with allergic rhinitis who do not require treatment, or for whom an ongoing allergy treatment meets the definition of a stable regimen under Concomitant Treatment(s) section, may be eligible to participate in the study).
6. Has any planned surgical or medical procedure that would overlap with study participation from Screening through the end of study.
7. Has any cancer or have a history of cancers within the last 5 years (except curatively treated with surgical excised squamous cell carcinoma, basal cell carcinoma, or carcinoma in situ of the skin or cervix).
8. Has a known sensitivity to any of the components of the investigational product.
9. History of regular alcohol consumption exceeding 7 drinks/week for female patients or 14 drinks/week for male patients (1 drink = 5 ounces [150 mL] of wine or 12 ounces [360 mL] of beer or 1.5 ounces [45 mL] of hard liquor) within 6 months before Screening.
10. Treatment with an investigational product within 30 days or 5 half-lives preceding the first dose of investigational product (whichever is longer).
11. Treatment with CYP3A4 and P-gp inhibitors within 30 days or 5 half-lives preceding the first dose of investigational product (whichever is longer).
12. Treatment with any QTc prolonging medications within 5 half-lives preceding the first dose of investigational product
13. Bradycardia (pulse <50 bpm).
14. Uncontrolled hypertension defined as BP ≥140 mm Hg (systolic) or ≥90 mm Hg (diastolic) despite maximal medical intervention.
15. Screening supine 12-lead triplicate ECG demonstrating a mean QTcF interval >440 msec or a QRS interval >110 msec.
16. Any major cardiovascular events (e.g. myocardial infarct, unstable angina, coronary revascularization, stroke, or transient ischemic attack) within 3 months prior to screening.
17. Congestive heart failure (NYHA Class 2-4), greater than Class 1 angina pectoris, acute coronary syndrome within prior 6 months, known structural heart disease with left ventricular ejection fraction (LVEF) <40% at baseline.
18. History of ventricular tachycardia or torsade de pointe, personal or family history of sudden death or long QT syndrome, unexplained syncope or syncope within prior 2 years.
19. Patients with any of the following abnormalities in clinical laboratory tests at Screening, as assessed by the study-specific laboratory and confirmed by a single repeat test, if deemed necessary:
    a) Hepatitis B surface antigen (HBsAg), Hepatitis B core antibody , HBV_DNA, Hepatitis C antibody, HIV antibody, or TB
    b) Aspartate aminotransferase or ALT level ≥1.5 × ULN;

c) Total bilirubin level ≥1.5 × ULN; patients with a history of Gilbert's syndrome may have direct bilirubin measured and would be eligible for this study provided the direct bilirubin level is ≤ULN.

d) Serum potassium, magnesium, or calcium below lower limit of normal

Note: may qualify if repeat assessments are within normal limits following treatment for abnormal values

20. Use of prescription or nonprescription drugs, including topical corticosteroids more potent than hydrocortisone 1%, vitaminic and dietary supplements, for the treatment of HS or AD within 5 half-lives, or within 28 days (whichever is shorter prior to the first dose of investigational product. Limited use of prescription or nonprescription medications used for treatment of conditions other than HS or AD that are not believed to affect patient safety or the overall results of the study may be permitted on a case-by-case basis following approval by the Sponsor.
21. Use of any QTc prolonging medications must have been discontinued for at least 5 half-lives prior to first dose of investigational product, and cannot be used until completion of the follow-up visit.
22. Herbal supplements (including St. John's Wort) must have been discontinued at least 28-days prior to the first dose of investigational product.
23. Pregnant female patients; breastfeeding female patients; female patients of childbearing potential who are unwilling or unable to use a highly effective method of contraception as outlined in this protocol for the duration of the study and for at least 90 days after the last dose of investigational product.
24. Blood donation (excluding plasma donations and platelet donations) of approximately ≥400 mL within 3 months or ≥200 mL within a month prior to dosing.
25. History of sensitivity to heparin or heparin-induced thrombocytopenia.
26. History of HIV, hepatitis B, hepatitis C, or syphilis;
27. Unwilling or unable to comply with the criteria in this protocol.
28. Patients who are Investigator site staff members directly involved in the conduct of the study and their family members, site staff members otherwise supervised by the Investigator, or patients who are Kymera employees, including their family members, directly involved in the conduct of the study.
29. Other acute or chronic medical or psychiatric condition including recent (within the past year) or active suicidal ideation or behavior or laboratory abnormality that may increase the risk associated with study participation or investigational product administration or may interfere with the interpretation of study results and, in the judgment of the Investigator, would make the patient inappropriate for entry into this study.
30. Patients who have received the COVID-19 vaccine including booster less than 14 days prior to first dose of drug on study.

5.3 Lifestyle Considerations

5.3.1 Meals and Dietary Restrictions

Parts A and B

All meals and beverage will be provided by the site. Study participants must not consume any other food or beverage during their inpatient periods.

All study participants will refrain from consumption of grapefruit, grapefruit juice, Seville oranges, Seville orange marmalade, and Seville orange juice or other products containing grapefruit or Seville oranges from 7 days prior to admission to the study center and for the duration of the residential period.

Part A

- The HVs will fast for 10 hours, overnight, prior to dosing on Day 1.
- The HVs will receive their treatment allocation in fasted state on Treatment Period 1.
- A standard lunch will be served at approximately 4-hours postdose (after procedures), an evening dinner at 10-hours postdose, and an evening snack at 13-hours postdose after all scheduled assessments have been performed.
- The HVs will take the dose with 240 mL of water. Water will be permitted ad libitum, 1-hour postdose. The HVs need to consume at least 2 L of water within 24-hours postdose.
- On all other inpatient days, meals will be served at standard times after any scheduled assessments have been performed.
- The HVs in those cohorts participating in a second treatment period to investigate the effect food on the PK profile of KT-474 and those participating in a SAD cohort where KT-474 is planned to be administered in fed state will do so following an overnight fast will receive an FDA standard high-fat breakfast 30 minutes prior to dosing which has to be completed at least 5 minutes prior to dosing.
- The HVs participating in the additional non-randomized cohorts will fast overnight and then receive a standard breakfast 30 minutes prior to dosing that must be completed at least 5 minutes prior to dosing.
- The site will provide a standard diet including details of composition (protein, carbohydrate, fat) and calorie intake for all HVs in Part A, ensuring that all HVs in Part A receive the same meals, other than those in the FE investigation who will additionally receive an FDA standard high-fat breakfast on Day 1 in Treatment Period 2.

Part B – Cohorts with daily dosing

- The HVs will receive a standard diet throughout their inpatient period.
- On Day-1 through Day 13, HVs will fast for 10 hours, overnight, prior to dosing on Day 1 through Day 14. On days 1 through Day 14, a standard lunch will be served at approximately 4-hours postdose (after procedures), an evening dinner at 10-hours postdose, and an evening snack at 13-hours postdose after any scheduled assessments have been performed.
- On all other inpatient days, meals will be served at standard times after any scheduled assessments have been performed.
- The site will provide a standard diet including details of composition (protein, carbohydrate, fat) and calorie intake for all HVs in Part B.
- The HVs will take the dose with 240 mL of water. Water will be permitted ad libitum, 1-hour postdose. The HVs will have to consume at least 2 L of water within 24-hours postdose.

Part B – Cohorts with every other day (QOD) intermittent dosing

- In additional cohorts where KT-474 or placebo will be administered every other day (QOD), HVs will fast for 10 hours, overnight, prior to dosing days (i.e., Days 1, 3, 5, 7, 9, 11, and 13). A standard lunch will be served at approximately 4-hours postdose (after procedures), an evening dinner at 10-hours postdose, and an evening snack at 13-hours postdose after any scheduled assessments have been performed.
- On all other inpatient days, meals will be served at standard times after any scheduled assessments have been performed.
- The HVs would take the dose with 240 mL of water. Water will be permitted ad libitum, 1-hour postdose. The HVs will have to consume at least 2 L of water within 24-hours postdose.

Part B – Cohorts with twice weekly (BIW) intermittent dosing

- In additional cohorts where KT-474 or placebo will be administered twice weekly, HVs will fast for 10 hours, overnight, prior to dosing days (i.e., Days 1, 4, 8, 11 and 15). A standard lunch will be served at approximately 4-hours postdose (after procedures), an evening dinner at 10-hours postdose, and an evening snack at 13-hours postdose after any scheduled assessments have been performed.
- On all other inpatient days, meals will be served at standard times after any scheduled assessments have been performed.
- The HVs would take the dose with 240 mL of water. Water will be permitted ad libitum, 1-hour postdose. The HVs will have to consume at least 2 L of water within 24-hours postdose.

Part C

- Part C visits will be conducted on an outpatient basis
- Patients will be instructed to take their dose with food every morning within approximately 30 minutes of starting breakfast. They will be instructed to make every effort to dose at the same time every morning.
- On clinic visit days, patients will be dosed in the clinic so they should be instructed to withhold dosing at home. A breakfast will either be provided in the clinic or the patient may bring their own.
- Patient will be dispensed enough study drug to allow for at-home dosing between study visits. However, at the discretion of the study investigator, patients may return to the clinic more regularly to receive their study medication

5.3.2 Caffeine and Alcohol

- During Parts A and B, HVs will abstain from alcohol intake or ingesting caffeine- or xanthine-containing products (eg, coffee, tea, cola drinks, and chocolate) for 48 hours prior to Screening and admission, for the duration of the residential period, and for 48 hours prior to any follow-up visits.
- During Part C, patients will abstain from alcohol intake for 48 hours prior to Screening and for 48 hours prior to each clinic visit.

5.3.3 Activity

- Study participants will abstain from strenuous exercise for 24 hours before the Screening medical examination, before admission, and throughout the study.
- Study participants will be advised not to donate blood or plasma donation for at least 3 months after the last dose administration.

5.4 Screen Failures

Screen failures are defined as study participants who consent to participate in the clinical study but are not subsequently dosed. A minimal set of information is required to ensure transparent reporting of screen failures so as to meet the Consolidated Standards of Reporting Trials publishing requirements and to respond to the queries from regulatory authorities. Minimal information includes demography, screen failure details, eligibility criteria, and any SAE.

Individuals who do not meet the criteria for participation in this study (screen failure) may be rescreened after consultation with the Medical Monitor. Rescreened study participants should not be assigned the same study participant number as for the initial screening.

6.0 STUDY ASSESSMENTS AND PROCEDURES

- Protocol waivers or exemptions are not allowed.
- Immediate safety concerns should be discussed with the Sponsor immediately upon occurrence or awareness to determine if the study participant should continue or discontinue investigational product.
- Adherence to the study design requirements, including those specified in the Schedule of Assessments (SoA), is essential and required for study conduct.
- All screening evaluations must be completed and reviewed to confirm that potential study participants meet all eligibility criteria. The Investigator will maintain a screening log to record details of all study participants screened and to confirm eligibility or record reasons for screening failure, as applicable.
- For patient study part: procedures conducted as part of the patient's routine clinical management (eg, blood count) and obtained before signing of the ICF may be utilized for Screening or baseline purposes provided the procedures met the protocol-specified criteria and were performed within the time frame defined in the SoA.
- The maximum amount of blood collected from each study participant over the duration of the study, including any extra assessments that may be required, will not exceed 450 mL. Repeat or unscheduled samples may be taken for safety reasons or for technical issues with the samples.

6.1 Efficacy Assessments

Planned time points for all efficacy assessments for Part C are provided in Section 1.3, Table 5.

Patients should have photographs taken of their disease sites at the designated study visits as noted in Table 5, unless prior approval is provided from the Sponsor to proceed without photography.

6.1.1 HS Patients

Total Abscess and Inflammatory Nodule (AN) Count

The number of inflammatory nodules and abscesses, as well as the physical location, will be recorded at the designated study visits as listed in the SoA, Table 5. The site should make every effort to have the same investigator conduct these assessments at all visits.

Hidradenitis Suppurativa Physician's Global Assessment (PGA)

The HS-PGA categorizes HS into six degrees of progressive severity (clear, minimal, mild, moderate, severe or very severe) based on number of nodules, abscesses and fistulae.

Skin Pain Numeric Rating Scale (NRS)

The Skin Pain NRS will be used to assess the worst skin pain and the average skin pain due to HS in the past 24 hours and in the past week. Ratings for the two items will range from 0 (no skin pain) to 10 (skin pain as bad as you can imagine).

Subjects should be instructed to respond to the items based on a recall period of the "last 24 hours."

Peak Pruritus NRS

The severity and frequency of pruritus due to HS will be assessed using the Peak Pruritus NRS, a validated horizontal NRS. Severity will be evaluated by asking subjects to assess their worst itching due to HS over the past 24 hours and past week on an NRS anchored by the terms “no itch” (0) and “worst itch imaginable” (10) and frequency will be evaluated by asking subjects to assess frequency of pruritus over the past 24 hours and past week on an NRS anchored by the terms “never/no itching” (0) and “always/constant itching” (10).

6.1.2 AD Patients

Eczema Area and Severity Index (EASI)

The EASI quantifies the severity of a subject’s atopic dermatitis based on both severity of lesion clinical signs and the percent of BSA affected. EASI is a composite scoring by the atopic dermatitis clinical evaluator of the degree of erythema, edema/papulation, excoriation, and lichenification (each scored separately) for each of four body regions, with adjustment for the percent of body surface area involved for each body region and for the proportion of the body region to the whole body.

Peak Pruritus NRS

The severity and frequency of pruritus due to AD will be assessed using the Peak Pruritus NRS, a validated horizontal NRS. Severity will be evaluated by asking subjects to assess their worst itching due to AD over the past 24 hours and past week on an NRS anchored by the terms “no itch” (0) and “worst itch imaginable” (10) and frequency will be evaluated by asking subjects to assess frequency of pruritus over the past 24 hours and past week on an NRS anchored by the terms “never/no itching” (0) and “always/constant itching” (10).

Validated Investigator Global Assessment scale for Atopic Dermatitis (vIGA-AD™)

The Investigator’s Global Assessment for atopic dermatitis is scored on a 5-point scale (0-4), reflecting a global consideration of the erythema, induration and scaling. The clinical evaluator of atopic dermatitis will perform an assessment of the overall severity of atopic dermatitis and assign an IGA score and category. The assessment will be a static evaluation without regard to the score at a previous visit.

6.2 Safety Assessments

Planned time points for all safety assessments are provided in the SoA (Section 1.3).

6.2.1 Physical Examinations

A complete physical examination will include, at a minimum, assessments of the cardiovascular, respiratory, GI, abdomen, skin, eyes, ears, nose, throat, and neurological systems. Height and weight will also be measured and recorded.

- A brief physical examination will include, at a minimum, assessments of the skin, lungs, cardiovascular system, and abdomen (liver and spleen).

6.2.2 Vital Signs

- Axillary, skin, or digital touchless temperature, pulse rate, BP, and respiratory rate will be assessed.
- Blood pressure (systolic/diastolic) and pulse measurements will be assessed in supine position with a completely automated device. Manual techniques will be used only if an automated device is not available.
- Blood pressure and pulse measurements should be preceded by at least 5 minutes of rest for the study participant in a quiet setting without distractions (eg, television, cell phones).

6.2.3 Holter Monitoring

Holter 12-lead ECG measurements will be obtained as outlined in Section 1.3.

All ECGs for QT evaluation will be recorded by using high resolution (1000 Hz) 12-lead digital Holter recorders provided by the ECG central laboratory. All Holter recording relevant procedures will be described in a manual provided by the core laboratory.

The study center staff involved in the recording of the Holter ECGs will receive a training comprising study participant preparation, electrode placement, recording of the Holter ECG under resting conditions, and data upload from the Holter ECG recorded.

The same recorder/model will be used for all recordings for a study participant. Prior to each recording, the Holter recorder should be loaded with fresh batteries and date and time settings must be checked at the start of each study day and synchronized with an official timekeeper for all machines used in the study.

Skin preparation must be thorough and electrode positions must be according to electrode positions recommended by the laboratory manual. Electrode positions will be marked with an indelible pen at the start of the study day to ensure exact reposition. Electrodes will be applied 24 hours prior to study treatment administration and left in place until the 24-hour postdose measurements are completed.

Holter ECG monitoring will be conducted until 24-hours postdose.

The 12-lead digital ECGs will be extracted after the study participant has been resting quietly in the supine position for at least 10 minutes.

Triplicate 12-lead digital ECGs will be extracted within a 5-minute window preceding and up to the specified ECG acquisition time point.

Each extracted ECG will be analyzed by the cardiologist who will be blinded to study participant identifiers, time, and treatment. Electrocardiograms will be analyzed for PR, QRS, RR, and QT intervals using semi-automated measurements and for morphological abnormalities by experienced cardiologists. A limited number of skilled readers will be employed in the over-read process to control for variability in interpretation. All ECGs from a single study participant will be read together by the same reader. Consistent leads will be used interval estimation to the extent possible, with lead II as the primary lead. If technical issues prevent measurement in lead II, the QT will be measured in an alternative lead as per the core laboratory procedure.

On days when digital ECG Holter measurements are being taken, study participants should be restricted to a low level of physical activity and should refrain from any activities likely to stimulate or excite them (eg, video games, stimulating movies or television shows, etc). Additionally, they should refrain from using hand-held electronic or electrical devices (eg, cell phones, hair dryers, etc) as these have a potential to interfere with ECG signals. Food and drink should be restricted as detailed in the protocol as this also has the potential to affect ECG signals. Date and time of intake (start and completion) of any food during the Holter ECG recording period will be recorded on the source documents and electronic case report form (eCRF).

6.2.4 Telemetry

Study participants will continuously be monitored for safety by lead II monitoring (24-hour telemetry) until 24-hours postdose. The lead II monitoring system will be reviewed by the Investigator or Research Nurse. Paper print-outs of clinically significant events or trends will be stored as source data. For telemetry ECG individual 24-hour ECG telemetry data will be not presented in listings, since these data are only used for online safety monitoring. In the case of clinically significant results, the interpretation would result in an AE.

6.2.5 Electrocardiograms in Parts A and B

- Electrocardiograms will be taken prior to simultaneously scheduled vital signs and blood collections.
- A 10-minute resting period prior to ECG collections is required, during which the subject should rest quietly without distractions, or noise.
- Triplicate 12-lead ECG will be obtained as outlined in the SoA (see Section 1.3) using the ECG machine supplied by the Core ECG Laboratory that automatically calculates the HR and measures PR, QRS, QT, and QTcF intervals.

- At each time point at which triplicate ECGs are required, 3 individual ECG tracings should be obtained as closely as possible in succession, but no more than 2 minutes apart. The full set of triplicates should be completed in less than 4 minutes.

The Investigator or qualified designee must review the ECGs in a timely manner (i.e within 24 hours) during the subject's confinement and record any clinically relevant changes in the AE section of the eCRF.

6.2.6 Electrocardiogram Monitoring and Dose Modifications in the Intermittent Dosing Cohorts and in Part C

- Electrocardiograms will be taken prior to simultaneously scheduled vital signs and blood collections.
- A 10-minute resting period prior to ECG collections is recommended, during which the subject should rest quietly without distractions, or noise
- Triplicate 12-lead ECG will be obtained as outlined in the SoA (see Section 1.3, Table 5, Table 6 and Table 7) using the ECG machine supplied by the Core ECG Laboratory that automatically calculates the HR and measures PR, QRS, QT, and QTcF intervals.
- At each time point, 3 individual ECG tracings should be obtained as closely as possible in succession, but no more than 2 minutes apart. The full set of triplicates should be completed in less than 4 minutes.

All ECGs performed at the study centers will use the ECG machines supplied by the core laboratory and must be evaluated by the Investigator or medically qualified designee prior to the next dose.

If the Investigator or medically qualified designee determines that the ECGs meet any of the criteria noted below, then dosing must be interrupted, repeat triplicate ECGs must be performed within approximately 1 hour (preferably within 5-10 minutes), and all ECGs from this timepoint and the baseline triplicate ECGs must be submitted to the core laboratory and the Investigator must wait for assessment prior to the next dose:

- Clinically significant ECG abnormalities
- QTcF (average of triplicate ECGs) 480 msec - 500 msec and an increase from baseline > 50 msec
- QTcF (average of triplicate ECGs) > 500 msec
- QTcF (average of triplicate ECGs) increase from baseline > 75 msec

6.2.6.1 Part B - Intermittent Dosing Cohorts

For subjects in the intermittent dosing cohorts, if any of the following are confirmed by the core ECG laboratory, **KT-474 should be discontinued for the subject, the medical monitor should**

be notified immediately, and serial ECGs should be performed on an interval as discussed and agreed to with the medical monitor until resolution:

- Clinically significant ECG abnormality
- QTcF (average of triplicate ECGs) > 480 msec
- QTcF (average of triplicate ECGs) increase from baseline > 50 msec

6.2.6.2 Part C

For patients in Part C, all dose modifications based on prolonged QTcF values as noted in Table 10 **should be based on those analyzed by the core ECG laboratory.**

[redacted] [redacted]

[redacted]	[redacted]
[redacted]	[redacted]

[redacted]	[redacted]
[redacted]	[redacted]

In the event a subject or patient presents with documented Torsades de Pointes, polymorphic ventricular tachycardia with a prolonged baseline QTc interval as confirmed by the ECG core lab, the medical monitor must be immediately notified and dosing should be suspended in all ongoing subjects or patients until discussion with medical monitor and SRC.

6.2.7 Clinical Safety Laboratory Assessments

- See Appendix 3 for the list of clinical laboratory tests to be performed and to the SoA (Section 1.3) for the timing and frequency.
- The Investigator must review the laboratory report, document this review, and record any clinically relevant changes occurring during the study in the AE section of the eCRF. The laboratory reports must be filed with the source documents. Clinically significant abnormal laboratory findings are those that are not associated with the underlying disease/study

participant's health status, unless judged by the Investigator to be more severe than expected for the study participant's condition.

- All laboratory tests with values considered clinically significantly abnormal during participation in the study or within 14 days after the last dose of investigational product should be repeated until the values return to normal or baseline or are no longer considered clinically significant by the Investigator or Medical Monitor.
  - If these values do not return to normal/baseline within a period of time judged reasonable by the Investigator, the etiology should be identified, and the Sponsor notified.
  - All protocol-required laboratory assessments, as defined in Appendix 3, must be conducted in accordance with the laboratory manual and the SoA (Section 1.3).
  - If laboratory values from non-protocol-specified laboratory assessments performed at the institution's local laboratory require a change in study participant management or are considered clinically significant by the Investigator (eg, SAE or AE or dose modification), then the results must be recorded in the eCRF.

6.3 Adverse Events

The definitions of an AE or SAE can be found in Appendix 4.

Adverse events will be reported by the study participant (or, when appropriate, by a caregiver, surrogate, or the participant's legally authorized representative).

The Investigator and any designees are responsible for detecting, documenting, and recording events that meet the definition of an AE or SAE and remain responsible for following up AEs that are serious, considered related to the investigational product or study procedures, or that caused the study participant to discontinue the investigational product and/or the study (see Section 8.0).

6.3.1 Time Period and Frequency for Collecting Adverse Event and Serious Adverse Event Information

All AEs will be collected from the signing of the ICF until the follow-up visit at the time points specified in the SoA (Section 1.3).

Medical occurrences that begin before the start of investigational product but after obtaining informed consent will be recorded on the Medical History/Current Medical Conditions section of the eCRF, not the AE section.

All SAEs will be collected from the signing of the ICF until the follow-up visit at the time points specified in the SoA (Section 1.3).

All SAEs will be recorded and reported to the Sponsor or designee within 24 hours, as indicated in Appendix 4. The Investigator will submit any updated SAE data to the Sponsor within 24 hours of it being available.

Investigators are not obligated to actively seek AE or SAE after conclusion of the study participation. However, if the Investigator learns of any SAE, including a death, at any time after a study participant has been discharged from the study, and he/she considers the event to be reasonably related to the investigational product or study participation, the Investigator must promptly notify the Sponsor.

The method of recording, evaluating, and assessing causality of AE and SAE and the procedures for completing and transmitting SAE reports are provided in Appendix 4.

6.3.2 Method of Detecting Adverse Events and Serious Adverse Events

Care will be taken not to introduce bias when detecting AEs and/or SAEs. Open-ended and non-leading verbal questioning of the study participant is the preferred method to inquire about AE occurrences.

6.3.3 Follow-up of Adverse Events and Serious Adverse Events

After the initial AE/SAE report, the Investigator is required to proactively follow each study participant at subsequent visits/contacts. All SAEs will be followed until resolution, stabilization, the event is otherwise explained, or the study participant is lost to follow-up (as defined in Section 9.3). Further information on follow-up procedures is given in Appendix 4.

6.3.4 Regulatory Reporting Requirements for Serious Adverse Events

- Prompt notification by the Investigator to the Sponsor of an SAE is essential, so that legal obligations and ethical responsibilities toward the safety of study participants and the safety of an investigational product under clinical investigation are met.
- The Sponsor has a legal responsibility to notify both the local regulatory authority and other regulatory agencies about the safety of an investigational product under clinical investigation. The Sponsor will comply with country-specific regulatory requirements relating to safety reporting to the regulatory authority, IRB/Independent Ethics Committees (IEC), and Investigators.
- Investigator safety reports must be prepared for suspected unexpected serious adverse reactions according to local regulatory requirements and Sponsor policy and forwarded to Investigators as necessary.
- An Investigator who receives an Investigator safety report describing an SAE or other specific safety information (eg, summary or listing of SAEs) from the Sponsor will review and then file it along with the Investigator's Brochure and will notify the IRB/IEC, if appropriate according to local requirements.

6.3.5 Adverse Events of Special Interest

The following will be considered adverse events of special interest (AESI):

- Torsade de pointes
- Sudden death
- Ventricular tachycardia
- Ventricular fibrillation and flutter
- Syncope
- Seizures

All of these events should be considered serious adverse events (SAE) regardless of severity or causality.

In the event a patient presents with any of the AESIs listed above, the medical monitor must be immediately notified and dosing should be suspended in all ongoing patients until discussion with Medical Monitor and SRC.

6.3.6 Pregnancy

- Details of all pregnancies in female study participants and, if indicated, female partners of male study participants will be collected after the start of investigational product and until at least 90 days or 5 terminal half-lives, whichever is longer, after the last dose.
- If a pregnancy is reported, the Investigator should inform the Sponsor within 24 hours of learning of the pregnancy and should follow the procedures outlined in Appendix 6.
- Abnormal pregnancy outcomes (eg, spontaneous abortion, fetal death, stillbirth, congenital anomalies, ectopic pregnancy) are considered SAEs.

6.4 Treatment of Overdose

There is no antidote for IRAK4. If a study participant develops an infection that may or may not be related to IRAK4 lowering (ie, due to immunosuppression), the infection would be treated accordingly based on system organ class.

In the event of an overdose, the Investigator should:

1. Contact the Medical Monitor immediately.
2. Closely monitor the study participant for any AE/SAE and laboratory abnormalities until KT-474 can no longer be detected systemically (at least 14 days).
3. Obtain a plasma sample for PK analysis within 2 to 3 half-lives from the date of the last dose of investigational product if requested by the Medical Monitor (determined on a case-by-case basis).

4. Document the quantity of the excess dose as well as the duration of the overdose in the eCRF.

Decisions regarding dose interruptions or modifications will be made by the Investigator in consultation with_the Medical Monitor based on the clinical evaluation of the study participant.

6.5 Pharmacokinetics

Blood samples will be collected for measurement of plasma concentrations of KT-474 and its diastereomers KT-5481 and KT-5482 in SAD/FE (Part A), MAD (Part B), and patient cohort (Part C). Blood samples will also be used for profiling circulating metabolites of KT-474 as part of MIST analysis. Urine samples will be collected for measurement of the concentrations of KT-474 in SAD (Part A) and MAD (Part B). Concentrations of the diastereomers KT-5481 and KT-5482 may be determined in urine based on results of KT-474 measurement in urine. Based on initial data on the PK of the diastereomers, it may be decided to not measure plasma concentrations of KT-5481 and KT-5482 in future SAD/MAD cohorts and Part C. Similarly, it may be decided to not measure KT-5481 and KT-5482 in urine based on the urinary excretion data for KT-474.

Drug concentration information that may unblind the study will not be reported to study centers or blinded personnel until the study has been unblinded.

The timing and number of blood sample collection may be modified based on emerging clinical data. Any changes in the timing or addition of up to 3 time points for planned study assessments must be documented and approved by the relevant study team member and then archived in the Sponsor and study center study files but will not constitute a protocol amendment. The IRB/IEC will be informed of any safety issues that require alteration of the safety monitoring scheme or amendment of the ICF.

6.5.1 [redacted]

[redacted]

[redacted].

6.5.2 Determination of Drug Concentration

- [redacted]

6.5.3 Calculation of Pharmacokinetic Variables

[redacted]

[redacted]

[redacted]

[redacted]

[redacted] [redacted]

[redacted]	[redacted]
[redacted]	[redacted]
[redacted]	[redacted]
[redacted]	[redacted]
[redacted]	[redacted]
[redacted]	[redacted]
[redacted]	[redacted]
[redacted]	[redacted]
[redacted]	[redacted]
[redacted]	[redacted]
[redacted]	[redacted]
[redacted]	[redacted]
[redacted]	[redacted]
[redacted]	[redacted]
[redacted]	[redacted]
[redacted]	[redacted]
[redacted]	[redacted]
[redacted]	[redacted]
[redacted]	[redacted]
[redacted]	[redacted]
[redacted]	[redacted]
[redacted]	[redacted]

[redacted]

[redacted]

- [redacted]
- [redacted]
- [redacted]

[redacted]

[redacted]

[redacted]

[redacted] [redacted]

[redacted]	[redacted]
[redacted]	[redacted]
[redacted]	[redacted]
[redacted]	[redacted]
[redacted]	[redacted]
[redacted]	[redacted]
[redacted]	[redacted]
[redacted]	[redacted]

[redacted]

[redacted]

6.6 [redacted]

[redacted]

[redacted] [redacted]

[redacted]	[redacted]	[redacted]	[redacted]	[redacted]	[redacted]
[redacted]	[redacted]	[redacted]	[redacted]	[redacted]	[redacted]
[redacted]	[redacted]	[redacted]	[redacted]	[redacted]	[redacted]
[redacted]	[redacted]	[redacted]	[redacted]	[redacted]	[redacted]

[redacted]

[redacted] [redacted]

[redacted]	[redacted]	[redacted]	[redacted]	[redacted]	[redacted]
[redacted]	[redacted]	[redacted]	[redacted]	[redacted]	[redacted]
[redacted]	[redacted]	[redacted]	[redacted]	[redacted]	[redacted]
[redacted]	[redacted]	[redacted]	[redacted]	[redacted]	[redacted]
[redacted]	[redacted]	[redacted]	[redacted]	[redacted]	[redacted]
[redacted]	[redacted]	[redacted]	[redacted]	[redacted]	[redacted]
[redacted]	[redacted]	[redacted]	[redacted]	[redacted]	[redacted]

[redacted]

Table 15 [redacted]

[redacted]	[redacted]	[redacted]	[redacted]	[redacted]	[redacted]
[redacted]	[redacted]	[redacted]	[redacted]	[redacted]	[redacted]
[redacted]	[redacted]	[redacted]	[redacted]	[redacted]	[redacted]
[redacted]	[redacted]	[redacted]	[redacted]	[redacted]	[redacted]
[redacted]	[redacted]	[redacted]	[redacted]	[redacted]	[redacted]
[redacted]	[redacted]	[redacted]	[redacted]	[redacted]	[redacted]

[redacted]

Table 16 [redacted]

[redacted]	[redacted]	[redacted]	[redacted]	[redacted]	[redacted]
[redacted]	[redacted]	[redacted]	[redacted]	[redacted]	[redacted]
[redacted]	[redacted]	[redacted]	[redacted]	[redacted]	[redacted]
[redacted]	[redacted]	[redacted]	[redacted]	[redacted]	[redacted]
[redacted]	[redacted]	[redacted]	[redacted]	[redacted]	[redacted]
[redacted]	[redacted]	[redacted]	[redacted]	[redacted]	[redacted]

[redacted]

Table 17 [redacted]

[redacted]	[redacted]	[redacted]	[redacted]	[redacted]	[redacted]
[redacted]	[redacted]	[redacted]	[redacted]	[redacted]	[redacted]
[redacted]		[redacted]	[redacted]	[redacted]	[redacted]
[redacted]		[redacted]	[redacted]	[redacted]	[redacted]
[redacted]	[redacted]	[redacted]	[redacted]	[redacted]	[redacted]
[redacted]	[redacted]	[redacted]	[redacted]	[redacted]	[redacted]
[redacted]	[redacted]	[redacted]	[redacted]	[redacted]	[redacted]

[redacted]

6.7 Tissue Pharmacokinetics

[redacted]

6.8 Genetics

RNAseq will be performed on PBMCs collected from Parts B and C of this study for identification of candidate biomarkers of response to KT-474.

6.9 Health Economics OR Medical Resource Utilization and Health Economics

Health Economics/Medical Resource Utilization and Health Economics parameters are not evaluated in this study.

7.0 TREATMENT AND RESTRICTIONS

7.1 Treatment Regimen, Dosing, Duration

7.1.1 SAD Cohorts (Part A)

Within each cohort, 6 HVs will be randomized in a double-blind manner to receive KT-474 and 2 HVs will be randomized to receive placebo. At each dose level, 2 sentinel HVs (1 receiving KT-474 and 1 receiving placebo) will be administered the investigational product first.

For each cohort, the HVs will receive either a single dose of KT-474 (n = 6) or placebo (n = 2) in a double-blind manner according to the randomization scheme. Sequential dosing of the HVs within a cohort will be staggered so that there will be at least a gap of 10 minutes between dosing of individual HVs. For each cohort, HVs randomized to placebo will receive the same number of tablets as HVs randomized to KT-474.

Healthy volunteers will fast overnight for 10 hours prior to dosing and for 4 hours after dose administration. Water will be permitted ad libitum, 1-hour postdose.

In those cohorts where KT-474 is planned to be administered in fed state, healthy volunteers will fast overnight for 10 hours prior to dosing after which they will receive either an FDA standard high-fat or a standard breakfast on Day 1, to be consumed within 30 minutes prior to dosing.

Water will be permitted ad libitum, 1-hour postdose. Each dose will be administered orally with approximately 240 mL of water (additional water to complete dosing is allowed). The HVs will be instructed to swallow the tablets within 5 minutes. If the HV vomits, the dose should not be repeated. The HVs will be allowed to eat 4 hours after receiving KT 474/placebo. On all other inpatient days, standard meals will be provided at appropriate times.

7.1.2 Food Effect Cohorts (Part A)

The HVs in the 2 SAD cohorts will return for a second treatment for FE after a washout period. The washout period between the first treatment and admission on Day-2 of the second treatment will be 14 days or 5 times of KT-474 half-life, whichever is longer. The HVs will receive an FDA standard high-fat breakfast on Day 1, to be consumed within 30 minutes prior to dosing. All other meals and beverage and assessments will be same as those on Treatment Period 1 (Section 7.1.1).

7.1.3 Additional Non-randomized Cohorts (Part A)

Additional non-randomized cohorts may be added to establish the Part C dose in a fed state. In these cohorts, HVs will receive a standard breakfast on Day 1, to be consumed within 30 minutes prior to dosing. All other meals and beverages and assessments will be the same as in Section 7.1.1.

7.1.4 MAD Cohorts (Part B)

Healthy volunteers will fast overnight for 10 hours prior to dosing and for 4 hours after dose administration. KT-474 or placebo will be administered orally from Day 1 to Day 14, inclusive. Standard meals will be served at appropriate times relative to dosing. No other food or beverage may be consumed. The study participants will take the dose with 240 mL of water. The HVs will be instructed to swallow the tablets within 5 minutes. Water will be permitted ad libitum, 1-hour postdose. Study participants will have to consume at least 2 L of water within 24-hours postdose. Dosing will not occur until all pre-dose assessments have been completed.

7.1.5 Additional Intermittent Dosing Cohorts (Part B)

Additional cohorts may be added to evaluate QOD and/or BIW dosing schedules in a fasting state. In these cohorts, HVs will fast overnight, prior to dosing. All other meals and beverages and assessments will be the same as in Section 7.1.1.

7.1.6 Patient Cohort (Part C)

KT-474 will be administered orally once a day, in a fed state.

Patients will be instructed to take their dose with food every morning within 30 minutes of starting breakfast. KT-474 should be taken with approximately 240 mL (i.e., ~ 8 fluid ounces) of water. Every effort should be made to dose at the same time every morning. If the patient vomits, the dose should not be repeated.

On days that the patient has a clinic visit, dosing will not occur until all pre-dose assessments have been completed.

If any patient experiences any of the adverse events considered to be AESIs in Section 6.3.5, the medical monitor must be immediately notified and dosing should be suspended in all ongoing patients until discussion with medical monitor and SRC.

7.2 Measures to Minimize Bias: Randomization and Blinding

In Part A, HVs will be randomly assigned in a 6:2 ratio to receive KT-474 or placebo, in each cohort. Seven cohorts will be randomized (one for each dose level). Each cohort will start with 2 sentinel HVs (1 assigned to KT-474 and 1 to placebo). Thus, randomized cohorts of Part A will consist of up to 64 HVs.

Additional non-randomized cohorts may be included in which all 8 HVs will receive KT-474. Please see Section 4.1.2 for more details.

Part B MAD will have up to 4 cohorts randomized in a 9:3 ratio (KT-474 versus placebo) for a total of up to 48 HVs.

Part B intermittent dosing will have up to 3 cohorts randomized in a 9:3 ratio (KT-474 versus placebo) for a total of up to 36 HVs

Part C will be open-label, therefore, no randomization will be necessary.

If the study participant meets all eligibility criteria, randomization will be performed. Randomization lists for Part A and Part B will be prepared centrally by the Contract Research Organization using a validated computer program. The site will manually access randomization information on the study participants. Randomization will occur individually, the randomization code (and the associated treatment) will be assigned to the unique Subject Identification Number of each randomized study participant. The study is double-blind with limited access to the randomization code.

After each dose group, the SRC will determine the dosing regimen(s) for the next cohort. This decision will generally be made without breaking the randomization code. Emergency unblinding of treatment assignment for a study participant may be necessary due to a medical emergency or other adverse safety event. A designated member of each site staff shall have the ability to unblind a study participant or cohort through individually sealed documents. The unblinding of the study participant will be reported immediately by Investigator to the unblinded statistician, SRC, and other designated entities and agencies.

7.3 Investigational Product Compliance

For Parts A, B, and C, the prescribed dosage, timing, and mode of administration may not be changed, unless otherwise specified. Any departures from the intended regimen must be recorded in the eCRFs.

In Part C, prior to dispensing the investigational product, previously dispensed investigational product will be retrieved by the Investigator and compliance will be assessed. Patients exhibiting poor compliance as assessed by tablet counts should be counseled on the importance of good compliance to the study dosing regimen.

Noncompliance is defined as taking less than 80% or more than 120% of investigational product during any evaluation period (visit to visit).

During confinement, all dose administrations will be performed in the study center under the supervision of appropriately trained staff. A hand and mouth check will be performed following each dose administration. The date and time of each dose administration will be document in the study participant’s eCRF.

In Part C, study participants will record the dosing date and time, time of the last meal and any AEs or changes to concomitant medications in the diary, on days when dosing occurs outside the clinical unit.

7.3.1 Treatment Strategy

The clinical staff is responsible for the ongoing safety and wellbeing of the study participants while they are in the study center. There is a paging system to alert the clinical staff to any area in the center where a study participant may need medical attention. In the case of an emergency, cardiac resuscitation trolleys are found in the main ward areas of the study center. These trolleys contain drugs, equipment for airway insertion, circulation lines, defibrillation etc, together with oxygen cylinders, and portable suction machines. During the dosing days, the medical team member will be onsite 4 hours postdose. Additionally, a medical team member will be on call for 24 hours, and safety staff will be on site for safety supervision during the confinement period. In addition, if necessary, the clinical staff can contact further on-call physicians or public emergencies services in the event of a serious medical event. Equipment and emergency drugs are available to treat common medical emergencies that might occur in a Phase 1 study.

7.4 Study Restrictions

7.4.1 Concomitant Therapy

Any medication or vaccine (including over the counter or prescription medicines, vitamins, and/or herbal supplements) that the study participant is receiving at the time of enrollment or receives during the study must be recorded on the eCRF along with:

- Reason for use.
- Dates of administration including start and end dates.
- Dosage information including dose and frequency.

The Medical Monitor should be contacted if there are any questions regarding concomitant or prior therapy.

A list of excluded medications/therapy is provided in Appendix 5.

Healthy volunteers must abstain from taking prescription (within 30 days) or over the counter medication (within 7 days) excluding routine vitamins and acetaminophen (to be taken only if necessary) but including megadose (intake of 20 to 600 times the recommended daily dose) vitamin therapy, until completion of the follow-up visit, unless, in the opinion of the Investigator and Sponsor, the medication will not interfere with the study.

Patients with AD or HS must abstain from taking prescription or nonprescription drugs that are known to be QTc prolonging medications (Table 20).

Patients with AD or HS must abstain from taking prescription or nonprescription drugs including topical corticosteroids, Crestor, vitaminic, dietary supplements, and potential DDI medications listed below, within 5 half-lives, or within 28 days (whichever is shorter) prior to the first dose of investigational product until completion of the follow-up visit, unless, in the opinion of the Investigator and Sponsor, the medication will not interfere with the study.

- [redacted]

Paracetamol/acetaminophen, at doses of ≤1 gram/day, is permitted only if necessary, for use on the instruction of the Investigator, during the study. Other concomitant medication if medically indicated will be discussed on a case-by-case basis by the Investigator in consultation with the Medical Monitor.

8.0 INVESTIGATIONAL PRODUCTS

8.1 Clinical Trial Material

The following will be supplied by Kymera Therapeutics, Inc.:

- KT-474 drug product is presented in 25 mg and 100 mg dose strength tablets
- Placebo is identical in appearance to the KT-474 drug product

8.2 Pharmaceutical Formulations

[redacted]

8.3 Labeling and Packaging

- [redacted]e-mouthed round white HDPE induction sealed bottle fitted with a child resistant closure.

The containers are clearly labeled differentiating the dose strengths for active and placebo tablets in English. Following information are printed on labels per regulatory guidelines:

- Name of product (Product name: KT-474 tablets)
- Dose strength or placebo (KT-474 25 mg or KT-474 100 mg or Placebo 25 mg or Placebo 100 mg)
- Number of tablets per bottle (30 count)
- Lot number
- The following statement: “Caution: New Drug-Limited by Federal law to investigational use”

- Name of manufacturer: Kymera Therapeutics, Inc.

[redacted]

8.4 Dispensing Procedures and Storage Conditions

8.4.1 Dispensing Procedures

The investigational product will be dispensed and administered according to the site's applicable Standard Operation Procedures (SOPs) and randomization scheme. Details regarding the preparation and administration of the investigational product will be outlined in the study Pharmacy Manual, if needed. Only eligible study participants will receive the investigational product. Only authorized research site staff may supply or administer the investigational products.

8.4.2 Storage Conditions

All investigational products will be transported, received, stored, and handled strictly in accordance with the container or product label, the instructions supplied to the research site and its designated pharmacy, the site's SOPs, and applicable regulations. Appropriate storage temperature and transportation conditions will be maintained for the investigational product from the point of manufacture up to delivery of the investigational product.

Upon receipt by the study site, the investigational products will be promptly transferred to the appropriate environmentally controlled storage area. The research pharmacy staff will examine the shipment and temperature monitoring devices, if applicable, to verify that the investigational products were received in acceptable condition. Once inspected, the investigational products will be stored in a secure area with access restricted to authorized research pharmacy staff, under physical conditions consistent with the investigational product's specific requirements.

The research site's pharmacist or delegate is responsible for ensuring that all investigational product received at the site is inventoried and accounted for throughout the study, according to applicable regulations and the site's SOPs. All original containers, whether empty or containing investigational product will be returned to the pharmacy. Investigational products returned by study participants will be stored and disposed of according to the Sponsor's instructions. Contents of the investigational product containers will not be combined. Unused investigational products and investigational products returned by the study participants will be available for verification by the Sponsor's site monitor.

9.0 DISCONTINUATION OF INVESTIGATIONAL PRODUCT AND DISCONTINUATION/WITHDRAWAL OF THE STUDY PARTICIPANT

A study participant will be considered to have completed the study when he/she would have completed all scheduled visits as per the SoA. If a study participant misses the End of Study visit, she/he may still be considered a completer if they continue in their participation through the inpatient visit.

For all study cohorts (Parts A, B, or C), any study participant who voluntarily withdraws consent or is discontinued (eg, because of an AE) from the study prior to completion will be considered as withdrawn from the study. Study participant may be discontinued from the study at any time, at the discretion of the Investigator.

9.1 Discontinuation of Investigational product

Study Participants may withdraw or be withdrawn from study-related procedures and treatments under the following conditions:

1. Adverse event, laboratory abnormality, or any PK criteria due to which, in the opinion of the Investigator, continued participation in the study is not in the best interest of the study participant.
2. Recommendation of the SRC
3. Study participant experiences unacceptable toxicity
4. Any TEAE resulting in treatment discontinuation ≥1 week
5. Severe noncompliance to the protocol
6. Investigator's decision (based on risk to the study participant)
7. Pregnancy: Appendix 6 and Section 6.3.5 Pregnancy
8. Protocol violation
9. Withdrawal of consent
10. Lost to follow-up.
11. Termination of the study by the Investigator or Sponsor.
12. Inadvertent enrollment.

The process for dose escalation and the stopping criteria for the study are described in Section 4.0.

If a study participant who does not meet enrollment criteria is inadvertently enrolled, the investigational product must be discontinued, and the Sponsor or Sponsor designee must be contacted immediately. An exception may be granted in rare circumstances for which there is a compelling safety reason to allow the study participant to continue. In these rare cases, the

Investigator must obtain documented approval from the Sponsor or Sponsor designee to allow the study participant to continue in the study.

Study participants who discontinue investigational product will not be replaced in Parts A or B. Patients in Part C may be replaced if they discontinue treatment prior to Day 28, following a discussion with the Investigator and Sponsor.

9.2 Discontinuation/Withdrawal of Study Participant from the Study

- A study participant may withdraw from the study at any time at his/her own request or may be withdrawn at any time at the discretion of the Investigator for safety, behavioral, compliance, or administrative reasons.
- If the study participant withdraws consent for disclosure of future information, the Sponsor may retain and continue to use any data collected before the withdrawal of consent.
- If a study participant withdraws from the study, he/she may request destruction of any samples taken and not tested, and the Investigator must document this in the study center study records.
- See the SoA (Section 1.3) for data to be collected at the time of study discontinuation and follow-up and for any further evaluations that need to be completed. Should a study participant request or decide to withdraw from the study, all efforts must be made to complete and report the observations as thoroughly as possible up to the date of withdrawal.
- Study participants withdrawing due to an AE should be followed up at the follow-up visit.

9.3 Lost to Follow-up

A study participant will be considered as lost to follow-up if he or she repeatedly fails to return for scheduled visits and is unable to be contacted by the study center.

The following actions must be taken if a study participant fails to return to the clinic for a required study visit:

- The study center must attempt to contact the study participant, reschedule the missed visit as soon as possible, counsel the study participant on the importance of maintaining the assigned visit schedule, and ascertain whether or not the study participant wishes to and/or should continue in the study.
- Before a study participant is deemed lost to follow-up, the Investigator or designee must make every effort to regain contact with the study participant (where possible, 3 telephone calls and, if necessary, a certified letter to the study participant's last known mailing address or local equivalent methods). These contact attempts should be documented in the study participant's medical record.
- Should the study participant continue to remain unreachable, he/she will be considered to be withdrawn from the study.

10.0 STATISTICAL CONSIDERATIONS

10.1 Sample Size Determination

Sample size is based on practical considerations as no efficacy analysis are planned for Parts A and B in the study and only exploratory analyses are planned in Part C of this study.

There will be 8 HVs in each randomized cohort (6 active: 2 placebo) of SAD/FE (Part A), 12 HVs in each cohort (9 active: 3 placebo) for MAD (Part B), and up to 30 patients with AD or HS in the patient MAD cohort (Part C). Following completion of Part B, up to 3 non-randomized cohorts in Part A may be added with 8 HVs in each cohort. Up to 3 additional intermittent dosing cohorts may be added to Part B with 12 HVs (9 active: 3 placebo) in each cohort. This is considered to be sufficient for evaluation of safety, tolerability, PK and PD data of each cohort.

10.2 Populations for Analyses

For purposes of analysis, the analysis sets in are defined in Table 18.

Table 18 Analysis Sets

Analysis Set	Description
Safety Analysis Set	All study participants assigned to investigational product (KT-474 or placebo) and who take at least 1 dose of investigational product. Study participants will be analyzed according to the treatment they actually received.
Efficacy Analysis Set (Part C only)	All study participants in Part C who receive at least 50% of the planned doses of KT-474.
Pharmacokinetic (PK) Analysis Set	All study participants who take at least 1 dose of KT-474 and have at least 1 quantifiable plasma concentration for KT-474 collected postdose without important protocol deviations/violations or events thought to significantly affect the PK.
Pharmacodynamic (PD) Analysis Set	All study participants who take at least 1 dose of investigational product (KT-474 or placebo) and have at least 1 PD variable collected postdose without important protocol deviations/violations or events thought to significantly affect the PD.

10.3 Statistical Analyses

The SAP will be developed and finalized before database lock and will describe the study participant analysis sets to be included in the analyses, and procedures for accounting for missing, unused, and spurious data.

All analyses, summaries, and listings will be performed using SAS® software (version 9.4 or higher). No formal hypothesis testing is planned in this study. Descriptive statistics will be used as applicable to summarize the study data unless otherwise specified as follows and include at minimum:

- Continuous variables: sample size (n), mean, standard deviation (SD), median, minimum (min), and maximum (max). For PK, please see Section 10.3.2.
- Categorical variables: frequencies and percentages.

Individual study participant data will be presented in listings.

10.3.1 Efficacy Analyses

In Part C, descriptive statistics will be used to analyze the exploratory clinical efficacy endpoints:

- HS patients: Change from baseline in total abscess and inflammatory-nodule (AN) count, Skin Pain Numerical Rating Scale (NRS), Peak Pruritis NRS, and HS-Physician's Global Assessment (PGA) at 2, 4, 5 and 6 weeks in HS patients.
- AD patients: Change from baseline in Eczema Area and Severity Index (EASI), Peak Pruritus NRS, and Investigator's Global Assessment (IGA) at 2, 4, 5 and 6 weeks.

Summary statistics will be presented by visit and all efficacy data will be presented in the listings.

10.3.2 Pharmacokinetic Analyses

KT-474 and its diastereomers KT-5481 and KT-5482 plasma concentrations will be listed and summarized, as applicable by study part/treatment/cohort/day/scheduled time point, for all study parts. The summary statistics will include N (sample size), n (available data), mean, SD, coefficient of variation (expressed as percent), median, minimum, and maximum values. Data permitting, the ratio of the individual diastereomers (KT-5481 and KT-5482) to parent (KT-474) will be listed and summarized by study part/treatment/cohort/day/scheduled time point, for all study parts. Individual and mean concentrations will be graphically displayed by treatment and scheduled time on linear and semi-logarithmic scale. Attainment of steady state may be graphically explored.

Pharmacokinetic parameters will be summarized by study part/treatment/cohort/day for all study parts, using descriptive statistics. Geometric mean and geometric coefficient of variation (expressed in percent) will be included for PK parameters, where applicable. Only median, minimum, and maximum will be calculated for t_{max} and t_{lag}. A study participant listing of individual PK parameters for each study part/treatment/cohort/day will be provided, as applicable. Scatter plots of individual and geometric mean PK parameters (C_{max} and AUC) versus treatment/day will be presented by study part and/or combined across study part, as appropriate.

For SAD and MAD, the dose proportionality of the PK parameters AUCs and C_{max}, over the administered dose ranges, will be assessed using the power model. The Day 1 results from the MAD study part may be combined with the SAD results, if appropriate.

Food effect will be assessed by relative bioavailability based on PK parameters (C_{max} and AUC) between fast and fed state.

Pharmacokinetic results obtained in Part C (Patients with AD or HS) may be compared with the PK results obtained in HVs in Parts A and B. Effect of intrinsic demographic variables (eg, body weight, sex) on exposure to KT-474 may be graphically explored.

10.3.3 Pharmacodynamic Analyses

Effects of KT-474 on IRAK4 levels will be measured by FLOW and MS from blood collected in Parts A, B, and C of the study and in skin biopsies collected in Parts B and C. The change in IRAK4 levels will be calculated as a percent change from the predose sample and reported for each study part/treatment/cohort/day where PD samples are collected.

The percent change of IRAK4 in blood as measured by FLOW and MS will be correlated with KT-474 exposure in the plasma in Parts A, B and C. The percent change in skin biopsies as measured by IF and MS will be correlated with the exposure in skin in Parts B and C.

Effects of KT-474 on levels of serum high-sensitivity C-reactive protein from Parts B [not in intermittent dosing cohorts] and C and on levels of serum amyloid A protein in Part C will be evaluated as measured by Luminex. The geometric mean (90% confidence interval) change from predose in plasma will be explored out to Day 28.

Effects of KT-474 on ex vivo-stimulated cytokine and chemokine production will be evaluated in Parts A, B, and C utilizing Myriad RBM's truculture systems.

Effects of KT-474 on a panel of inflammatory cytokines will be evaluated in the blood by Luminex in Part C and in the skin by GEP in Parts B and C.

Reporting of the cytokine analyses will be further defined in the PD section of the SAP.

Effects of KT-474 on RNA transcript variation to inform candidate biomarkers of response will be evaluated on PBMCs from patients in parts B and C. Reporting will be further defined in the PD section of the SAP.

10.3.4 Tissue Pharmacokinetic Analysis

KT-474 concentration in skin tissue will be listed and summarized, as applicable by treatment/cohort/day/scheduled time point, for Part B and Part C. The summary statistics will include N (sample size), n (available data), mean, SD, coefficient of variation (expressed as percent), median, minimum, and maximum values. Data permitting, concentrations of KT-474 in skin tissues will be used to graphically explore the PK and PD relationship in skin tissue.

10.3.5 Safety Analyses

All safety analyses will be performed on the Safety Analysis Set.

Treatment-emergent AEs are defined as AEs that first occurred or worsened in severity after the first administration of investigational product and prior to 30 days after the last administration of investigational product.

Adverse events will be coded using the Medical Dictionary for Regulatory Activities. For each investigational product, numbers of TEAEs, and incidence rates will be tabulated by preferred term and system organ class.

Treatment-emergent AEs by maximum severity, TEAEs by relationship to investigational product, SAEs, TEAEs leading to death, and TEAEs leading to discontinuation of investigational product will be tabulated for each treatment group. Commonly occurring TEAEs, ie, those that occur in 5% or more of the study participants in either treatment group, will be summarized using descriptive statistics.

All laboratory test results, vital signs measurements, ECG results, weight, and BMI will be summarized for each treatment group using descriptive statistics at each visit for raw numbers and change from baseline. The incidence of treatment-emergent abnormal laboratory, vital sign, and ECG values will also be summarized using descriptive statistics.

10.3.6 Missing Data

Data from study participants who withdraw from the study, including AEs and any follow-up, will be included in the analyses of primary and secondary outcomes. Further details on how missing data will be handled (partial dates imputation etc.) will be described in the SAP.

10.4 Interim Analyses

No interim analysis is planned.

11.0 REFERENCES

Alikhan A, Sayed C, Alavi A, Alhusayen R, Brasasard A, et al. North American Clinical Management Guidelines for Hidradenitis Suppurativa: a Publication from the United States and Canadian Hidradenitis Suppurativa Foundations. Part II: Topical, Intralesional, and Systemic medical Management. *J Am Acad Dermatol.* 2019;81(1):91-101.

Bondeson D, Smith B, Burslem G, Hamman B, Ishchenko A, Crews C. Lessons in PROTAC Design from Selective Degradation with a Promiscuous Warhead. *Cell Chemical Biology*. 2018;25:78-87.

Cushing L, Stochaj W, Slegel M, Czerwinski R, Dower K, Wright Q, et al. Interleukin-1/Toll-like Receptor-induced Autophosphorylation Activates Interleukin-1 Receptor-associated Kinase 4 and Controls Cytokine Induction in a Cell Type-specific Manner. *Journal of Biological Chemistry*. 2014; 289(15):10865–10875.

Danto S, Shojaee N, Singh R, Manukyan Z, Mancuso J, Peeva E, et al. Efficacy and Safety of the Selective Interleukin-1 Receptor Associated Kinase 4 Inhibitor, PF-06650833, in Patients with Active Rheumatoid Arthritis and Inadequate Response to Methotrexate [abstract]. *Arthritis Rheumatol.* 2019;71 (suppl 10).

De Nardo D, Balka K, Gloria Y, Rao V, Latz E, Masters S. Interleukin-1 receptor–associated kinase 4 (IRAK4) plays a dual role in myddosome formation and Toll-like receptor signaling. *J. Biol Chem.* 2018;293(39):15195-15207.

Dinarello C. Overview of the IL-1 Family in Innate Inflammation and Acquired Immunity. *Immunol Rev*. 2018;281(1):8–27.

FDA Guidance, Assessing the Effects of Food on Drugs in INDs and NDAs – Clinical Pharmacology Considerations Guidance for Industry. February 2019.

Gooderham MJ, Hong HC, Eshtiaghi P, Papp KA. Dupilumab: A review of its use in the treatment of atopic dermatitis. J Am Acad Dermatol. 2018 Mar;78(3 Suppl 1):S28-S36. Doi: 10.1016/j.jaad.2017.12.022. PMID: 29471919

Halliwell WH. Cationic amphiphilic drug-induced phospholipidosis. *Toxico Pathol*. 1997;25(1):53-60.

Lowe M, Naik H, Clancy S, Pauli M, Smith K, Bi Y, et al. Immunopathogenesis of hidradenitis suppurativa and response to anti–TNF-α therapy. JCI Insight 2020;5(19):e139932. .org/10.1172/jci.

Nandi D, Tahiliani P, Kumar A, Chandu D. The ubiquitin-proteasome system. *J Biosci*. 2006 Mar;31(1):137-55.

Neklesa TK, Winkler JD, Crews CM. Targeted protein degradation by PROTACs. *Pharmacol Ther.* 2017;174:138-144.

Patra MC, Choi S. Recent Progress in the Molecular Recognition and Therapeutic Importance of Interleukin-1 Receptor-Associated Kinase 4. *Molecules*. 2016;21(11).

Picard C, Casanova JL, Puel A. Infectious diseases in patients with IRAK-4, MyD88, NEMO, or IκBα deficiency. *Clin Microbiol Rev*. 2011;24(3):490-497.

Reasor MJ, Hastings KL, Ulrich RG (2006). Drug-induced phospholipidosis: issues and future directions. Expert Opin. Drug Saf., 5: 567-583.

Sabat R, Jemec GBE, Matusiak Ł, Kimball AB, Prens E, Wolk K. Hidradenitis suppurativa. *Nat Rev Dis Primers*. 2020;6(1):18.

Schapira M, Calabrese MF, Bullock AN, Crews CM. Targeted protein degradation: expanding the toolbox. *Nat Rev Drug Discov*. 2019;18(12):949-963.

Simpson E, Bieber T, Guttman-Yassky E, Beck L, et al. Two Phase 3 Trials of Dupilumab versus Placebo in Atopic Dermatitis N Engl J Med 2016; 375:2335-2348

Uppal SK, Kearns DG, Chat VS, Han G, Wu JJ. Review and analysis of biologic therapies currently in phase II and phase III clinical trials for atopic dermatitis. J Dermatolog Treat. 2020; 8:1-11

von Bernuth H, Picard C, Puel A, Casanova JL. Experimental and natural infections in MyD88- and IRAK-4-deficient mice and humans. *Eur J Immunol.* 2012;42(12):3126-35.

Weidinger, S., Beck, L.A., Bieber, T. et al. Atopic dermatitis. Nat Rev Dis Primers 4, 1 (2018). .org/10.1038/s41572-018-0001-z

Wiese M, Manning-Bennett A, Abuhelwa A. Investigational IRAK-4 inhibitors for the treatment of rheumatoid arthritis. Expert Opin Investig Drugs. 2020;29(5):475-482.

Younes A, Nowakowski G, Rosenthal A, Leslie L, Tun H, et al. Open-Label, Dose Escalation and Expansion Trial Evaluating the Safety, PK, PD and Clinical Activity of CA-4948 in Patients with Relapsed or Refractory NH. Poster presented at: 2019 SOCO Annual Meeting; September 2019; Houston, Texas.

12.0 APPENDICES

Appendix 1 Abbreviations

Abbreviation	Definition
AD	Atopic dermatitis
AE	Adverse event
ALT	Alanine aminotransferase
AN	Abscess and inflammatory nodule
BCRP	Breast cancer resistance protein
BIW	Twice weekly
BMI	Body mass index
BP	Blood pressure
CRBN	Cereblon
DDI	Drug-drug interaction
EASI	Eczema Area and Severity Index
ECG	Electrocardiogram
eCRF	Electronic case report form
FIH	First in human
FE	Food effect
FFPE	Formalin-fixed paraffin-embedded
FSH	Follicle-stimulating hormone
GEP	Gene expression profiling
GI	Gastrointestinal
GLP	Good Laboratory Practices
HDPE	High density polyethylene
HED	Human equivalent dose
HIV	Human immunodeficiency virus
HR	Heart Rate
HRT	Hormonal replacement therapy
HS	Hidradenitis suppurativa
HV	Healthy volunteer
IC_{50}	Half-maximal inhibition concentrations
ICF	Informed consent form
ICH GCP	International Council for Harmonisation Guidelines for Good Clinical Practices

IEC	Independent Ethics Committees
IF	Immunofluorescence
IL	Interleukin
IGA	Investigator’s Global Assessment
IRAK4	Interleukin-1 receptor-associated kinase 4
IRB	Institutional Review Boards
MAD	Multiple ascending dose
MIST	Metabolites in safety testing
MS	Mass Spectrometry
MyD88	Myeloid differentiation factor 88
NOAEL	No-observed-adverse-effect level
NRS	Numerical Rating Scale
PBMC	Peripheral blood mononuclear cells
PD	Pharmacodynamics
PGA	Physician’s Global Assessment
P-gp	P-glycoprotein
PK	Pharmacokinetics
QD	Daily
QOD	Every other day
RA	Rheumatoid arthritis
SAD	Single ascending dose
SARS-CoV-2	Severe acute respiratory syndrome coronavirus 2
SDD	Spray-dried dispersion
SAE	Serious adverse event
SAP	Statistical analysis plan
SD	Standard deviation
SoA	Schedule of assessments
SOP	Standard Operating Procedures
SRC	Safety Review Committee
TEAE	Treatment-emergent adverse events
TLR	Toll-like receptors
TNF	Tumor necrosis factor
ULN	Upper limit of normal
UV	Ultraviolet

WOCBP	Woman of Childbearing Potential

DEFINITIONS

$Ae_{(t1\text{-}t2)}$	By-interval amount excreted in urine during each collection interval.
$Ae_{(0\text{-}t)}$	Cumulative amount excreted in urine during the pooled collection intervals
$AUC_{(0\text{-}\infty)}$	Area under the plasma concentration-time curve from time zero to infinity.
$AUC_{(0\text{-}last)}$	Area under the plasma concentration-time curve from time zero to last measurable concentration.
$AUC_{(0\text{-}tau)}$	Area under the plasma concentration-time curve during a dosing interval.
C_{avg}	Average concentration over the dosing interval.
CL/F	Apparent clearance.
C_{max}	Maximum observed concentration.
C_{trough}	Concentration at the end of dose interval.
F	Relative bioavailability fed/fasted.
$Fe_{(t1\text{-}t2)}$	By-interval fraction of dose excreted in urine during each collection interval.
$Fe_{(0\text{-}t)}$	Cumulative fraction of dose excreted in urine during the pooled collection intervals
MRT	Mean residence time.
$T_{1/2}$	Terminal half-life.
T_{max}	Time to $C_{max.}$
RAUC	Accumulation ratio for AUC.
RC_{max}	Accumulation ratio for $C_{max.}$
V_z/F	Apparent volume of distribution.

Appendix 2 Regulatory, Ethical, and Study Oversight Considerations

Regulatory and Ethical Considerations

- This study will be conducted in accordance with the protocol and with the following:
  - Consensus ethical principles derived from international guidelines including the Declaration of Helsinki and Council for International Organizations of Medical Sciences International Ethical Guidelines.
  - Applicable ICH GCP Guidelines.
  - Applicable laws and regulations.
- The protocol, protocol amendments, ICF, Investigator Brochure, and other relevant documents (eg, advertisements) must be submitted to an IRB/IEC by the Investigator and reviewed and approved by the IRB/IEC before the study is initiated.
- Any amendments to the protocol will require IRB/IEC and regulatory authority approval, when applicable, before implementation of changes made to the study design, except for changes necessary to eliminate an immediate hazard to study participants.
- The Investigator will be responsible for the following:
  - Providing written summaries of the status of the study to the IRB/IEC annually or more frequently in accordance with the requirements, policies, and procedures established by the IRB/IEC.
  - Notifying the IRB/IEC of SAEs or other significant safety findings as required by IRB/IEC procedures.
  - Providing oversight of the conduct of the study at the study center and adherence to requirements of 21 Code of Federal Regulations, ICH GCP, the IRB/IEC, and all other applicable local regulations.
- After reading the protocol, each Investigator will sign the protocol signature page and send a copy of the signed page to the Sponsor or representative (Appendix 8). The study will not start at any study center at which the Investigator has not signed the protocol.

Adequate Resources

The Investigator is responsible for supervising any individual or party to whom the Investigator delegates study-related duties and functions conducted at the study center.

If the Investigator/institution retains the services of any individual or party to perform study-related duties and functions, the Investigator/institution should ensure this individual or party is qualified to perform those study-related duties and functions and should implement procedures to ensure the integrity of the study-related duties and functions performed and any data generated.

Financial Disclosure

Investigators and sub-Investigators will provide the Sponsor with sufficient, accurate financial information as requested to allow the Sponsor to submit complete and accurate financial certification or disclosure statements to the appropriate regulatory authorities. Investigators are responsible for providing information on financial interests during the course of the study and for 1 year after completion of the study.

Insurance

The Sponsor has obtained liability insurance, which covers this study as required by local law and/or national regulations and/or ICH GCP, whichever is applicable. The terms of the insurance will be kept in the study files.

Informed Consent Process

- The Investigator or his/her representative will explain the nature of the study to the study participant or his/her legally authorized representative and answer all questions regarding the study.
- Study participants must be informed that their participation is voluntary. Study participants or their legally authorized representative will be required to sign a statement of informed consent that meets the requirements of 21 Code of Federal Regulations 50, local regulations, ICH GCP, Health Insurance Portability and Accountability Act requirements, where applicable, and the IRB/IEC or study center.
- The medical record must include a statement that written informed consent was obtained before the study participant was entered in the study and the date the written consent was obtained. The authorized person obtaining the informed consent must also sign the ICF.
- Study participants must be re-consented to the most current version of the ICF(s) during their participation in the study.
- A copy of the ICF(s) must be provided to the study participant or the study participant's legally authorized representative.

Study participants who are rescreened are required to sign a new ICF.

Data Protection

- Study participants will be assigned a unique identifier by the Sponsor. Any study participant records or datasets that are transferred to the Sponsor will contain the identifier only; study participant names or any information which would make the study participant identifiable will not be transferred.
- The study participant must be informed that his/her personal study-related data will be used by the Sponsor in accordance with local data protection law. The level of disclosure must also be explained to the study participant.

- The study participant must be informed that his/her medical records may be examined by Clinical Quality Assurance auditors or other authorized personnel appointed by the Sponsor, by appropriate IRB/IEC members, and by inspectors from regulatory authorities.

Administrative Structure

The SRC will be comprised at a minimum of the Investigator, IQVIA Medical Monitor, and a Sponsor Representative. The Investigator and the Sponsor, when appropriate, will invite other specialist individuals to participate in the review, eg, PK scientists, statisticians, clinical specialists etc.

Medical Monitor

Diego Fay

.com

Dissemination of Clinical Study Data

The results of the study should be reported within 1 year from the end of the clinical study. Irrespective of the outcome, the Sponsor will submit to the US database a summary of the results of the clinical study within 1 year from the end of the clinical study. It shall be accompanied by a summary written in a manner that is understandable to laypersons.

Data Quality Assurance

- All study participant data relating to the study will be recorded on printed or eCRFs unless transmitted to the Sponsor or designee electronically (eg, laboratory data). The Investigator is responsible for verifying that data entries are accurate and correct by signing the eCRF.
- The Investigator must maintain accurate documentation (source data) that supports the information entered in the eCRF.
- The Investigator must permit study-related monitoring, audits, IRB/IEC review, and regulatory agency inspections and provide direct access to source data documents.
- The Sponsor or designee is responsible for the data management of this study including quality checking of the data.
- Study monitors will perform ongoing source data verification to confirm that data entered into the eCRF by authorized study center personnel are accurate, complete, and verifiable from source documents; that the safety and rights of study participants are being protected; and that the study is being conducted in accordance with the currently approved protocol and any other study agreements, ICH GCP, and all applicable regulatory requirements.
- Records and documents, including signed ICFs, pertaining to the conduct of this study must be retained by the Investigator for 15 years after study completion unless local regulations or institutional policies require a longer retention period. No records may be destroyed during

the retention period without the written approval of the Sponsor. No records may be transferred to another location or party without written notification to the Sponsor.

Source Documents

The Investigator/institution should maintain adequate and accurate source documents and study records that include all pertinent observations on each of the study center's study participants. Source data should be attributable, legible, contemporaneous, original, accurate, and complete. Changes to source data should be traceable, should not obscure the original entry, and should be explained if necessary (eg, via an audit trail).

- Source documents provide evidence for the existence of the study participant and substantiate the integrity of the data collected. Source documents are filed at the Investigator's study center.
- Data entered in the eCRF that are transcribed from source documents must be consistent with the source documents or the discrepancies must be explained. The Investigator may need to request previous medical records or transfer records, depending on the study. Also, current medical records must be available.

Study and Study Center Closure

The Sponsor designee reserves the right to close the study center or terminate the study at any time for any reason at the sole discretion of the Sponsor. Study centers will be closed upon study completion. A study center is considered closed when all required documents and study supplies have been collected and a study center closure visit has been performed.

The Investigator may initiate study center closure at any time, provided there is reasonable cause and sufficient notice is given in advance of the intended termination.

Reasons for the early closure of a study center by the Sponsor or Investigator may include but are not limited to:

- Failure of the Investigator to comply with the protocol, the requirements of the IRB/IEC or local health authorities, the Sponsor's procedures, or ICH GCP guidelines.
- Inadequate recruitment of study participants by the Investigator.
- Discontinuation of further investigational product development.

Publication Policy

The data generated by this study are confidential information of the Sponsor. The Sponsor will make the results of the study publicly available. The publication policy with respect to the Investigator and study center will be set forth in the Clinical Trial Agreement.

Appendix 3 Clinical Laboratory Tests

- The tests detailed in Table 19 will be performed by the local laboratory.
- Protocol-specific requirements for inclusion or exclusion of study participants are detailed in Section 5.0 of the protocol.
- Additional tests may be performed at any time during the study as determined necessary by the Investigator or required by local regulations.

Table 19 Protocol-required Safety Laboratory Assessments

**Laboratory Assessments**	**Parameters**	
Hematology	Platelet Count	White Blood Cell Count with Differential:
	Red Blood Cell Count	Neutrophils
	Hemoglobin	Lymphocytes
	Hematocrit	Monocytes
	Red Blood Cell Indices:	Eosinophils
	Mean corpuscular volume	Basophils
	Mean corpuscular hemoglobin	
	Mean cell hemoglobin concentration	
	%Reticulocytes	
Clinical Chemistry	Blood urea nitrogen	Aspartate Aminotransferase
	Creatinine	Alanine Aminotransferase
	Glucose (fasting)	Alkaline phosphatase
	Gamma glutamyl transferase	Creatine kinase
	Urea	Creatine kinase myocardial band fraction will be performed if clinically indicated
	Magnesium	Chloride
	Cholesterol	Globulin*
	Potassium	Amylase
	Sodium	Total and direct bilirubin
	Calcium	Conjugated and unconjugated bilirubin will be performed if clinically indicated.
	Lactate dehydrogenase	Total Protein
	Albumin	Phosphate
	Uric acid	Potassium
	Triglycerides	Carbon dioxide (bicarbonate)

Laboratory Assessments	Parameters	
Coagulation	International normalized ratio	Activated partial thromboplastin time
	Prothrombin time	Thrombin time*
	Partial prothrombin time	Fibrinogen*
Urinalysis	Leucocytes	Red blood cells
	Protein	pH
	Bilirubin	Nitrite
	Urobilinogen	Specific gravity
	Ketones	Glucose
	Microscopy	Crystal, casts, microorganisms
	Urine creatinine	
Viral serology	HIV-1, HIV-2 antibody	Hepatitis B core antigen (HbcAg)<br>Hepatitis virus B DNA (HBV DNA)<br>Hepatitis C virus antibody
	Hepatitis B virus surface antigen (HbsAg)	QuantiFERON gold
Drugs of abuse and alcohol	Amphetamine	Opiates
	Ethanol	Benzodiazepines
	Cannabinoids	Methadone metabolites
	Cocaine metabolites	Barbiturates
	Cotinine	Ecstasy (3,4-Methylenedioxymethamphetamine)
	Tricyclic anti-depressants	Phencyclidine
**Note: Drug and alcohol screening is not required for Part C patients.**		
Thyroid profile	Thyroid stimulating hormone<br>Total T4 Free triiodothyronine (T3)	Free thyroxine (T4)<br>Total T3
Glycosylated hemoglobin	HbA1c	
Other screening tests	Serum follicle-stimulating hormone<br>Serum pregnancy test<br>.	

Investigators must document their review of each laboratory safety report.

*Day -2 values are not required for review prior to dosing on Day 1 as long as they were within normal limits or were abnormal but not clinical significant at screening

Appendix 4 Adverse Events: Definitions and Procedures for Recording, Evaluating, Follow-up, and Reporting

Definition of AE

AE Definition
• An AE is any untoward medical occurrence in a study participant, temporally associated with the use of investigational product, whether or not considered related to the investigational product.<br>• An AE can therefore be any unfavorable and unintended sign (including an abnormal laboratory finding), symptom, or disease (new or exacerbated) temporally associated with the use of investigational product.

Events Meeting the AE Definition
• Any abnormal laboratory test results (hematology, clinical chemistry, or urinalysis) or other safety assessments (eg, ECG, radiological scans, vital signs measurements), including those that worsen from baseline, considered clinically significant in the medical and scientific judgment of the Investigator (ie, not related to progression of underlying disease).<br>• Exacerbation of a chronic or intermittent pre-existing condition including either an increase in frequency and/or intensity of the condition.<br>• New conditions detected or diagnosed after investigational product administration even though it may have been present before the start of the study.<br>• Signs, symptoms, or the clinical sequelae of a suspected DDI.<br>• Signs, symptoms, or the clinical sequelae of a suspected overdose of either investigational product or a concomitant medication. Overdose per se will not be reported as an AE/SAE unless it is an intentional overdose taken with possible suicidal/self-harming intent. Such overdoses should be reported regardless of sequelae.

Events NOT Meeting the AE Definition
• Any clinically significant abnormal laboratory findings or other abnormal safety assessments which are associated with the underlying disease, unless judged by the Investigator to be more severe than expected for the study participant's condition.<br>• The disease/disorder being studied or expected progression, signs, or symptoms of the disease/disorder being studied, unless more severe than expected for the study participant's condition.<br>• Medical or surgical procedure (eg, endoscopy, appendectomy): the condition that leads to the procedure is the AE.<br>• Situations in which an untoward medical occurrence did not occur (social and/or convenience admission to a hospital).<br>• Anticipated day-to-day fluctuations of pre-existing disease(s) or condition(s) present or detected at the start of the study that do not worsen.

Definition of SAE

If an event is not an AE per definition above, then it cannot be an SAE even if serious conditions are met (eg, hospitalization for signs/symptoms of the disease under study, death due to progression of disease).

**An SAE is defined as any untoward medical occurrence that, at any dose:**
**a) Results in death**
**b) Is life-threatening**<br>The term 'life-threatening' in the definition of "serious" refers to an event in which the study participant was at risk of death at the time of the event. It does not refer to an event, which hypothetically might have caused death, if it were more severe.
**c) Requires inpatient hospitalization or prolongation of existing hospitalization**<br>In general, hospitalization signifies that the study participant has been detained (usually involving at least an overnight stay) at the hospital or emergency ward for observation and/or treatment that would not have been appropriate in the physician's office or outpatient setting. Complications that occur during hospitalization are AEs. If a complication prolongs hospitalization or fulfills any other serious criteria, the event is serious. When in doubt as to whether "hospitalization" occurred or was necessary, the AE should be considered serious.<br>Hospitalization for elective treatment of a pre-existing condition that did not worsen from baseline is not considered an AE.
**d) Results in persistent disability/incapacity**<br>• The term disability means a substantial disruption of a person's ability to conduct normal life functions.<br>• This definition is not intended to include experiences of relatively minor medical significance such as uncomplicated headache, nausea, vomiting, diarrhea, influenza, and accidental trauma (eg, sprained ankle) which may interfere with or prevent everyday life functions but do not constitute a substantial disruption.
**e) Is a congenital anomaly/birth defect**
**f) Other situations:**<br>• Medical or scientific judgment should be exercised in deciding whether SAE reporting is appropriate in other situations such as important medical events that may not be immediately life-threatening or result in death or hospitalization but may jeopardize the study participant or may require medical or surgical intervention to prevent 1 of the other outcomes listed in the above definition. These events should usually be considered serious.<br>Examples of such events include invasive or malignant cancers, intensive treatment in an emergency room or at home for allergic bronchospasm, blood dyscrasias or convulsions that do not result in hospitalization, or development of drug dependency or drug abuse.

Definition of an Adverse Event of Special Interest

An adverse event of special interest (serious or nonserious) is one of scientific and medical concern specific to the sponsor's product or program, for which ongoing monitoring and rapid communication by the investigator to the sponsor can be appropriate.

The following will be considered adverse events of special interest (AESI).

- Torsade de pointes
- Sudden death
- Ventricular tachycardia
- Ventricular fibrillation and flutter
- Syncope
- Seizures

Recording and Follow-up of AE and/or SAE

AE and SAE Recording
• When an AE/SAE occurs, it is the responsibility of the Investigator to review all documentation (eg, hospital progress notes, laboratory reports, and diagnostics reports) related to the event.<br>• The Investigator will then record all relevant AE/SAE information in the eCRF. Each event must be recorded separately.<br>• It is **not** acceptable for the Investigator to send photocopies of the study participant's medical records in lieu of completion of the AE/SAE eCRF page.<br>• There may be instances when copies of medical records for certain cases are requested by the Sponsor. In this case, all study participant identifiers, with the exception of the study participant number, will be redacted on the copies of the medical records before submission to the Sponsor.<br>• The Investigator will attempt to establish a diagnosis of the event based on signs, symptoms, and/or other clinical information. Whenever possible, the diagnosis (not the individual signs/symptoms) will be documented as the AE/SAE.
**Assessment of Intensity**
The Investigator will make an assessment of intensity for each AE and SAE reported during the study and assign it to 1 of the following categories:<br>• Mild: An event that is easily tolerated by the study participant, causing minimal discomfort, and not interfering with everyday activities.<br>• Moderate: An event that causes sufficient discomfort and interferes with normal everyday activities.<br>• Severe: An event that prevents normal everyday activities. An AE that is assessed as severe should not be confused with a SAE. Severe is a category utilized for rating the intensity of an event; and both AEs and SAEs can be assessed as severe.<br>An event is defined as 'serious' when it meets at least 1 of the predefined outcomes as described in the definition of an SAE, NOT when it is rated as severe.

Assessment of Causality
• The Investigator is obligated to assess the relationship between investigational product and each occurrence of each AE/SAE, prior to reporting. The AE must be characterized as unrelated, unlikely to be related, possibly related, probably related, or unknown (unable to judge).<br>o "Probably related" conveys that there are facts, evidence, and/or arguments to suggest a causal relationship, rather than a relationship cannot be ruled out.<br>o "Possibly related" suggests that the association of the AE with the investigational product is unknown; however, the AE is not reasonably supported by other conditions.<br>o "Unlikely to be related" suggests that only a remote connection exists between the investigational product and the AE. Other conditions, including chronic illness, progression or expression of the disease state or reaction to concomitant therapy, appear to explain the reported AE.<br>o "Unrelated" is used if there is not a reasonable possibility that the investigational product caused the AE.<br>o All efforts should be made to classify the AE according to the above categories. The category "unknown" (unable to judge) may be used only if the causality is not assessable, eg, because of insufficient evidence, conflicting evidence, conflicting data, or poor documentation.

• The Investigator will use clinical judgment to determine the relationship.<br>• Alternative causes, such as underlying disease(s), concomitant therapy, and other risk factors, as well as the temporal relationship of the event to investigational product administration will be considered and investigated.<br>• The Investigator will also consult the Investigator's Brochure and/or Product Information, for marketed products, in his/her assessment.<br>• For each AE/SAE, the Investigator must document in the medical notes that he/she has reviewed the AE/SAE and has provided an assessment of causality.<br>• There may be situations in which an SAE has occurred, and the Investigator has minimal information to include in the initial report. However, it is very important that the Investigator always make an assessment of causality for every event before the initial transmission of the SAE data.<br>• The Investigator may change his/her opinion of causality in light of follow-up information and send a SAE follow-up report with the updated causality assessment.<br>• The causality assessment is one of the criteria used when determining regulatory reporting requirements.

**Follow-up of AEs and SAEs**
• The Investigator is obligated to perform or arrange for the conduct of supplemental measurements and/or evaluations as medically indicated or as requested by Sponsor to elucidate the nature and/or causality of the AE or SAE as fully as possible. This may include additional laboratory tests or investigations, histopathological examinations, or consultation with other health care professionals.<br>• If a study participant dies during participation in the study or during a recognized follow-up period, the Investigator will provide the Sponsor with a copy of any postmortem findings including histopathology.<br>• New or updated information will be recorded in the originally completed eCRF.<br>• The Investigator will submit any updated SAE data to the Sponsor within 24 hours of receipt of the information.

Reporting of SAEs

**SAE Reporting to IQVIA Clinical Safety via an Electronic Data Collection Tool**
• The primary mechanism for reporting an SAE to IQVIA Clinical Safety will be the electronic data collection tool.<br>• If the electronic system is unavailable for more than 24 hours, then the study center will use the paper SAE data collection tool (see next section).<br>• The study center will enter the SAE data into the electronic system as soon as it becomes available.<br>• After the study is completed at a given study center, the electronic data collection tool will be taken off-line to prevent the entry of new data or changes to existing data.<br>• If a study center receives a report of a new SAE from a study participant or receives updated data on a previously reported SAE after the electronic data collection tool has been taken off-line, then the study center can report this information on a paper SAE form (see next section) or to the Medical Monitor/SAE coordinator by telephone.

SAE Reporting to IQVIA Clinical Safety via Paper eCRF
• Send an e-mail to IQVIA Safety at Safety_OZA78873@IQVIA.com or call (1-866-599-1341), and fax (1-866-599-1342)/e-mail the completed paper SAE form to IQVIA within 24 hours of awareness.<br>•<br>• In rare circumstances and in the absence of facsimile equipment, notification by telephone is acceptable with a copy of the SAE data collection tool sent by overnight mail or courier service.<br>• Initial notification via telephone does not replace the need for the Investigator to complete and sign the SAE eCRF pages within the designated reporting time frames.

Appendix 5 Excluded Medications/Therapy

Excluded medications/therapy is listed below in Table 20. The use of an excluded medication/therapy is a protocol violation and must be recorded in the eCRF.

The following were excluded/prohibited in the HVs-

- Use of any prescribed medications other than hormonal contraceptives within 30 days of investigational product administration, or less than 5 half-lives (whichever is longer). Female subjects using oral contraceptives may enroll.
- Use of over the counter medication excluding routine vitamins and acetaminophen but including megadose (intake of 20 to 600 times the recommended daily dose) vitamin therapy within 7 days of first dosing.
- Healthy volunteers who have participated in any investigational drug or device clinical trial within 3 months prior to first dosing on this study.
- Healthy volunteers who have previously participated in a study with an investigational product or device involving the dosing of a biological targeted at any immune pathway within 1 year prior to Screening.
- Healthy volunteers who have received the last dose of investigational product greater than 3 months ago but who are on extended follow-up.

The following were excluded/prohibited in the patients with AD or HS-

- Use of prescription or nonprescription drugs including topical corticosteroids more potent than hydrocortisone 1%, Crestor, vitaminic and dietary supplements within 5 half-lives, or within 28 days (whichever is shorter) prior to the first dose of investigational product.
- As an exception, acetaminophen/paracetamol may be used at doses of ≤1 g/day, only if necessary.
- Limited use of prescription or nonprescription medications that are not believed to affect patient safety or the overall results of the study may be permitted on a case-by-case basis following approval by the Sponsor.
- QTc prolonging medications included but not limited to those listed at www.crediblemeds.org are excluded within 5 half-lives prior to the first dose of investigational product through Day 28.
- Herbal supplements (including St. John’s Wort) must have been discontinued at least 28-days prior to the first dose of investigational product.

Table 20 Allowed and Excluded Medications

Allowed Medications in HVs	Excluded Medications in HVs
• Routine vitamins and acetaminophen excluding megadose (intake of 20 to 600 times the recommended daily dose)<br>• Female subjects using hormonal contraceptives	• Any prescribed medications within 30 days of investigational product administration, or less than 5 half-lives (whichever is longer)<br>• Over the counter medications within 7 days of first dosing<br>• Megadose (intake of 20 to 600 times the recommended daily dose) vitamin therapy within 7 days of first dosing<br>• Any investigational drug within 3 months prior to first dosing on this study<br>• Any investigational product involving the dosing of a biological targeted at any immune pathway within 1 year prior to Screening
**Allowed Medications in Patients with AD or HS**	**Excluded Medications in Patients with AD or HS**
• Acetaminophen/paracetamol may be used at doses of ≤1 g/day, only if necessary<br>• Herbal supplements (including St. John's Wort) allowed, but must have been discontinued at least 28-days prior to the first dose of investigational product<br>• Limited use of prescription or nonprescription medications used for treatment of conditions other an AD or HS that are not believed to affect patient safety or the overall results of the study may be permitted on a case-by-case basis following approval by the Sponsor	• Prescription or nonprescription drugs including topical corticosteroids more potent than hydrocortisone 1%, Crestor, vitaminic and dietary supplements for treatment of HS or AD and potential drug-drug interaction medications listed below, within 5 half-lives, or within 28 days (whichever is shorter) prior to the first dose of investigational product.<br>• Herbal supplements (including St. John's Wort) excluded 28-days prior to the first dose of investigational product and during the trial.<br>• Strong/moderate inhibitors and inducers of CYP3A4, P-glycoprotein, and BCRP.<br>• Narrow index drugs in which either CYP3A4 or CYP2C19 are mainly responsible for metabolism.<br>• Drugs (eg, rosuvastatin) where BCRP-mediated efflux (eg, rosuvastatin) is the major limiting factor of oral absorption.<br>• QTc prolonging medications included but not limited to those listed at www.crediblemeds.org.<br>• Any vaccine (including a COVID-19 vaccine or booster) if received 14 days prior to first dose or 14 days after last dose

Abbreviations: AD = Atopic dermatitis, BCRP = Breast cancer resistant protein, CYP = Cytochrome P450, HS = Hidradenitis suppurativa, HVs = Healthy volunteers

Appendix 6 Contraceptive Guidance and Collection of Pregnancy Information

Definitions:

Woman of Childbearing Potential (WOCBP)

A woman is considered fertile following menarche and until becoming postmenopausal unless permanently sterile (see below).

Women in the following categories are not considered WOCBP

1. Premenarchal
2. Premenopausal female with 1 of the following:
   a) Documented hysterectomy.
   b) Documented bilateral salpingectomy.
   c) Documented bilateral oophorectomy.
   Note: Documentation can come from the study center personnel's: review of the study participant's medical records, medical examination, or medical history interview.
3. Postmenopausal female:
   a) A postmenopausal state is defined as no menses for 12 months without an alternative medical cause. A high FSH level in the postmenopausal range may be used to confirm a postmenopausal state in women not using hormonal contraception or hormonal replacement therapy (HRT). However, in the absence of 12 months of amenorrhea, a single FSH measurement is insufficient.
   b) Females on HRT and whose menopausal status is in doubt will be required to use 1 of the non-estrogen hormonal highly effective contraception methods if they wish to continue their HRT during the study. Otherwise, they must discontinue HRT to allow confirmation of postmenopausal status before study enrollment.

All other female patients (including female patients with tubal ligations) are considered to be of childbearing potential.

4.

Contraception Guidance

Male study participants

- Male study participants with female partners of childbearing potential are eligible to participate if they agree to ONE of the following, until 90 days after the investigational product administration:
  - Agree to use a male condom and have their partner use one of the following contraceptive methods:

    - Barrier method of contraception: condoms (male or female) with or without a spermicidal agent, diaphragm or cervical cap with spermicide
    - IUD
    - Hormone-based contraceptive

- In addition, male study participants must refrain from donating sperm for the duration of the study and for 90 days after the last dose of investigational product.
- Male study participants with a pregnant or breastfeeding partner must agree to remain abstinent from penile-vaginal intercourse or use a male condom during each episode of penile penetration for 90 days after the last dose of investigational product.

Female study participants

- Female study participants of childbearing potential are eligible to participate if they agree to a combination of TWO of the following until 90 days after the investigational product administration:

  – Barrier method of contraception: condoms (male or female) with or without a spermicidal agent, diaphragm or cervical cap with spermicide
  – IUD
  – Hormone-based contraceptive

Pregnancy Testing:

- Pregnancy testing should be performed as indicated in Section 1.3.

Collection of Pregnancy Information

Male study participants with partners who become pregnant

- The Investigator will attempt to collect pregnancy information on any male study participant’s female partner who becomes pregnant while the male study participant is in this study. This applies only to male study participants who receive the investigational product.
- After obtaining the necessary signed informed consent from the pregnant female partner directly, the Investigator will record pregnancy information on the appropriate form and submit it to the Sponsor within 24 hours of learning of the partner’s pregnancy. The female partner will also be followed to determine the outcome of the pregnancy. Information on the status of the mother and child will be forwarded to the Sponsor. Generally, the follow-up will be no longer than 6 to 8 weeks following the estimated delivery date. Any termination of the pregnancy will be reported regardless of fetal status (presence or absence of anomalies) or indication for the procedure.

Female Study participants who become pregnant

- The Investigator will collect pregnancy information on any female study participant who becomes pregnant while participating in this study. Information will be recorded on the appropriate form and submitted to the Sponsor within 24 hours of learning of a study participant's pregnancy. The study participant will be followed to determine the outcome of the pregnancy. The Investigator will collect follow-up information on the study participant and the neonate, and the information will be forwarded to the Sponsor. Generally, follow-up will not be required for longer than 6 to 8 weeks beyond the estimated delivery date. Any termination of pregnancy will be reported, regardless of fetal status (presence or absence of anomalies) or indication for the procedure.
- While pregnancy itself is not considered to be an AE or SAE, any pregnancy complication or elective termination of a pregnancy will be reported as an AE or SAE. A spontaneous abortion is always considered to be an SAE and will be reported as such.
- Any post-study pregnancy related SAE considered reasonably related to the investigational product by the Investigator will be reported to the Sponsor as described in Section 6.3.4. While the Investigator is not obligated to actively seek this information in former study participants, he or she may learn of an SAE through spontaneous reporting.
- Any female study participant who becomes pregnant while participating in the study will be discontinued and followed up till there is an outcome.

Appendix 7 Food Effect Meal Composition

Breakfast Composition

		Fat		
**Meal Type**	**Total Kcal**	**Kcal**	**Grams**	**Percent**
High-Fat*	800-1000	500-600	55-65	50
**Standard**	**500-800**	**150-240**	**17-27**	**30**
Low-Fat*	400-500	100-125	11-14	25

*FDA Guidance 2019

Composition of a Standard Breakfast

**Total Calories**	500-800
**Calories from Protein**	80
**Calories from Carbohydrates**	314
**Calories from Fat**	150-240
Example:<br>Two Pancakes<br>One Pkt Pancake Syrup<br>One Boiled Egg<br>½ Cup Peaches<br>8oz 2% Milk	

Appendix 8 Signature of Investigator

PROTOCOL TITLE: A Phase 1 randomized, placebo-controlled, single and multiple ascending dose trial to evaluate the safety, tolerability, pharmacokinetics, and pharmacodynamics of orally administered KT-474 in healthy adult volunteers and patients with atopic dermatitis (AD) or hidradenitis suppurativa (HS)

PROTOCOL NO: KT474-HV-101

VERSION: Version 9.0, 05 August 2022

This protocol is a confidential communication of Kymera Therapeutics, Inc. I confirm that I have read this protocol, I understand it, and I will work according to this protocol. I will also work consistently with the ethical principles that have their origin in the Declaration of Helsinki and that are consistent with Good Clinical Practices and the applicable laws and regulations. Acceptance of this document constitutes my agreement that no unpublished information contained herein will be published or disclosed without prior written approval from the Sponsor.

Instructions to the Investigator: Please SIGN and DATE this signature page. PRINT your name, title, and the name of the study center in which the study will be conducted. Return the signed copy to Sponsor.

I have read this protocol in its entirety and agree to conduct the study accordingly:

Signature of Investigator: ___________________________ Date: __________

Printed Name: ____________________________

Investigator Title: ____________________________

Name/Address of Center: ____________________________

____

____
