# Supplementary material for: IRAK4 degrader in hidradenitis suppurativa and atopic dermatitis: a phase 1 trial
Source: Nat Med. 2023 Nov 13;29(12):3127–36. doi: 10.1038/s41591-023-02635-7 (PMC10719089; doi:10.1038/s41591-023-02635-7)
Supplement: Supplementary file 2 — Reporting Summary [file 41591_2023_2635_MOESM2_ESM.pdf]

nature portfolio

Corresponding author(s): Jared A. Gollob

Last updated by author(s): 2023/09/26

Reporting Summary

Nature Portfolio wishes to improve the reproducibility of the work that we publish. This form provides structure for consistency and transparency in reporting. For further information on Nature Portfolio policies, see our Editorial Policies and the Editorial Policy Checklist.

Statistics

For all statistical analyses, confirm that the following items are present in the figure legend, table legend, main text, or Methods section.

n/a	Confirmed	
☐	☒	The exact sample size (*n*) for each experimental group/condition, given as a discrete number and unit of measurement
☐	☒	A statement on whether measurements were taken from distinct samples or whether the same sample was measured repeatedly
☐	☒	The statistical test(s) used AND whether they are one- or two-sided<br>*Only common tests should be described solely by name; describe more complex techniques in the Methods section.*
☒	☐	A description of all covariates tested
☒	☐	A description of any assumptions or corrections, such as tests of normality and adjustment for multiple comparisons
☐	☒	A full description of the statistical parameters including central tendency (e.g. means) or other basic estimates (e.g. regression coefficient) AND variation (e.g. standard deviation) or associated estimates of uncertainty (e.g. confidence intervals)
☐	☒	For null hypothesis testing, the test statistic (e.g. *F*, *t*, *r*) with confidence intervals, effect sizes, degrees of freedom and *P* value noted<br>*Give P values as exact values whenever suitable.*
☒	☐	For Bayesian analysis, information on the choice of priors and Markov chain Monte Carlo settings
☒	☐	For hierarchical and complex designs, identification of the appropriate level for tests and full reporting of outcomes
☒	☐	Estimates of effect sizes (e.g. Cohen's *d*, Pearson's *r*), indicating how they were calculated

Our web collection on statistics for biologists contains articles on many of the points above.

Software and code

Policy information about availability of computer code

Data collection	Raw proteomics data were processed with MaxQuant52 (version 1.6.14.0) and searched with the Andromeda53 search engine against a comprehensive SwissProt database release for human. Concentrations of KT-474 in plasma and urine were measured using validated liquid chromatography mass spectrometry (LC/MS/MS) assays with a lower limit of quantification (LLOQ) of 0.150 ng/mL and a quantitation range of 0.150 to 200 ng/mL. Targeted mass spectrometry (LC-PRM-MS) was performed at Biognosys AG on isolated PBMCs collected from blood samples (6 mL K2EDTA) or frozen skin biopsies. Preclinical cytokines were collected using Discovery workbench v4.0. Preclinical FLOW data was collected using Attune cytometric software v5.3.0. FLOW cytometry data were acquired using the BD FACSDiva V9.0 software and stored in the format of flow cytometry standard (*.fcs) files on a secured read-only “Raw Data” server. The TruCulture system with Optimap detection is a commercially available and clinically validated system that was developed by RBM, Austin Texas, USA. Libraries were sequenced on an Illumina NextSeq 500 sequencer.

Data analysis	All statistical analyses were performed with SAS Version 9.4. A paired statistical analysis was performed using the limma R package. The concentration of cytokines in supernatant was determined using MSD Discovery Workbench v4.0 software. Prism Graphpad v9.5.1 was used to generate IC50's using a 4-parameter logistic regression curve, free-fit. B-cell phospho FLOW data were analyzed using FlowJoTM v10.8.0. PK parameters for KT-474 were estimated from plasma and urine concentration data via noncompartmental analysis using Phoenix WinNonlin (v. 8.3.2; Certara, St. Louis, MO). Signal processing and data analysis were carried out using SpectroDrive software. Peak integration, retention time alignment and peak scoring was passed on mProphet and automated peak integration was verified by trained personnel for all experiments. FLOW cytometry data were analyzed using the CellEngine v2.0 templates established during development of the study. Raw RNA sequencing data were processed by the standard Illumina software package bcl2fastq to demultiplex the reads and perform base-calling. Differential expression analysis was performed using sleuth. Analytical pipelines were implemented using snakemake. R 4.2.0 < https://www.r-project.org> and the R tidyverse 2.0.0 libraries <https://www.tidyverse.org> were used to generate figures.

For manuscripts utilizing custom algorithms or software that are central to the research but not yet described in published literature, software must be made available to editors and reviewers. We strongly encourage code deposition in a community repository (e.g. GitHub). See the Nature Portfolio guidelines for submitting code & software for further information.

Data

Policy information about availability of data

All manuscripts must include a data availability statement. This statement should provide the following information, where applicable:

- Accession codes, unique identifiers, or web links for publicly available datasets
- A description of any restrictions on data availability
- For clinical datasets or third party data, please ensure that the statement adheres to our policy

Data Availability Statement
The clinical data that support the findings of this study are not openly available due to reasons of sensitivity and are available from the corresponding author (jared@kymeratx.com) upon reasonable request. RNAseq data is available at the NCBI Sequence Read Archive as BioProject number PRJNA1003129 (https://dataview.ncbi.nlm.nih.gov/object/PRJNA1003129?reviewer=o2jfesi1s6rtlgcsoofigfnft5).Requests will be responded to within 3 weeks of receipt.

Research involving human participants, their data, or biological material

Policy information about studies with human participants or human data. See also policy information about sex, gender (identity/presentation), and sexual orientation and race, ethnicity and racism.

Reporting on sex and gender	Sex is used to describe the biological attribute. Sex was self-reported. Sex- and gender-based analyses have not been performed because the sample size was insufficient to determine sex/gender-based differences in this Phase 1 study.
Reporting on race, ethnicity, or other socially relevant groupings	Race and ethnicity was self reported based on available groups (White/Hispanic, Latino; White/non- Hispanic, Latino; Black/Hispanic, Latino; Black/non-Hispanic, Latino; Other [Asian/Hispanic, Latino; Asian/non-Hispanic, Latino]).
Population characteristics	Key eligibility criteria for HVs included males or females aged 18 to 55 years without clinically relevant medical histories or presence of clinically relevant medical disorders. Subjects who had used any prescribed medications other than hormonal contraceptives within 30 days or five half-lives (whichever was longer) of KT-474 administration were excluded.<br><br>Key eligibility criteria for AD or HS patients included males or females aged 18 to 75 years with body mass index of 17.5 to 40.0 kg/m2 who weighed >45 kg and who were generally in good health. AD patients must have had involvement of ≥10% treatable body surface area excluding the scalp and designated venous access areas at screening or on admission, and HS patients must have had a Total AN count ≥4 at baseline with fistula and tunnel count <20. Patients who had received prescription or non-prescription drugs for the treatment of AD or HS (including corticosteroids more potent than hydrocortisone 1% and vitamin and dietary supplements) within five half-lives or within 28 days (whichever is shorter) prior to first dose of KT-474 were excluded.
Recruitment	Participants were recruited by the clinical trial sites and were selected based on their commitment to follow all protocol requirements and who were motivated to participate in a clinical trial. Participants were recruited by the clinical trial sites through own databases of available participants as well as with online and print advertisements. Since all participants were also required to meet eligibility criteria in the protocol, it is unlikely that potential self-selection bias or other biases that were present significantly impacted the results of the trial.
Ethics oversight	All participants were required to provide informed consent before participating in the trial.<br>Central IRB used for this study:<br><br>Advarra IRB<br><br>6100 Merriweather Drive<br><br>Suite 600<br><br>Columbia, Maryland 21044<br><br>IRB Chair Director: Sara Harnish

Note that full information on the approval of the study protocol must also be provided in the manuscript.

Field-specific reporting

Please select the one below that is the best fit for your research. If you are not sure, read the appropriate sections before making your selection.

☒ Life sciences ☐ Behavioural & social sciences ☐ Ecological, evolutionary & environmental sciences

For a reference copy of the document with all sections, see nature.com/documents/nr-reporting-summary-flat.pdf

Life sciences study design

All studies must disclose on these points even when the disclosure is negative.

Sample size	Sample size was based on practical considerations as efficacy analyses were considered exploratory for this study. There were 8 HVs in each cohort (6 active: 2 placebo) of SAD/FE, 12 HVs in each cohort (9 active: 3 placebo) for MAD, and up to 30 patients with AD or HS in the patient cohort . This was considered to be sufficient for evaluation of safety, tolerability, PK and PD data of each cohort.
Data exclusions	Dropouts and study participants withdrawn due to protocol violations were to be replaced following discussion with the Investigator and Sponsor.
Replication	Initial phase 1 study results have been reported in this manuscript. Further studies are warranted.<br><br>A validation study was performed at Cell Carta (the clinical testing laboratory) where the following assay qualification parameters were established: intra-assay precision, inter-operator precision, inter-instrument precision, post-draw stability and post processing stability. Each of these parameters passed the recommended assay acceptance criteria as defined by Cell Carta.<br><br>A validation study was performed at Cell Carta (the clinical testing laboratory) where the following assay qualification parameters were established: Intra-Assay Precision (3 donors, 3 replicates performed on a single day), Inter-Operator Precision (3 donors, 3 replicates performed on a single day), Inter-Instrument Precision (1 donor, 3 replicates over 2 days), Post-Draw Stability (3 donors, 3 runs over 4 days) and Post-Processing Stability (3 donors, 3 replicates over 4 days). The recommended acceptance criteria for assay qualifications was the following: Intra-Assay Precision was defined as %CV≤25% across replicates for at least 2 of 3 donors for cellular subsets/readouts with abundance greater than 1% of total PBMC/WBC or greater than 250 MFI value. Inter-Operator Precision was defined as %CV≤25% across all samples analyzed by both operators for at least 2 of 3 donors for cellular subsets/readouts with abundance greater than 1% of total PBMC/WBC or greater than 250 MFI value. Inter-Instrument precision was %CV≤25% across all samples analyzed across two instruments by one operator for cellular subsets/readouts with abundance greater than 1% of total PBMC/WBC or greater than 250 MFI value. Post-Draw Stability was W%D of replicates for at least 2 of 3 donors each time point is ≤ 25% for cellular subsets and readouts with percentage higher than 1% of total PBMC/WBC or greater than 250 MFI value. Post Process Stability was W%D of replicates for at least 2 of 3 donors each time point is ≤ 25% for cellular subsets and readouts with percentage higer than 1% of total PBMC/WBC or greater than 250 MFI value. All attempts at replication passed qualification. For additional details, please see the qualification report 6012 (Supplementary Information).
Randomization	For HV SAD: Within each cohort, 6 HVs were randomized in a double-blind manner to receive KT-474 and 2 HVs were randomized to receive placebo. At each dose level, 2 sentinel HVs (1 receiving KT-474 and 1 receiving placebo) were administered the investigational product first.<br>For each cohort, the HVs received either a single dose of KT-474 (n = 6) or placebo (n = 2) in a double-blind manner according to the randomization scheme. Sequential dosing of the HVs within a cohort was staggered so that there was at least a gap of 10 minutes between dosing of individual HVs. For each cohort, HVs randomized to placebo received the same number of tablets as HVs randomized to KT-474.<br><br>HV MAD had up to 4 cohorts randomized in a 9:3 ratio (KT-474 versus placebo) for a total of up to 48 HVs.<br><br>HS/AD Patient cohort was open-label, therefore, no randomization was necessary.
Blinding	Randomization occured individually, the randomization code (and the associated treatment) was assigned to the unique Subject Identification Number of each randomized study participant. The study was double-blind with limited access to the randomization code.

Reporting for specific materials, systems and methods

We require information from authors about some types of materials, experimental systems and methods used in many studies. Here, indicate whether each material, system or method listed is relevant to your study. If you are not sure if a list item applies to your research, read the appropriate section before selecting a response.

Materials & experimental systems

n/a	Involved in the study
	☒ Antibodies
	☒ Eukaryotic cell lines
☒	Palaeontology and archaeology
☒	Animals and other organisms
	☒ Clinical data
	Dual use research of concern
☒	Plants

Methods

n/a	Involved in the study
☒	ChIP-seq
	☒ Flow cytometry
☒	MRI-based neuroimaging

Antibodies

Antibodies used

Fluorescently tagged antibody (phycoerythrin [PE] phospho-p65, clone K10-895.12.50 (#558423, BD Biosciences); a pre-permeabilization antibody cocktail (CD14 [1:25], CD56 [1:50], CD19 [1:100], CD3 [1:200], CD4 [1:200], CD8 [1:200], CD45 [1:200], CD15 [1:200]); IRAK4 unconjugated antibody [1:20]; post-perm antibody cocktail (CD16 [1:200], IRAK4 [1:20])

Validation

Cell surface antibody staining was validated in human whole peripheral cells compared to the appropriate antibody isotype negative control (manufacturer validation)

For the IRAK4 L29-525 a human cell line (Hela S3) was transfected with IRAK4 siRNA to demonstrate antibody specificity by flow and western (manufacturer validation)

CD14 BUV395 antibody by BD Biosciences, clone MφP9, catalog 563561, Manufacturer's validation: CD14 staining was demonstrated in monocytes from human whole blood compared to negative staining using mouse IgG2b, K isotype control

CD16 PE antibody by BioLegend, clone B73.1, catalog 360704, Manufacturer's validation: CD16 staining was demonstrated in lymphocytes from human whole blood compared to negative staining using mouse IgG1, K isotype control

CD56 BV711 antibody by BioLegend, clone HCD56, catalog 318336, Manufacturer's validation: CD56 staining was demonstrated in lymphocytes from human whole blood compared to negative staining using mouse IgG1, K isotype control

CD19 BV786 antibody by BD Biosciences, clone SJ25-C1, catalog 563325, Manufacturer's validation: CD19 staining was demonstrated in monocytes from human whole blood compared to negative staining using mouse IgG1, K isotype control

CD3 Pacific Blue antibody by BioLegend, clone UCHT1, catalog 300417, Manufacturer's validation: CD3 staining was demonstrated in lymphocytes from human whole blood compared to negative staining using mouse IgG1, K isotype control

CD4 Ax700 antibody by BioLegend, clone RPA-T4, catalog 300526, Manufacturer's validation: CD4 staining was demonstrated in lymphocytes from human whole blood compared to negative staining using mouse IgG1, K isotype control

CD8 FITC antibody by BioLegend, clone RPA-T8, catalog 301060, Manufacturer's validation: CD8 staining was demonstrated in lymphocytes from human whole blood compared to negative staining using mouse IgG1, K isotype control

CD45 BUV805 antibody by BD Biosciences, clone HI30, catalog 564914, Manufacturer's validation: CD45 staining was demonstrated in leukocytes from human whole blood compared to negative staining using mouse IgG2a, K isotype control

IRAK4 Ax647 antibody by BD Biosciences, clone L29-525, catalog 560315, Manufacturer's validation: Hela S3 human cells transfected with IRAK4 siRNA or untreated with stained by flow and by western to confirm specificity of L29-525 antibody

CD15 PE-Cy7 antibody by BioLegend, clone W6D3, catalog 323030, Manufacturer's validation: CD15 staining was demonstrated in granulocytes from human whole blood compared to negative staining using mouse IgG1, K isotype control

Eukaryotic cell lines

Policy information about cell lines and Sex and Gender in Research

Cell line source(s)

isolated human PBMCs (AllCells); CD19+ B cells were purchased from BioIVT or isolated in-house using Stemcell's negative selection kit (#17954); participant blood and skin samples

Authentication

None of the cell lines were authenticated

Mycoplasma contamination

None of the cell lines were tested for mycoplasma contamination

Commonly misidentified lines
(See ICLAC register)

NA

Clinical data

Policy information about clinical studies

All manuscripts should comply with the ICMJE guidelines for publication of clinical research and a completed CONSORT checklist must be included with all submissions.

Clinical trial registration	NCT04772885
Study protocol	Uploaded as material available for review
Data collection	Between February 4, 2021 and September 7, 2022, 105 healthy volunteers (HVs) were enrolled into the placebo-controlled single and multiple ascending dose escalation cohorts (SAD and MAD) and 21 HS and AD patients were enrolled into the open-label patient cohort. Data was collected at the respective clinical sites. Data were collected at 2 phase 1 research centers that recruited HVs and at the principal investigators' offices where the HS and AD patients were enrolled.

Outcomes

Primary Objective: To determine the safety and tolerability of KT-474 when administered as single and multiple oral doses at escalating dose levels in HVs and as multiple doses in patients with AD or HS. Assessed by treatment emergent (serious) adverse events, concomitant medications, clinical laboratory tests, vital signs, and safety ECG and Holter monitoring.
Secondary Objectives: To characterize the PK profile of KT-474 and its diastereomers KT-5481 and KT 5482, following single and multiple doses of KT-474 in HVs and following multiple doses in patients with AD or HS. Assessed by: plasma and urine PK parameters of KT-474, KT-5481, and KT-5482.

Exploratory Objectives:
• To characterize the PD profile of KT-474 following single and multiple doses in HVs and following multiple doses in patients with AD or HS.
• To characterize the concentration of KT-474 in skin following multiple doses in HVs and patients with AD or HS.
• To evaluate the effect of food on the PK profile of KT-474 and its diastereomers KT-5481 and KT-5482 following a single dose of KT-474 in HVs.
• To evaluate the metabolite profile of KT-474 following multiple doses of KT-474 in HVs.
• To assess blood and skin for messenger ribonucleic acid (mRNA) for candidate biomarkers following multiple doses of KT-474 in HV and patients with AD or HS.
• To assess preliminary efficacy in patients with AD and HS.

Flow Cytometry

Plots

Confirm that:

☒ The axis labels state the marker and fluorochrome used (e.g. CD4-FITC).

☒ The axis scales are clearly visible. Include numbers along axes only for bottom left plot of group (a 'group' is an analysis of identical markers).

☒ All plots are contour plots with outliers or pseudocolor plots.

☒ A numerical value for number of cells or percentage (with statistics) is provided.

Methodology

Sample preparation	Whole blood samples were collected in Na-Hep tubes at the clinical site and shipped on the day of collection at 4°C. Upon receipt, samples were lysed/fixed and stored at -80°C until processing. Lyse/fixed samples were divided into plates for unblocked and blocked conditions, stained with a pre-permeabilization antibody cocktail (CD14, CD56, CD19, CD3, CD4, CD8, CD45, CD15) for 30 minutes at RT, washed, permeabilized with 60% methanol at 4°C for 10 minutes, followed by incubation with IRAK4 unconjugated antibody (blocking condition) or Bovine Serum Albumin (unblocked) for 30 minutes at RT. Finally, samples were stained with post-perm antibody cocktail (CD16, IRAK4) for 30 minutes at RT, washed and processed.
Instrument	BD LSR Fortessa flow cytometer
Software	FLOW cytometry data were acquired using the BD FACSDiva software and stored in the format of flow cytometry standard (*.fcs) files on a secured read-only “Raw Data” server. Data were analyzed using the CellEngine templates established during development of the study.
Cell population abundance	1. Review of sample collection over time (exclusion of fluidic instability);<br>2. Exclusion of doublets using FSC-A vs FSC-H, and SSC-A vs SSC-H;<br>3. WBC selection based on SSC-A and FSC-A;<br>4. Exclusion of Neutrophils using CD16 vs CD15 staining;<br>5. PBMC selection based on CD45 marker

Gating strategy

Cytometer settings and compensations were performed as per CellCarta's internal procedures.
The following gating strategy was utilized for the flow samples to identify PBMC populations from the clinical samples (note a figure with depiction of gating strategy is provided) :
1. Review of sample collection over time (exclusion of fluidic instability);
2. Exclusion of doublets using FSC-A vs FSC-H, and SSC-A vs SSC-H;
3. WBC selection and gating was based on SSC-A and FSC-A distribution;
4. Exclusion of Neutrophils using CD16 vs CD15 staining (gate out double positive CD16+CD5+ population);
5. To identify the PBMC population the double negative (CD16-CD15-) population was then gated by CD45+ marker

☒ Tick this box to confirm that a figure exemplifying the gating strategy is provided in the Supplementary Information.
